# Synthesis of (P^N^C)Gold(III) Complexes via Tandem Oxidative Addition/C–H Auration

**DOI:** 10.1021/acsorginorgau.5c00057

**Published:** 2025-08-23

**Authors:** Michał Biedrzycki, Jaime Martín, Alexandre Genoux, Elia Boschi, Cristina Nevado

**Affiliations:** Department of Chemistry, 27217 University of Zurich, Winterthurerstrasse 190, Zurich CH 8057, Switzerland

**Keywords:** gold, ligands, oxidative addition, cyclometalation, phosphorescence

## Abstract

A new method for the synthesis of cyclometalated gold­(III) complexes featuring (P^N^C) ligands is reported. This protocol employs a tandem oxidative addition/cycloauration process, enabling the formation of (P^N^C)­gold­(III)-aryl, -alkenyl and -alkynyl derivatives by creating two Au–C bonds in one pot from a structurally diverse range of C­(sp^2^)- and C­(sp)-iodide substrates. Additionally, a complementary two-step method was developed to access (P^N^C)­gold­(III)-alkynyl complexes, which exhibit emission in the blue and green light regions.

## Graphical abstract

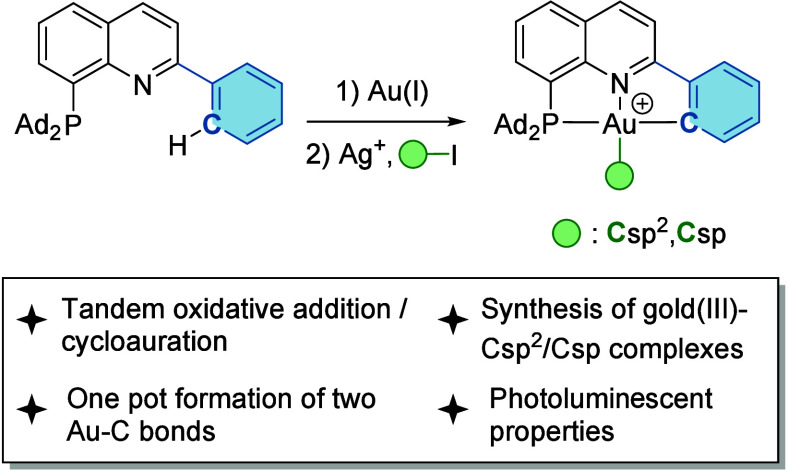

Gold­(III) complexes have emerged as versatile tools in catalysis,^1–4^ medicine^5–8^ and material science.^9–12^ One of the major challenges to advance these fields is to stabilize gold in high oxidation states, given its high redox potentials (E_red_^Au(III)/Au(I)^ = +1.41 V and E_red_^Au(III)/Au(0)^ = +1.50 V in aqueous solution)^13^ and its subsequent propensity to undergo reductive elimination. Bi- and tridentate ligands, predominantly featuring carbon and nitrogen donor groups, present an effective strategy to modify the redox properties of the metal and stabilize gold­(III) via cyclometalation.^14^ Interestingly, these complexes exhibit unique catalytic performances,^15–19^ promising antitumoral activities,^20–24^ and versatile photochemical properties.^25–28^

Seminal work by Constable on the synthesis of cyclometalated (N^C)­gold­(III) compounds via transmetalation from organomercury precursors^29^ inspired the widespread incorporation of (N^C) ligands into gold by innovative synthetic methods. Some of these involve oxidative addition of aryl diazonium salts and aryl iodides to gold­(I) precursors, and direct cycloauration under microwave and high temperature conditions (Figure 1, top left).^30–34^ The introduction of an additional phenyl ring to access biscyclometalated (N^C^C)­gold­(III) derivatives^35–42^ was attained by our group using microwave irradiation (Figure 1, top right).^35^

**Figure 1:**
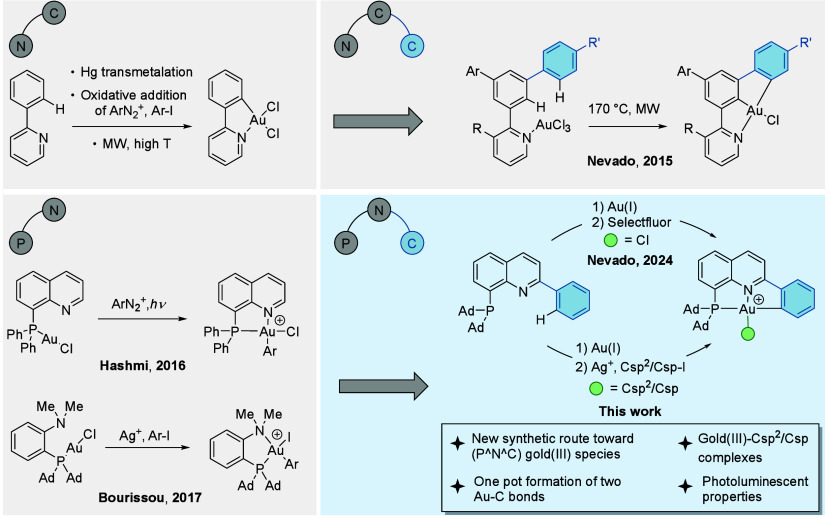
Evolution of bidentate to tridentate gold­(III) complexes. Top: from (N^C) to (N^C^C)­gold­(III) complexes. Bottom: from (P^N) to (P^N^C)­gold­(III) complexes via oxidation with Selectfluor and tandem oxidative addition/C–H auration (this work).

Bidentate (P^N) ligands, featuring both soft and hard coordination sites, represent an appealing alternative to conventional (N^C) templates for the stabilization of gold species^43–47^ and have proved fruitful in the implementation of gold­(I)/gold­(III) redox catalytic cycles.^48–54^ (P^N)­gold­(III) complexes are typically synthesized by coordination of a gold­(I) precursor to the phosphine group followed by oxidative addition of aryl diazonium salts upon blue light irradiation^55,56^ or of aryl iodides in the presence of silver salts (Figure 1, bottom left).^57^

Recently, our group disclosed the extension of these systems with a straightforward synthesis of (P^N^C) pincer ligands.^58,59^ Coordination of the phosphine group to gold­(I), followed by oxidation with Selectfluor enabled the cycloauration step delivering the corresponding (P^N^C)­gold­(III) chlorides (Figure 1, bottom right). These species underwent facile exchange of the chloro ligand, enabling the structural characterization of elusive gold­(III) hydride, hydroxo and formate species.

We hypothesized that the κ^1^-P coordination of (P^N^C) ligands to a soft gold­(I) center would stabilize a cationic gold intermediate which, upon oxidative addition of aryl-halides, would facilitate the coordination of the N quinoline group and trigger the concomitant Csp^2^-H bond activation of the phenyl ring delivering the cyclometalated species.

Herein, we report the successful implementation of this concept showcasing the synthesis of (P^N^C)­gold­(III)-aryl, -alkenyl and -alkynyl derivatives via oxidative addition of Csp^2^-I and Csp-I bonds onto (P^N^C)­gold­(I) precursors. This sequence results in the formation of tridentate (P^N^C)­gold­(III) complexes in the absence of external oxidants, following a tandem oxidative addition/cycloauration mechanism featuring the concomitant formation of two new Au–C bonds in one pot. Additionally, the photoluminescent properties of (P^N^C)­gold­(III)-alkynyl complexes were investigated, revealing emission maxima in the blue and green spectral regions.

The (P^N^C) ligands were synthesized by using 2,8-dibromoquinoline as a precursor, introducing the additional aryl groups via selective Suzuki cross-coupling with arylboronic acids at the C2 position of the quinoline. The resulting 2-aryl-8-bromoquinolines were functionalized with the corresponding diadamantyl and ditertbutylphosphines through a Pd-catalyzed phosphorylation step, yielding the final tridentate (P^N^C) ligands **I**–**VI**.^58,60^

The reaction of ligand **I** with AuCl­(DMS) followed by treatment with 1-fluoro-4-iodobenzene in the presence of AgSbF_6_ led to the formation of the (P^N^C)­gold­(III)-aryl complex **1a** in 68% isolated yield (Figure 2). The ^31^P­{^1^H} NMR spectrum showed a singlet at 63.9 ppm, confirming the oxidation of the metal center. Additionally, X-ray diffraction analysis of **1a** confirmed the κ^3^-P^N^C coordination of the pincer ligand, with a P1–Au1–C7 bite angle of 164.15(8)°, consistent with a distorted square planar geometry around gold, and a short Au1–C1 distance of 2.049(3) Å, in agreement with the weak *trans* influence of the quinoline nitrogen. (P^N^C) ligand **II**, featuring *tert*-butyl substituents on the phosphine group, displayed similar reactivity, delivering the corresponding (P^N^C)­gold­(III)-aryl complex **1b** in 56% yield. The scope and versatility of this method was explored next. Oxidative addition of *para*-substituted phenyl-iodides containing electron-donating (OMe) and electron-withdrawing (CF_3_) groups furnished the corresponding (P^N^C)­gold­(III)-aryl complexes in good yields (**1c**, 57%; **1d**, 61%). Substitution at the 4-position of the phenyl ring of the (P^N^C) ligand with OMe and CF_3_ groups produced the corresponding cyclometalated complexes in excellent yields (**1e**, 87%; **1f**, 80%). Finally, when the 3-position of the phenyl ring was substituted with OMe or CF_3_ groups, the less sterically congested regioisomers were exclusively obtained (**1g**, 77%; **1h**, 75%).

**Figure 2:**
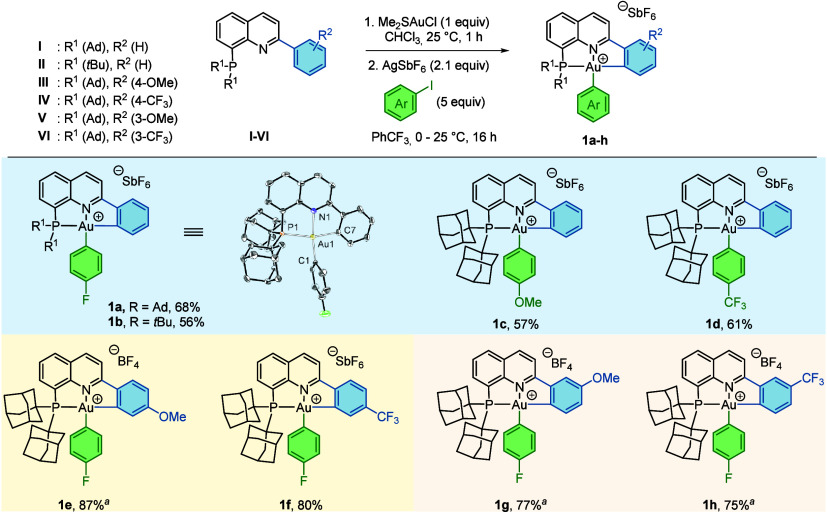
Synthesis of cyclometalated (P^N^C)­Au­(III)­aryl complexes **1a**–**h** by oxidative addition of aryl iodides. ORTEP representation of **1a** with 50% probability ellipsoids. The counteranion and hydrogen atoms are omitted for clarity. Selected bonds (Å): Au1–C1 2.049(3), Au1–C7 2.057(3), Au1–N1 2.071(2), Au1–P1 2.3932(7); selected angles (deg): C1–Au1–N1 171.07(11), C7–Au1–P1 164.15(8). ^*a*^AgBF_4_ was used instead of AgSbF_6_.

This methodology was also compatible with other Csp^2^-based electrophiles (Figure 3). When 1 equiv of vinyl iodide was used in combination with AgBF_4_, the corresponding (P^N^C)­gold­(III)-vinyl complex **2a** was obtained in 99% yield. Furthermore, oxidative addition of 1,2-disubstituted alkenyl iodides proceeded with retention of the configuration, as demonstrated by the reactions of (*E*)-β-iodostyrene and (*Z*)-β-iodostyrene, which produced the corresponding (P^N^C)­gold­(III)-alkenyl complexes **2b** and **2c** in 82 and 96% yields, respectively. The ^1^H NMR spectrum of **2b** in dichloromethane-*d*_2_ at 298 K exhibited a doublet of doublets at 7.16 ppm (*J*_HH_ = 16.2 Hz, *J*_HP_ = 1.7 Hz) corresponding to a vinylic proton, consistent with a *trans* configuration of the double bond. In contrast, the vinylic signals of **2c** overlapped with the aromatic region of the (P^N^C) ligand, while the two adamantyl groups became inequivalent, unlike in compound **2b**. This observation suggests that the alkenyl group in **2c** lies perpendicular to the (P^N^C)Au moiety due to steric constraints imposed by the double bond configuration, in line with previous hydroauration products reported for this ligand system.^59^

**Figure 3:**
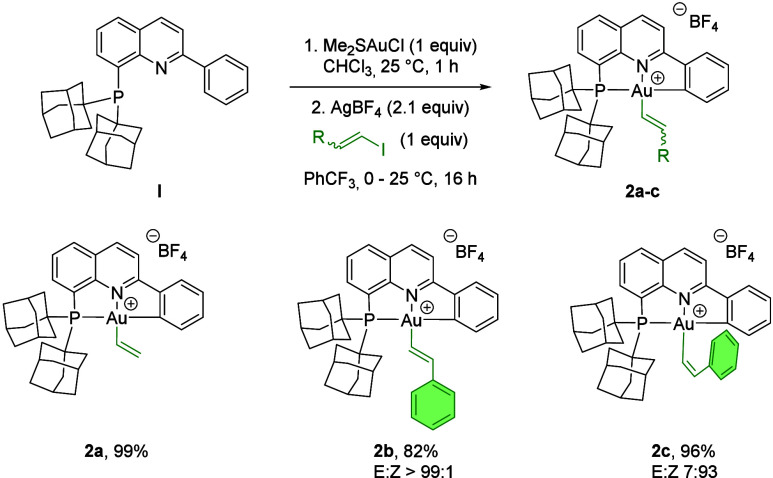
Synthesis of cyclometalated (P^N^C)­Au­(III)­alkenyl complexes **2a**–**c** by oxidative addition of alkenyl iodides.

The excellent stability of these new (P^N^C)­gold­(III) complexes^60^ prompted us to investigate their photophysical behavior. Tridentate (C^N^C) and (N^C^C)­gold­(III)-alkynyl complexes are known for their remarkable emissive properties and some have been used as dopants in OLED devices.^26,61^ Interestingly, the tandem oxidative addition/cycloauration protocol with phenylethynyl iodide and silver tetrafluoroborate allowed us to prepare the corresponding (P^N^C)­gold­(III)-alkynyl compound **4a**, demonstrating versatility of this method to incorporate not only C­(sp^2^)- but also C­(sp)-I based reagents (Figure 4, top).^60^ However, the isolation of gold­(III) complexes with more complex alkynyl iodides using this approach proved challenging, prompting the development of an alternative route via a gold­(III)-halide precursor.^25,26,61^ Thus, using (P^N^C)­gold­(III)-chloride precursor **3**,^58^ a copper-mediated transmetalation process with various terminal alkynes delivered the corresponding alkynyl derivatives **4a**, **4b**, **4c** and **4d** in 90, 95, 74, and 75% yields, respectively (Figure 4, top).

**Figure 4:**
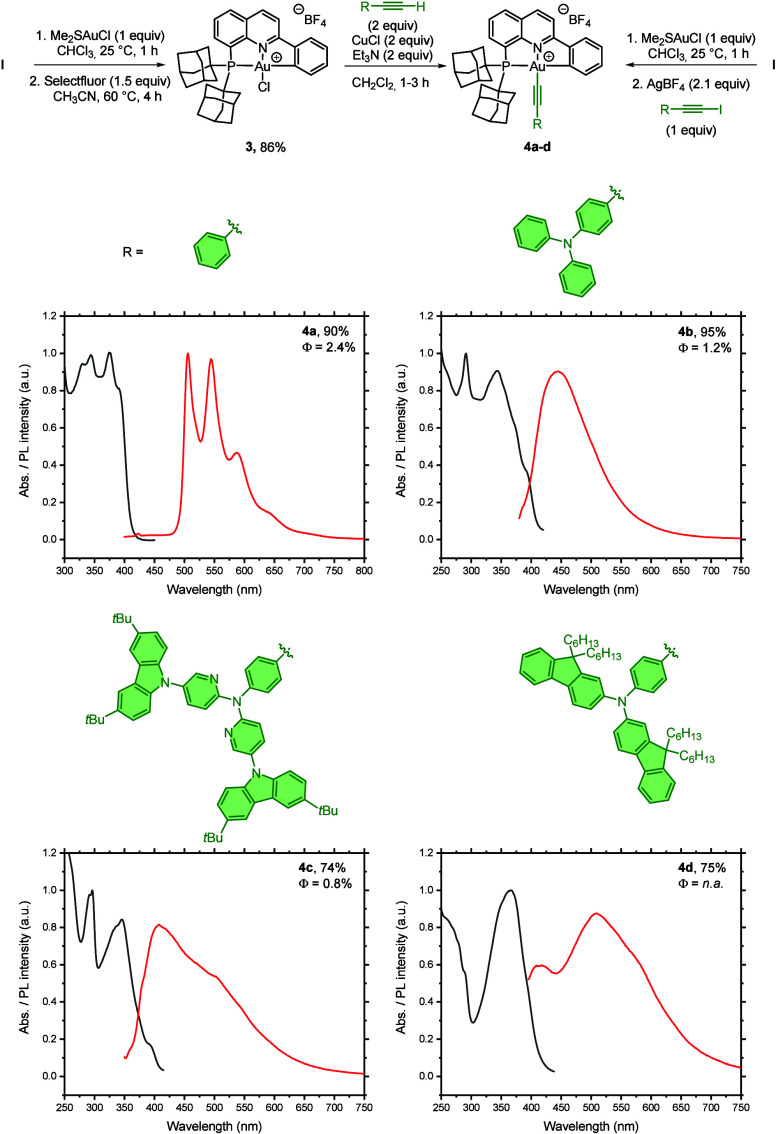
Synthesis of (P^N^C)­Au­(III)-alkynyl complexes **4a**–**d**. Absorption (black) and emission (red) spectra in dichloromethane.

Complex **4a** emitted green light with maxima at 506, 545, and 588 nm when excited at λ ≤ 375 nm in degassed dichloromethane at room temperature (quantum yield φ­(CH_2_Cl_2_) = 2.4%). In contrast, compounds **4b** and **4c** exhibited blue light emissions with maxima at 445 and 407 nm when excited at λ ≤ 345 nm, with quantum yields of 1.2% and 0.8%, respectively. Complex **4d** displayed a weak emission with a maximum at 510 nm and quantum yield below the detection threshold (Figure 4, bottom).^26,61^ The quantum yields of these complexes are comparable to other luminescent cationic gold­(III) complexes reported by the groups of Venkatesan (0.3–0.9%)^62^ and Yam (0.1–0.6%)^63^ in solution at room temperature. The different emission maxima in the green or blue region of our complexes showcase the versatility of the ligand scaffold to tune the emission properties of gold­(III) species.

In conclusion, this study presents a novel approach to cyclometalated gold­(III) complexes featuring (P^N^C) ligands. The tandem oxidative addition/cycloauration protocol accommodates both electron-withdrawing and electron-donating groups on C­(sp^2^)-iodides and (P^N^C) ligands, enabling the synthesis of a wide range of derivatives including gold­(III)-aryl, -alkenyl and -alkynyl complexes. Additionally, an alternative two-step method facilitated the preparation of (P^N^C)­gold­(III)-alkynyl complexes, which exhibited emissive properties in the blue and green light regions.

## Supplementary Material



## Data Availability

The data underlying this study are available in the published article and its Supporting Information.

## Notes

Experimental procedures, synthesis and characterization details of the new compounds, NMR spectra, photophysical properties and X-ray diffraction analyses (PDF)
