# Supplementary material for: Synthesis of (P^N^C)Gold(III) Complexes via Tandem Oxidative Addition/C–H Auration
Source: ACS Org Inorg Au. 2025 Aug 23;5(5):322–7. doi: 10.1021/acsorginorgau.5c00057 (PMC12492041; doi:10.1021/acsorginorgau.5c00057)
Supplement: Supplementary file 1 [file gg5c00057_si_001.pdf]

Supporting Information

Synthesis of (P^N^C) Gold(III) Complexes *via* Tandem Oxidative Addition/C-H Auration

Michał Biedrzycki, Jaime Martín, Alexandre Genoux, Elia Boschi, and Cristina Nevado*

Department of Chemistry, University of Zurich, Winterthurerstrasse 190, Zurich, CH 8057, Switzerland

E-mail: cristina.nevado@chem.uzh.ch

Table of Contents

1. General information S2
2. Experimental procedures S3
3. Compounds sythesized S5
4. Control experiments S19
5. Photophysical properties of compounds **4a-d** S21
6. NMR spectra of new compounds S27
7. X-ray diffraction analyses S67
8 References S82

1. General information

Unless otherwise stated, reactions were performed under nitrogen atmosphere using Schlenk techniques and dry solvents. Commercial chemicals were used as received. Compounds 2,8-dibromoquinoline,[1] (*E*)-(2-iodovinyl)benzene,[2] (*Z*)-(2-iodovinyl)benzene,[3] **Alkyne 3** and **Alkyne 4**[4] were prepared according to reported procedures. Flash column chromatography was performed over silica gel (230-400 mesh). NMR (^{1}H, ^{13}C, ^{19}F, ^{31}P) were recorded on either AV2 400, AV2 500 or Avance Neo 500 MHz Bruker spectrometers. Chemical shifts are given in ppm. The spectra were referenced to residual solvent peaks. Multiplicities are abbreviated: singlet (s), doublet (d), triplet (t), quartet (q), septuplet (sept), and multiplet (m). Constant couplings are given in Hz. High-resolution electrospray ionization and electronic impact mass spectrometry were performed on a Finnigan MAT 900 (Thermo Finnigan, San Jose, CA; USA) double focusing magnetic sector mass spectrometer. Ten spectra were acquired. A mass accuracy ≤ 2 ppm was obtained in the peak matching acquisition mode by using a solution containing 2 < lPEG200, 2 < IPPG450, and 1.5 mg NaOAc (all obtained from Sigma-Aldrich, CH-Buchs) dissolved in 100 mL MeOH (HPLC Supra grade, Scharlau, E-Barcelona) as internal standard. UV-Vis measurements were carried out on a Shimadzu UV-1900i UV-Vis spectrophotometer using 10 mm quartz cells. Concentrations of the samples were adjusted to keep absorbance below 0.1. Emission spectra were acquired on Edinburgh Instruments FS5 Spectrofluorometer using 450 W Xenon lamp excitation by exciting at the longest-wavelength absorption maxima. Absolute quantum yields ϕ_{em} were determined at 298 K in dichloromethane using a SC-30 Integrating Sphere on the Edinburgh Instruments Spectrofluorometer FS5. All samples for emission and phosphorescence measurements were prepared in anaerobic cuvettes in a glovebox under Ar atmosphere using solvents degassed by at least three freeze-pump-thaw cycles and pressurized with Ar following each cycle.

2. Experimental procedures

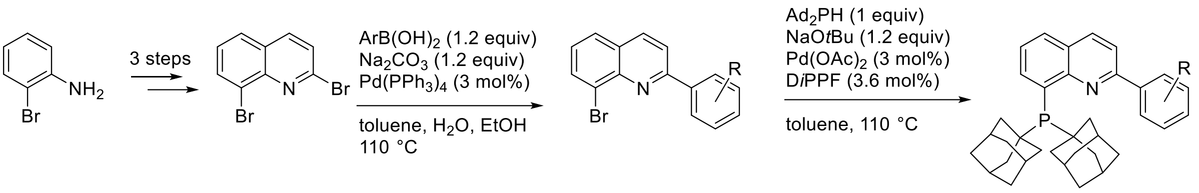

A S1-5 B I-VI

General procedure A. In a Schlenk tube, 2,8-dibromoquinoline (1 equiv), arylboronic acid (1.1 - 1.3 equiv), sodium carbonate (1.2 equiv) and tetrakis(triphenylphosphine)palladium(0) (3 - 5 mol%) were dissolved/suspended in a mixture of toluene, ethanol and water (10:1:1). The mixture was degassed and then stirred at 110 °C in an oil bath until complete consumption of 2,8-dibromoquinoline (usually 20 h, monitored by TLC). After cooling, solvents were evaporated, the residue was dissolved in EtOAc and washed with water and brine. After drying and evaporation, the products were purified using column chromatography.

General procedure B. In a Schlenk tube, the corresponding 2-aryl-8-bromoquinoline (1 equiv), phosphine (1 equiv), sodium *tert*-butoxide (1.2 equiv), palladium acetate (3 mol%) and DiPPF (3.6 mol%) were dissolved in anhydrous toluene. The mixture was heated at 110 °C in an oil bath until complete consumption of phosphine (usually 16 h, monitored by $^{31}P\{^1H\}$ NMR). After cooling, the mixture was filtered through a plug of silica using a mixture of dichloromethane/ethyl acetate (1:1). The filtrate was concentrated, redissolved in minimal amount of dichloromethane, and precipitated using acetone or acetonitrile. The products were pure enough to use in the next step without further purification.

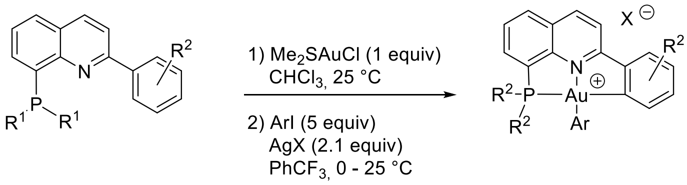

I-VI C 1a-h

General procedure C. In a vial, the corresponding ligand **I-VI** (1 equiv) and chloro(dimethylsulfide)gold(I) (1 equiv) were dissolved in chloroform (0.66 M). The mixture was stirred until complete consumption of the free ligand (usually 1 h, monitored by ^{31}P NMR). After completion, the mixture was filtered through a plug of Celite and concentrated *in vacuo*. The resulting solid was washed with pentane (x3), dried *in vacuo* and used directly in the next step. In a Schlenk tube, AgX (2.1 equiv) and aryl iodide (5 equiv) were suspended in anhydrous trifluorotoluene. The gold(I) complex was added as a solid and the reaction was stirred for 20 h at room temperature. Upon completion, the mixture was filtered through a plug of Celite, the solvent was removed under vacuum and the residue was redissolved in the minimal amount of dichloromethane and precipitated with diethyl ether/pentane (1:1). The formed solid was washed with pentane and dried *in vacuo*. Note: the reaction was also attempted using 2-phenyl-8-(diphenylphosphino)quinoline ligand,[5] however no cyclometalated product was detected.

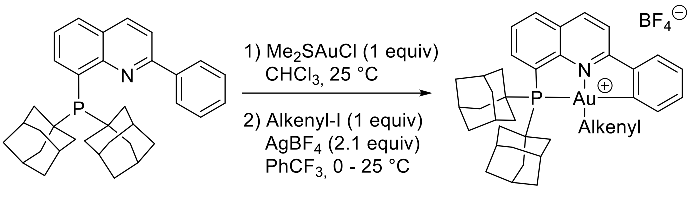

General procedure D. In a vial, the ligand **I** (1 equiv) and chloro(dimethylsulfide)gold(I) (1 equiv) were dissolved in chloroform (0.66 M). The mixture was stirred until the complete consumption of free ligand (usually 1 h, monitored by ^{31}P NMR). After completion, the mixture was filtered through a plug of Celite and concentrated *in vacuo*. The resulting solid was washed with pentane (x3), dried *in vacuo* and used directly in the next step. In a Schlenk tube, $AgBF_4$ (2.1 equiv) and alkenyl iodide (1 equiv) were suspended in trifluorotoluene. The gold(I) complex was added as a solid and the reaction was stirred for 20 h at room temperature. Upon completion, the mixture was filtered through a plug of Celite, the solvent was removed under vacuum and the residue was redissolved in the minimal amount of dichloromethane and precipitated with pentane. The resulting solid was washed with pentane and dried *in vacuo*.

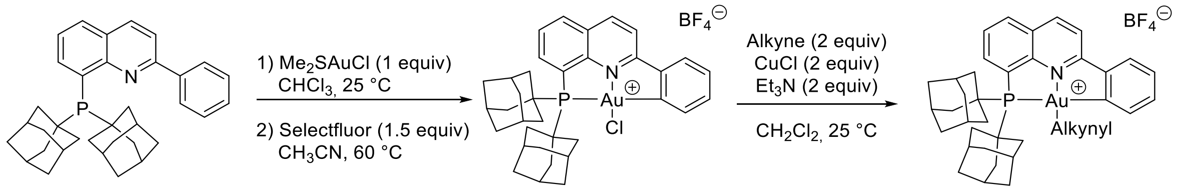

General procedure E. In a vial, the ligand **I** (1 equiv) and chloro(dimethylsulfide)gold(I) (1 equiv) were dissolved in chloroform (0.66 M). The mixture was stirred until complete consumption of free ligand (usually 1 h, monitored by ^{31}P NMR). After completion, the mixture was filtered through a plug of Celite and concentrated *in vacuo*. The resulting solid was washed with pentane (x3), dried *in vacuo* and used directly in the next step. In a Schlenk tube, the gold(I) complex and Selectfluor (1.5 equiv) were added as solids and suspended in acetonitrile. The reaction was stirred for 20 h at 60 °C in an oil bath. Upon completion, the mixture was filtered through a plug of Celite, the solvent was removed under vacuum and the residue was redissolved in dichloromethane, washed with water (x3), dried over anhydrous $MgSO_4$ and filtered. The solvent was concentrated to minimal volume and pentane was added. The resulting solid was washed with pentane and dried *in vacuo*.

General procedure F. In a vial, complex **3** and CuCl (2 equiv) were dissolved in dichloromethane. The alkyne (2 equiv) was added followed by Et_3N (2 equiv). The reaction mixture was stirred until complete consumption of complex **3** (monitored by $^{31}P\{^1H\}$ NMR). Upon completion, the reaction mixture was filtered through a plug of Celite, washed with water (x3), dried over anhydrous $MgSO_4$ and filtered. The solvent was concentrated to minimal volume and pentane was added. The resulting solid was collected and washed with pentane until all remaining alkyne was removed, and then dried *in vacuo*.

3. Compounds synthesized

8-bromo-2-phenylquinoline (S1)

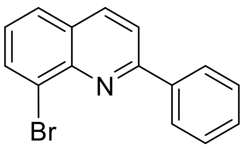

This compound was synthesized by following general procedure A using 2,8-dibromoquinoline (143.5 mg, 0.5 mmol), phenylboronic acid (73.7 mg, 0.60 mmol), sodium carbonate (63.6 mg, 0.6 mmol) and tetrakis(triphenylphosphine)palladium(0) (17.3 mg, 0.015 mmol) in toluene (5 mol), ethanol (0.5 mL) and H_2O (0.5 mL). A brown oil that slowly solidify was isolated (129.1 mg, 0.45 mmol, 91%) using hexane/CH_2Cl_2 (10:1) as an eluent mixture for column chromatography. The analytical data is in agreement with literature.[6]

8-bromo-2-(4-methoxyphenyl)quinoline (S2)

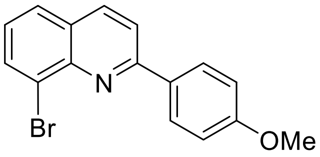

This compound was synthesized by following general procedure A using 2,8-dibromoquinoline (143.5 mg, 0.5 mmol), 4-methoxyphenylboronic acid (91.2mg, 0.60 mmol), sodium carbonate (63.6 mg, 0.6 mmol) and tetrakis(triphenylphosphine)palladium(0) (10.8 mg, 0.010 mmol) in toluene (5 mol), ethanol (0.5 mL) and H_2O (0.5 mL). A yellow oil was isolated (147 mg, 0.47 mmol, 94%) using toluene as an eluent for column chromatography. The analytical data is in agreement with literature.[6]

8-bromo-2-(4-trifluoromethylphenyl)quinoline (S3)

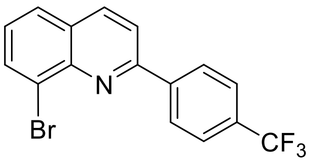

This compound was synthesized by following general procedure A using 2,8-dibromoquinoline (143.5 mg, 0.5 mmol), 4-trifluorophenylboronic acid (114 mg, 0.60 mmol), sodium carbonate (63.6 mg, 0.6 mmol) and tetrakis(triphenylphosphine)palladium(0) (17.3 mg, 0.015 mmol) in toluene (5 mol), ethanol (0.5 mL) and H_2O (0.5 mL). A colorless oil was isolated (175.1 mg, 0.5 mmol, 99%) using hexane/CH_2Cl_2 (10:1) as an eluent mixture for column chromatography. The analytical data is in agreement with literature.[7]

8-bromo-2-(3-methoxyphenyl)quinoline (S4)

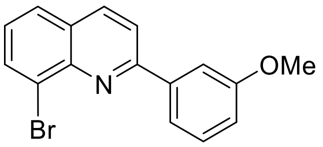

This compound was synthesized by following general procedure A using 2,8-dibromoquinoline (143.5 mg, 0.5 mmol), 3-methoxyphenylboronic acid (91.2 mg, 0.60 mmol), sodium carbonate (63.6 mg, 0.6 mmol) and tetrakis(triphenylphosphine)palladium(0) (17.3 mg, 0.015 mmol) in toluene (5 mol), ethanol (0.5 mL) and H_2O (0.5 mL). A white solid was isolated (122.3 mg, 0.43 mmol, 86%) using toluene as an eluent for column chromatography. ^1H NMR (500.30 MHz, $CDCl_3$): δ 8.19 (d, *J* = 8.6 Hz, 1H), 8.06 (dd, *J* = 7.4, 1.0 Hz, 1H), 8.00–7.97 (m, 1H), 7.95 (d, *J* = 8.6 Hz, 1H), 7.83 (d, *J* = 7.7 Hz, 1H), 7.78 (dd, *J* = 8.1, 1.0 Hz, 1H), 7.44 (t, *J* = 7.9 Hz, 1H), 7.36 (t, *J* = 7.8 Hz, 1H), 7.04 (dd, *J* = 8.2, 2.0 Hz, 1H), 3.95 (s, 3H). $^{13}C\{^1H\}$ NMR (125.81 MHz, $CDCl_3$): δ 160.3, 157.4, 145.1, 140.5, 137.4, 133.4, 130.0, 128.6, 127.4, 126.8, 125.7, 120.1, 119.4, 115.9, 113.0, 55.5. HR-MS (ESI) m/z calcd for $C_{16}H_{13}BrNO$ [M+H$^+$] 314.0175, found 314.0180.

8-bromo-2-(3-trifluoromethylphenyl)quinoline (S5)

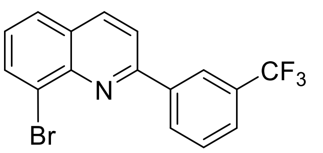

This compound was synthesized by following general procedure A using 2,8-dibromoquinoline (143.5 mg, 0.5 mmol), 3-trifluoromethylphenylboronic acid (114 mg, 0.60 mmol), sodium carbonate (63.6 mg, 0.6 mmol) and tetrakis(triphenylphosphine)palladium(0) (17.3 mg, 0.015 mmol) in toluene (5 mol), ethanol (0.5 mL) and H_2O (0.5 mL). A white solid was isolated (149.5 mg, 0.42 mmol, 85% (95% pure)) using hexane/CH_2Cl_2 (10:1) as an eluent mixture for column chromatography. ^1H NMR (500.30 MHz, $CDCl_3$): δ 8.54 (s, 1H), 8.52 (s, 1H), 8.25 (d, *J* = 8.6 Hz, 1H), 8.09 (dd, *J* = 7.4, 1.0 Hz, 1H), 7.98 (d, *J* = 8.5 Hz, 1H), 7.81 (dd, *J* = 8.3, 0.8 Hz, 1H), 7.74 (d, *J* = 7.7 Hz, 1H), 7.67 (d, *J* = 7.6 Hz, 1H), 7.40 (t, *J* = 7.8 Hz, 1H). $^{13}C\{^1H\}$ NMR (125.81 MHz, $CDCl_3$): δ 156.0, 145.2, 139.8, 137.9, 133.8, 131.4 (q, *J* = 32.4 Hz), 131.1, 129.6, 128.8, 127.5, 127.3, 126.5 (q, *J* = 3.7 Hz), 125.8, 124.5 (q, *J* = 3.9 Hz), 124.3 (q, *J* = 272.4 Hz), 119.1. ^{19}F NMR (470.71 MHz, $CDCl_3$): δ -62.61. HR-MS (ESI) m/z calcd for $C_{16}H_{10}BrF_3N$ [M+H$^+$] 351.9943, found 351.9945.

^{Ad}P^N^C^H (I)

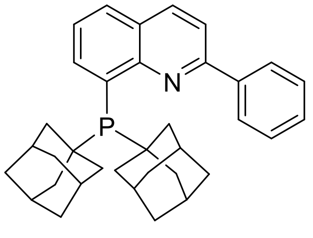

This compound was synthesized by following general procedure B using 8-bromo-2-phenylquinoline (177 mg, 0.62 mmol), (diadamant-1-yl)phosphine (188 mg, 0.62 mmol), sodium *tert*-butoxide (72 mg, 0.74 mmol), Pd(OAc)$_2$ (4.2 mg, 0.019 mmol) and DiPPF (9.4 mg, 0.022 mmol) in toluene (3.7 mL). A

yellow solid (253 mg, 81%) was isolated. For NMR characterization compound was suspended in CD_3CN and aqueous soln. of HBF_4 (1 equiv) was added, after stirring for 30 min mixture was filtered through Celite directly into the NMR tube. ^{1}H NMR (500.30 MHz, CD_3CN): δ 8.57 (dd, *J* = 8.7, 1.1 Hz, 1H), 8.42 (d, *J* = 8.2 Hz, 1H), 8.38–8.31 (m, 3H), 8.25 (d, *J* = 8.7 Hz, 1H), 7.87 (td, *J* = 7.8, 2.6 Hz, 1H), 7.67 (d, *J* = 488.5 Hz, 1H), 7.66–7.57 (m, 3H), 2.33–2.26 (m, 6H), 2.18–2.11 (m, 6H), 2.06–1.99 (m, 6H), 1.81–1.71 (m, 12H). ^{13}C{^{1}H} NMR (125.81 MHz, CD_3CN): δ 159.4, 149.2, 139.7, 138.9, 138.5 (d, *J* = 7.2 Hz), 136.0 (d, *J* = 2.7 Hz), 131.7 (d, *J* = 73.8 Hz), 130.2, 129.1, 128.9 (d, *J* = 5.4 Hz), 127.0 (d, *J* = 12.6 Hz), 121.6, 113.5, 40.0 (d, *J* = 33.5 Hz), 39.3 (d, *J* = 1.9 Hz), 36.0 (d, *J* = 1.5 Hz), 28.8 (d, *J* = 10.1 Hz). ^{31}P{^{1}H} NMR (202.52 MHz, CD_3CN): δ 10.85. HR-MS (ESI) m/z calcd for $C_{35}H_{41}NP$ [M+H$^+$] 506.2971, found 506.2973.

^{tBu}P^N^C^H (II)

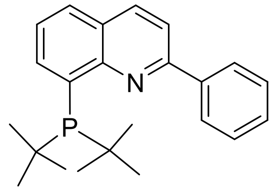

This compound was synthesized by following general procedure B using 8-bromo-2-phenylquinoline (675 mg, 2.37 mmol), di-*tert*-butylphosphine (347 mg, 0.44 mL, 2.37 mmol), sodium *tert*-butoxide (270 mg, 2.84 mmol), $Pd(OAc)_2$ (16 mg, 0.07 mmol) and DiPPF (40 mg, 0.095 mmol) in toluene (16 mL). A dark yellow solid (610 mg, 74%) was isolated. ^{1}H NMR (400.13 MHz, CD_2Cl_2, 298 K): *δ* 8.29 (d, *J* = 7.5 Hz, 2H), 8.23–8.19 (m, 2H), 7.92 (d, *J* = 8.6 Hz, 1H), 7.86 (d, *J* = 8.0 Hz, 1H), 7.56–7.53 (m, 3H), 7.47 (t, *J* = 7.1 Hz, 1H), 1.28 (d, *J* = 11.4 Hz, 18H). ^{13}C{^{1}H} NMR (100.65 MHz, CD_2Cl_2, 298 K): *δ* 155.9 (d, *J* = 2.0 Hz), 152.4 (d, *J* = 21.0 Hz), 140.1, 139.0 (d, *J* = 29.7 Hz), 137.5 (d, *J* = 2.0 Hz), 137.3 (d, *J* = 4.8 Hz), 129.7, 129.1, 128.9, 127.9, 127.7 (d, *J* = 2.9 Hz), 125.4, 118.3 (d, *J* = 1.8 Hz), 32.7 (d, *J* = 26.4 Hz), 31.1 (d, *J* = 15.6 Hz). ^{31}P{^{1}H} NMR (161.99 MHz, CD_2Cl_2, 298 K): *δ* 14.1. HR-MS (ESI) m/z calcd for $C_{23}H_{29}NP$ [M+H]$^+$ 350.2032; found, 350.2032. Analytical data is in agreement with literature.[8]

^{Ad}P^N^*C*^{pOMe} (III)

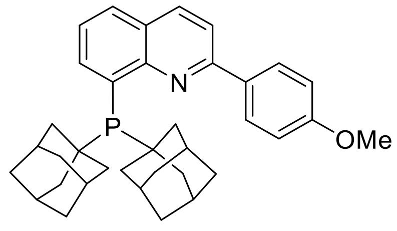

This compound was synthesized by following general procedure B using 8-bromo-2-(4-methoxyphenyl)quinoline (148 mg, 0.47 mmol), (diadamant-1-yl)phosphine (142 mg, 0.47 mmol), sodium *tert*-butoxide (54 mg, 0.56 mmol), $Pd(OAc)_2$ (3.2 mg, 0.014 mmol) and DiPPF (7.1 mg, 0.017 mmol) in toluene (2.8 mL). A yellowish solid (112 mg, 44%) was isolated. For NMR characterization compound was suspended in CD_3CN and aqueous soln. of HBF_4 (1 equiv) was added, after stirring for 30 min mixture was filtered through Celite directly into NMR tube. ^{1}H NMR (500.30 MHz, $CDCl_3$): δ 8.49 (dd, *J* = 8.7, 1.1 Hz, 1H), 8.37 (d, *J* = 8.1 Hz, 1H), 8.34–8.28 (m, 3H), 8.18 (d, *J* = 8.7 Hz, 1H), 7.82 (td, *J* = 7.8, 2.6 Hz, 1H), 7.65 (d, *J* = 488.2 Hz, 1H), 7.16–7.12 (m, 2H), 3.90 (s, 3H), 2.32–2.24 (m, 6H), 2.17–2.10 (m, 6H), 2.06–1.99 (m, 6H), 1.82–1.70 (m, 12H). ^{13}C{^{1}H} NMR (125.81 MHz, CD_3CN): δ 163.0, 159.0, 149.2, 139.4, 138.3 (d, *J* = 7.2 Hz), 136.0 (d, *J* = 2.6 Hz), 131.3, 130.7, 128.6 (d, *J* = 5.5 Hz), 126.6 (d, *J* =

12.6 Hz), 121.1, 115.5, 113.1 (d, *J* = 73.8 Hz), 56.3, 40.0 (d, *J* = 33.7 Hz), 39.3 (d, *J* = 1.9 Hz), 36.0 (d, *J* = 1.5 Hz), 28.8 (d, *J* = 10.1 Hz). $^{31}P\{^1H\}$ NMR (202.52 MHz, CD_3CN): δ 10.83. HR-MS (ESI) m/z calcd for $C_{36}H_{43}NOP$ [M+H$^+$] 536.3077, found 536.3075.

^{Ad}P^N^C^{pCF3} (IV)

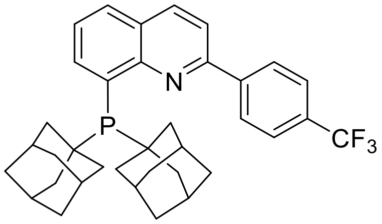

This compound was synthesized by following general procedure B using 8-bromo-2-(4-trifluoromethylphenyl)quinoline (163 mg, 0.46 mmol), (diadamant-1-yl)phosphine (139 mg, 0.46 mmol), sodium *tert*-butoxide (53 mg, 0.55 mmol), $Pd(OAc)_2$ (2.5 mg, 0.011 mmol) and DiPPF (6.9 mg, 0.016 mmol) in toluene (2.8 mL). A yellowish solid (153 mg, 58%) was isolated. For NMR characterization compound was suspended in CD_3CN and aqueous soln. of HBF_4 (1 equiv) was added, after stirring for 30 min mixture was filtered through Celite directly into NMR tube. ^1H NMR (500.30 MHz, CD_3CN): δ 8.63 (dd, *J* = 8.7, 1.1 Hz, 1H), 8.49 (d, *J* = 8.2 Hz, 2H), 8.45 (d, *J* = 8.2 Hz, 1H), 8.38 (ddd, *J* = 11.2, 7.3, 0.9 Hz, 1H), 8.29 (d, *J* = 8.7 Hz, 1H), 7.95–7.88 (m, 3H), 7.68 (d, *J* = 488.6 Hz, 1H), 2.33–2.24 (m, 6H), 2.18–2.10 (m, 6H), 2.06–1.99 (m, 6H), 1.81–1.70 (m, 12H). $^{13}C\{^1H\}$ NMR (125.81 MHz, CD_3CN): δ 157.9, 149.1, 142.6, 140.2, 138.9 (d, *J* = 7.1 Hz), 136.1 (d, *J* = 2.7 Hz), 132.3 (q, *J* = 32.3 Hz), 129.8, 129.2 (d, *J* = 5.4 Hz), 127.6 (d, *J* = 12.6 Hz), 126.9 (q, *J* = 3.8 Hz), 125.4 (q, *J* = 271.5 Hz), 121.8, 113.8 (d, *J* = 73.3 Hz), 40.0 (d, *J* = 33.4 Hz), 39.2 (d, *J* = 1.9 Hz), 36.0 (d, *J* = 1.6 Hz), 28.8 (d, *J* = 10.1 Hz). ^{19}F NMR (470.71 MHz, CD_3CN): δ -63.22, -151.72, -151.77 (BF_4). $^{31}P\{^1H\}$ NMR (202.52 MHz, CD_3CN): δ 10.83. HR-MS (ESI) m/z calcd for $C_{36}H_{40}F_3NP$ [M+H$^+$] 574.2845, found 574.2844.

^{Ad}P^N^C^{mOMe} (V)

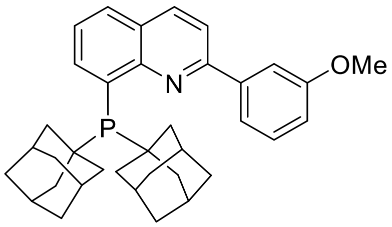

This compound was synthesized by following general procedure B using 8-bromo-2-(3-methoxyphenyl)quinoline (62.8 mg, 0.2 mmol), (diadamant-1-yl)phosphine (60.5 mg, 0.2 mmol), sodium *tert*-butoxide (23.1 mg, 0.24 mmol), $Pd(OAc)_2$ (1.4 mg, 0.006 mmol) and DiPPF (3.0 mg, 0.007 mmol) in toluene (1.5 mL). A yellowish solid (62.4 mg, 57%) was isolated. For NMR characterization compound was suspended in CD_3CN and aqueous soln. of HBF_4 (1 equiv) was added, after stirring for 30 min mixture was filtered through Celite directly into NMR tube. ^1H NMR (500.30 MHz, CD_3CN): δ 8.56 (dd, *J* = 8.7, 1.2 Hz), 8.42 (d, *J* = 8.2 Hz, 1H), 8.35 (ddd, *J* = 11.2, 7.3, 1.1 Hz, 1H), 8.23 (d, *J* = 8.7 Hz, 1H), 7.91–7.85 (m, 2H), 7.78 (t, *J* = 2.1 Hz, 1H), 7.64 (d, *J* = 487.3 Hz, 1H), 7.54 (t, *J* = 8.0 Hz, 1H), 7.15 (ddd, *J* = 8.2, 2.7, 0.7 Hz, 1H), 3.95 (s, 3H), 2.33–2.25 (m, 6H), 2.20–2.10 (m, 6H), 2.06–2.00 (m, 6H), 1.81–1.72 (m, 12H). $^{13}C\{^1H\}$ NMR (125.81 MHz, CD_3CN): δ 161.3, 159.3, 149.1, 140.6, 139.7, 138.5 (d, *J* = 7.2 Hz), 136.1 (d, *J* = 2.6 Hz), 131.3, 129.0 (d, *J* = 5.5 Hz), 127.1 (d, *J* = 12.6 Hz), 121.9, 121.5, 117.3,

114.1, 113.5 (d, J = 73.7 Hz), 56.2, 40.0 (d, J = 33.5 Hz), 39.3 (d, J = 1.9 Hz), 36.0 (d, J = 1.5 Hz), 28.7 (d, J = 10.1 Hz). $^{31}P\{^1H\}$ NMR (202.52 MHz, CD_3CN): δ 11.26. HR-MS (ESI) m/z calcd for $C_{36}H_{43}NOP$ [M+H$^+$] 536.3077, found 536.3080.

^{Ad}P^N^C^{mCF3} (VI)

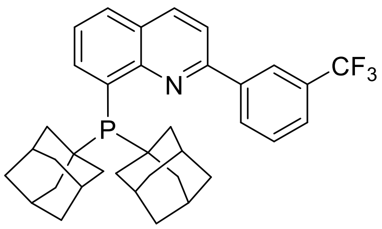

This compound was synthesized by following general procedure B using 8-bromo-2-(3-trifluoromethylphenyl)quinoline (70.4 mg, 0.2 mmol), (diadamant-1-yl)phosphine (60.5 mg, 0.2 mmol), sodium *tert*-butoxide (23.1 mg, 0.24 mmol), $Pd(OAc)_2$ (1.4 mg, 0.006 mmol) and DiPPF (3.0 mg, 0.007 mmol) in toluene (1.5 mL). An off-white solid (70 mg, 61%) was isolated. For NMR characterization compound was suspended in CD_3CN and aqueous soln. of HBF_4 (1 equiv) was added, after stirring for 30 min mixture was filtered through Celite directly into NMR tube. ^1H NMR (500.30 MHz, CD_3CN): δ 8.65–8.59 (m, 2H), 8.51 (s, 1H), 8.45 (dt, J = 8.2, 1.4 Hz, 1H), 8.39 (ddd, J = 11.3, 7.3, 1.1 Hz, 1H), 8.29 (d, J = 8.7 Hz, 1H), 7.94–7.88 (m, 2H), 7.84 (t, J = 7.8 Hz, 1H), 7.62 (d, J = 487.9 Hz, 1H), 2.33–2.26 (m, 6H), 2.18–2.11 (m, 6H), 2.06–2.00 (m, 6H), 1.81–1.72 (m, 12H). $^{13}C\{^1H\}$ NMR (125.81 MHz, CD_3CN): δ 157.9, 149.1, 140.1, 140.0, 138.8 (d, J = 7.1 Hz), 136.1 (d, J = 2.7 Hz), 132.9, 131.7 (q, J = 32.3 Hz), 131.2, 129.2 (d, J = 5.4 Hz), 128.0 (q, J = 3.6 Hz), 127.5 (d, J = 12.6 Hz), 125.6 (q, J = 3.9 Hz), 125.3 (q, J = 271.7 Hz), 121.7, 113.8 (d, J = 73.2 Hz), 40.1 (d, J = 33.3 Hz), 39.3 (d, J = 1.9 Hz), 36.0 (d, J = 1.5 Hz), 28.8 (d, J = 10.2 Hz). ^{19}F NMR (470.71 MHz, CD_3CN): δ -63.35, -152.58, -152.63 (BF_4). $^{31}P\{^1H\}$ (202.52 MHz, CD_3CN): δ 11.22. HR-MS (ESI) m/z calcd for $C_{36}H_{40}F_3NP$ [M+H$^+$] 574.2845, found 574.2843.

P^N^C^{H}Au(4-F-C_6H_4) (1a)

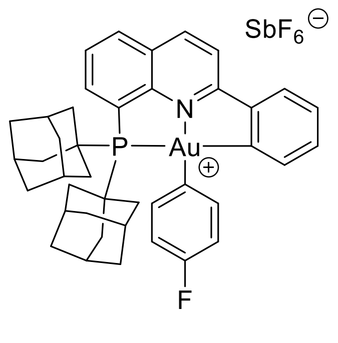

This compound was synthesized by following general procedure C using **I** (16.7 mg, 0.033 mmol) and dimethylsulfidegold(I) chloride (9.7 mg, 0.033 mmol) in $CHCl_3$ (0.5 mL). Then 4-fluoroiodobenzene (19 µl, 0.165 mmol) and silver hexafluoroantimonate (23.8 mg, 0.069 mmol) in trifluorotoluene (2 mL). A white solid (23.3 mg, 68%) was isolated. ^1H NMR (500.30 MHz, CD_2Cl_2): δ 8.86 (dd, J = 8.8, 1.4 Hz, 1H), 8.39 (t, J = 7.3 Hz, 1H), 8.35 (d, J = 8.1 Hz, 1H), 8.31 (d, J = 8.8 Hz, 1H), 8.06–8.01 (m, 2H), 7.68 (dd, J = 8.2, 5.7 Hz, 2H), 7.49 (t, J = 7.5 Hz, 1H), 7.39 (tdd, J = 7.4, 2.9, 1.4 Hz, 1H), 7.21 (t, J = 8.9 Hz, 2H), 6.65 (td, J = 7.6, 1.2 Hz, 1H), 2.31–2.23 (m, 6H), 2.09–1.99 (m, 12H), 1.81–1.67 (m, 12H). $^{13}C\{^1H\}$ NMR (125.81 MHz, CD_2Cl_2): δ 166.4 (d, J = 5.9 Hz), 165.0 (d, J = 114.5 Hz), 161.8 (d, J = 245.3 Hz), 151.4 (d, J = 10.5

Hz), 147.8, 144.1, 142.7 (d, J = 2.0 Hz), 137.4 (dd, J = 7.4, 3.3 Hz), 134.25, 134.18, 134.1 (d, J = 54.6 Hz), 134.0 (d, J = 2.2 Hz), 130.5 (d, J = 6.4 Hz), 129.7 (d, J = 6.1 Hz), 129.4, 128.4 (d, J = 5.3 Hz), 126.7 (d, J = 34.7 Hz), 119.7, 117.5 (d, J = 20.2 Hz), 44.9 (d, J = 12.5 Hz), 41.1, 36.1 (d, J = 1.8 Hz), 28.7 (d, J = 9.6 Hz). ^{19}F (470.71 MHz, CD_2Cl_2): δ -117.13– -117.24 (m). ^{31}P{^{1}H} NMR (202 MHz, CD_2Cl_2): δ 63.90. HR-MS (ESI) m/z calcd for $C_{41}H_{43}AuFNP$ [M$^+$] 796.2777, found 796.2774.

^{tBu}P^N^C^{H}Au(4-F-C_6H_4) (1b)

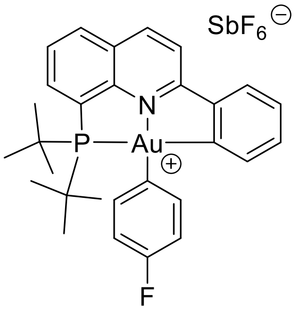

This compound was synthesized by following general procedure C using **II** (11.5 mg, 0.033 mmol) and dimethylsulfidegold(I) chloride (9.7 mg, 0.033 mmol) in $CHCl_3$ (0.5 mL). Then 4-fluoroiodobenzene (19 µl, 0.165 mmol) and silver hexafluoroantimonate (23.8 mg, 0.069 mmol) in trifluorotoluene (2 mL). An off-white solid (16.1 mg, 56%) was isolated. ^{1}H NMR (500.30 MHz, CD_2Cl_2): δ 8.87 (dd, J = 8.8, 1.4 Hz, 1H), 8.42 (td, J = 7.5, 1.2 Hz, 1H), 8.36 (dt, J = 8.1, 1.4 Hz, 1H), 8.34 (d, J = 8.8 Hz, 1H), 8.06–8.00 (m, 2H), 7.64–7.59 (m, 2H), 7.50 (tt, J = 7.7, 1.0 Hz, 1H), 7.39 (tdd, J = 7.5, 3.0, 1.4 Hz, 1H), 7.22–7.16 (m, 2H), 6.66 (td, J = 7.7, 1.2 Hz, 1H), 1.46 (d, J = 16.5 Hz, 18H). ^{13}C{^{1}H} NMR (125.81 MHz, CD_2Cl_2): δ 166.5 (d, J = 6.1 Hz), 164.4 (d, J = 116.9 Hz), 161.9 (d, J = 244.7 Hz), 150.9 (d, J = 11.0 Hz), 147.9, 144.2, 142.1 (d, J = 1.7 Hz), 137.4 (dd, J = 7.7, 3.3 Hz), 134.3, 134.2, 134.1, 133.9 (d, J = 6.5 Hz), 130.3 (d, J = 6.5 Hz), 129.9 (d, J = 6.2 Hz), 129.4, 128.9 (d, J = 35.4 Hz), 128.5 (d, J = 5.4 Hz), 119.9, 117.6 (d, J = 20.4 Hz), 39.8 (d, J = 15.3 Hz), 29.9 (d, J = 3.9 Hz). ^{19}F (470.71 MHz, CD_2Cl_2): δ -116.89– -117.09 (m). ^{31}P{^{1}H} NMR (202 MHz, CD_2Cl_2): δ 72.04. HR-MS (ESI) m/z calcd for $C_{29}H_{31}AuFNP$ [M$^+$] 640.1838, found 640.1838.

^{Ad}P^N^C^{H}Au(4-MeO-C_6H_4) (1c)

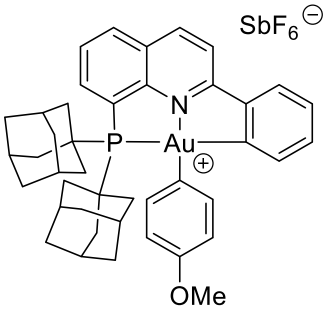

This compound was synthesized by following general procedure C using **I** (16.2 mg, 0.032 mmol) and dimethylsulfidegold(I) chloride (9.4 mg, 0.032 mmol) in $CHCl_3$ (0.5 mL). Then 4-iodoanisole (38.6 mg, 0.165 mmol) and silver hexafluoroantimonate (23.8 mg, 0.069 mmol) in trifluorotoluene (2 mL). A yellow solid (19 mg, 57%) was isolated. ^{1}H NMR (500.30 MHz, CD_2Cl_2): δ 8.86 (dd, J = 8.8, 1.4 Hz, 1H), 8.39 (t, J = 7.2 Hz, 1H), 8.35 (d, J = 8.2 Hz, 1H), 8.32 (d, J = 8.8 Hz, 1H), 8.04–8.01 (m, 2H), 7.58 (d, J =

7.8 Hz, 2H), 7.47 (t, J = 7.5 Hz, 1H), 7.39 (tdd, J = 7.4, 2.9. 1.4 Hz, 1H), 7.02 (d, J = 8.3 Hz, 2H), 6.71 (td, J = 7.6, 1.2 Hz, 1H), 3.88 (s, 3H), 2.29–2.24 (m, 6H), 2.09–2.03 (m, 12H), 1.81–1.68 (m, 12H). $^{13}C\{^1H\}$ NMR (125.81 MHz, CD_2Cl_2): δ 166.3 (d, J = 5.9 Hz), 165.4 (d, J = 116.3 Hz), 158.5, 151.4 (d, J = 10.5 Hz), 147.8, 143.8, 142.6 (d, J = 1.9 Hz), 134.13, 134.09, 133.8 (d, J = 2.1 Hz), 133.54, 133.48, 130.4 (d, J = 6.2 Hz), 129.6 (d, J = 6.2 Hz), 129.2, 128.3 (d, J = 5.3 Hz), 127.1 (d, J = 34.2 Hz), 119.7, 116.2, 55.6, 44.8 (d, J = 12.4 Hz), 41.1, 36.2 (d, J = 1.8 Hz), 28.8 (d, J = 9.6 Hz). $^{31}P\{^1H\}$ NMR (202 MHz, CD_2Cl_2): δ 63.07. HR-MS (ESI) m/z calcd for $C_{42}H_{46}AuNOP$ [M^+] 808.2977, found 808.2975.

^{Ad}P^N^C^{H}Au(4-CF_3-C_6H_4) (1d)

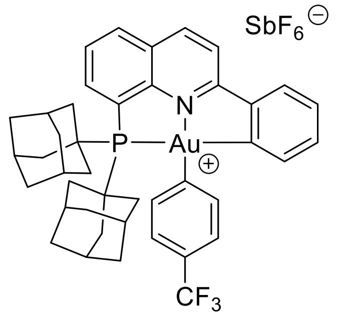

This compound was synthesized by following general procedure C using **I** (16.2 mg, 0.032 mmol) and dimethylsulfidegold(I) chloride (9.4 mg, 0.032 mmol) in $CHCl_3$ (0.5 mL). Then 4-iodobenzotrifluoride (24 µl, 0.165 mmol) and silver hexafluoroantimonate (23.8 mg, 0.069 mmol) in trifluorotoluene (2 mL). A white solid (21.7 mg, 61%, 88% pure) was isolated. ^1H NMR (500.30 MHz, CD_2Cl_2): δ 8.89 (dd, J = 8.8, 1.2 Hz, 1H), 8.41 (t, J = 7.4 Hz, 1H), 8.38 (d, J = 8.2 Hz, 1H), 8.34 (d, J = 8.8 Hz, 1H), 8.08–8.03 (m, 2H), 7.89 (d, J = 8.0 Hz, 2H), 7.69 (d, J = 8.0 Hz, 2H), 7.50 (t, J = 7.5 Hz, 1H), 7.38 (tdd, J = 7.7, 2.8, 1.3 Hz, 1H), 6.56 (t, J = 7.7 Hz, 1H), 2.32–2.23 (m, 6H), 2.07–1.98 (m, 12H), 1.82 – 1.68 (m, 12H). $^{13}C\{^1H\}$ NMR (125.81 MHz, CD_2Cl_2): δ 166.5 (d, J = 6.0 Hz), 164.7 (d, J = 111.9 Hz), 151.4 (d, J = 10.5 Hz), 148.1 (d, J = 7.3 Hz), 147.8, 144.3, 142.8, 134.3 (d, J = 7.9 Hz), 134.1 (d, J = 2.2 Hz), 134.0, 133.7, 130.5 (d, J = 6.4 Hz), 129.8 (d, J = 6.4 Hz), 129.5, 128.94 (d, J = 4.6 Hz), 128.88 (d, J = 32.2 Hz), 128.6 (d, J = 5.2 Hz), 127.0 (d, J = 4.0 Hz), 126.4 (d, J = 35.4 Hz), 124.6 (q, J = 271.6 Hz), 119.8, 45.0 (d, J = 12.3 Hz), 41.1, 36.1, 28.7 (d, J = 9.6 Hz). ^{19}F NMR (470.71 MHz, CD_2Cl_2): δ -62.96. $^{31}P\{^1H\}$ NMR (202.52 MHz, CD_2Cl_2): δ 65.31. HR-MS (ESI) m/z calcd for $C_{42}H_{43}AuF_3NP$ [M^+] 846.2745, found 846.2740.

^{Ad}P^N^C^{pOMe}Au(4-F-C_6H_4) (1e)

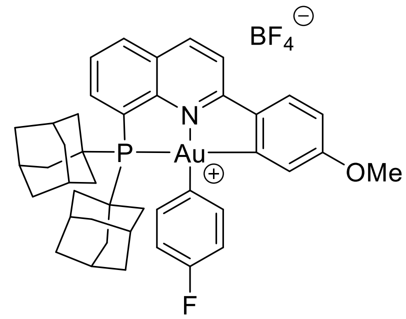

This compound was synthesized by following general procedure C using **III** (17.7 mg, 0.033 mmol) and dimethylsulfidegold(I) chloride (9.7 mg, 0.033 mmol) in $CHCl_3$ (0.5 mL). Then 4-fluoroiodobenzene (19 µl, 0.165 mmol) and silver tetrafluoroborate (13.5 mg, 0.069 mmol) in trifluorotoluene (2 mL). A yellow solid (25.9 mg, 87%) was isolated. ^{1}H NMR (500.30 MHz, CD_2Cl_2): δ 8.83 (dd, *J* = 8.9, 1.4 Hz, 1H), 8.37–8.32 (m, 2H), 8.23 (d, *J* = 8.9, Hz, 1H), 8.04 (dd, *J* = 8.7, 4.3 Hz, 1H), 7.98 (td, *J* = 7.7, 1.7 Hz, 1H), 7.68 (dd, *J* = 8.2, 5.8 Hz, 2H), 7.20 (t, *J* = 8.9 Hz, 2H), 6.96 (dd, *J* = 8.6, 2.6 Hz, 1H), 6.14 (dd, *J* = 8.8, 2.6 Hz, 1H), 3.70 (s, 3H), 2.30–2.23 (m, 6H), 2.06–1.99 (m, 12H), 1.80–1.68 (m, 12H). $^{13}C\{^{1}H\}$ NMR (125.81 MHz, CD_2Cl_2): δ 166.8 (d, *J* = 114.7), 166.2 (d, *J* = 5.5 Hz), 164.1 (d, *J* = 10.1 Hz), 161.8 (d, *J* = 244.9 Hz), 151.5 (d, *J* = 10.6 Hz), 143.7, 142.3 (d, *J* = 2.5 Hz), 139.8, 137.8 (dd, *J* = 7.4, 3.3 Hz), 134.4 (d, *J* = 6.5 Hz), 134.1 (d, *J* = 2.2 Hz), 130.6 (d, *J* = 6.5 Hz), 129.9 (d, *J* = 6.5 Hz), 129.0 (d, *J* = 6.4 Hz), 125.8 (d, *J* = 35.6 Hz), 120.3, 119.7, 117.3 (d, *J* = 20.3 Hz), 113.5, 56.0, 44.8 (d, *J* = 12.7 Hz), 41.1, 36.2 (d, *J* = 1.8 Hz), 28.7 (d, *J* = 9.6 Hz). ^{19}F NMR (470.71 MHz, CD_2Cl_2): δ -116.83– -116.93 (m), -152.96, -153.02. $^{31}P\{^{1}H\}$ NMR (202.52 MHz, CD_2Cl_2): δ 64.58. HR-MS (ESI) m/z calcd for $C_{42}H_{45}AuFNOP$ [M^+] 826.2883, found 826.2874.

^{Ad}P^N^C^{pCF3}Au(4-F-C_6H_4) (1f)

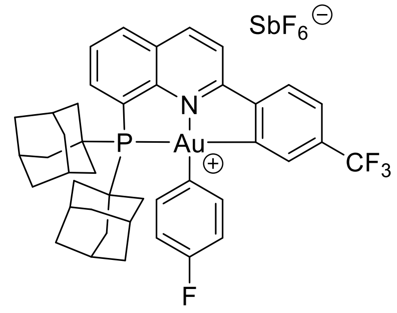

This compound was synthesized by following general procedure C using **IV** (18.9 mg, 0.033 mmol) and dimethylsulfidegold(I) chloride (9.7 mg, 0.033 mmol) in $CHCl_3$ (0.5 mL). Then 4-fluoroiodobenzene (19 µl, 0.165 mmol) and silver hexafluoroantimonate (23.8 mg, 0.069 mmol) in trifluorotoluene (2 mL). A white solid (28.9 mg, 80%) was isolated. ^{1}H NMR (500.30 MHz, CD_2Cl_2): δ 8.97 (dd, *J* = 8.8, 1.3 Hz, 1H), 8.46–8.39 (m, 3H), 8.18 (dd, *J* = 8.2, 3.3 Hz, 1H), 8.09 (dt, *J* = 7.7, 1.7 Hz, 1H), 7.75 (d, *J* = 8.2 Hz, 1H), 7.68 (dd, *J* =8.1, 5.6 Hz, 2H), 7.24 (t, *J* = 8.9 Hz, 2H), 6.88 (dd, *J* = 7.7, 1.8 Hz, 1H), 2.31–2.24 (m, 6H), 2.08–2.00 (m, 12H), 1.81–1.68 (m, 12H). $^{13}C\{^{1}H\}$ NMR (125.81 MHz, CD_3CN): δ 166.3 (d, *J* = 117.8 Hz), 165.2 (d, *J* = 5.4 Hz), 162.3 (d, *J* = 243.3 Hz), 152.7, 151.9 (d, *J* = 10.3 Hz), 145.2, 144.2 (d, *J* = 1.8 Hz), 137.6 (dd, *J* = 6.9, 3.1 Hz), 135.3 (d, *J* = 6.5 Hz), 134.4 (d, *J* = 2.2 Hz), 133.7 (dq, *J* = 32.4, 8.3 Hz), 131.7 (d, *J* = 6.4 Hz), 130.8 (d, *J* = 6.5 Hz), 129.8 (dd, *J* = 2.1 Hz), 129.3 (d, *J* = 5.3 Hz), 127.5 (d, *J* = 36.2 Hz), 126.4 (q, *J* = 3.8 Hz), 124.8 (dq, *J* = 272.5, 1.5 Hz), 120.9, 117.9 (d, *J* = 20.5 Hz), 45.3 (d, *J* = 13.4 Hz),

41.1, 36.3, 29.3 (d, J = 9.7 Hz). ^{19}F NMR (470.71 MHz, CD_2Cl_2): δ -64.08, -116.30— -116.38 (m). $^{31}P\{^1H\}$ NMR (202.52 MHz, CD_2Cl_2): δ 64.55. HR-MS (ESI) m/z calcd for $C_{42}H_{42}AuF_4NP$ [M^+] 864.2651, found 864.2647.

^{Ad}P^N^CAu^{mOMe}(4-F-C_6H_4) (1g)

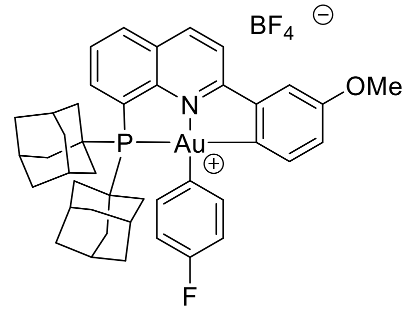

This compound was synthesized by following general procedure C using **V** (17.7 mg, 0.033 mmol) and dimethylsulfidegold(I) chloride (9.7 mg, 0.033 mmol) in $CHCl_3$ (0.5 mL). Then 4-fluoroiodobenzene (19 µl, 0.165 mmol) and silver tetrafluoroborate (13.5 mg, 0.069 mmol) in trifluorotoluene (2 mL). A yellow solid (23.1 mg, 77%) was isolated. ^{1}H NMR (500.30 MHz, CD_2Cl_2): δ 8.96 (d, J = 8.8 Hz, 1H), 8.43–8.37 (m, 3H), 8.04 (t, J = 7.7 Hz, 1H), 7.68 (dd, J = 8.2, 5.7 Hz, 2H), 7.59 (t, J = 2.9 Hz, 1H), 7.19 (t, J = 8.8 Hz, 2H, 6.92–6.87 (m, 1H), 6.53 (t, J = 7.9 Hz, 1H), 3.89 (s, 3H), 2.31–2.22 (m, 6H), 2.06–1.98 (m, 12H), 1.80–1.67 (m, 12H). $^{13}C\{^1H\}$ NMR (125.81 MHz, CD_2Cl_2): δ 166.1 (d, J = 5.8 Hz), 161.7 (d, J = 244.5 Hz), 160.4, 156.6 (d, J = 116.7 Hz), 151.4 (d, J = 10.7 Hz), 148.9, 144.4, 142.7 (d, J = 1.9 Hz), 137.2 (dd, J = 7..3, 3.3 Hz), 134.8 (d, J = 1.8 Hz), 134.3 (d, J = 6.3 Hz), 134.1 (d, J = 1.8 Hz), 130.6 (d, J = 6.4 Hz), 129.7 (d, J = 6.2 Hz), 126.6 (d, J = 34.7 Hz), 120.0, 119.0 (d, J = 8.3 Hz), 117.4 (d, J = 20.2 Hz), 114.2 (d, J = 5.8 Hz), 56.1, 44.8 (d, J = 12.5 Hz), 41.1, 36.1 (d, J = 1.9 Hz), 28.7 (d, J = 9.6 Hz). ^{19}F NMR (470.71 MHz, CD_2Cl_2): δ -117.11– -117.20 (m), -152.82, -152.87. $^{31}P\{^1H\}$ NMR (202.52 MHz, CD_2Cl_2): δ 64.03. HR-MS (ESI) m/z calcd for $C_{42}H_{45}Au_FNOP$ [M^+] 826.2883, found 826.2881.

^{Ad}P^N^C^{mCF3}Au(4-F-C_6H_4) (1h)

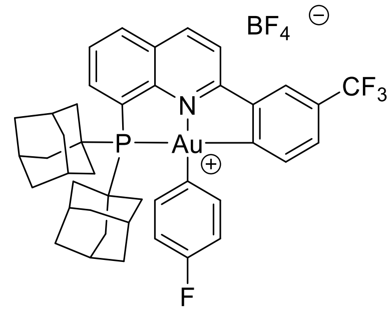

This compound was synthesized by following general procedure C using **VI** (18.9 mg, 0.033 mmol) and dimethylsulfidegold(I) chloride (9.7 mg, 0.033 mmol) in $CHCl_3$ (0.5 mL). Then 4-fluoroiodobenzene (19 µl, 0.165 mmol) and silver tetrafluoroborate (13.5 mg, 0.069 mmol) in trifluorotoluene (2 mL). A off-white solid (23.5 mg, 75%) was isolated. ^{1}H NMR (500.30 MHz, CD_2Cl_2): δ 9.08 (dd, J = 8.9, 1.1 Hz, 1H), 8.51–8.43 (m, 3H), 8.27 (t, J = 2.5 Hz, 1H), 8.12 (td, J = 8.0, 1.5 Hz, 1H), 7.68 (dd, J = 8.0, 5.8 Hz, 2H), 7.62 (dt, J = 8.1, 2.3 Hz, 1H), 7.23 (t, J = 8.9 Hz, 2H), 6.80 (t, J = 7.6 Hz, 1H), 2.31–2.24 (m, 6H), 2.07–2.00 (m, 12H), 1.81–1.67 (m, 12H). $^{13}C\{^1H\}$ NMR (125.81 MHz, CD_2Cl_2): δ 168.4 (d, J = 115.3 Hz), 164.9

(d, J = 5.6 Hz), 161.9 (d, J = 245.3 Hz), 151.3 (d, J = 10.2 Hz), 148.7, 145.2, 143.3, 136.5 (dd, J = 6.8, 3.3 Hz), 134.45 (d, J = 2.0 Hz), 134.39 (d, J = 1.1 Hz), 134.1 (d, J = 6.5 Hz), 131.3 (q, J = 33.3 Hz), 131.0 (d, J = 6.4 Hz), 130.3 (d, J = 6.3 Hz), 130.1 (dd, J = 7.8, 3.7 Hz), 126.6 (d, J = 35.4 Hz), 124.6 (dq, J = 7.8, 3.8 Hz), 124.0 (q, J = 271.7 Hz), 119.8, 117.7 (d, J = 20.8 Hz), 45.1 (d, J = 12.6 Hz), 41.1, 36.1 (d, J = 1.3 Hz), 28.7 (d, J = 9.6 Hz). ^{19}F NMR (470.71 MHz, CD_2Cl_2): δ -63.24, -116.40– -116.50 (m), -152.81, -152.86. ^{31}P{^{1}H} NMR (202.52 MHz, CD_2Cl_2): δ 64.64. HR-MS (ESI) m/z calcd for $C_{42}H_{42}AuF_4NP$ [M$^+$] 864.2651, found 864.2636.

^{Ad}P^N^C^{H}Au(vinyl) (2a)

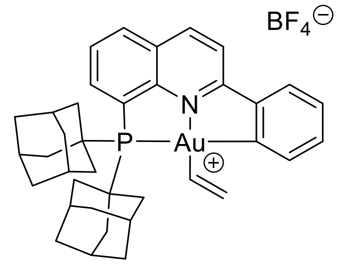

This compound was synthesized by following general procedure D using **I** (8.6 mg, 0.017 mmol) and dimethylsulfidegold(I) chloride (5.0 mg, 0.017 mmol) in $CHCl_3$ (0.25 mL). Then vinyl iodide (1.5 µl, 0.017 mmol) and silver tetrafluoroborate (7.0 mg, 0.036 mmol) in trifluorotoluene (1 mL). A white solid (13.7 mg, 99%) was isolated. ^{1}H NMR (500.30 MHz, CD_2Cl_2): δ 8.81 (dd, J = 8.8, 1.4 Hz, 1H), 8.36 (td, J = 7.6, 1.2 Hz, 1H), 8.32 (dt, J = 8.1, 1.4 Hz, 1H), 8.28 (d, J = 8.8 Hz, 1H), 8.05–7.98 (m, 2H), 7.93 (td, J = 7.6, 1.2 Hz, 1H), 7.68–7.60 (m, 2H), 7.55 (td, J = 7.5, 1.3 Hz, 1H), 6.64 (dd, J = 9.6, 2.3 Hz, 1H), 5.86 (dd, 17.2, 1.6 Hz, 1H), 2.36–2.28 (m, 6H), 2.18–2.10 (m, 6H), 2.10–2.04 (m, 6H), 1.82–1.69 (m, 12H). ^{13}C{^{1}H} NMR (125.81 MHz, CD_2Cl_2,): δ 166.1 (d, J = 5.6 Hz), 164.6 (d, J = 112.8 Hz), 151.2 (d, J = 10.7 Hz), 148.1, 143.7, 142.5 (d, J = 2.0 Hz), 138.9 (d, J = 7.9 Hz), 134.1 (d, J = 7.5 Hz), 133.8 (d, J = 2.4 Hz), 133.7 (d, J = 1.3 Hz), 130.4 (d, J = 6.5 Hz), 129.6 (d, J = 6.5 Hz), 129.3, 128.4 (d, J = 5.5 Hz), 126.8 (d, J = 34.9 Hz), 123.0 (d, J = 2.3 Hz), 119.6, 44.6 (d, J = 13.0 Hz), 41.1, 36.1 (d, J = 1.6 Hz), 28.8 (d, J = 9.5 Hz). ^{31}P{^{1}H} NMR (162.02 MHz, CD_2Cl_2): δ 64.54. HR-MS (ESI) m/z calcd for $C_{37}H_{42}AuNP$ [M$^+$] 728.2715, found 728.2713.

^{Ad}P^N^C^{H}Au((*E*)-styryl) (2b)

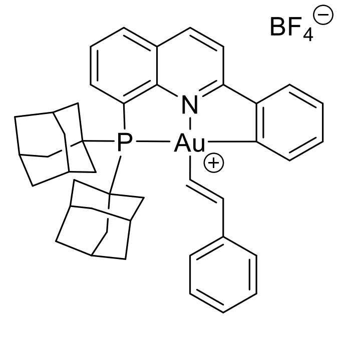

This compound was synthesized by following general procedure D using **I** (8.6 mg, 0.017 mmol) and dimethylsulfidegold(I) chloride (5.0 mg, 0.017 mmol) in $CHCl_3$ (0.25 mL). Then (*E*)-styryl iodide (3.9 mg, 0.017 mmol, E/Z >99:1) and silver tetrafluoroborate (7.0 mg, 0.036 mmol) in trifluorotoluene (1 mL). An off-white solid (12.5 mg, 82%, E/Z >99:1) was isolated. ^{1}H NMR (500.30 MHz, CD_2Cl_2): δ 8.93 (dd, J

= 8.8, 1.4 Hz, 1H), 8.43–8.35 (m, 3H), 8.09 (ddd, *J* = 7.5, 3.5. 1.7 Hz, 1H), 8.04 (td, *J* = 7.7, 1.7 Hz, 1H), 7.95 (dd, *J* = 16.3, 4.2 Hz, 1H), 7.84 (td, *J* = 7.6, 1.6 Hz, 1H), 7.60–7.52 (m, 2H), 7.50 (d, *J* = 7.3 Hz, 2H), 7.43 (t, *J* = 7.6 Hz, 2H), 7.36–7.32 (m, 1H), 7.16 (dd, *J* = 16.2, 1.7 Hz, 1H), 2.38–2.31 (m, 6H), 2.18–2.12 (m, 6H), 2.09–2.04 (m, 6H), 1.82–1.69 (m, 12H). $^{13}C\{^1H\}$ NMR (125.81 MHz, CD_2Cl_2): δ 166.2 (d, *J* = 5.5 Hz), 165.1 (d, *J* = 113.8 Hz), 151.3 (d, *J* = 10.6 Hz), 148.2, 144.2, 142.5 (d, *J* =1.9 Hz), 139.8 (d, *J* = 1.8 Hz), 136.9 (d, *J* = 2.4 Hz), 134.14, 134.07, 133.7, 131.0 (d, *J* = 8.7 Hz), 130.6 (d, *J* = 6.5 Hz), 129.8 (d, *J* = 6.3 Hz), 129.4, 129.3, 128.6 (d, *J* = 5.1 Hz), 128.1, 126.4 (d, *J* = 35.4 Hz), 126.2, 119.9, 44.5 (d, *J* = 13.0 Hz), 41.1, 36.2 (d, *J* = 1.8 Hz), 28.8 (d, *J* = 9.6 Hz). $^{31}P\{^1H\}$ NMR (202.52 MHz, CD_2Cl_2): δ 65.17. HR-MS (ESI) m/z calcd for $C_{43}H_{46}AuNP$ [M^+] 804.3028, found 804.3023.

^{Ad}P^N^C^{H}Au((*Z*)-styryl) (2c)

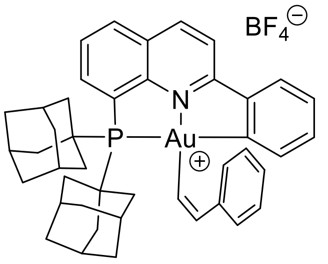

This compound was synthesized by following general procedure D using **I** (8.6 mg, 0.017 mmol) and dimethylsulfidegold(I) chloride (5.0 mg, 0.017 mmol) in $CHCl_3$ (0.25 mL). Then (*Z*)-styryl iodide (3.9 mg, 0.017 mmol, *Z/E* 93:7) and silver tetrafluoroborate (7.0 mg, 0.036 mmol) in trifluorotoluene (1 mL). A white solid (14.6 mg, 96%, *Z/E* 93:7) was isolated. ^1H NMR (500.30 MHz, CD_2Cl_2): δ 8.95 (dd, *J* = 8.8, 1.4 Hz, 1H), 8.41 (d, *J* = 8.6 Hz, 2H), 8.36 (td, *J* = 7.3, 1.2 Hz, 1H), 8.11 (ddd, *J* = 7.7, 3.5. 1.5 Hz, 1H), 8.03 (td, *J* = 7.7, 1.6 Hz, 1H), 7.87–7.79 (m, 2H), 7.75–7.70 (m, 3H), 7.55–7.49 (m, 2H), 7.47 (tdd, *J* = 7.5, 2.9, 1.6 Hz, 1H), 7.23–7.17 (m, 2H), 2.41–2.29 (m, 3H), 2.15–2.06 (m, 9H), 1.96–1.87 (m, 3H), 1.83–1.69 (m, 9H), 1.48–1.41 (m, 3H), 1.21–1.13 (m, 3H). $^{13}C\{^1H\}$ NMR (125.81 MHz, CD_2Cl_2): δ 166.3 (d, *J* = 5.9 Hz), 162.3 (d, *J* = 111.7 Hz), 151.1 (d, *J* = 10.5 Hz), 148.3, 144.2, 142.3 (d, *J* =1.7 Hz), 138.2, 134.2 (d, *J* = 9.4 Hz), 134.1 (d, *J* = 4.0 Hz), 133.8 (d, *J* = 1.6 Hz), 132.3 (d, *J* = 2.5 Hz), 130.7 (d, *J* = 8.5 Hz), 130.6 (d, *J* = 5.9 Hz), 129.7 (d, *J* = 6.4 Hz), 129.3, 129.1, 128.8 (d, *J* = 5.1 Hz), 128.2, 128.1, 126.7 (d, *J* = 35.4 Hz), 119.9, 44.5 (d, *J* = 13.5 Hz), 44.3 (d, *J* = 12.4 Hz), 41.1, 40.7, 36.2 (d, *J* = 2.0 Hz), 35.6 (d, *J* = 1.7 Hz), 28.8 (d, *J* = 9.5 Hz), 28.6 (d, *J* = 9.5 Hz). $^{31}P\{^1H\}$ NMR (202 MHz, CD_2Cl_2): δ 70.21. HR-MS (ESI) m/z calcd for $C_{43}H_{46}AuNP$ [M^+] 804.3028, found 804.3023.

^{Ad}P^N^C^{H}AuCl (3)

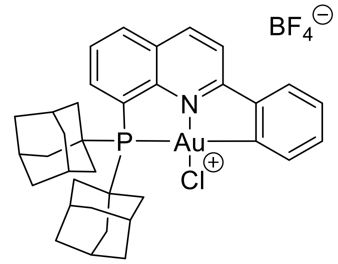

This compound was synthesized by following general procedure E using **I** (33.4 mg, 0.066 mmol) and dimethylsulfidegold(I) chloride (19.4 mg, 0.066 mmol) in $CHCl_3$ (2 mL). Then Selectfluor (35.1 mg, 0.099 mmol) in acetonitrile (3 mL). An off-white solid (46.5mg, 86%) was isolated. ^{1}H NMR (500.30 MHz, CD_2Cl_2): δ 9.06 (dd, *J* = 8.8, 1.5 Hz, 1H), 8.50 (dt, *J* = 8.1, 1.4 Hz, 1H), 8.43 (td, *J* = 7.4, 1.2 Hz, 1H), 8.39 (d, *J* = 8.8 Hz, 1H), 8.10 (ddd, *J* = 8.1, 7.5, 1.6 Hz, 1H), 8.06–7.99 (m, 2H), 7.68 (tdd, *J* = 7.7, 3.7, 1.5 Hz, 1H), 7.59 (tt, *J* = 7.5, 1.0 Hz, 1H), 2.40–2.21 (m, 12H), 2.12–2.06 (m, 6H), 1.82–1.70 (m, 12H). ^{13}C{^{1}H} NMR (125.81 MHz, CD_2Cl_2): δ 170.1 (d, *J* = 120.2 Hz), 169.1 (d, *J* = 5.7 Hz), 151.9 (d, *J* = 11.4 Hz), 146.8, 146.3, 143.1 (d, *J* = 1.9 Hz), 135.0 (d, *J* = 2.0 Hz), 134.7 (d, *J* = 9.0 Hz), 130.9 (d, *J* = 7.0 Hz), 130.5 (d, *J* = 1.0 Hz), 130.44, 130.40, 129.3 (d, *J* = 5.8 Hz), 123.5 (d, *J* = 36.7 Hz), 120.5, 45.5 (d, *J* = 11.3 Hz), 41.0, 36.1 (d, *J* = 1.9 Hz), 28.8 (d, *J* = 9.6 Hz). ^{31}P{^{1}H} NMR (202.52 MHz, CD_2Cl_2): δ 72.70. HR-MS (ESI) m/z calcd for $C_{35}H_{39}AuClNP$ [M^+] 736.2169, found 736.2163.

^{Ad}P^N^C^{H}Au(Alkyne1) (4a)

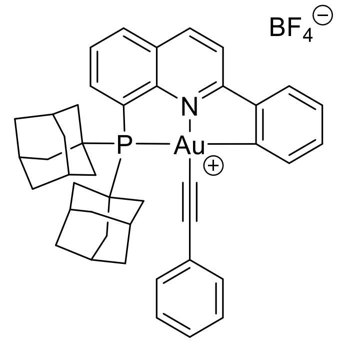

This compound was synthesized by following general procedure F using **3** (20.6 mg, 0.025 mmol), copper iodide (5.0 mg, 0.05 mmol), phenylacetylene **Alkyne1** (5.1 mg, 0.05 mmol) and triethylamine (5.1 mg, 0.5 mmol) in dichloromethane (2.5 mL). An off-white solid (20.0 mg, 90%) was isolated. ^{1}H NMR (500.30 MHz, CD_2Cl_2): δ 9.02 (dd, *J* = 8.8, 1.5 Hz, 1H), 8.46 (d, *J* = 8.0 Hz, 1H), 8.43 (t, *J* = 7.5 Hz, 1H), 8.38 (d, *J* = 8.8 Hz, 1H), 8.32 (td, *J* = 8.0, 1.1 Hz, 1H), 8.12–8.05 (m, 2H), 7.67 (tdd, *J* = 7.5, 3.2, 1.4 Hz, 1H), 7.60 (t, *J* = 7.6 Hz, 1H), 7.56 (dd, *J* = 7.8, 1.9 Hz, 2H), 7.44–7.38 (m, 3H), 2.42–2.29 (m, 12H), 2.12–2.06 (m, 6H), 1.82–1.73 (m, 12H). ^{13}C{^{1}H} NMR (125.81 MHz, CD_2Cl_2): δ 168.5 (d, *J* = 5.5 Hz), 164.6 (d, *J* = 113.6 Hz), 152.2 (d, *J* = 11.1 Hz), 147.7, 145.1, 142.9 (d, *J* = 1.7 Hz), 134.8 (d, *J* = 8.4 Hz), 134.6 (2C), 131.7, 130.7 (d, J = 7.3 Hz), 130.3 (d, *J* = 6.4 Hz), 129.7, 128.9 (2C), 128.7, 125.3 (d, J = 35.9 Hz), 125.2, 120.1, 107.6, 83.3 (d, J = 13.7 Hz), 45.0 (d, J = 12.5 Hz), 41.2, 36.1 (d, J = 1.9 Hz), 28.9 (d, J = 9.8 Hz). ^{31}P{^{1}H} NMR (202.52 MHz, CD_2Cl_2): δ 75.50. HR-MS (ESI) m/z calcd for $C_{43}H_{44}AuNP$ [M^+] 802.2877, found 802.2864.

^{Ad}P^N^C^{H}Au(Alkyne2) (4b)

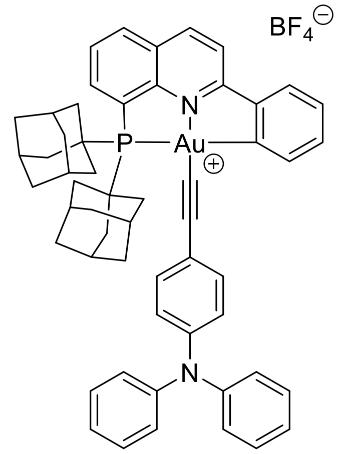

This compound was synthesized by following general procedure F using **3** (16.5 mg, 0.02 mmol), copper iodide (4.0 mg, 0.04 mmol), 4-ethynyltriphenylamine **Alkyne2** (10.8 mg, 0.04 mmol) and triethylamine (4.0 mg, 0.4 mmol) in dichloromethane (2.5 mL). An orange solid (20.0 mg, 95%) was isolated. ^{1}H NMR (500.30 MHz, CD_2Cl_2): δ 8.99 (dd, *J* = 8.9, 1.5 Hz, 1H), 8.46–8.39 (m, 2H), 8.36 (d, *J* = 8.9 Hz, 1H), 8.32 (td, *J* = 8.2, 1.3 Hz, 1H), 8.10–8.04 (m, 2H), 7.67 (tdd, *J* = 7.5, 3.2, 1.4 Hz, 1H), 7.59 (td, *J* = 7.5, 1H), 7.39 (d, *J* = 8.6 Hz, 2H), 7.31 (t, *J* = 8.1 Hz, 4H), 7.14 (d, *J* = 7.4 Hz, 4H), 7.10 (td, *J* = 7.3, 1.2 Hz, 2H), 7.03 (d, *J* = 8.7 Hz, 2H), 2.42–2.28 (m, 12H), 2.12–2.05 (m, 6H), 1.81–1.72 (m, 12H). ^{13}C{^{1}H} NMR (125.81 MHz, CD_2Cl_2): δ 168.5 (d, *J* = 5.5 Hz), 164.7 (d, *J* = 114.3 Hz), 152.2 (d, *J* = 11.4 Hz), 148.5, 147.7, 147.6, 145.0, 142.8 (d, *J* = 1.9 Hz), 134.7 (d, *J* = 8.4 Hz), 134.64, 134.58 (d, *J* = 2.2 Hz), 132.6, 130.7 (d, *J* = 7.0 Hz), 130.2 (d, *J* = 6.4 Hz), 129.8, 129.7, 128.9 (d, *J* = 5.3 Hz), 125.52 (d, *J* = 8.2 Hz), 125.47, 124.1, 122.5, 120.1, 117.9, 107.8, 82.2 (d, *J* = 13.8 Hz), 44.9 (d, *J* = 12.5 Hz), 41.2, 36.1 (d, *J* = 1.9 Hz), 28.9 (d, *J* = 9.8 Hz). ^{31}P{^{1}H} NMR (202.52 MHz, CD_2Cl_2): δ 75.21. HR-MS (ESI) m/z calcd for $C_{55}H_{53}AuN_2P$ [M$^+$] 969.3606, found 969.3588.

^{Ad}P^N^C^{H}Au(Alkyne3) (4c)

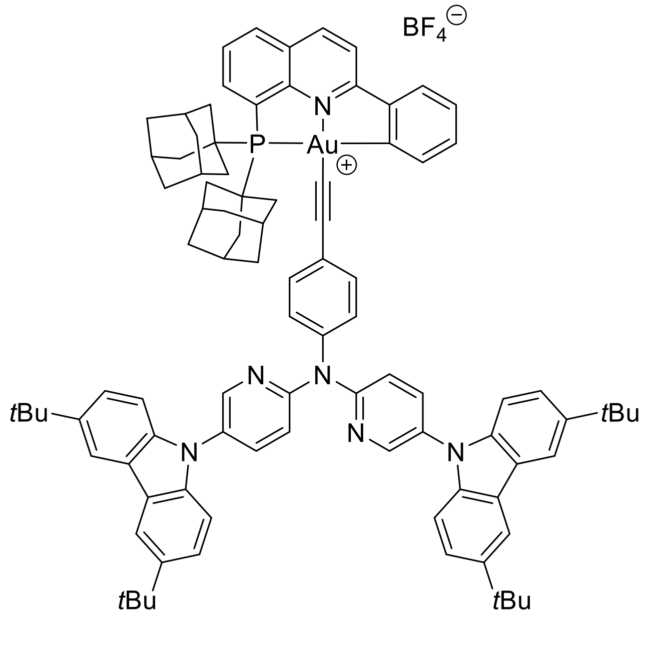

This compound was synthesized by following general procedure F using **3** (4.1 mg, 0.005 mmol), copper iodide (1.0 mg, 0.01 mmol), **Alkyne3** (6.2 mg, 0.075 mmol) and triethylamine (1.0 mg, 0.01 mmol) in dichloromethane (1 mL). A yellowish solid (6.0 mg, 74%) was isolated. ^{1}H NMR (400.13 MHz, CD_2Cl_2): δ 9.01 (dd, *J* = 8.9, 1.3 Hz, 1H), 8.59 (d, *J* = 1.9 Hz, 2H), 8.48–8.33 (m, 4H), 8.18 (d, *J* = 1.4 Hz, 4H), 8.12–8.06 (m, 2H), 7.88 (dd, *J* = 8.8, 2.6 Hz, 2H), 7.72–7.65 (m, 3H), 7.61 (t, *J* = 7.5 Hz, 1H), 7.53 (dd, *J* = 8.7, 2.0 Hz, 4H), 7.47–7.39 (m, 8H), 2.45–2.30 (m, 12H), 2.15–2.08 (m, 6H), 1.78 (br, 12H), 1.47 (s, 36H). ^{13}C{^{1}H} NMR (125.81 MHz, CD_2Cl_2): δ 168.6 (d, *J* = 5.5 Hz), 164.7 (d, *J* = 113.6 Hz), 156.3, 152.2 (d, *J* = 11.1 Hz), 147.7, 146.9, 145.2, 145.1, 143.9, 142.8, 139.7, 136.4, 134.9 (d, *J* = 8.5 Hz), 134.7, 134.6, 133.1, 130.7 (d, *J* = 7.1 Hz), 130.5, 130.2 (d, *J* = 6.3 Hz), 129.8, 128.9 (d, *J* = 5.6 Hz), 127.9, 125.4 (d, *J* = 35.6 Hz), 124.3, 123.9, 122.6, 120.1, 118.0, 116.9, 109.3, 107.3, 83.9 (d, *J* = 14.0 Hz), 45.0 (d, *J* = 12.2 Hz), 41.3, 36.1, 35.1, 32.1, 28.9 (d, *J* = 9.8 Hz). ^{31}P{^{1}H} NMR (202.52 MHz, CD_2Cl_2): δ 75.72. HR-MS (ESI) m/z calcd for $C_{93}H_{97}AuN_6P$ [M+H$^+$] 1526.7245, found 1526.7183.

^{Ad}P^N^C^{H}Au(Alkyne4) (4d)

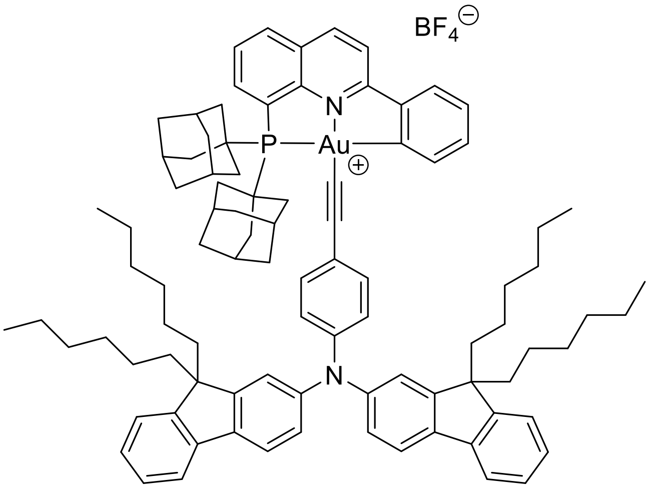

This compound was synthesized by following general procedure F using **3** (8.2 mg, 0.01 mmol), copper iodide (2.0 mg, 0.02 mmol), **Alkyne4** (11.7 mg, 0.015 mmol) and triethylamine (2.0 mg, 0.02 mmol) in dichloromethane (1 mL). A red solid (11.8 mg, 75%) was isolated. ^{1}H NMR (500.30 MHz, CD_2Cl_2) δ 8.98 (dd, *J* = 8.8, 1.4 Hz, 1H), 8.46–8.39 (m, 2H), 8.37–8.30 (m, 2H), 8.10–8.04 (m, 2H), 7.67–7.57 (m, 6H), 7.41–7.37 (m, 2H), 7.36–7.26 (m, 6H), 7.25 (d, *J* = 2.0 Hz, 2H), 7.14–7.06 (m, 4H), 2.43–2.29 (m, 12H), 2.12–2.05 (m, 6H), 1.99–1.84 (m, 8H), 1.82–1.72 (m, 12H), 1.21–1.12 (m, 8H), 1.12–1.03 (m, 16H), 0.81 (t, *J* = 7.2 Hz, 12H), 0.75–0.56 (m, 8H). ^{13}C{^{1}H} NMR (125.81 MHz, CD_2Cl_2) δ 168.5 (d, *J* = 5.5 Hz), 164.7 (d, *J* = 114.2 Hz), 152.7, 152.2 (d, *J* = 11.3 Hz), 151.0, 148.8, 147.7, 146.8, 145.0, 142.8, 141.2, 137.6, 134.7 (d, *J* = 8.4 Hz), 134.62, 134.59, 132.6, 130.7 (d, *J* = 7.0 Hz), 130.2 (d, *J* = 6.3 Hz), 129.7, 128.9 (d, *J* = 5.4 Hz), 127.2, 127.0, 125.4 (d, *J* = 35.8 Hz), 124.4, 123.3, 122.0, 120.8, 120.2, 120.1, 119.6, 117.4, 107.9, 82.2 (d, *J* = 13.8 Hz), 55.5, 45.0 (d, *J* = 12.5 Hz), 41.2, 40.6, 36.1 (d, *J* = 1.9 Hz), 32.0, 30.0, 28.9 (d, *J* = 9.8 Hz), 24.2, 23.0, 14.3. ^{31}P{^{1}H} NMR (202.52 MHz, CD_2Cl_2): δ 75.18. HR-MS (ESI) m/z calcd for $C_{93}H_{109}AuN_2P$ [M$^+$] 1481.7989, found 1481.7992.

4. Control experiments

A) Reactivity tests

In order to investigate the ability of the (P^N^C)gold(III) aryl complexes to undergo reductive elimination we subjected them to the conditions listed in Table S1. The reductive elimination reactions leading to the formation of C(sp^2)-C(sp^2) bonds in gold(III) complexes are strongly dependent on the structure of the ligands. These processes were widely studied in catalytic applications with various bidentate ligands, including (P^N)[9] and tridentate as reported on the example of easily accessible (C^N^C) complexes.[10]

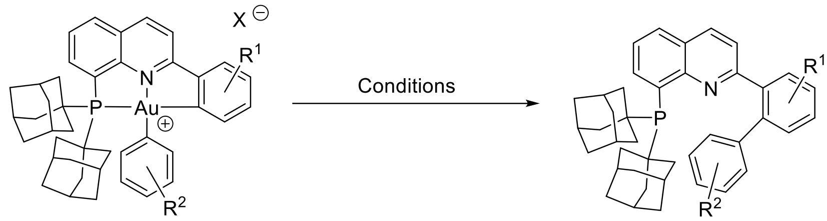

1a, b, d

Table S1. Tested conditions for reductive elimination of (P^N^C)gold(III) aryl complexes.

Compound	Conditions	Result
**1a**	100 °C, 1,2-DCB, 24 h	n.r.
**1e**	100 °C, 1,2-DCE, 24 h	n.r.
**1e**	100 °C, CH_3CN, 24 h	n.r.
**1g**	100 °C, 1,2-DCE, 3 h	n.r.
**1g**	150 °C, MW, 1,2-DCB, 1 h	n.r.
**1e**	HBF_4 • Et_2O (10 equiv), CD_2Cl_2, 2 h	n.r.

These results showed lack of reactivity under the chosen conditions, which demonstrates high stability of the synthesized complexes towards reductive elimination under thermal conditions as well as protic environment.

B) Oxidative addition of (iodoethynyl)benzene

Following the general procedure D: compound **I** (8.6 mg, 0.017 mmol) and dimethylsulfidegold(I) chloride (5.0 mg, 0.017 mmol) were dissolved in $CHCl_3$ (0.25 mL) and stirred for 1h. A white solid obtained after the work-up was transferred to a Schlenk tube at 0 °C containing (iodoethynyl)benzene (4.2 µl, 0.017 mmol) and silver tetrafluoroborate (7.0 mg, 0.036 mmol) in trifluorotoluene (1 mL). The reaction was carried out independently for 1 h and 2 h. After work-up, **4a** was detected as major species in the reaction mixture. No improvement in the reaction outcome was observed by increasing the reaction time.

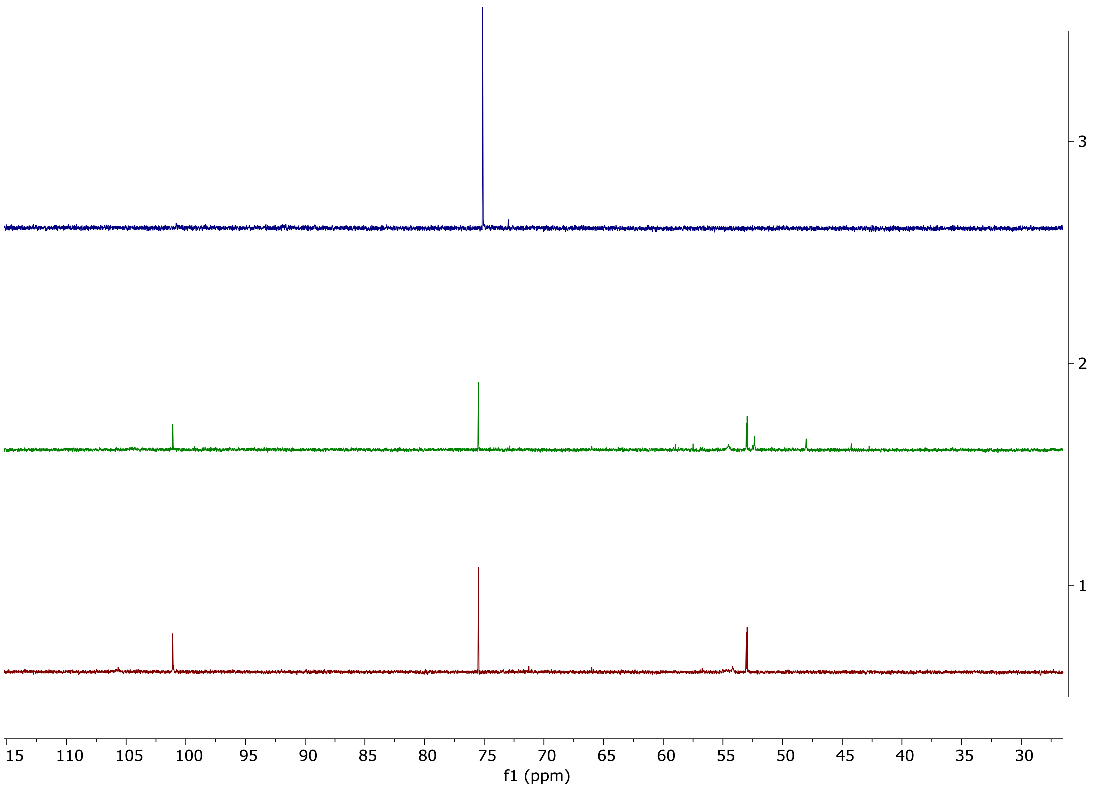

Figure S1. $^{31}P\{^1H\}$ NMR traces of **4a** after oxidative addition. From top to bottom: reference compound, crude mixture after 1 h, crude mixture after 2 h.

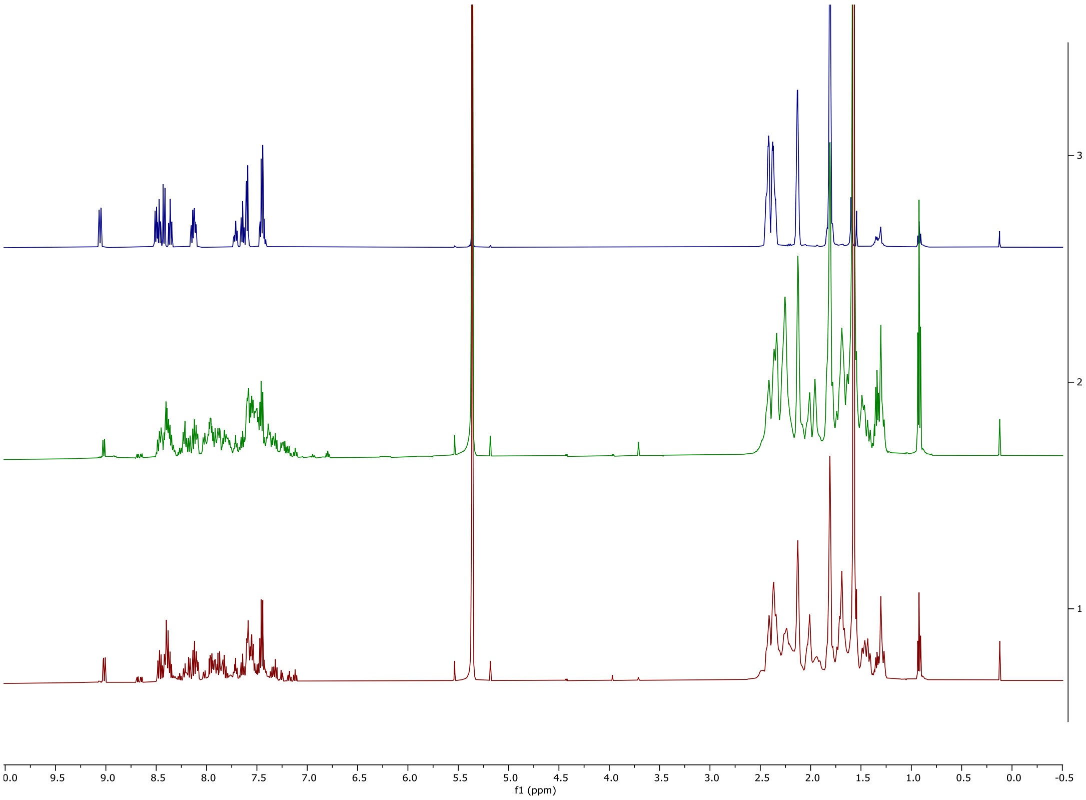

Figure S2. ^1H NMR traces of **4a** after oxidative addition. From top to bottom: reference compound, crude mixture after 1 h, crude mixture after 2 h.

5. Photophysical properties of compounds 4a-d

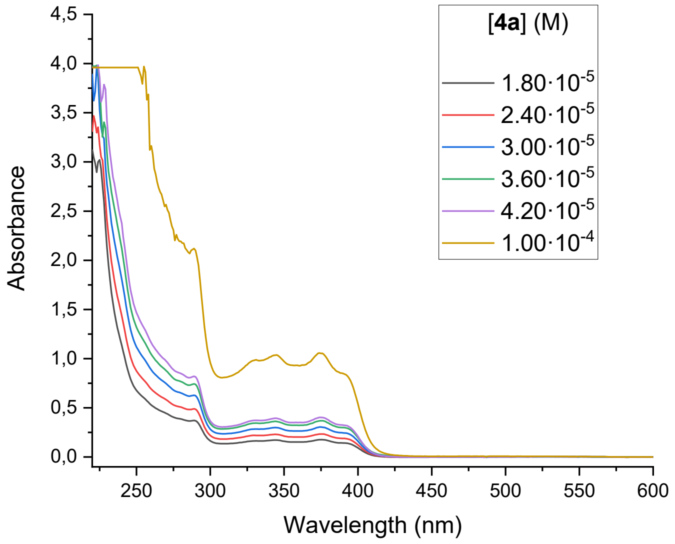

Figure S3. Absorption of compound **4a** in dichloromethane at 298 K in the concentration range $1.80 \cdot 10^{-5}$ M–$1.00 \cdot 10^{-4}$ M.

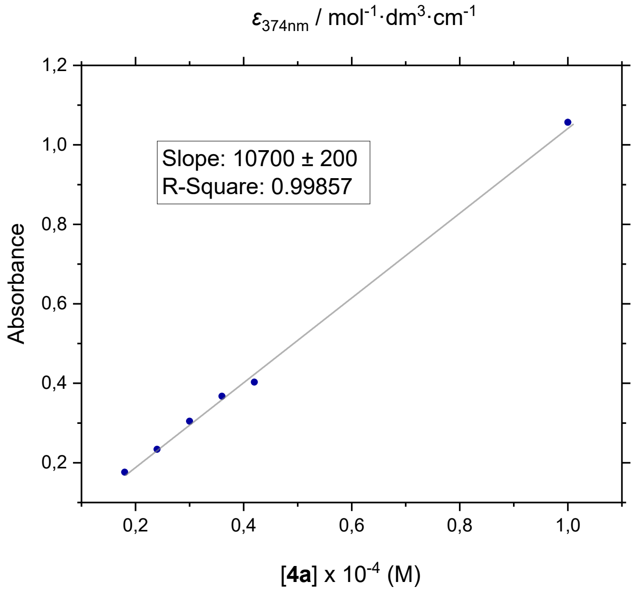

Figure S4. Molar absorption coefficient ε_{374nm} of compound **4a** in dichloromethane at 298 K.

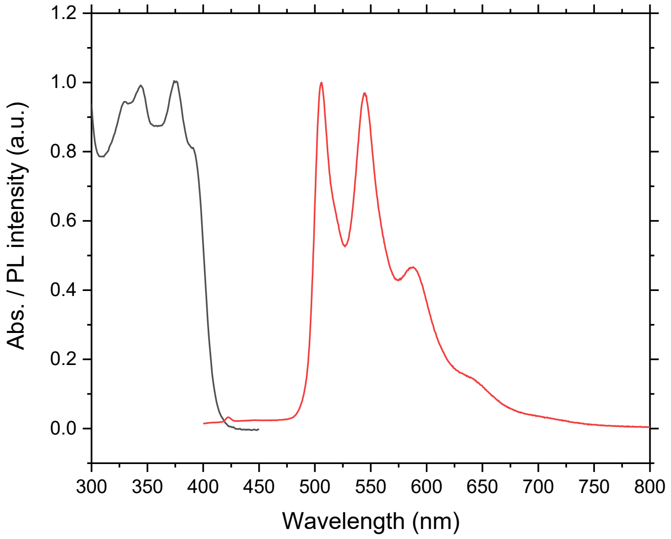

Figure S5. Absorption (in black) and PL spectra (in red, excitation at 375 nm) of **4a** in dichloromethane at 298 K.

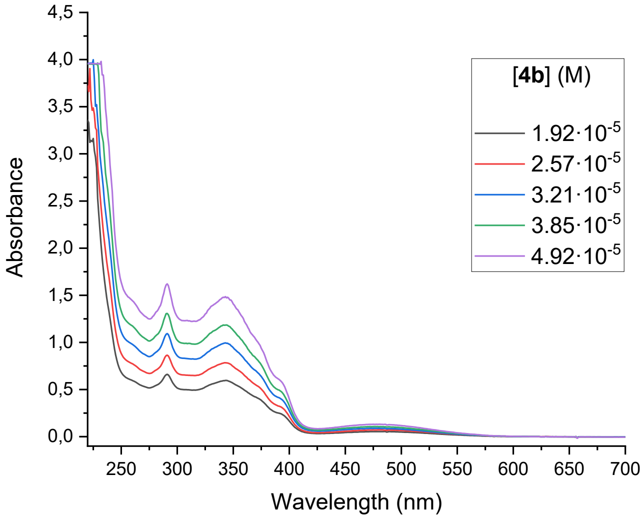

Figure S6. Absorption of compound **4b** in dichloromethane at 298 K in the concentration range $1.92{\cdot}10^{-5}$ M–$4.92{\cdot}10^{-5}$ M.

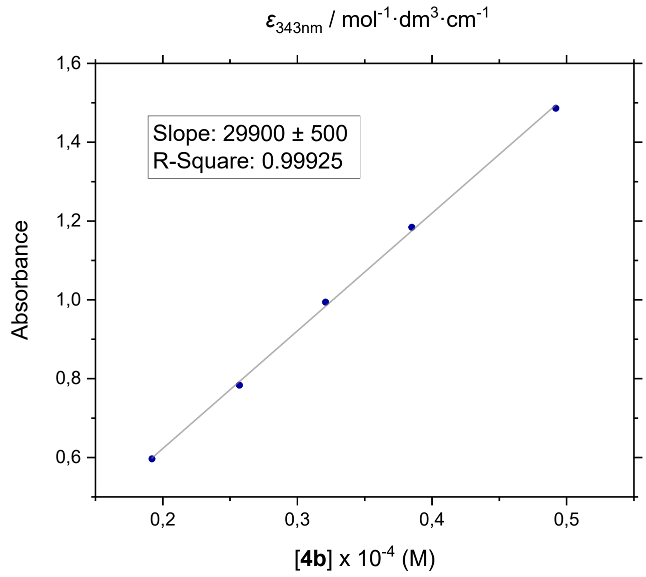

Figure S7. Molar absorption coefficient ε_{343nm} of compound **4b** in dichloromethane at 298 K.

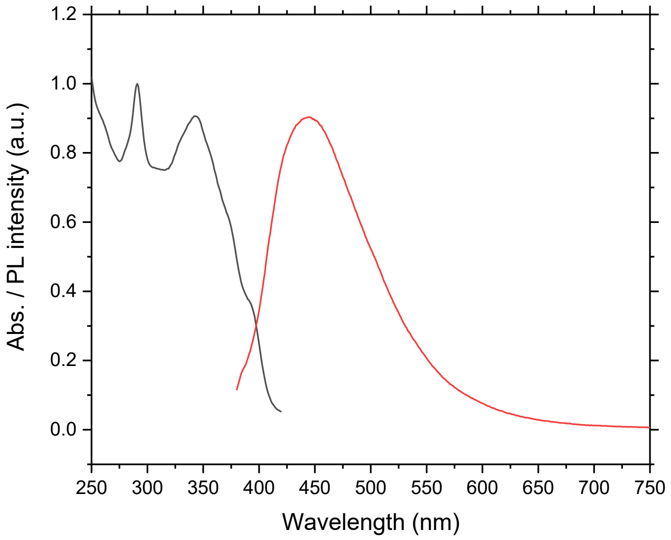

Figure S8. Absorption (in black) and PL spectra (in red, excitation at 345 nm) of **4b** in dichloromethane at 298 K.

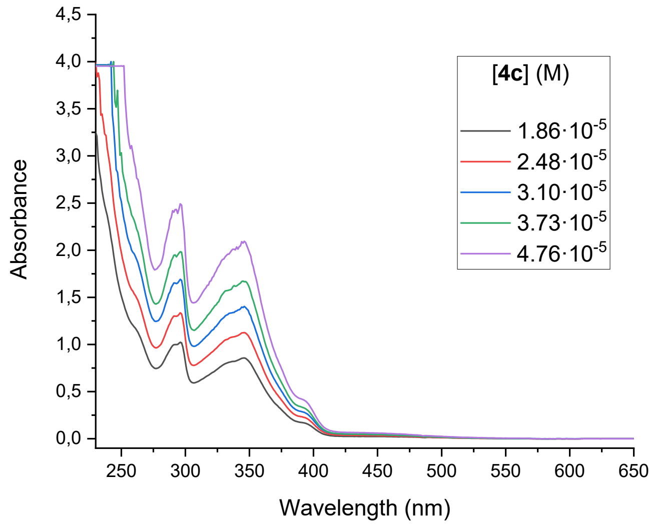

Figure S9. Absorption of compound **4c** in dichloromethane at 298 K in the concentration range $1.86 \cdot 10^{-5}$ M–$4.76 \cdot 10^{-5}$ M.

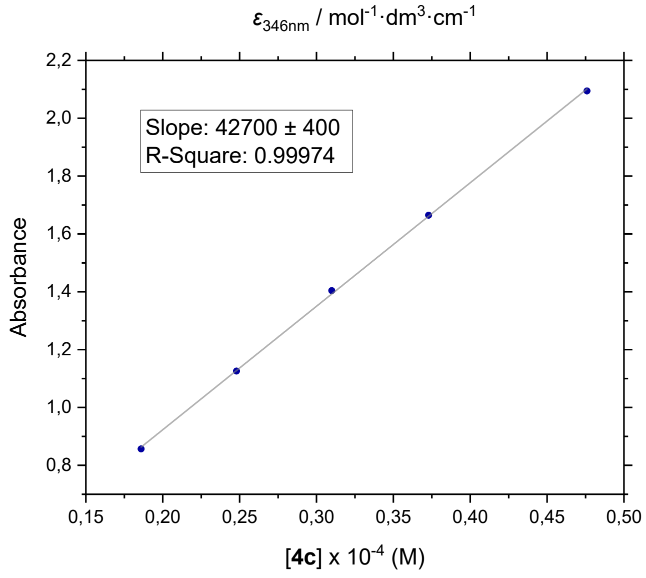

Figure S10. Molar absorption coefficient ε_{346nm} of compound **4c** in dichloromethane at 298 K.

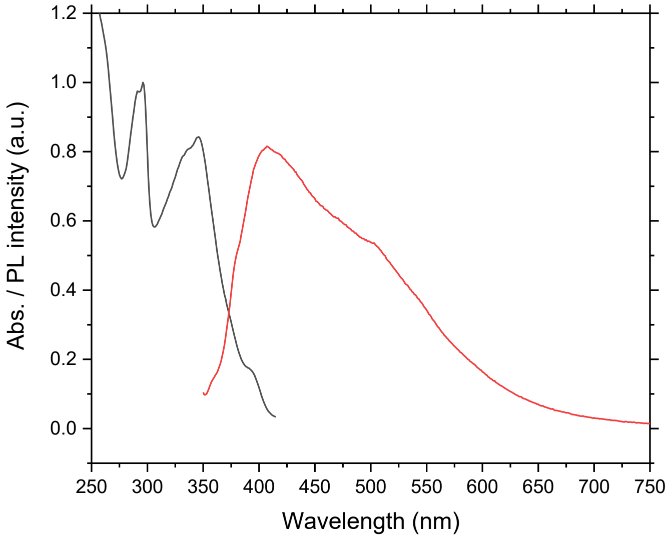

Figure S11. Absorption (in black) and PL spectra (in red, excitation at 340 nm) of **4c** in dichloromethane at 298 K.

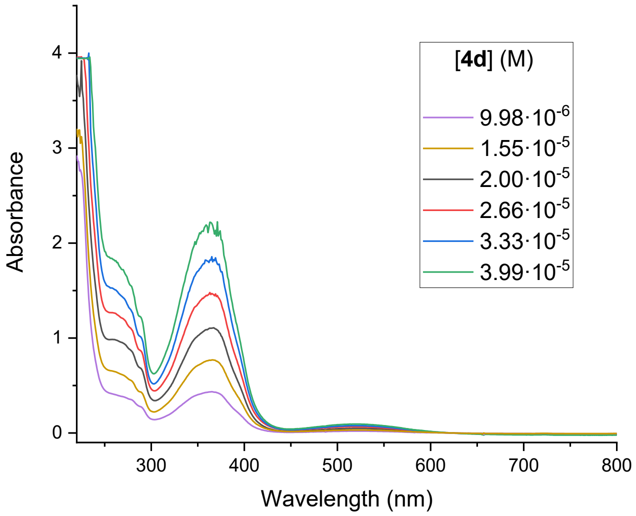

Figure S12. Absorption of compound **4d** in dichloromethane at 298 K in the concentration range $9.98{\cdot}10^{-6}$ M–$3.99{\cdot}10^{-5}$ M.

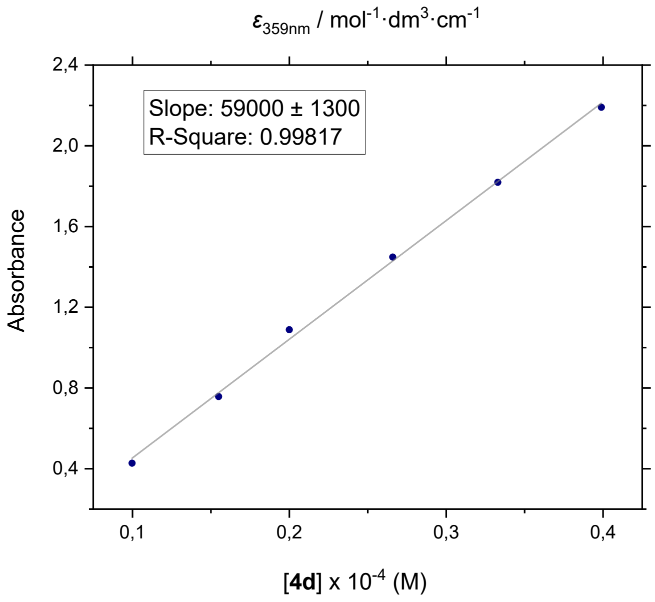

Figure S13. Molar absorption coefficient ε_{359nm} of compound **4d** in dichloromethane at 298 K.

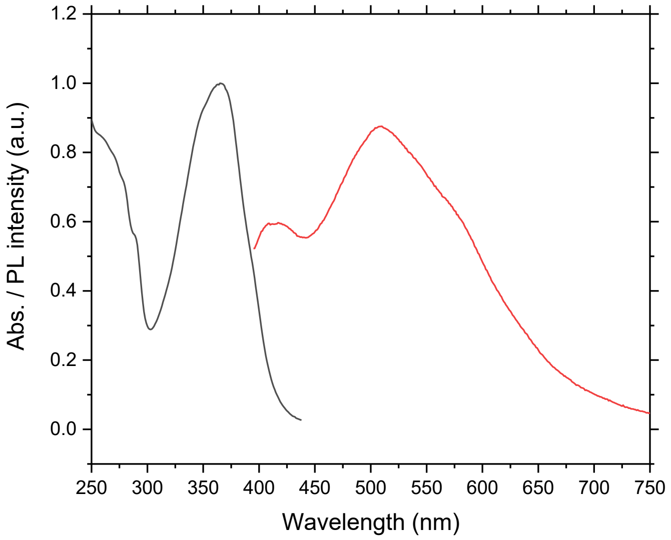

Figure S14. Absorption (in black) and PL spectra (in red, excitation at 345 nm) of **4d** in dichloromethane at 298 K.

6. NMR spectra of new compounds

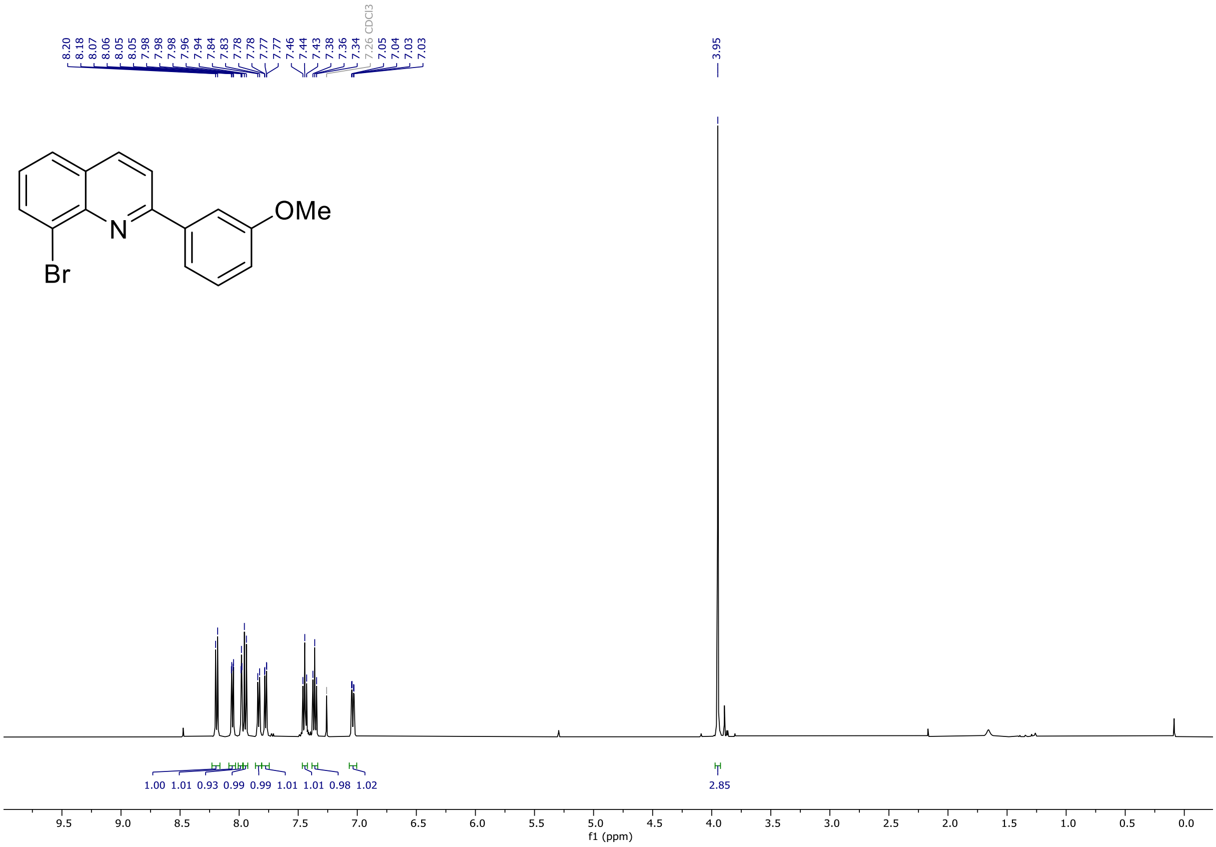

Figure S15. ^{1}H NMR (500.30 MHz, $CDCl_3$, 298 K) spectrum of compound **S4**.

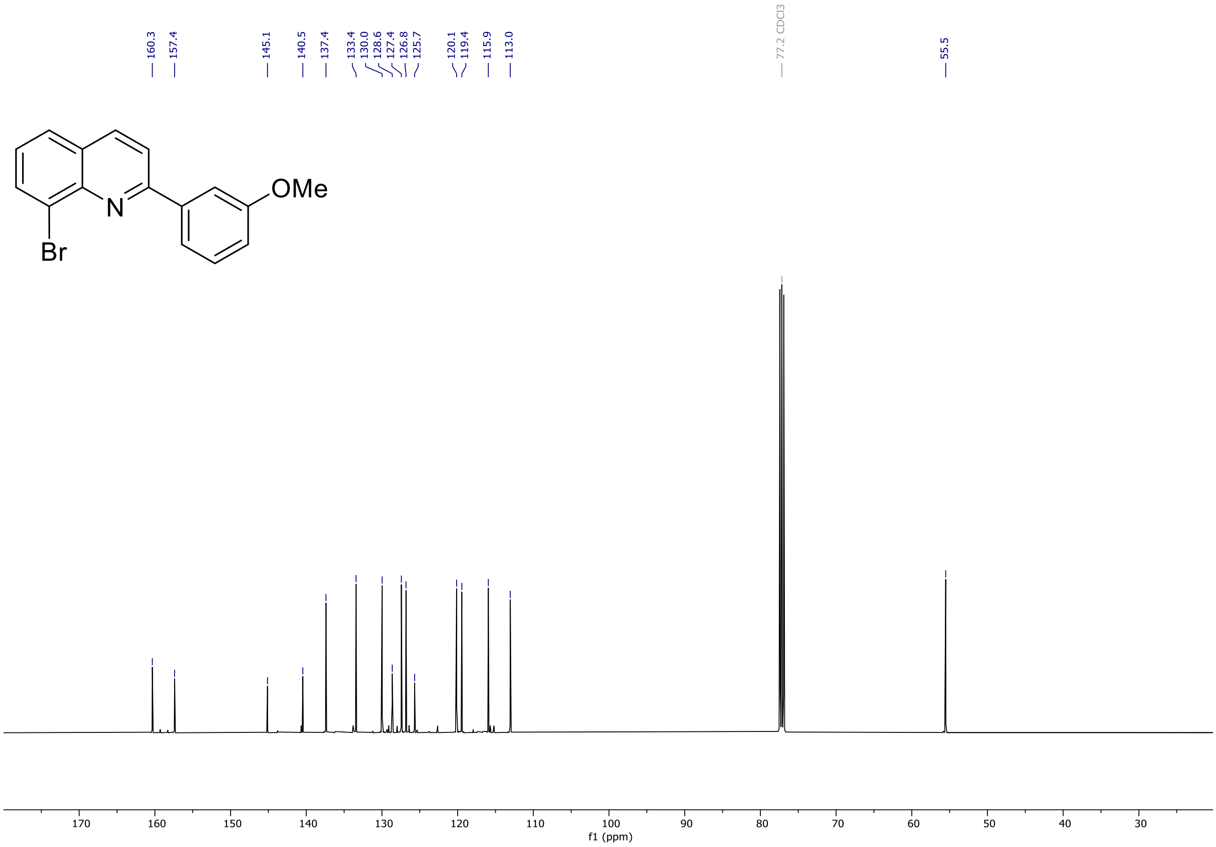

Figure S16. $^{13}C\{^{1}H\}$ NMR (125.81 MHz, $CDCl_3$, 298 K) of compound **S4**.

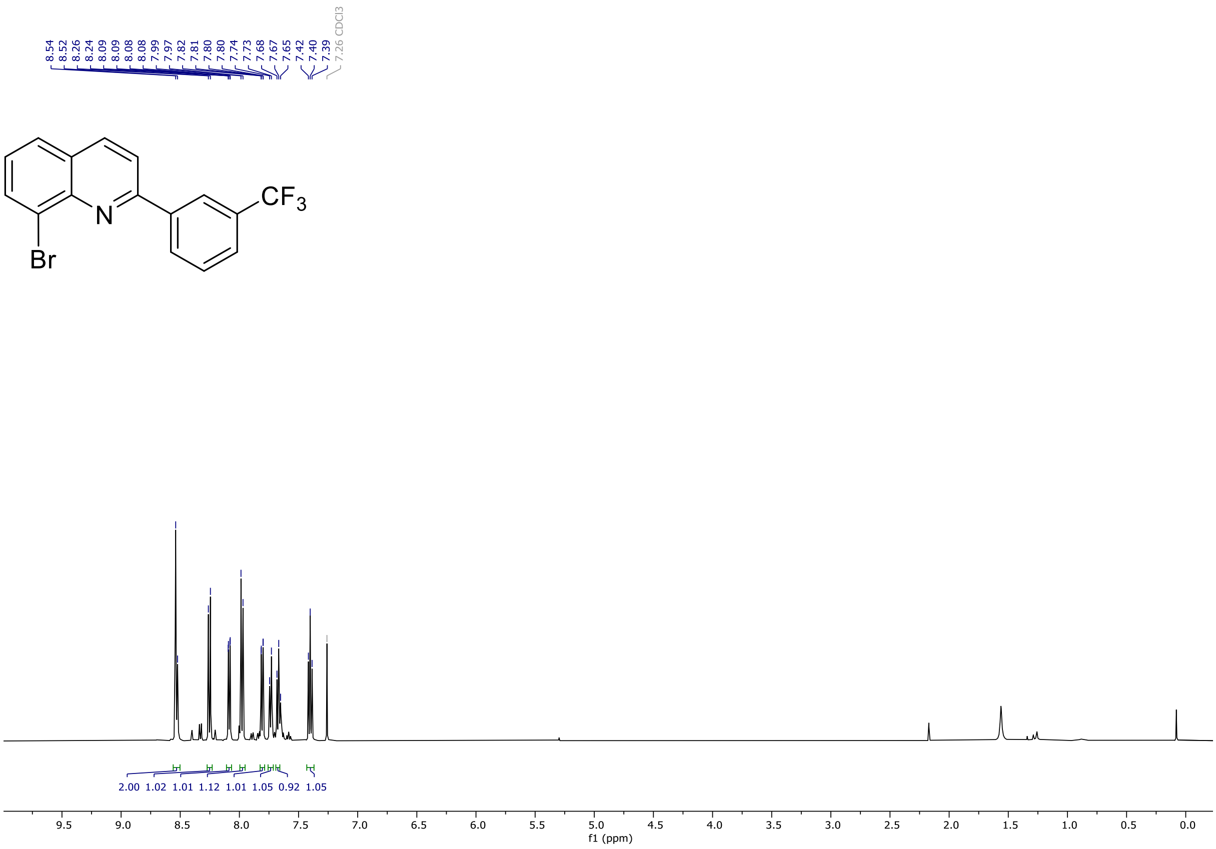

Figure S17. ^{1}H NMR (500.30 MHz, $CDCl_3$, 298 K) of compound **S5**.

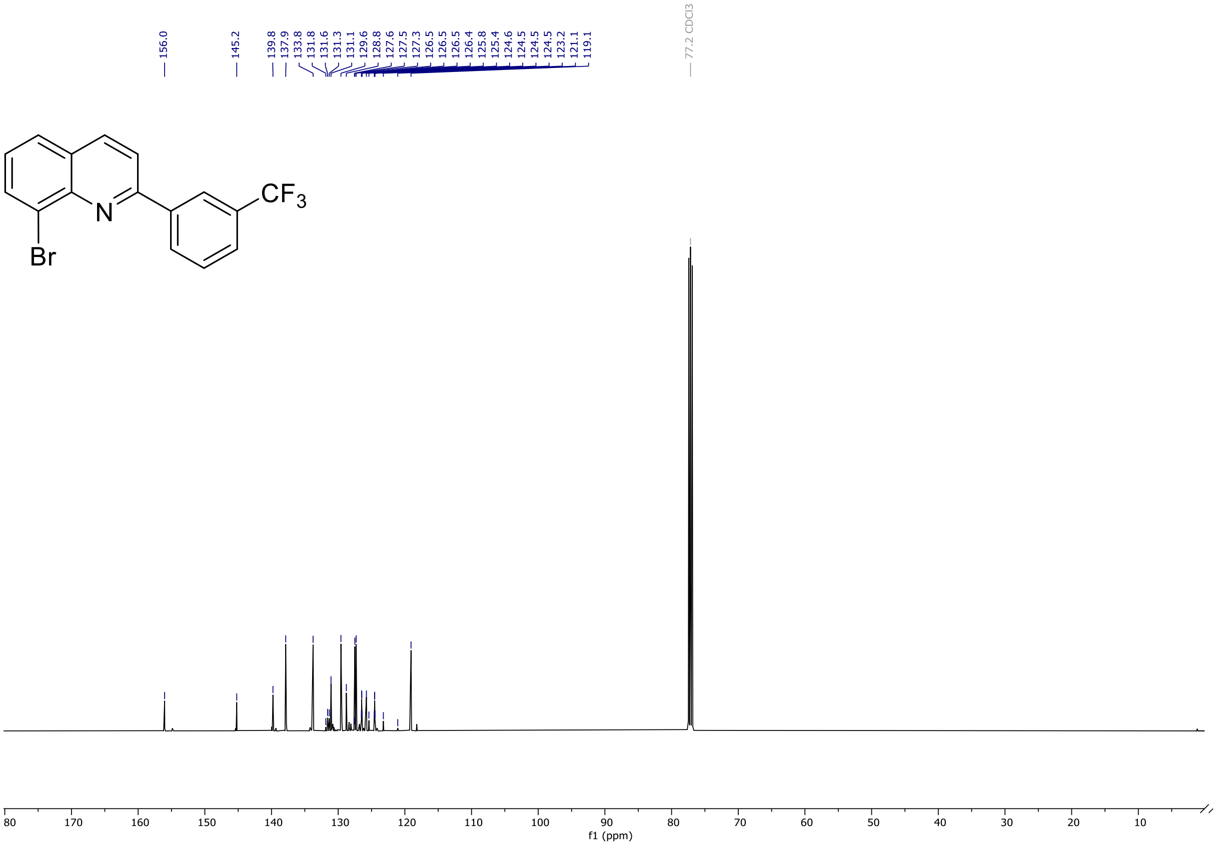

Figure S18. $^{13}C\{^{1}H\}$ NMR (125.81 MHz, $CDCl_3$, 298 K) of compound **S5**.

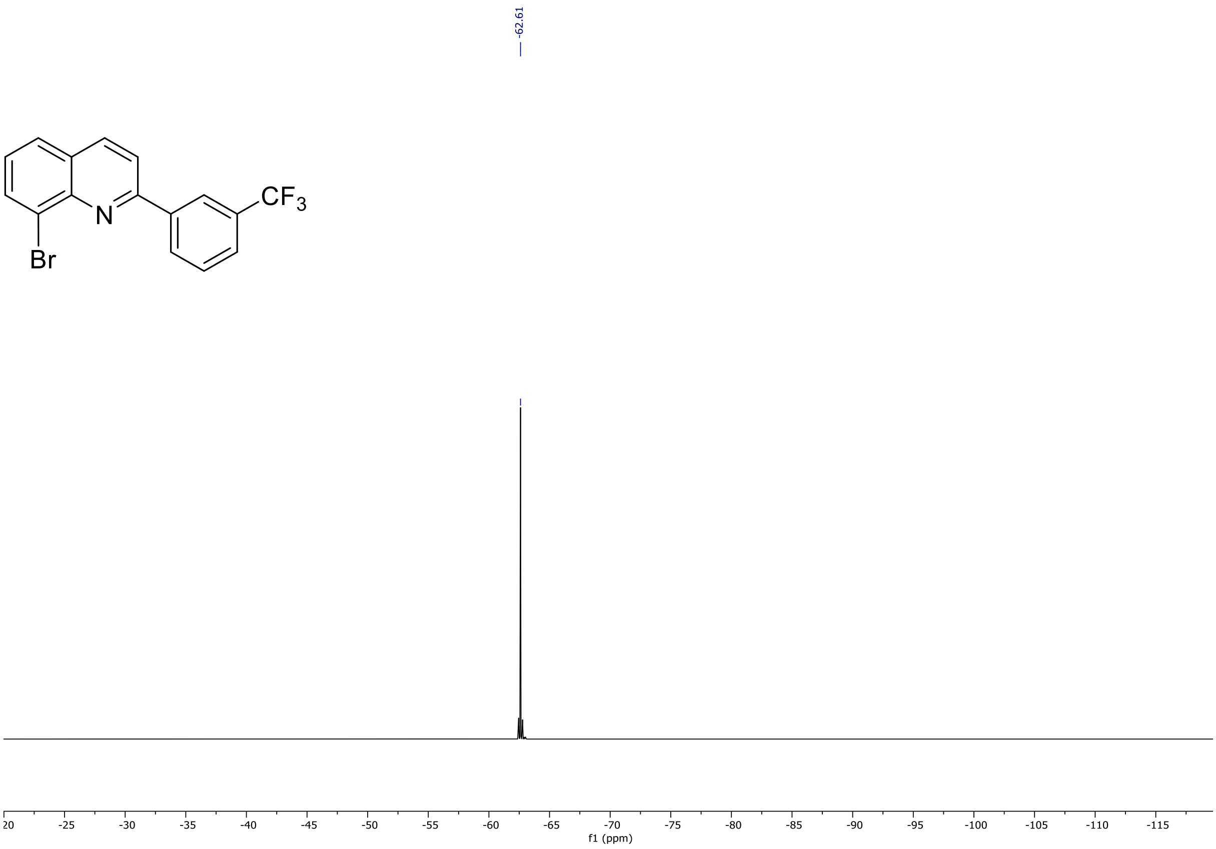

Figure S19. ^{19}F NMR (470.71 MHz, $CDCl_3$, 298 K) of compound **S5**.

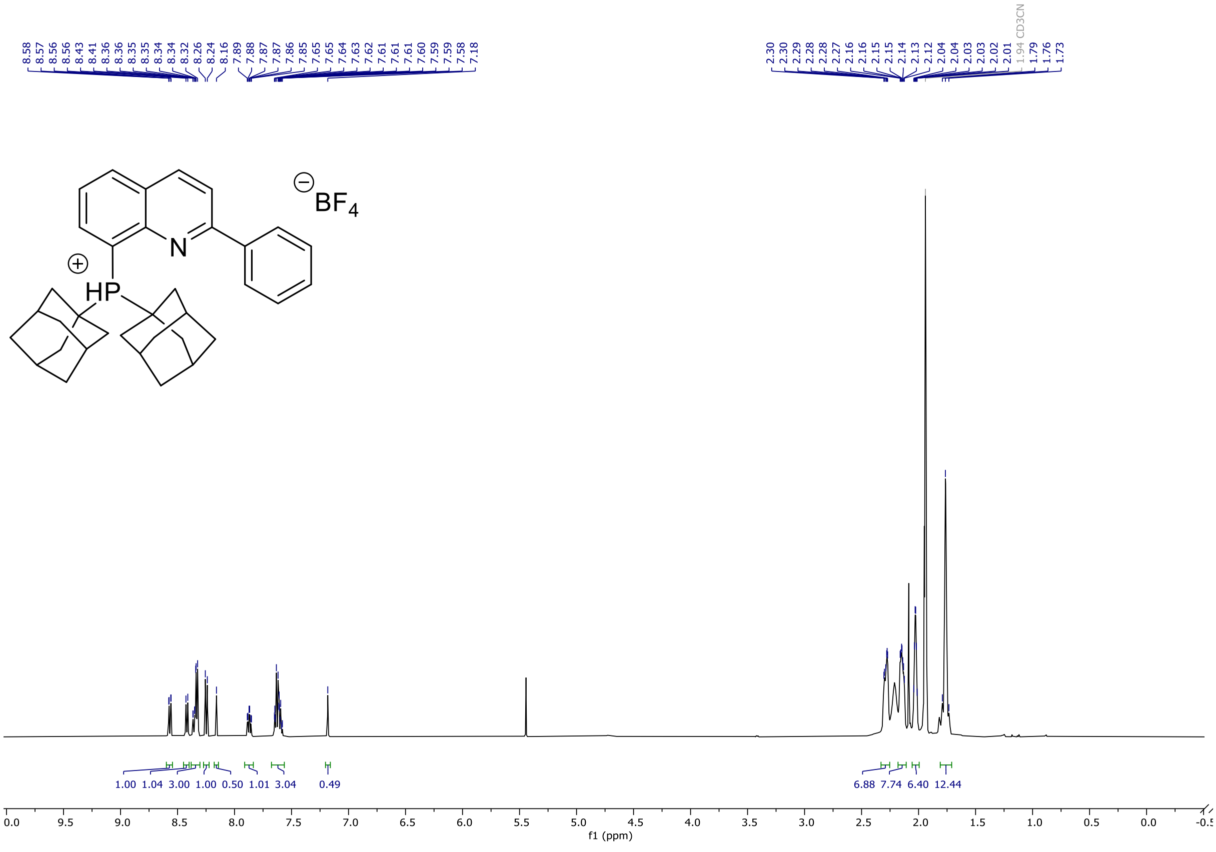

Figure S20. ^{1}H NMR (500.30 MHz, CD_3CN, 298 K) of compound **I**.

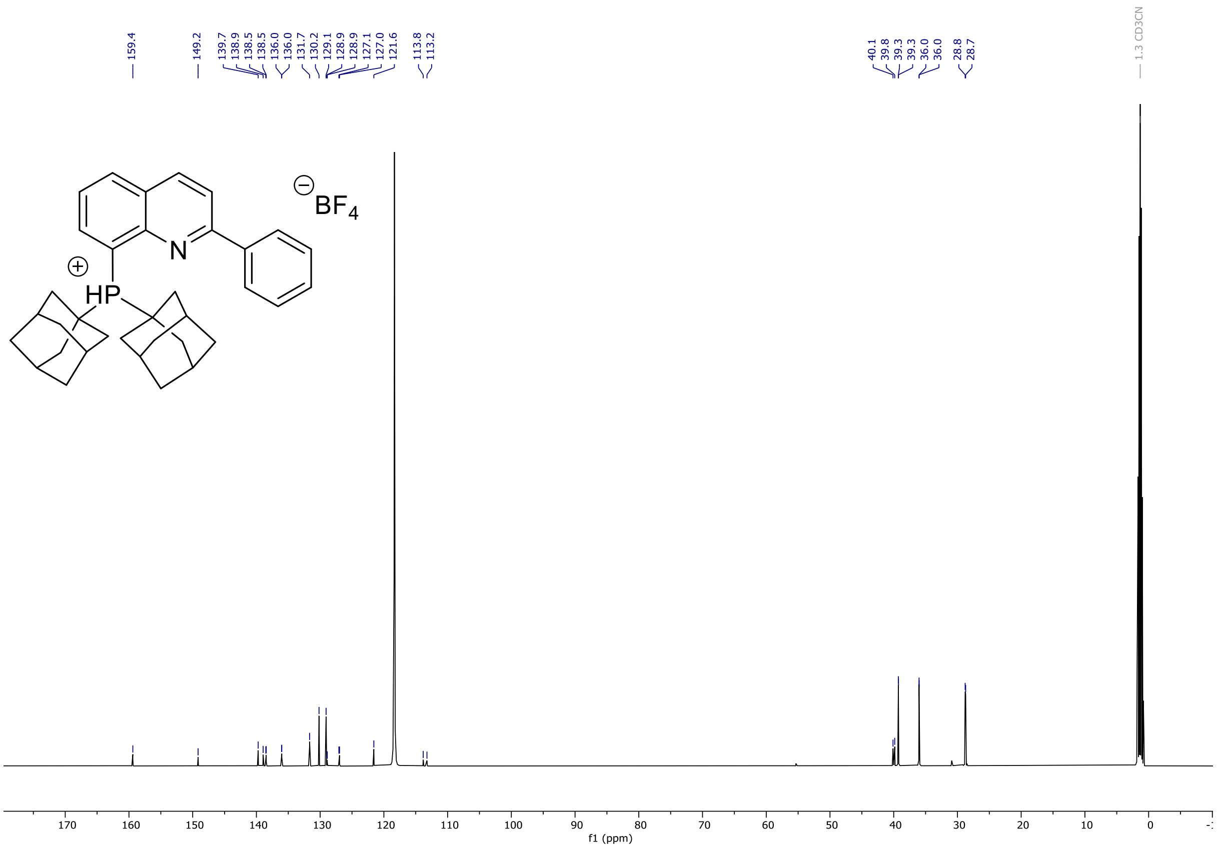

Figure S21. $^{13}C\{^{1}H\}$ NMR (125.81 MHz, CD_3CN, 298 K) of compound **I**.

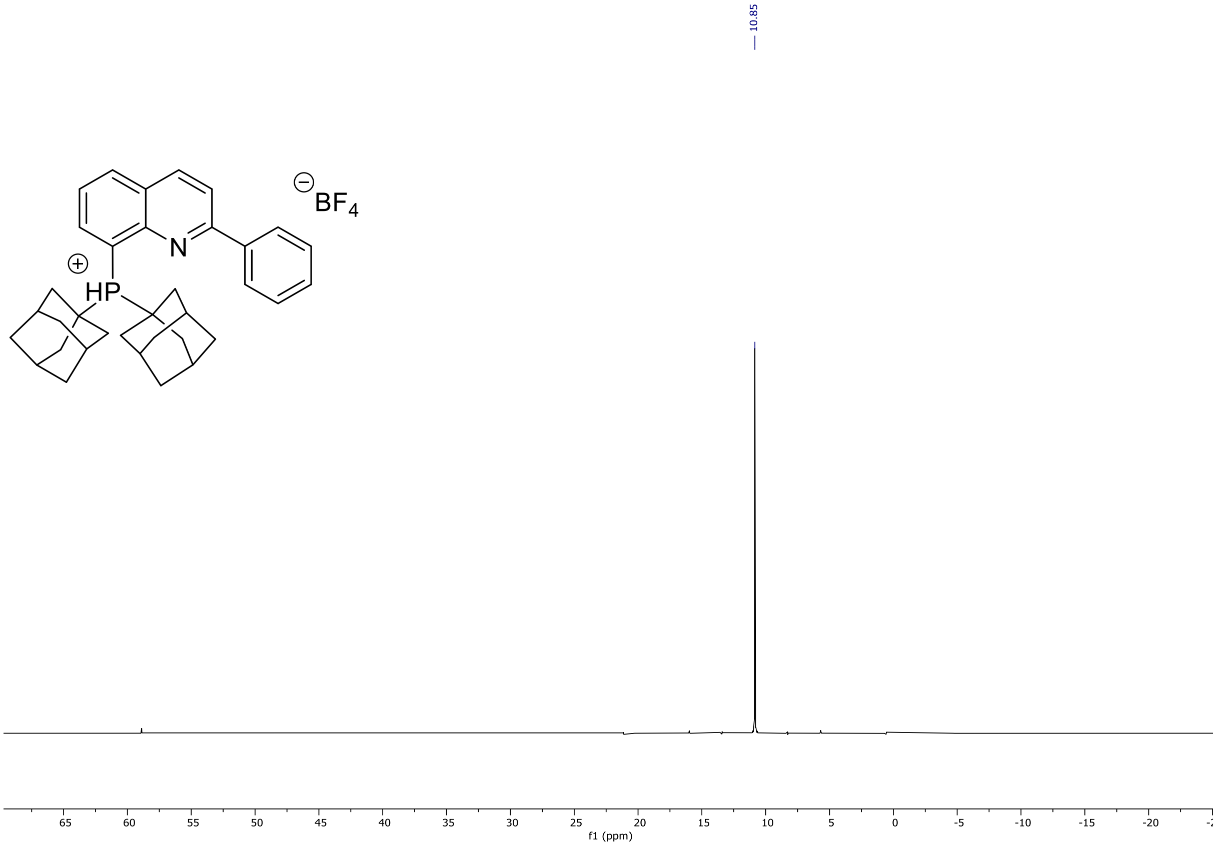

Figure S22. $^{31}P\{^{1}H\}$ NMR (202.52 MHz, CD_3CN, 298 K) of compound **I**.

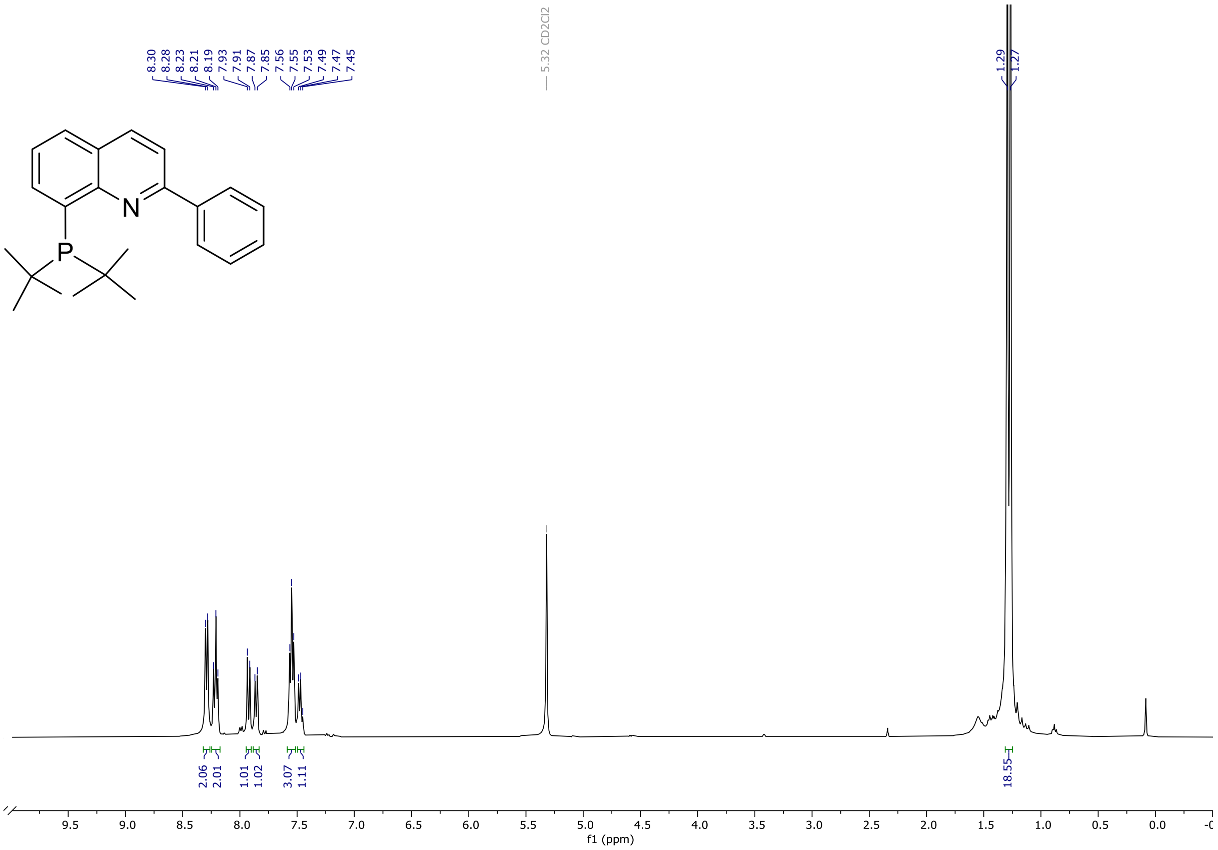

Figure S23. ^{1}H NMR (400.13 MHz, CD_2Cl_2, 298 K) of compound **II**.

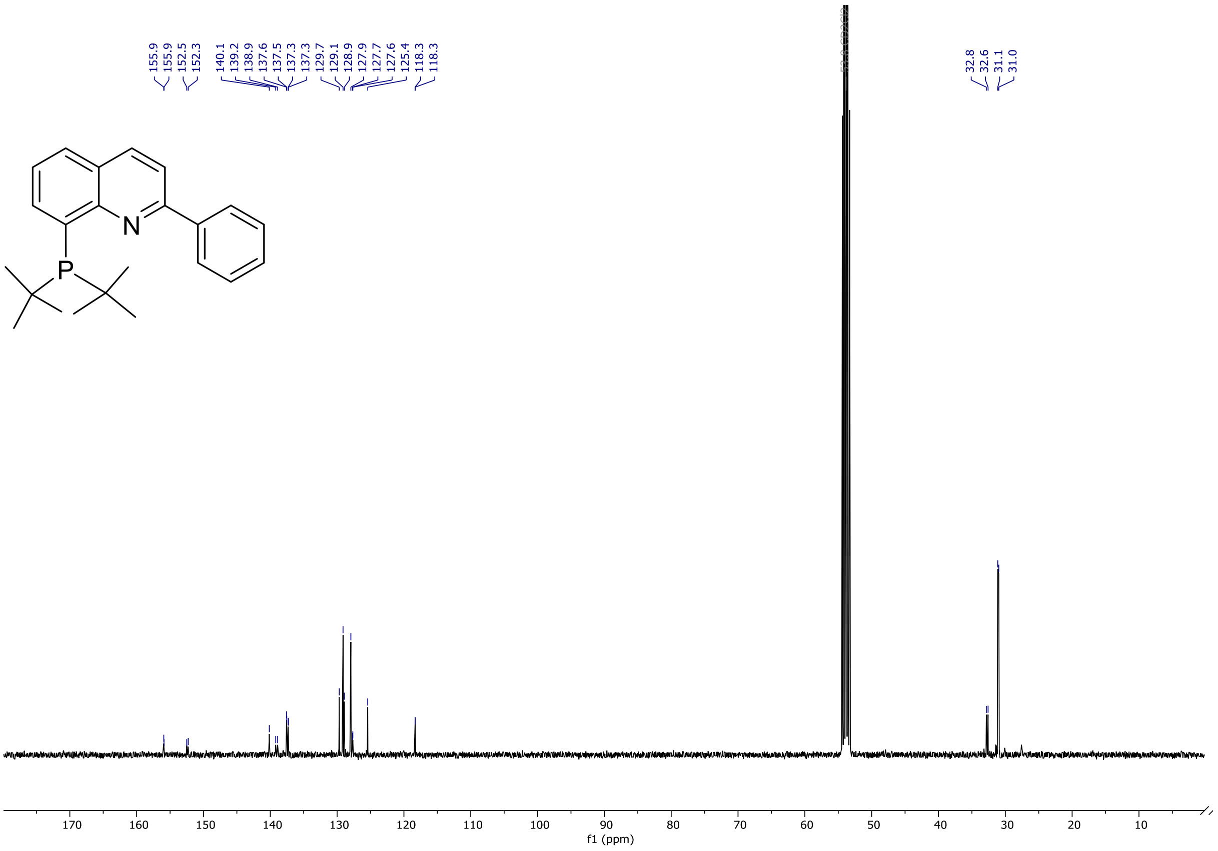

Figure S24. $^{13}C\{^{1}H\}$ NMR (100.65 MHz, CD_2Cl_2, 298 K) of compound **II**

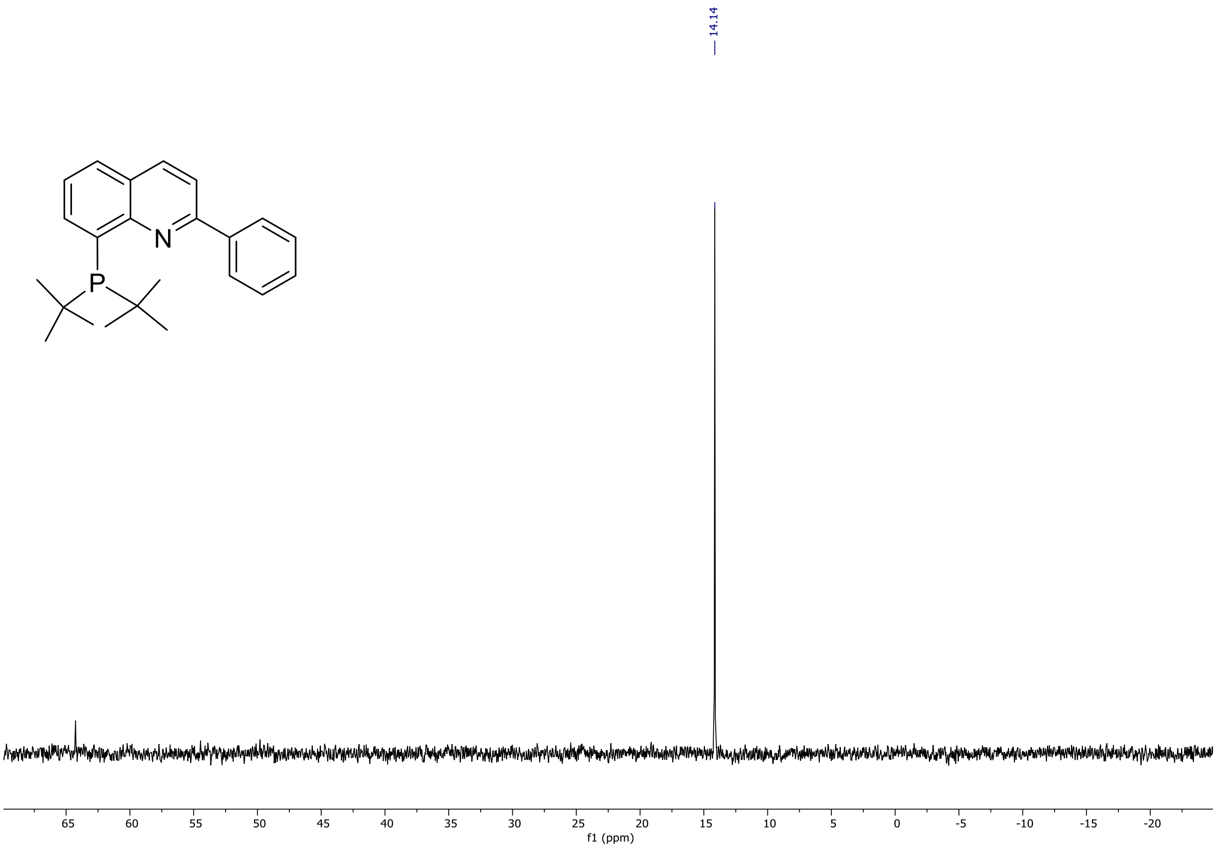

Figure S25. $^{31}P\{^{1}H\}$ NMR (161.99 MHz, CD_2Cl_2, 298 K) of compound **II**.

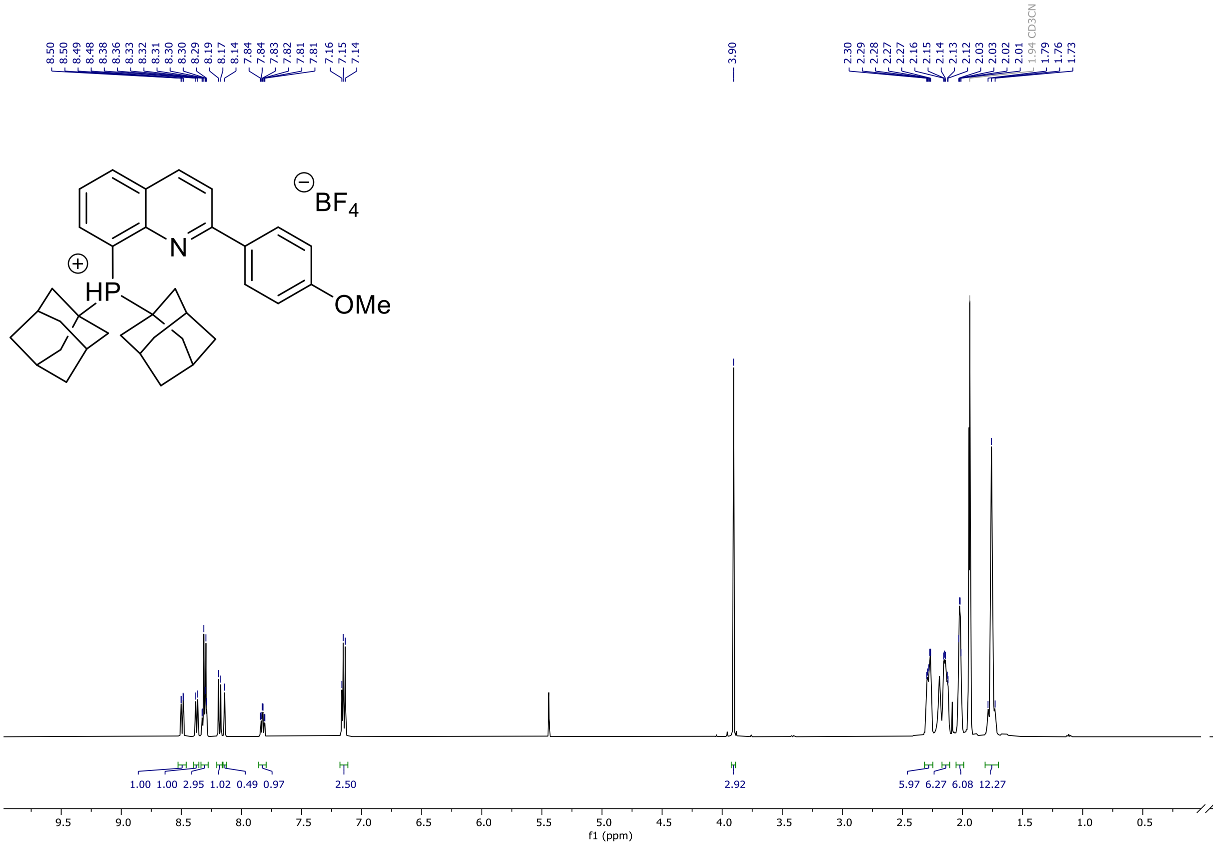

Figure S26. ^{1}H NMR (500.30 MHz, CD_3CN, 298 K) of compound **III**.

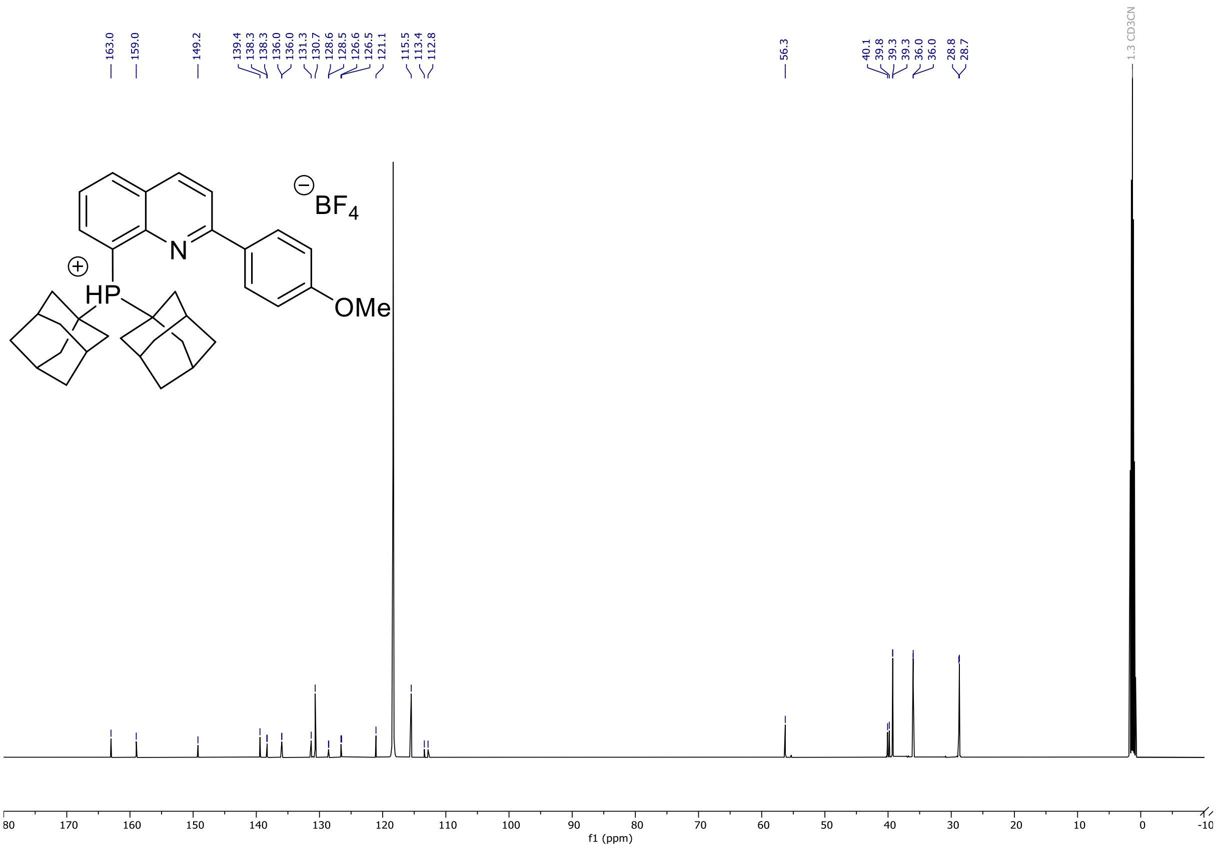

Figure S27. $^{13}C\{^1H\}$ NMR (125.81 MHz, CD_3CN, 298 K) of compound **III**.

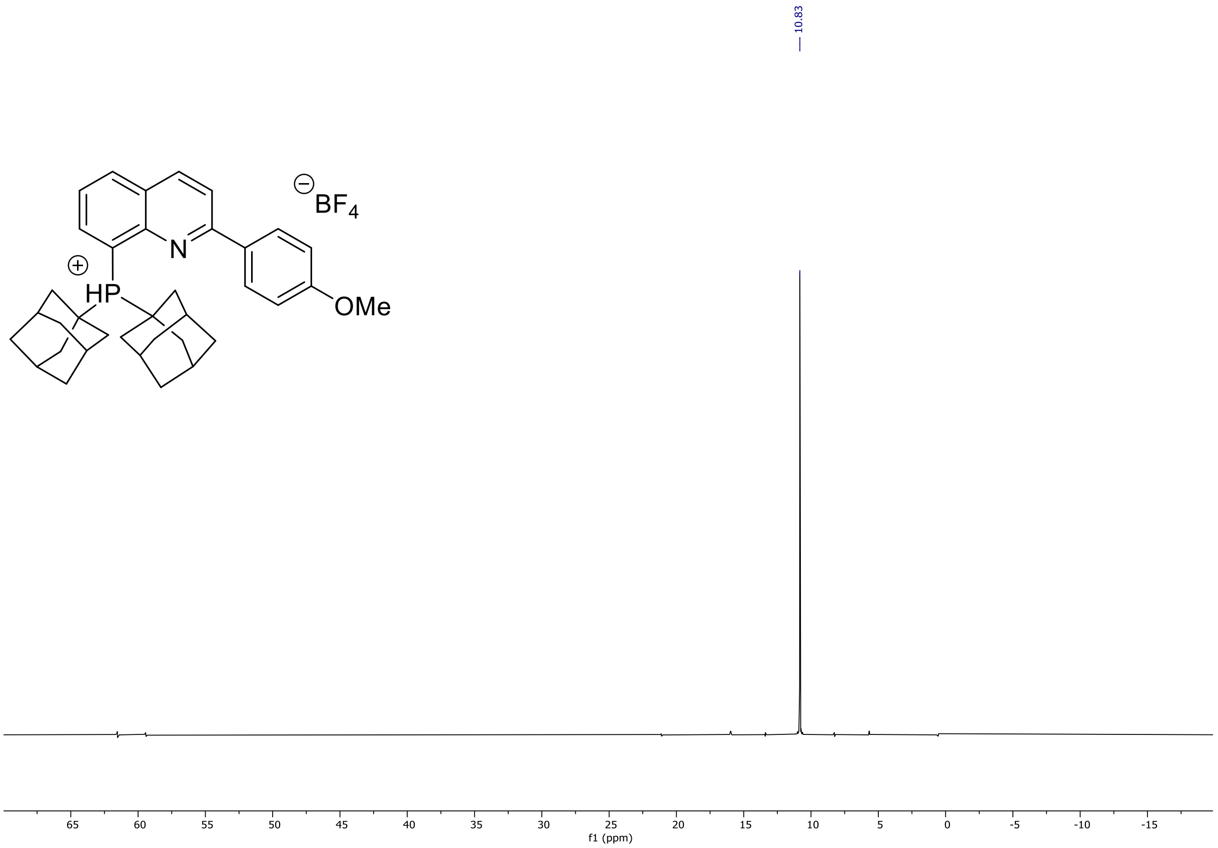

Figure S28. $^{31}P\{^1H\}$ NMR (202.52 MHz, CD_3CN, 298 K) of compound **III**.

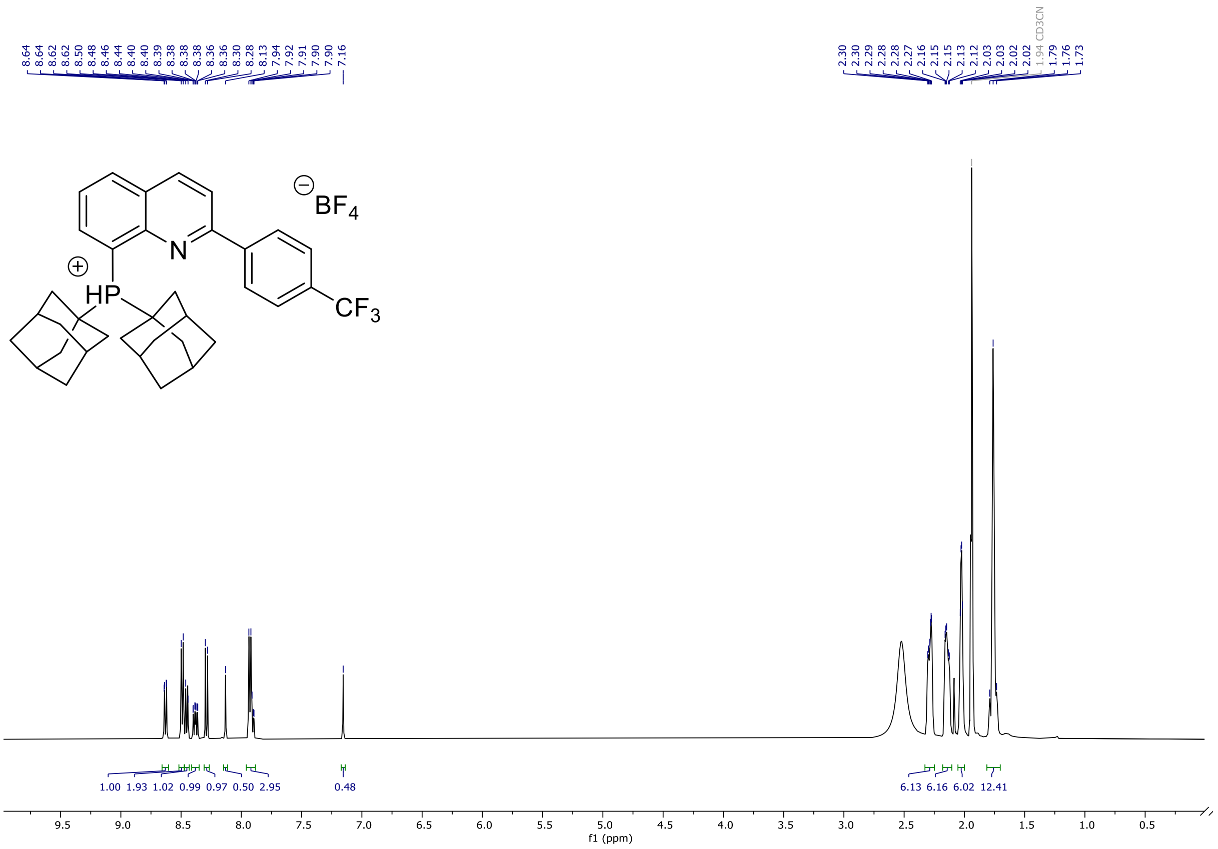

Figure S29. ^{1}H NMR (500.30 MHz, CD_3CN, 298 K) of compound **IV**.

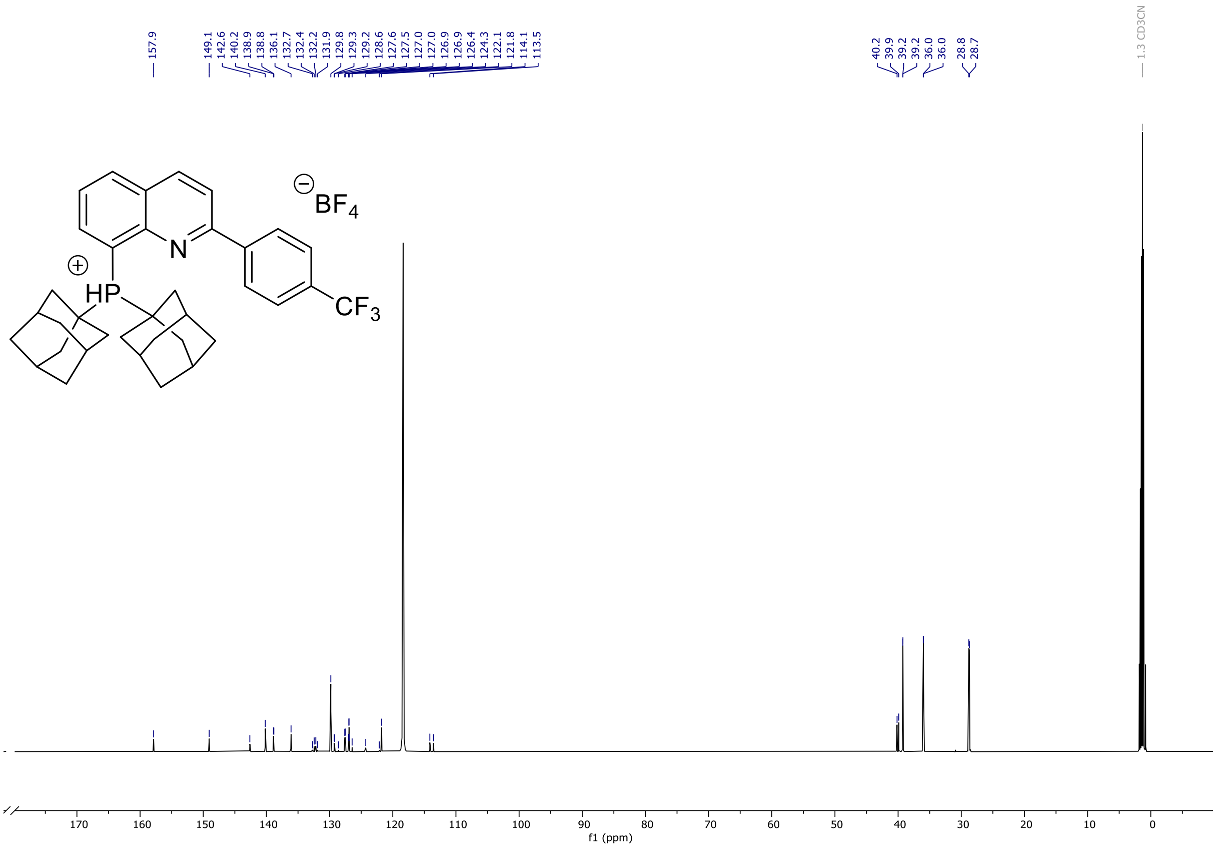

Figure S30. $^{13}C\{^{1}H\}$ NMR (125.81 MHz, CD_3CN, 298 K) of compound **IV**.

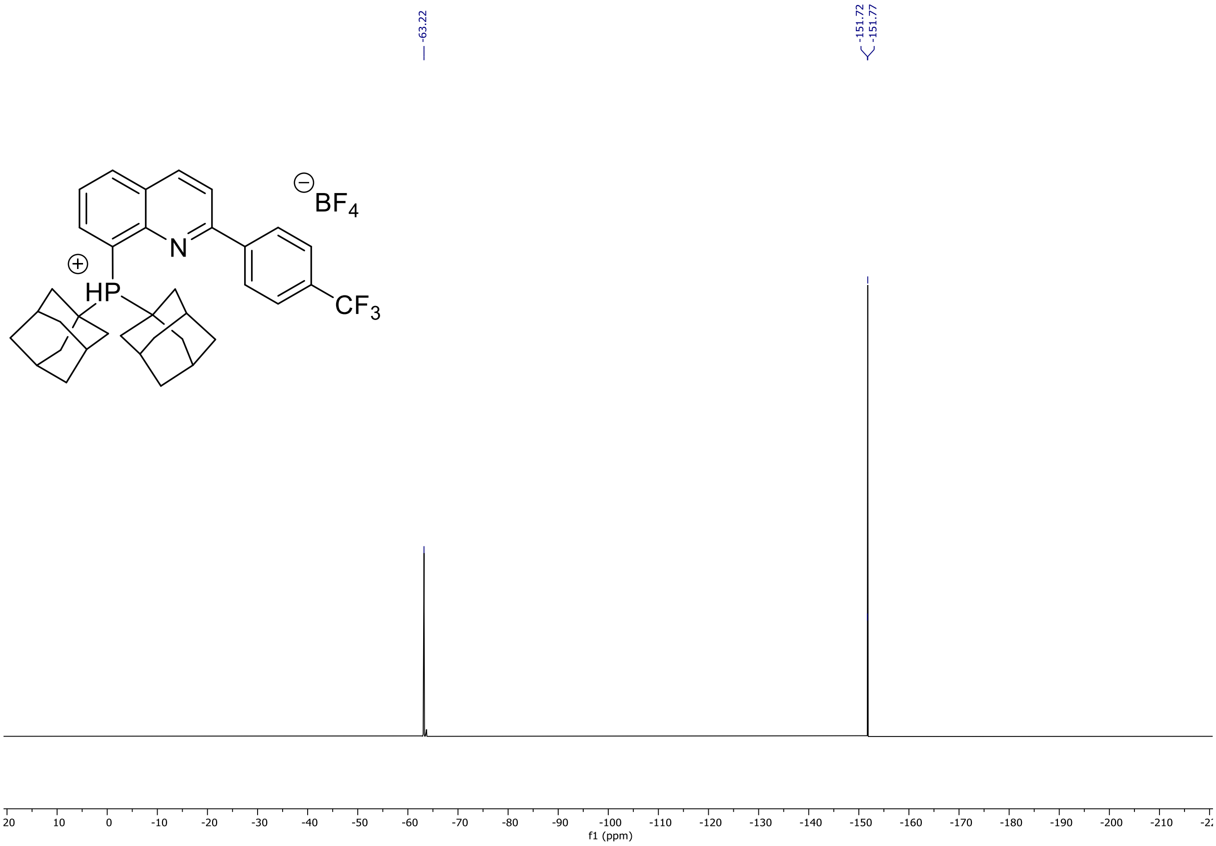

Figure S31. ^{19}F NMR (470.71 MHz, CD_3CN, 298 K) of compound **IV**.

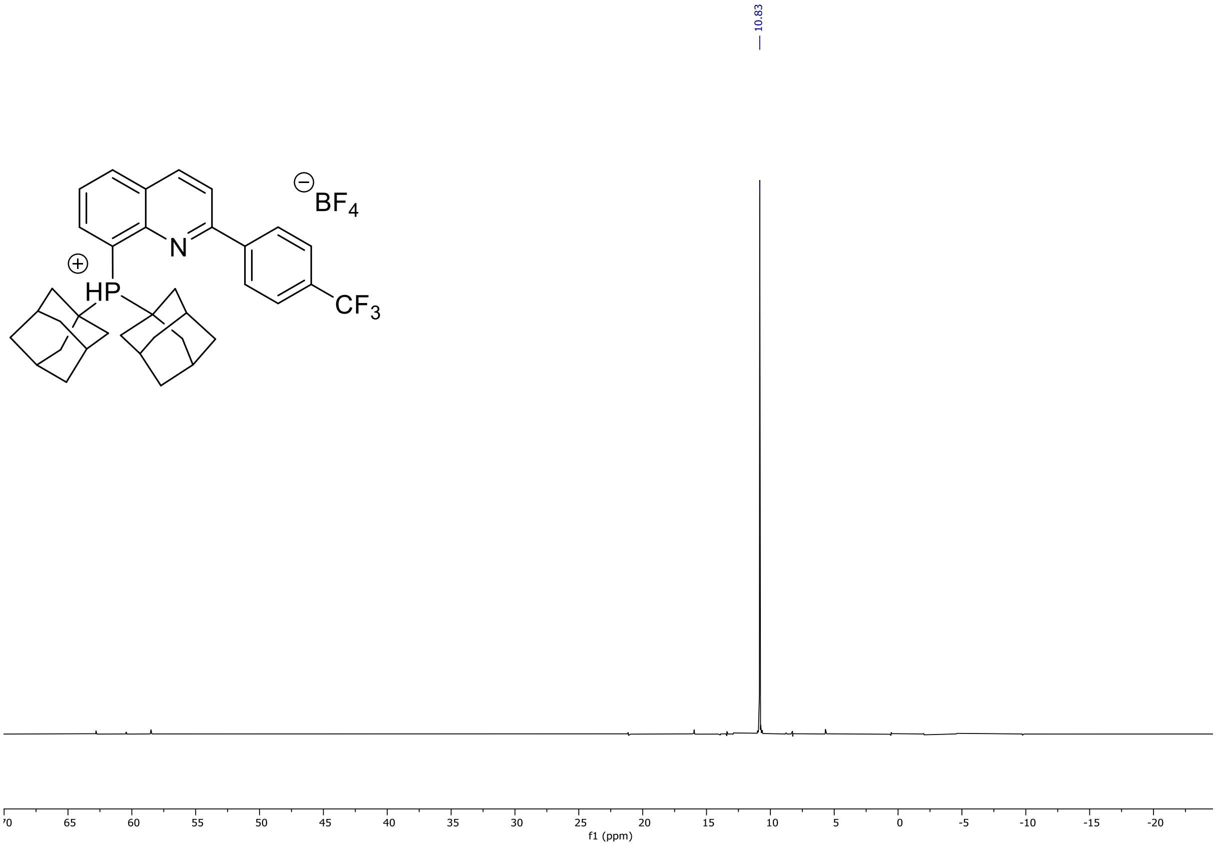

Figure S32. $^{31}P\{^{1}H\}$ NMR (202.52 MHz, CD_3CN, 298 K) of compound **IV**.

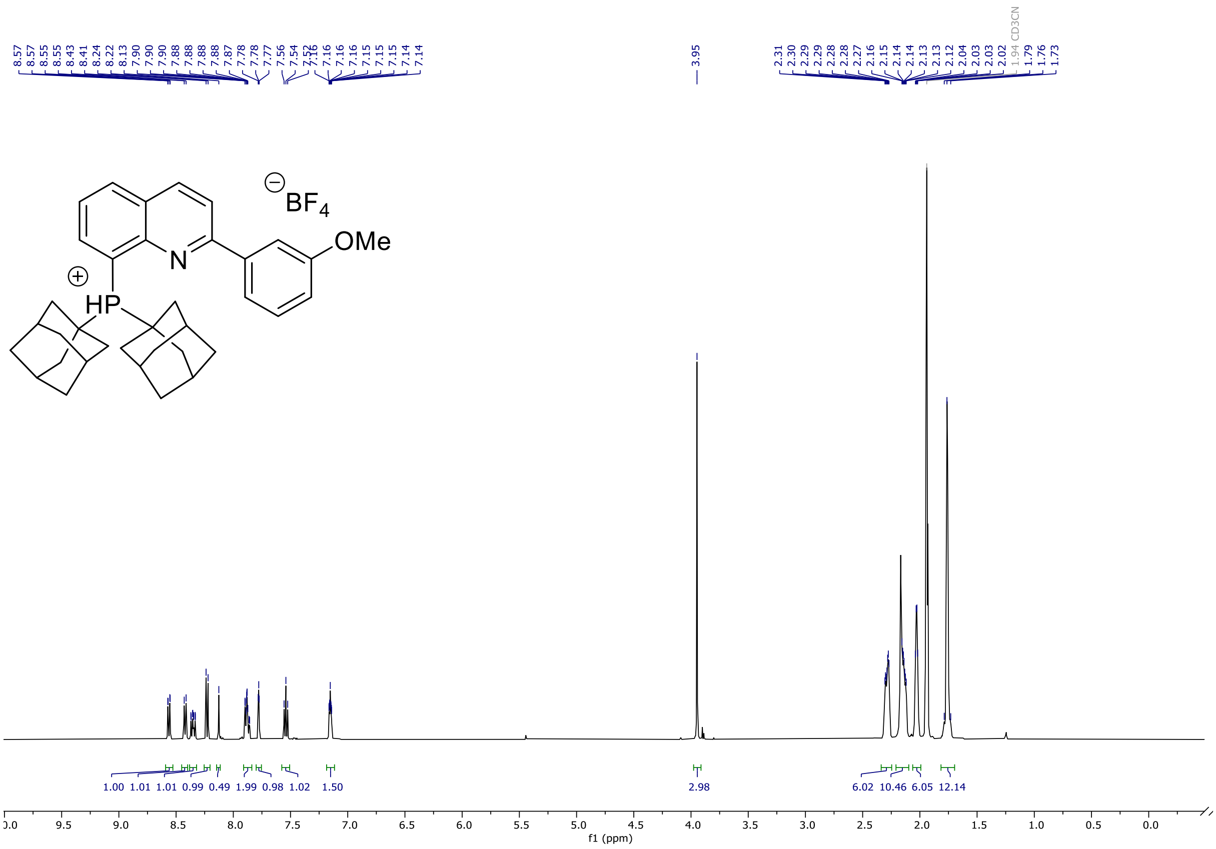

Figure S33. ^{1}H NMR (500.30 MHz, CD_3CN, 298 K) of compound **V**.

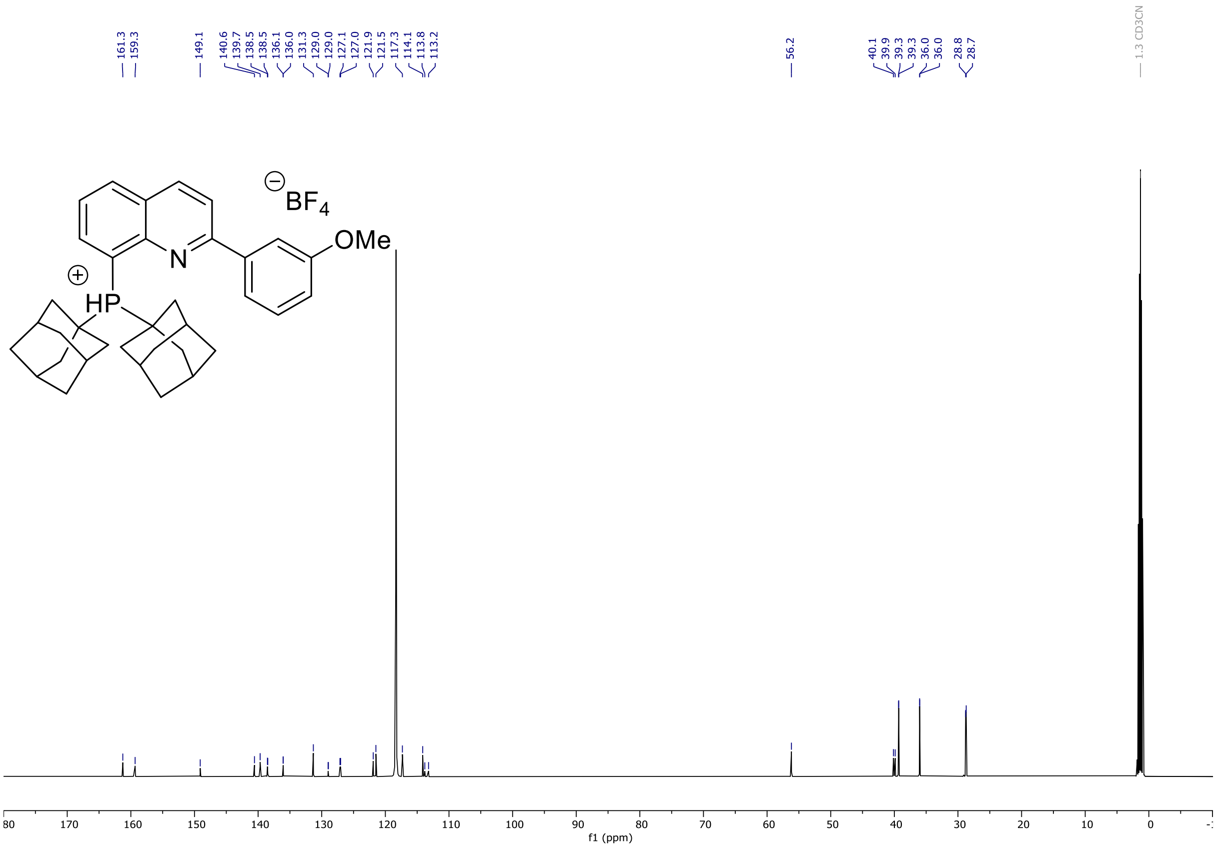

Figure S34. $^{13}C\{^{1}H\}$ NMR (125.81 MHz, CD_3CN, 298 K) of compound **V**.

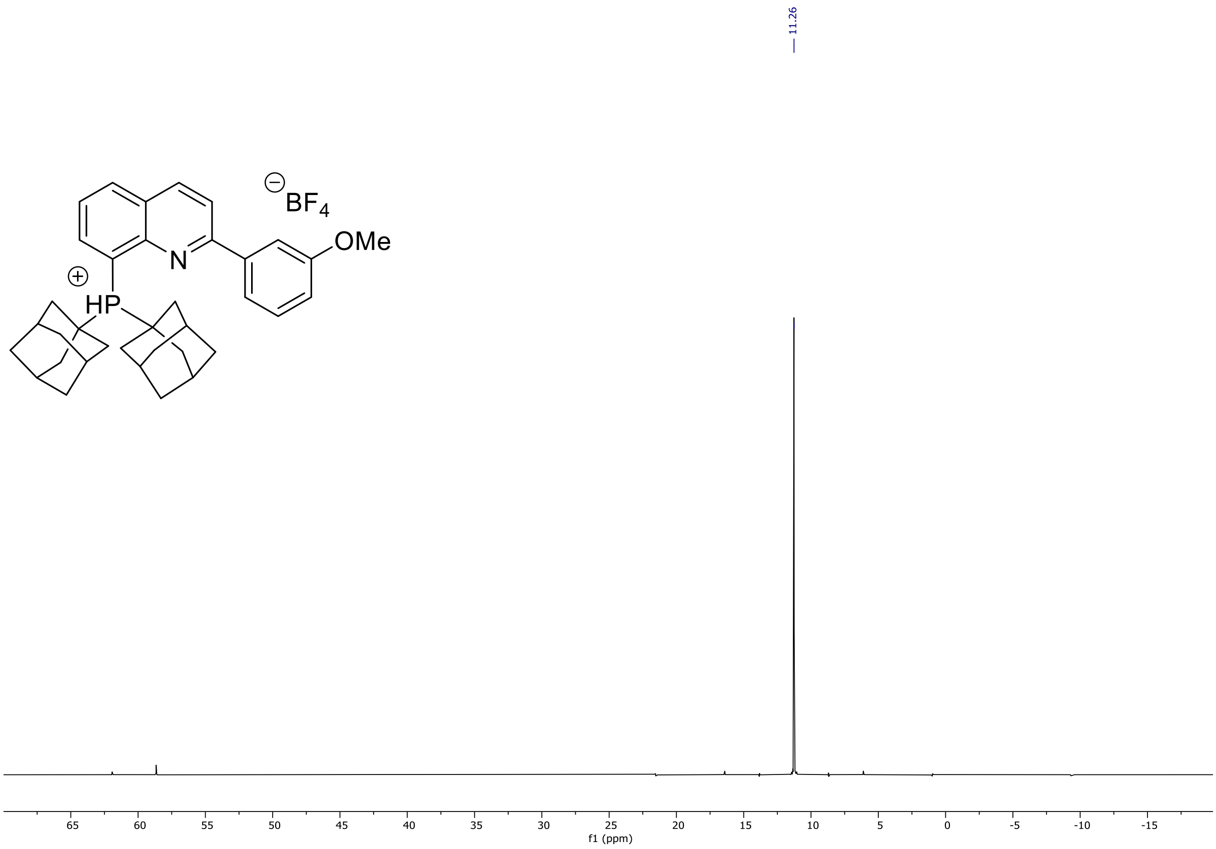

Figure S35. $^{31}P\{^{1}H\}$ NMR (202.52 MHz, CD_3CN, 298 K) of compound **V**.

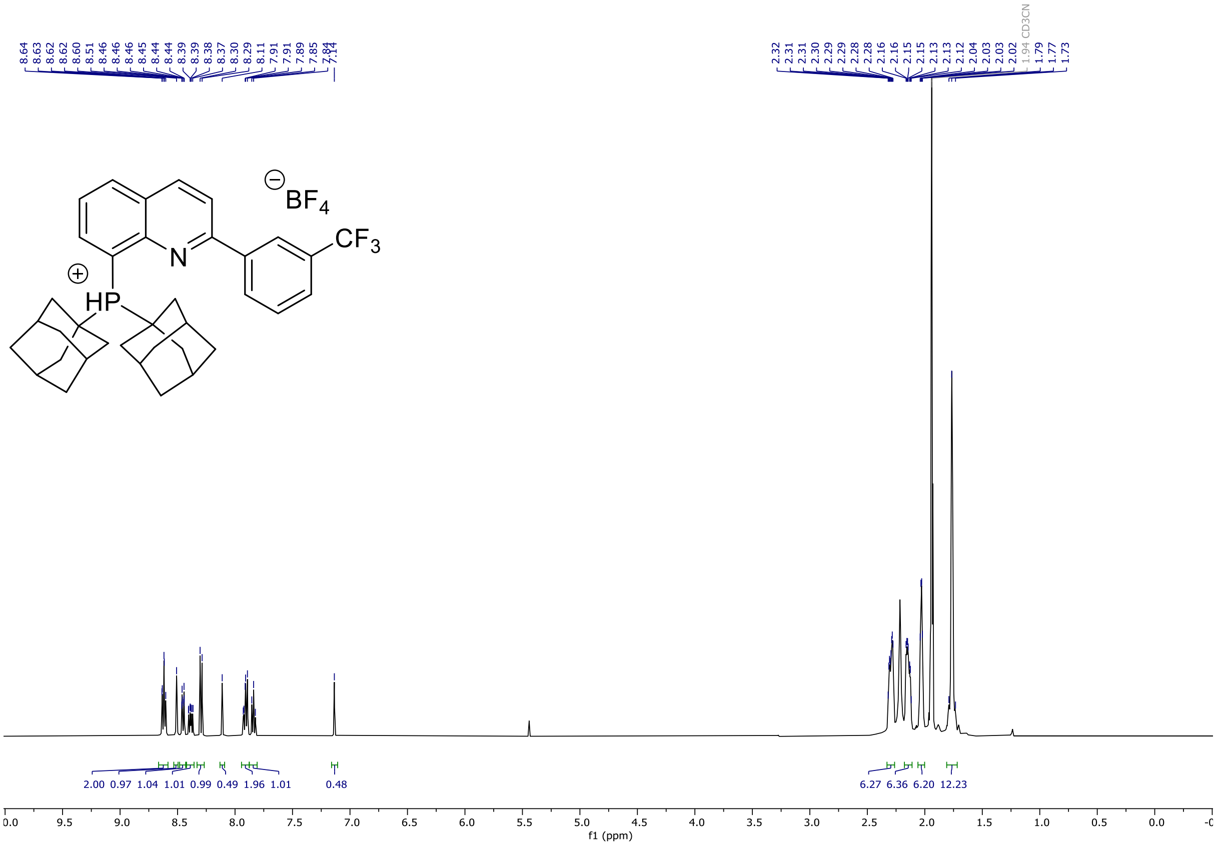

Figure S36. ^{1}H NMR (500.30 MHz, CD_3CN, 298 K) of compound **VI**.

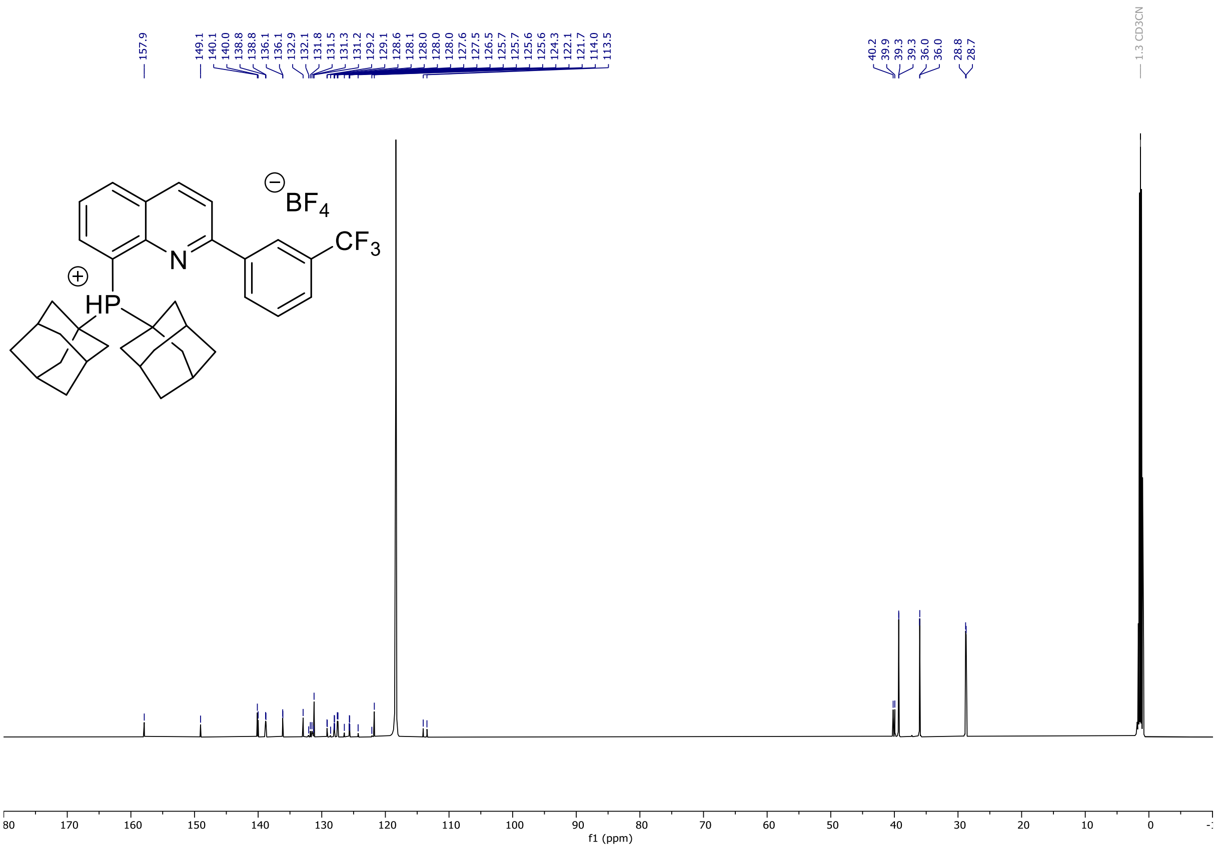

Figure S37. $^{13}C\{^1H\}$ NMR (125.81 MHz, CD_3CN, 298 K) of compound **VI**.

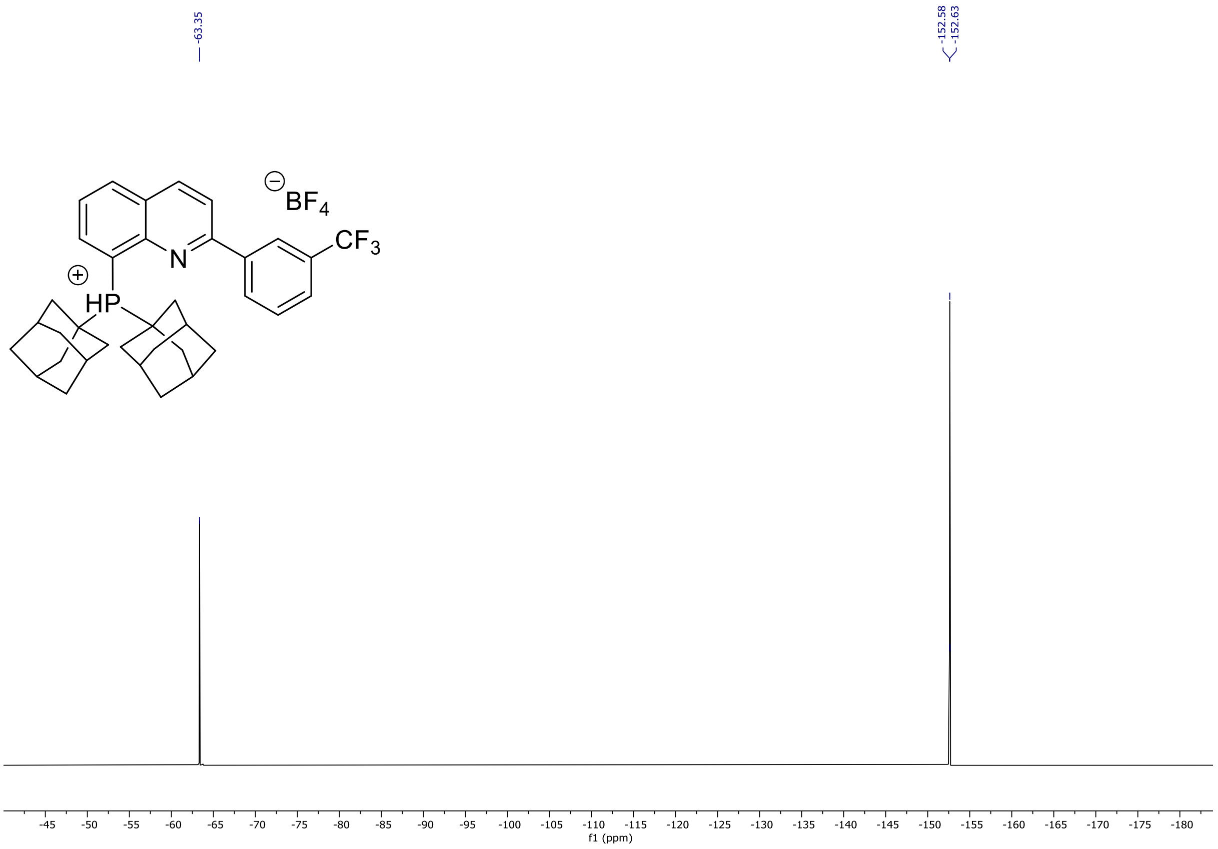

Figure S38. ^{19}F NMR (470.71 MHz, CD_3CN, 298 K) of compound **VI**.

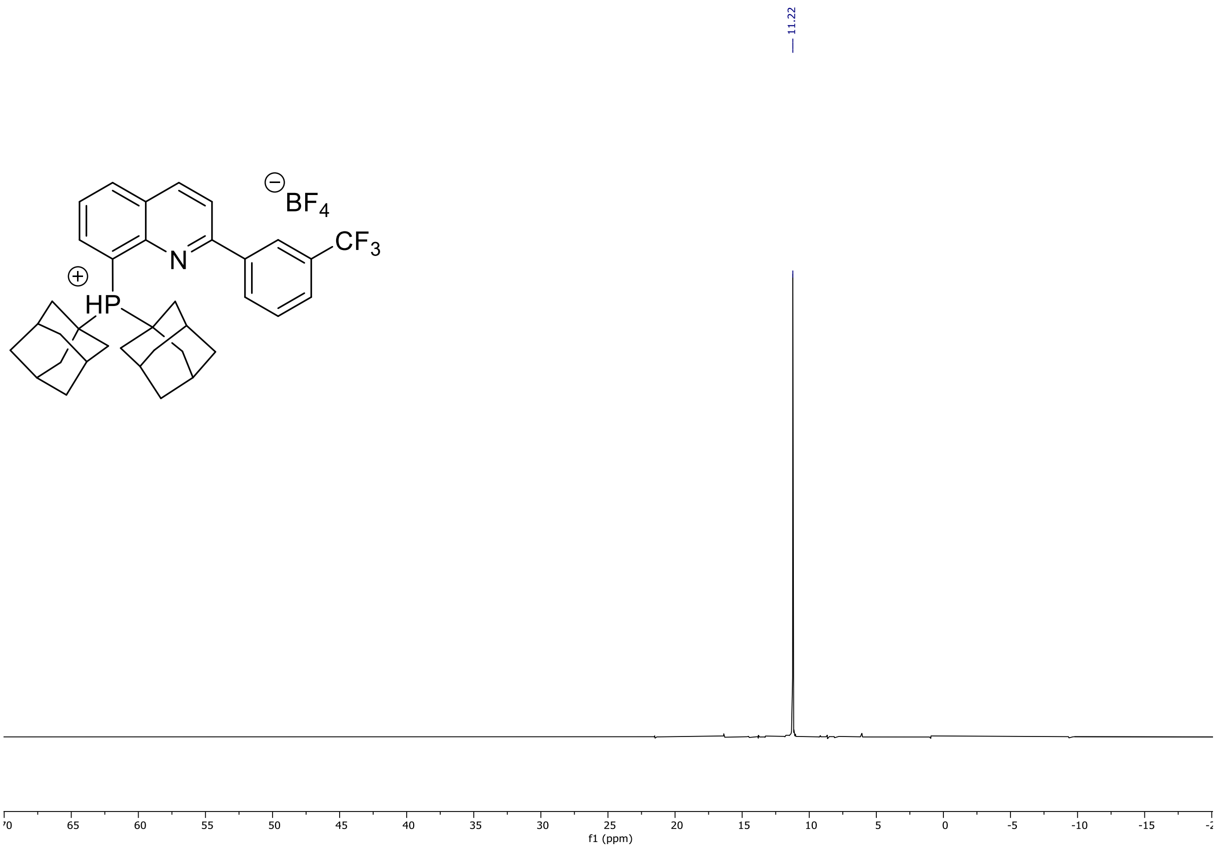

Figure S39. $^{31}P\{^{1}H\}$ NMR (202.52 MHz, CD_3CN, 298 K) of compound **VI**.

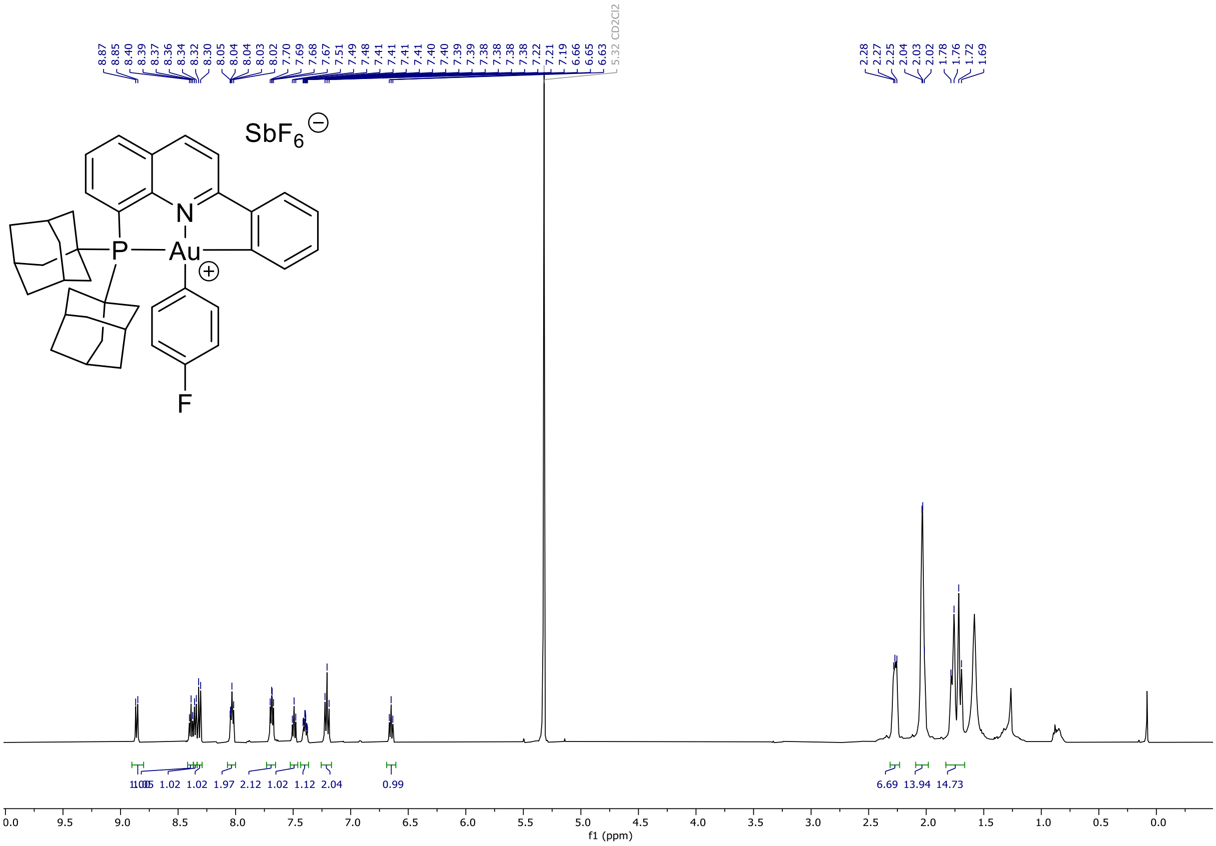

Figure S40. ^{1}H NMR (500.30 MHz, CD_2Cl_2, 298 K) of compound **1a**.

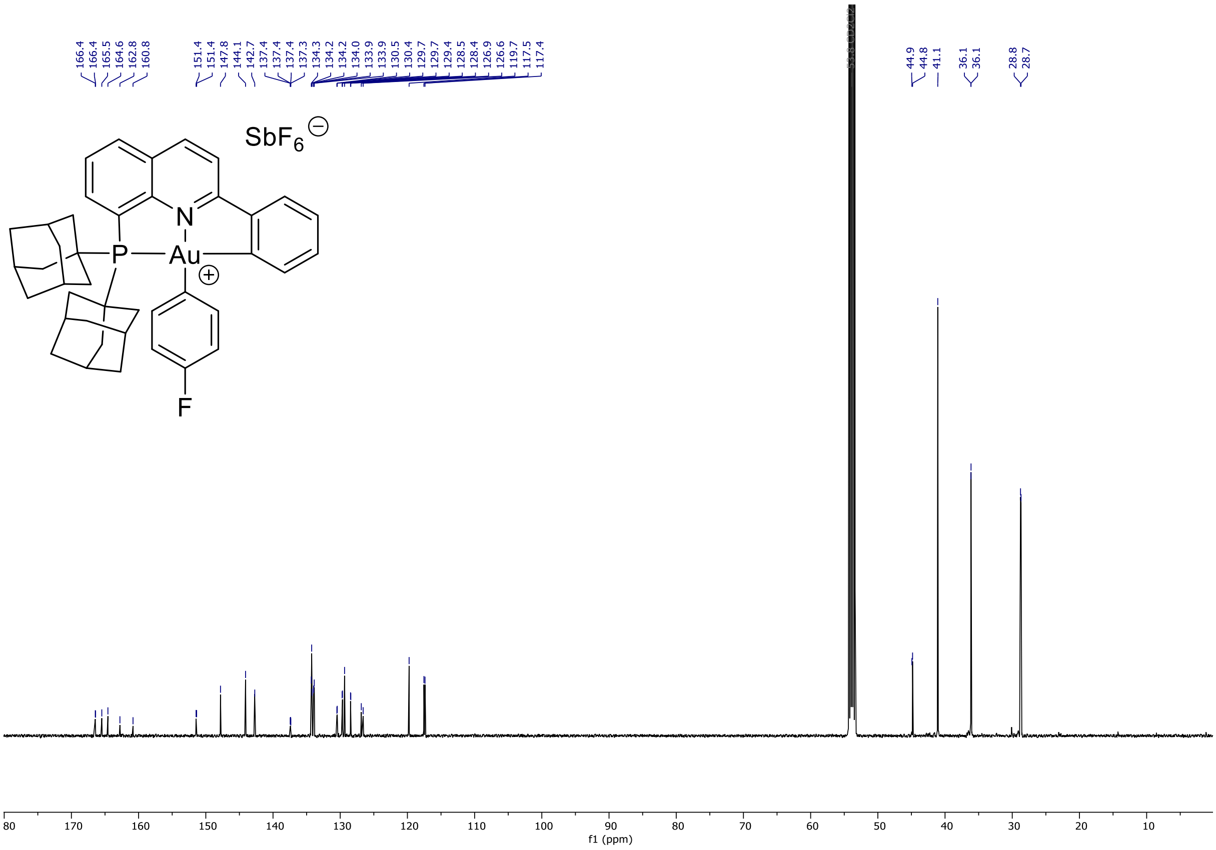

Figure S41. $^{13}C\{^{1}H\}$ NMR (125.81 MHz, CD_2Cl_2, 298 K) of compound **1a**.

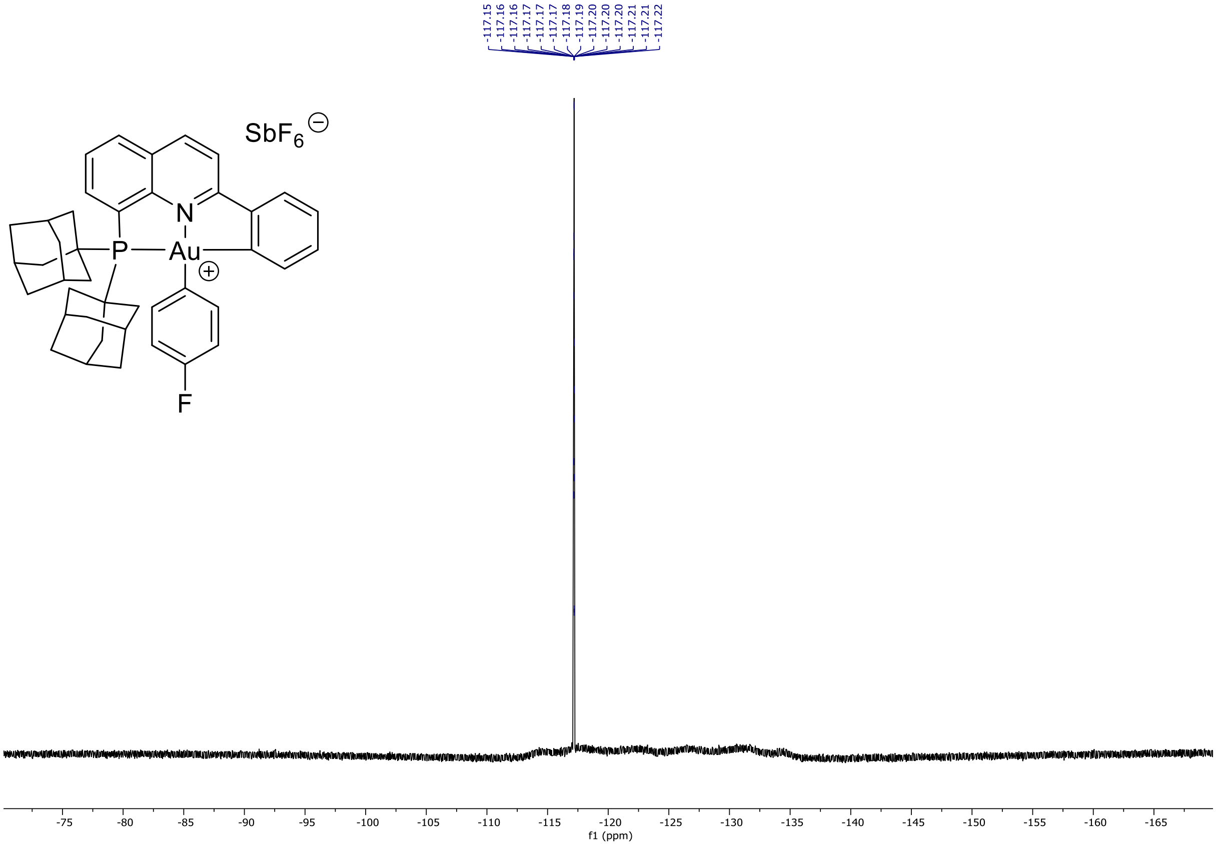

Figure S42. ^{19}F NMR (470.71 MHz, CD_2Cl_2, 298 K) of compound **1a**.

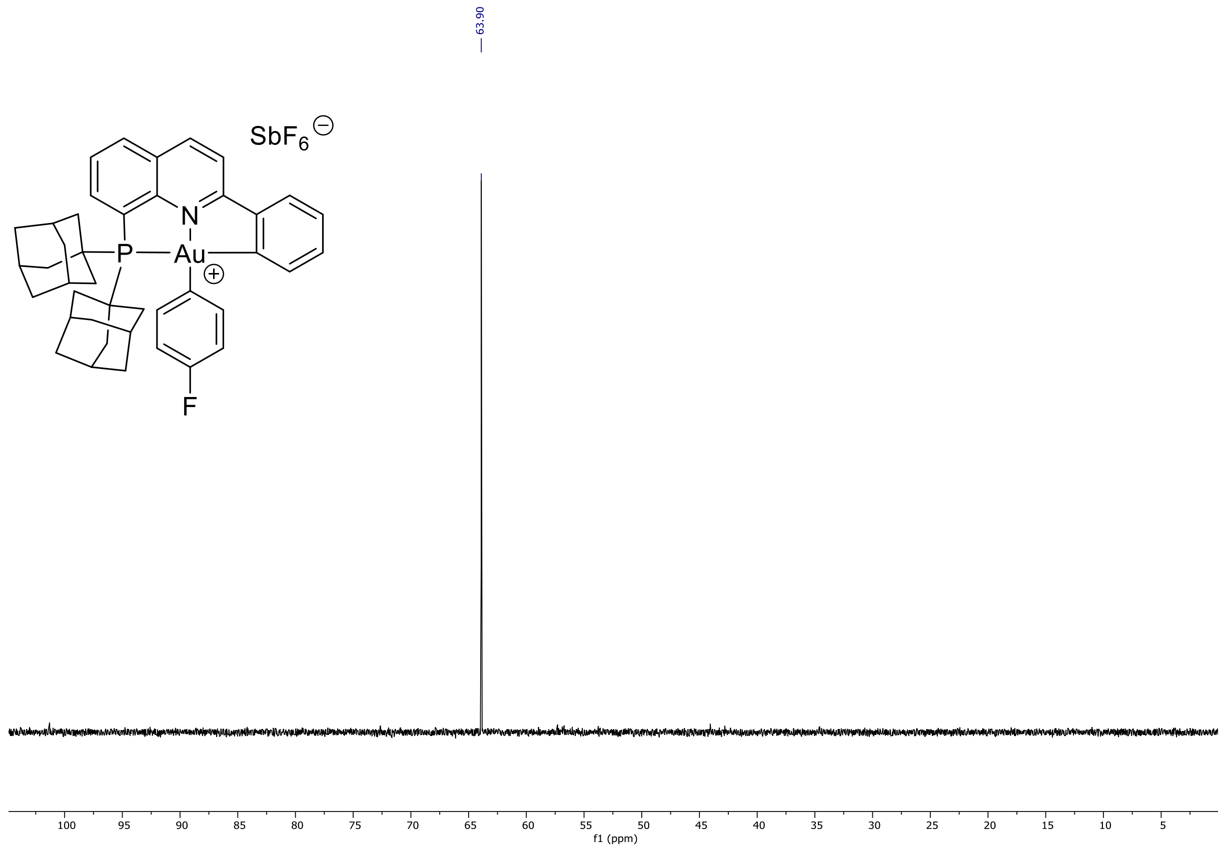

Figure S43. $^{31}P\{^{1}H\}$ NMR (202.52 MHz, CD_2Cl_2, 298 K) of compound **1a**.

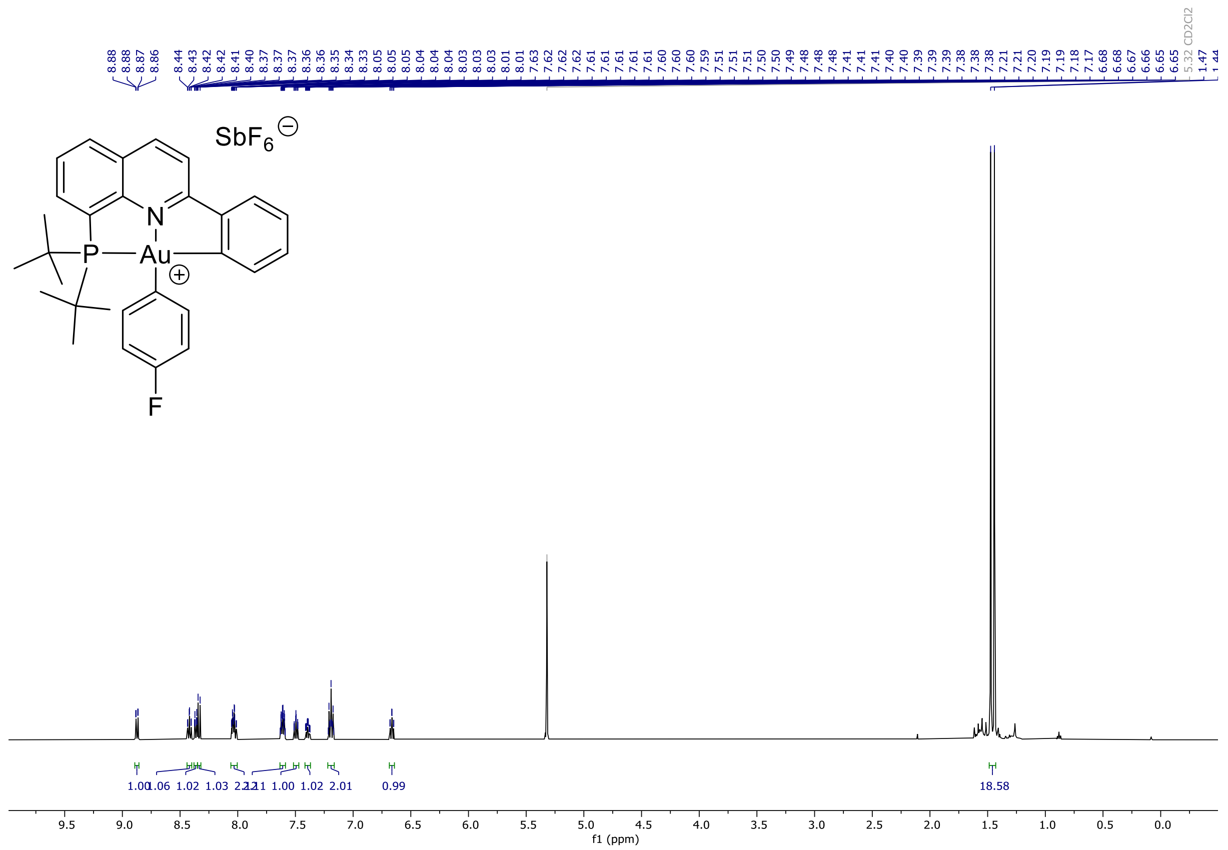

Figure S44. ^{1}H NMR (500.30 MHz, CD_2Cl_2, 298 K) of compound **1b**.

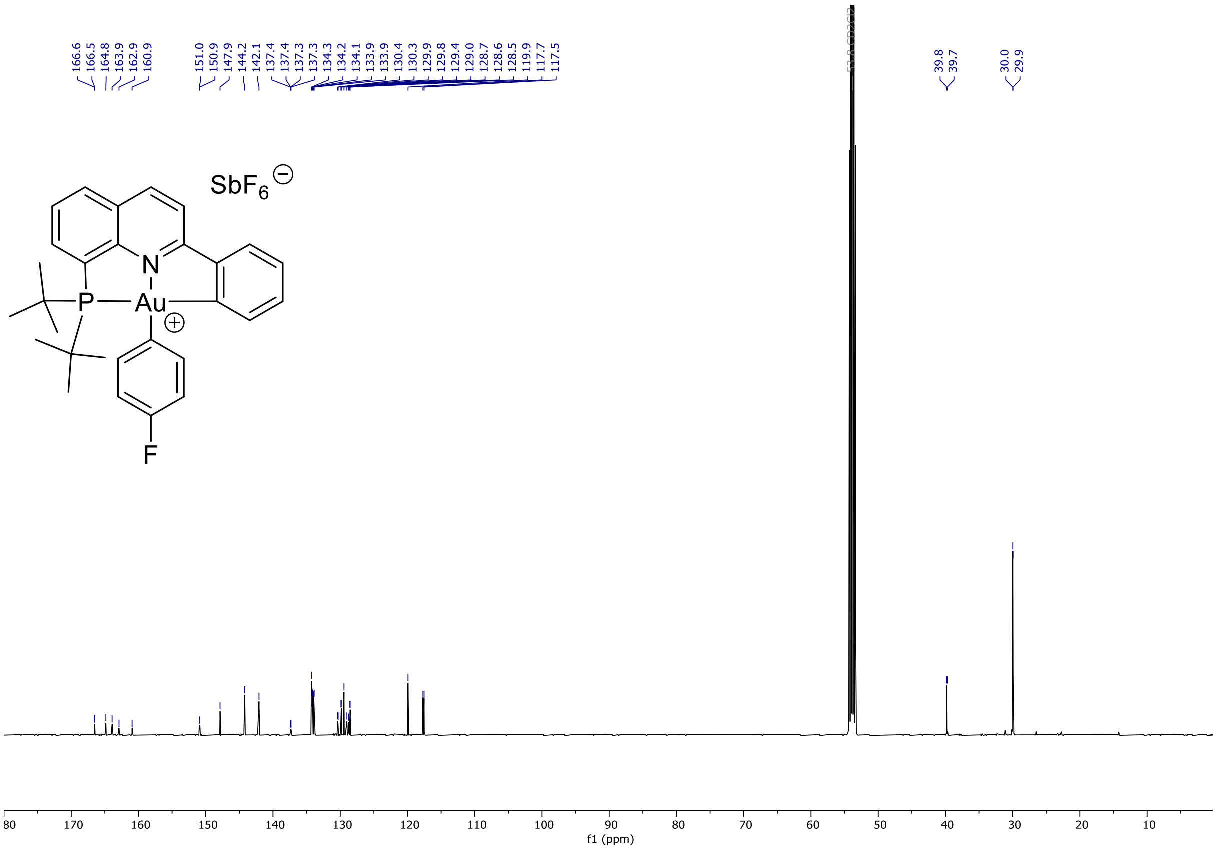

Figure S45. $^{13}C\{^1H\}$ NMR (125.81 MHz, CD_2Cl_2, 298 K) of compound **1b**.

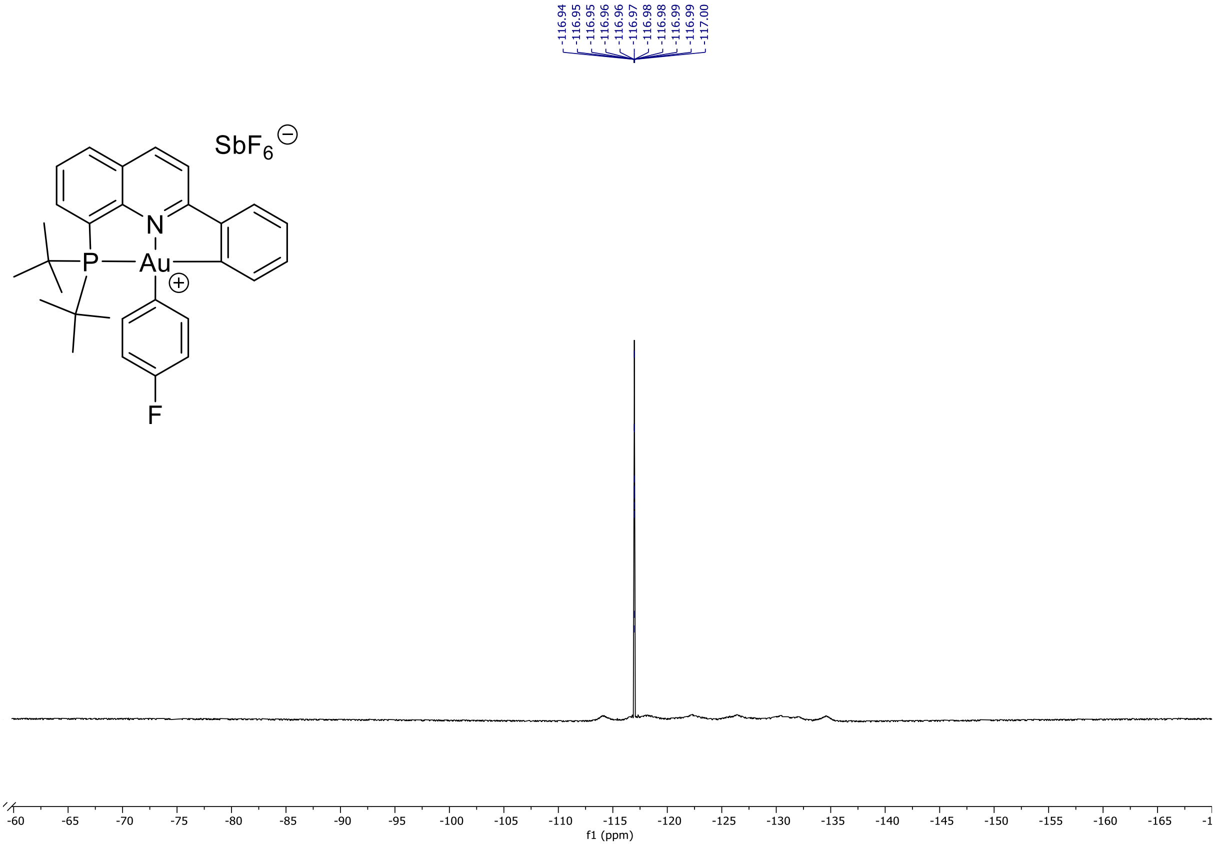

Figure S46. ^{19}F NMR (470.71 MHz, CD_2Cl_2, 298 K) of compound **1b**.

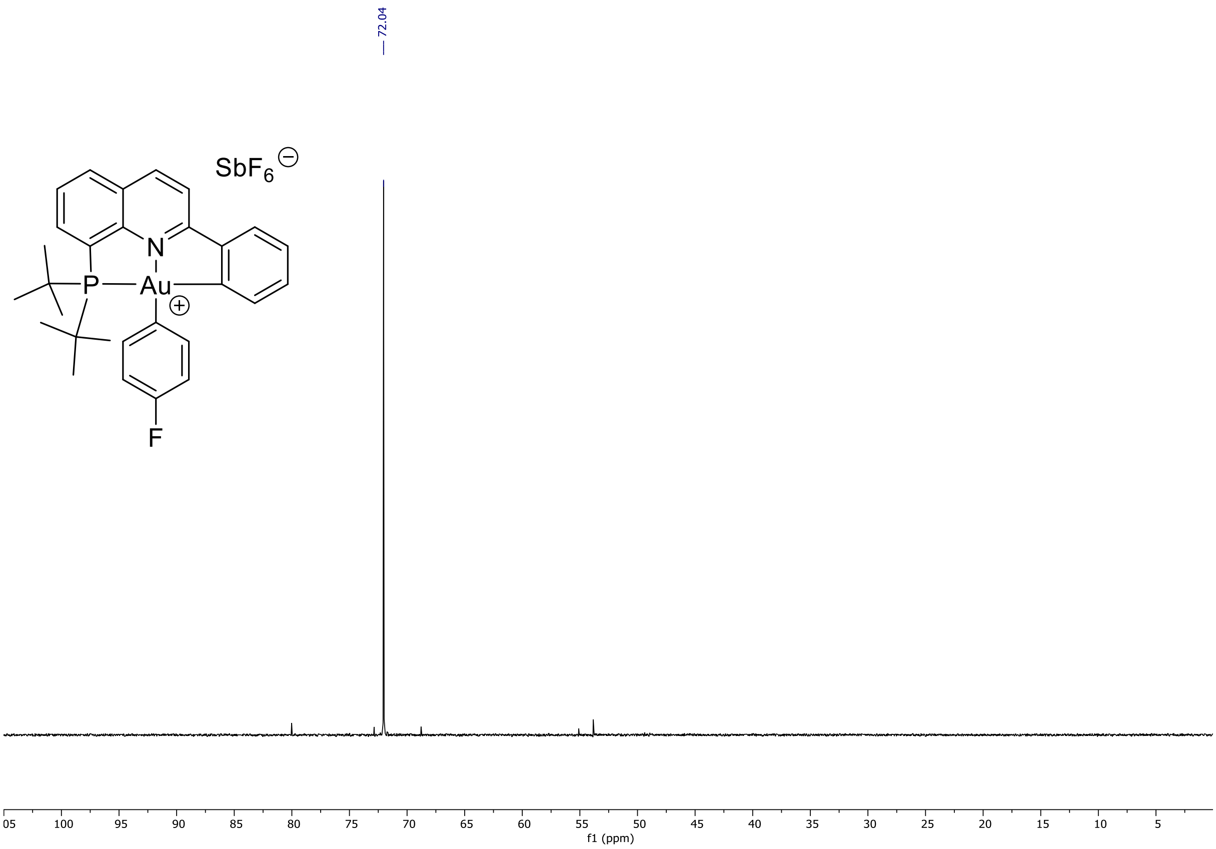

Figure S47. $^{31}P\{^{1}H\}$ NMR (202.52 MHz, CD_2Cl_2, 298 K) of compound **1b**.

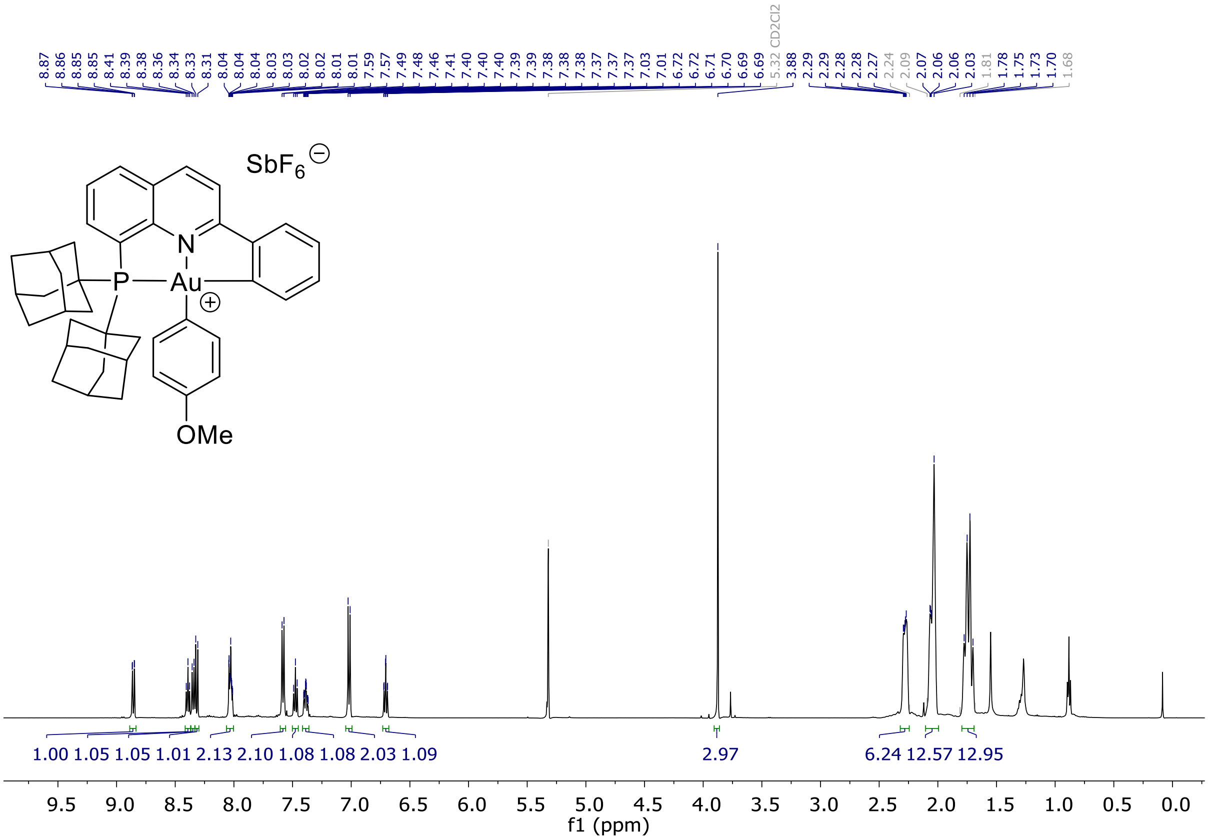

Figure S48. ^{1}H NMR (500.30 MHz, CD_2Cl_2, 298 K) of compound **1c**.

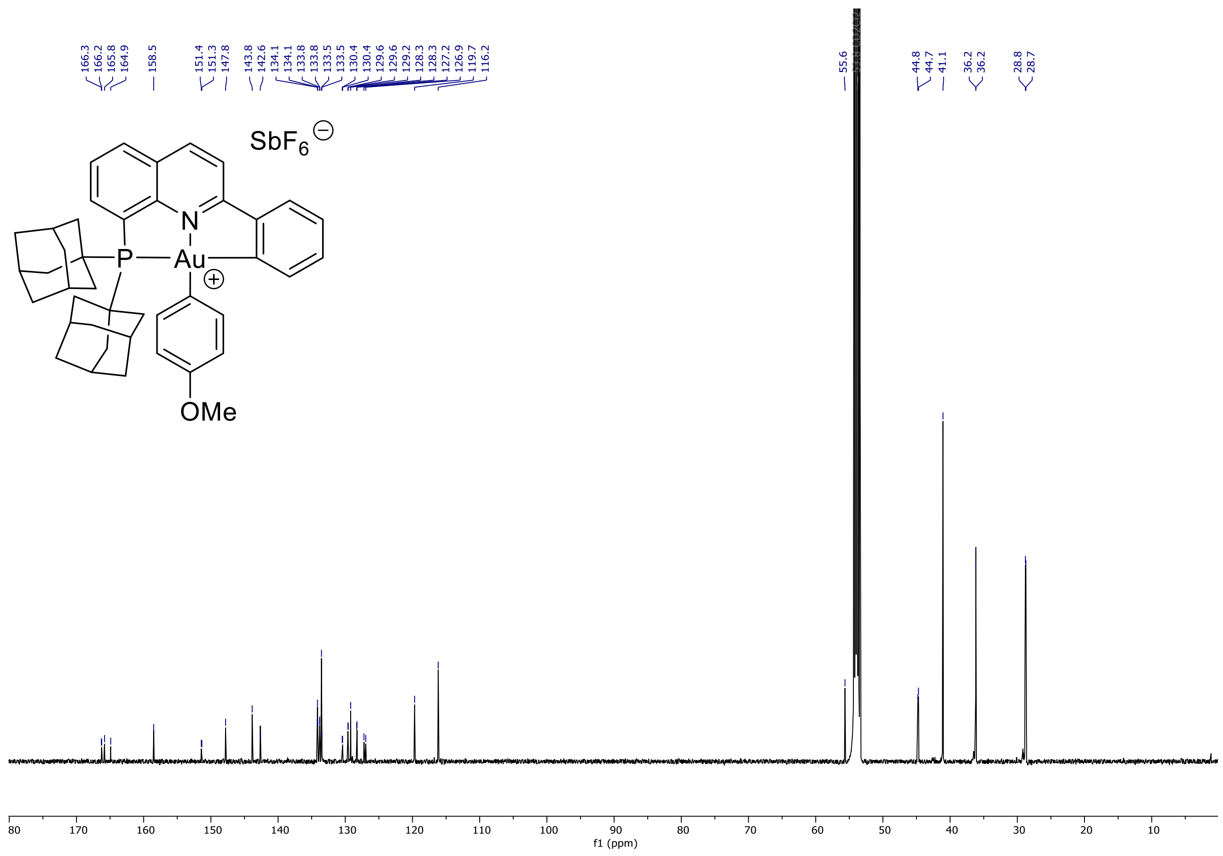

Figure S49. $^{13}C\{^1H\}$ NMR (125.81 MHz, CD_2Cl_2, 298 K) of compound **1c**.

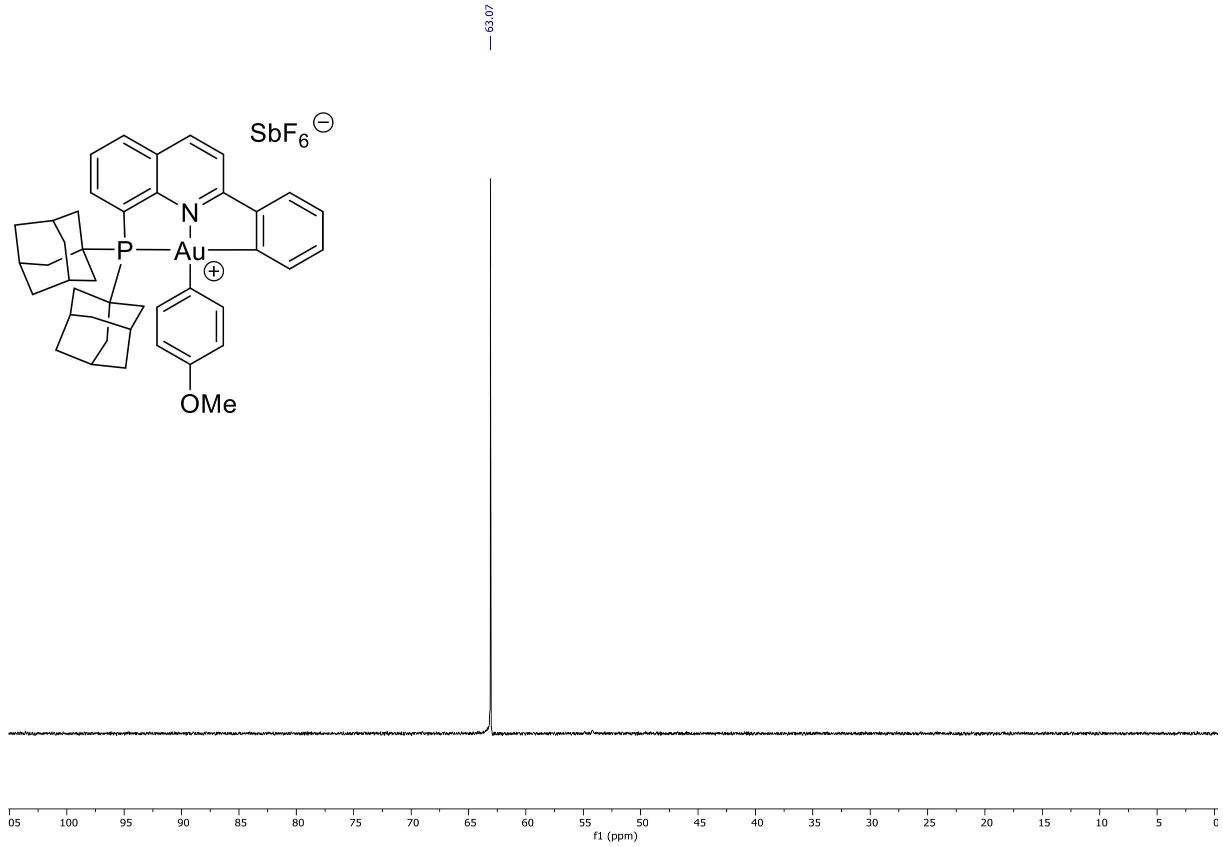

Figure S50. $^{31}P\{^1H\}$ NMR (202.52 MHz, CD_2Cl_2, 298 K) of compound **1c**.

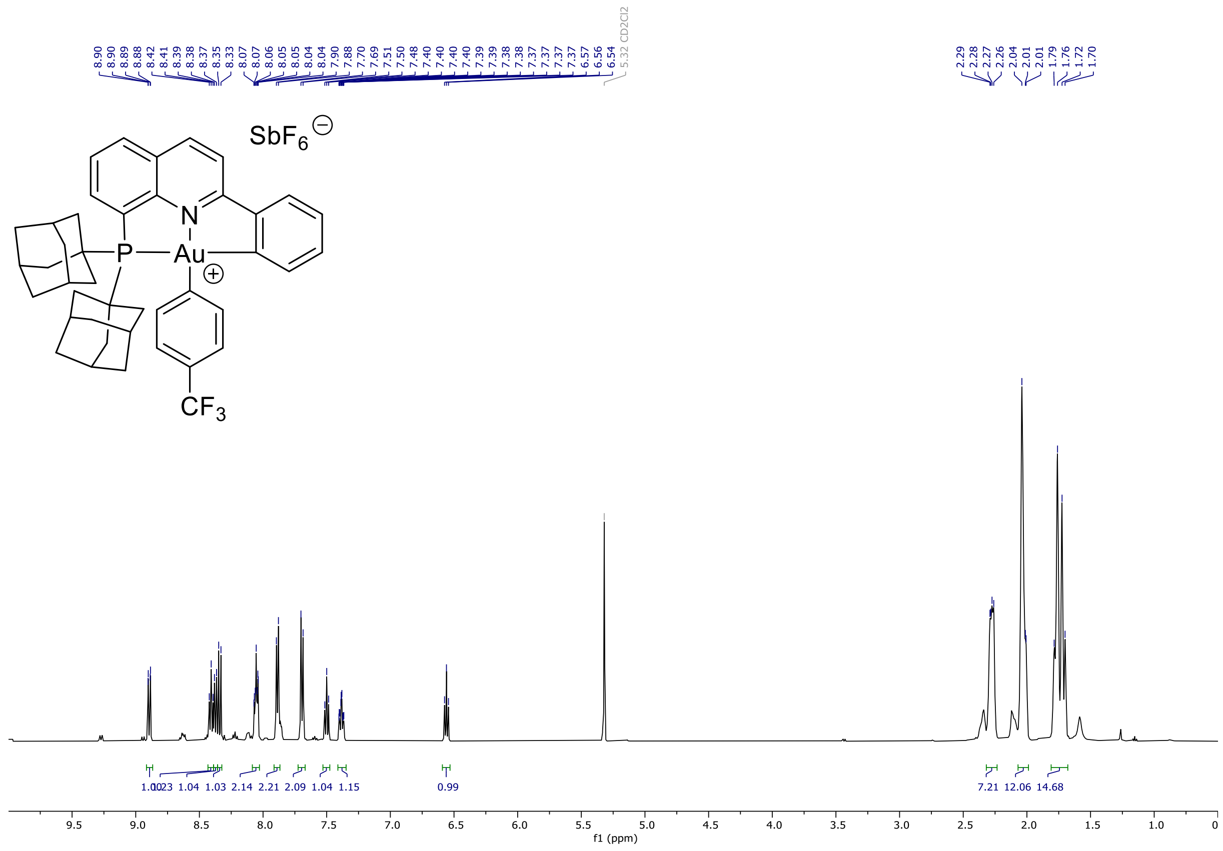

Figure S51. ^{1}H NMR (500.30 MHz, CD_2Cl_2, 298 K) of compound **1d**.

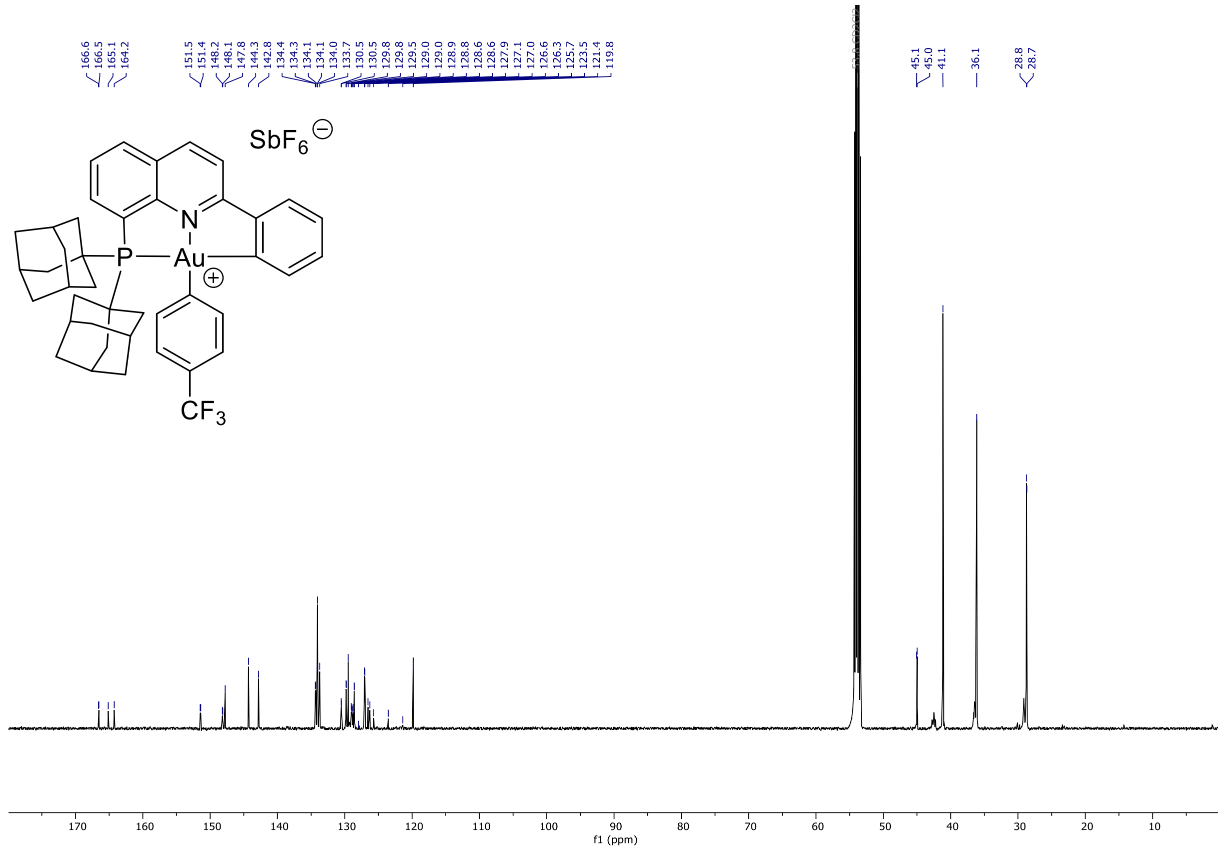

Figure S52. $^{13}C\{^{1}H\}$ NMR (125.81 MHz, CD_2Cl_2, 298 K) of compound **1d**.

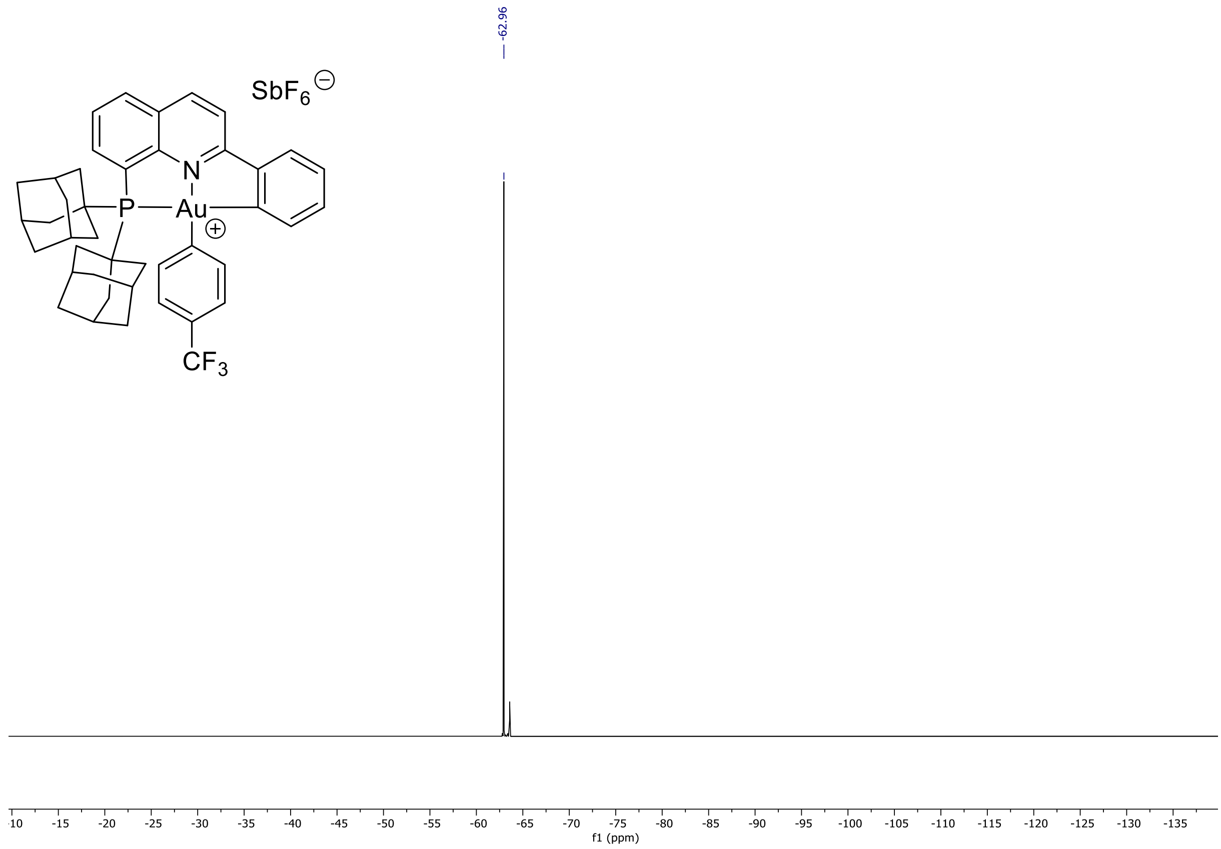

Figure S53. ^{19}F NMR (470.71 MHz, CD_2Cl_2, 298 K) of compound **1d**.

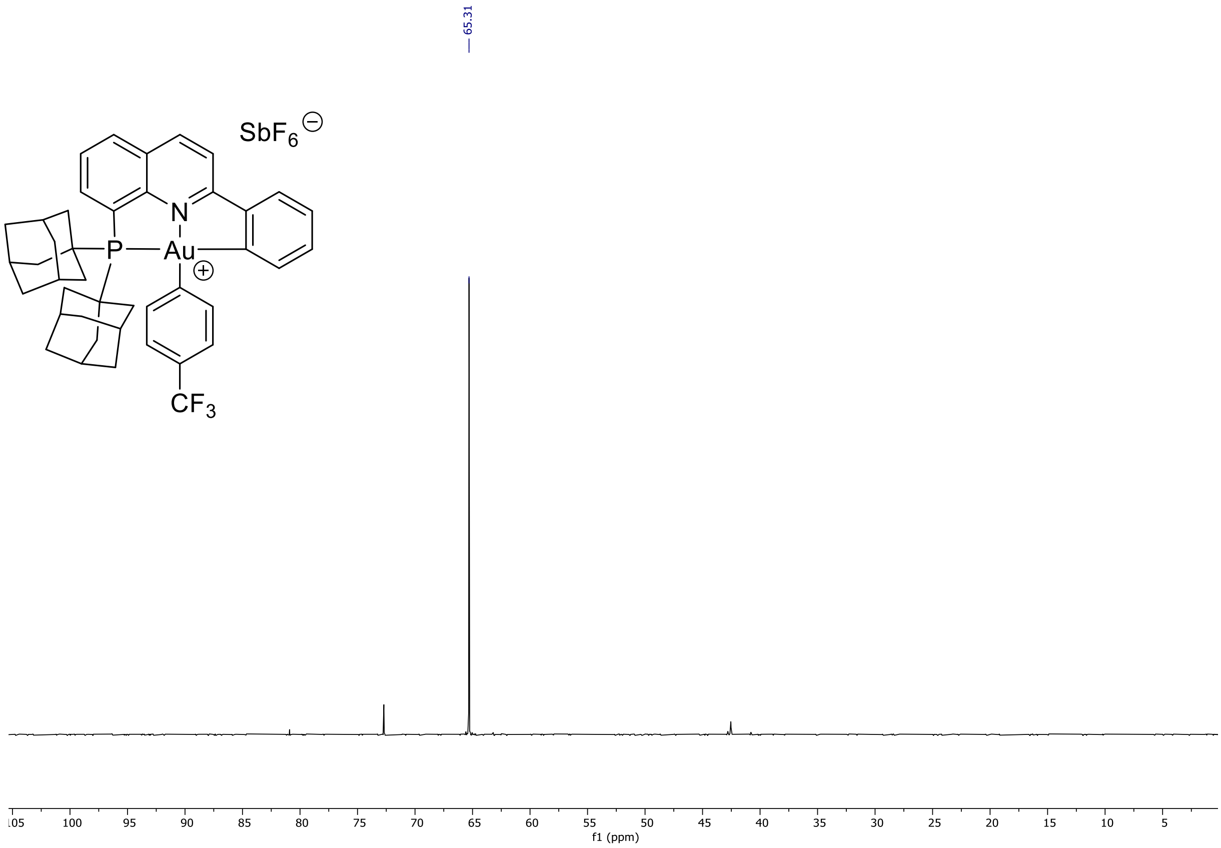

Figure S54. $^{31}P\{^1H\}$ NMR (202.52 MHz, CD_2Cl_2, 298 K) of compound **1d**.

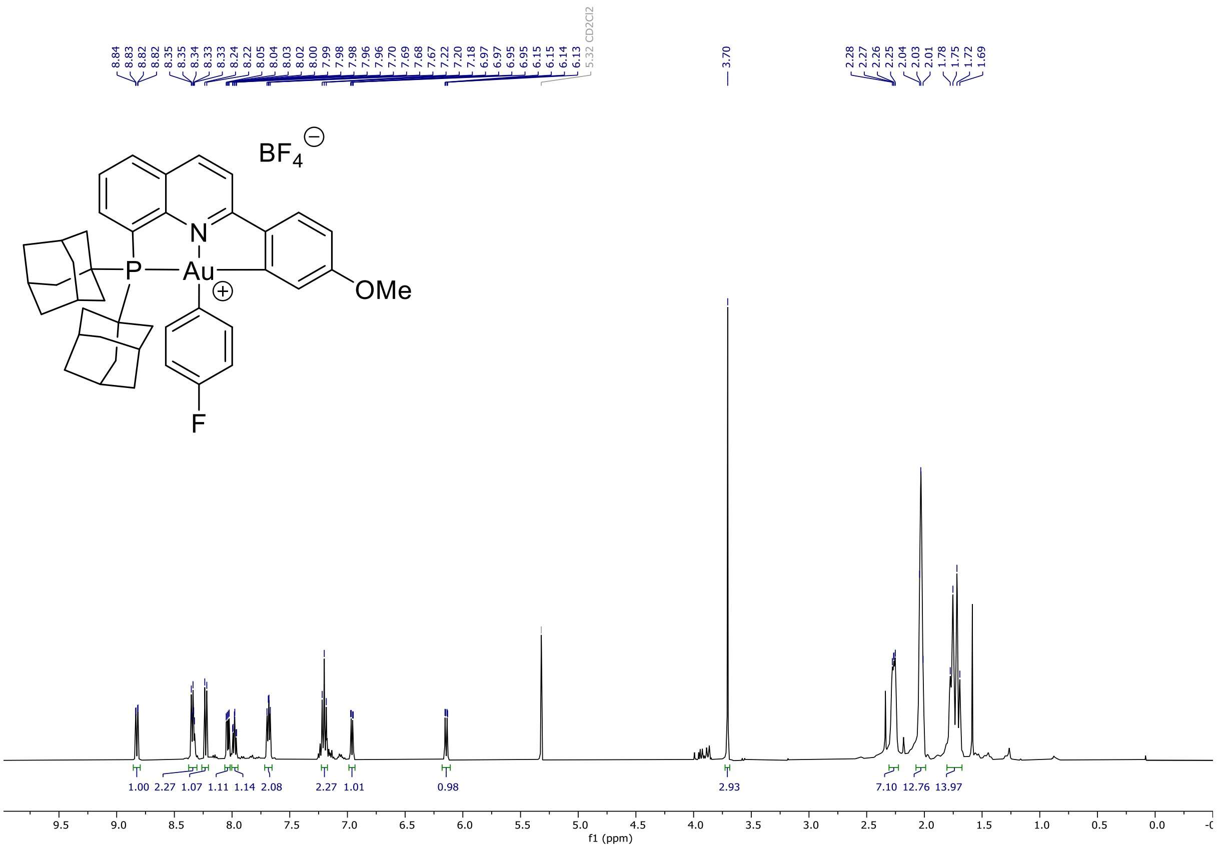

Figure S55. ^{1}H NMR (500.30 MHz, CD_2Cl_2, 298 K) of compound **1e**.

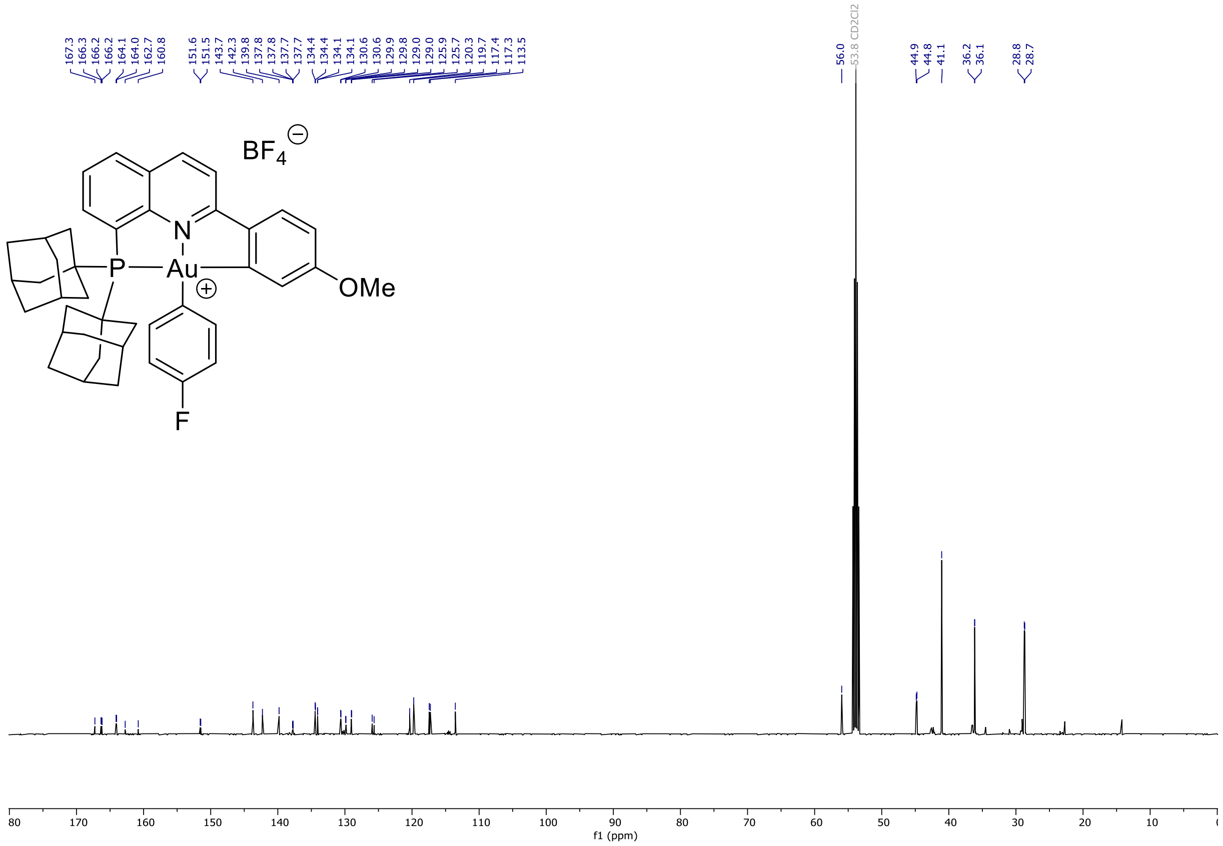

Figure S56. $^{13}C\{^{1}H\}$ NMR (125.81 MHz, CD_2Cl_2, 298 K) of compound **1e**.

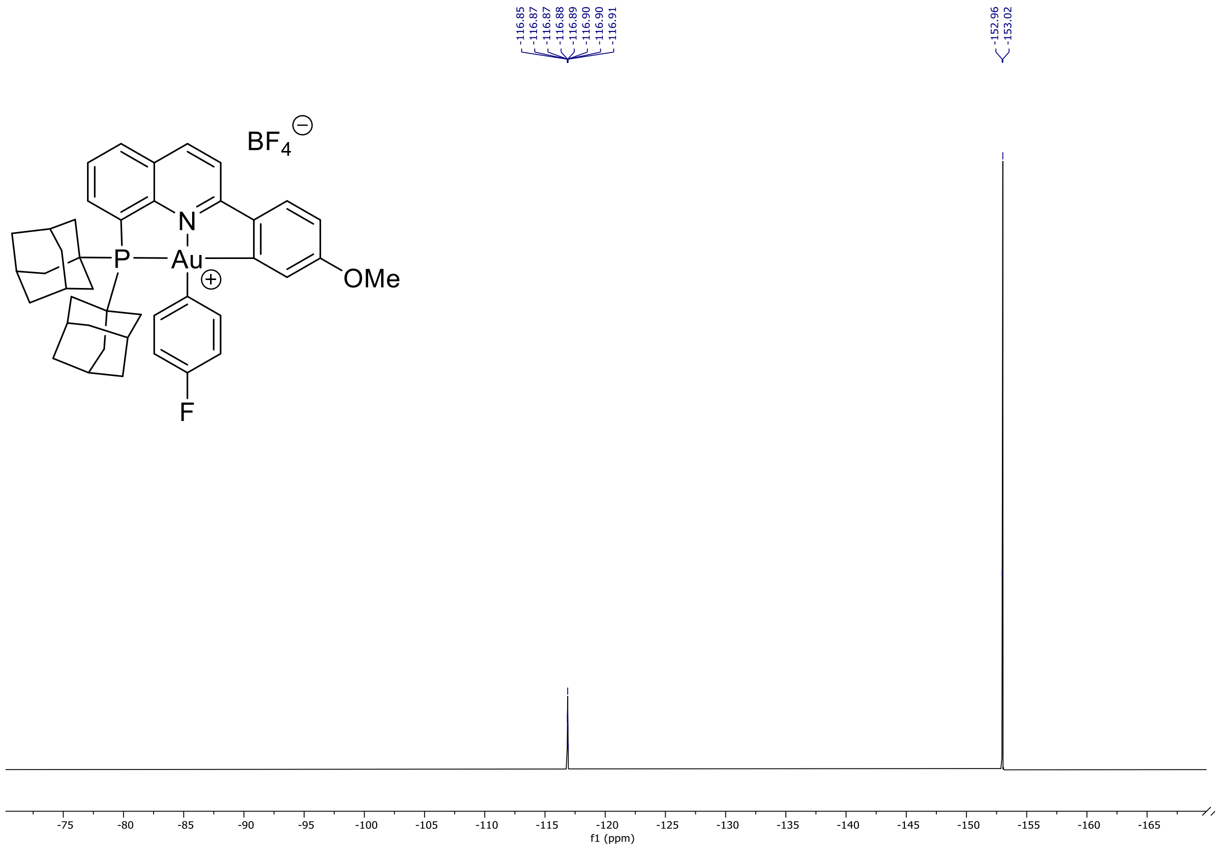

Figure S57. ^{19}F NMR (470.71 MHz, CD_2Cl_2, 298 K) of compound **1e**.

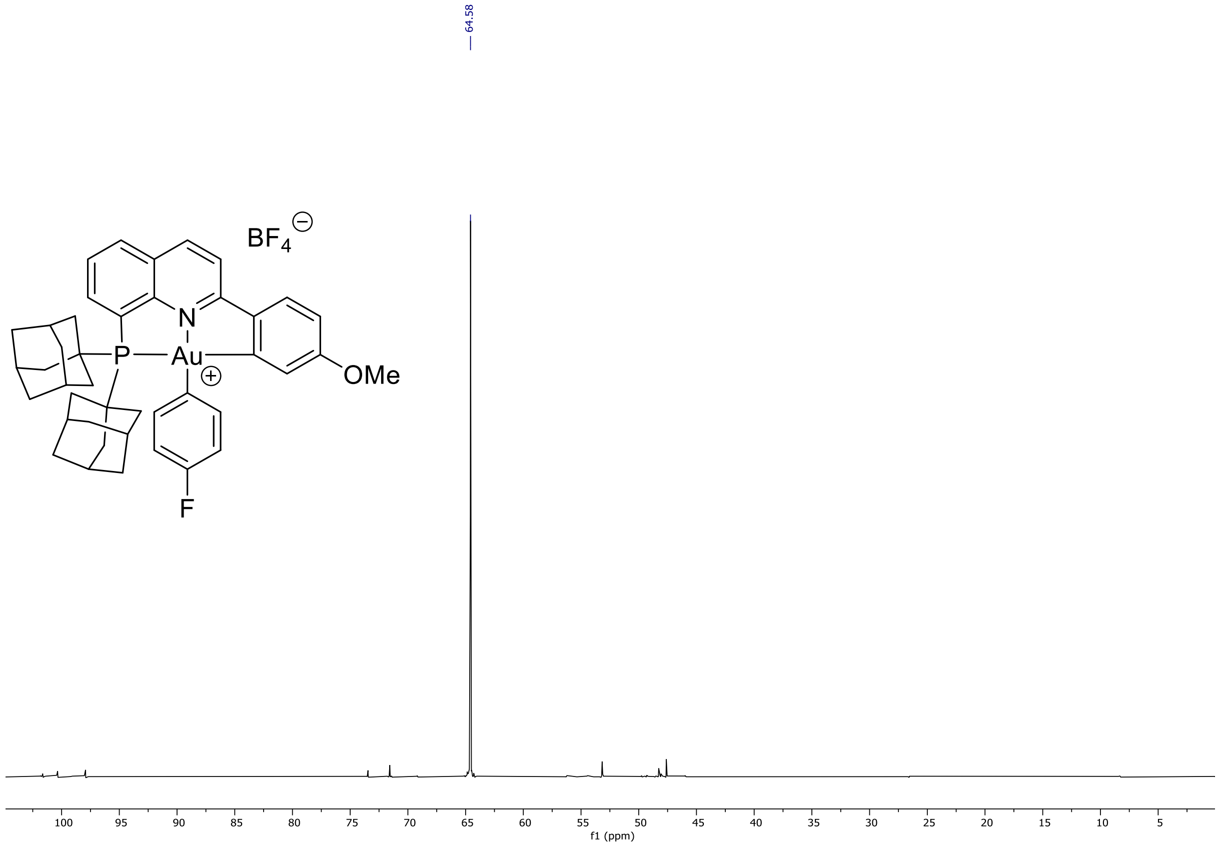

Figure S58. $^{31}P\{^1H\}$ NMR (202.52 MHz, CD_2Cl_2, 298 K) of compound **1e**.

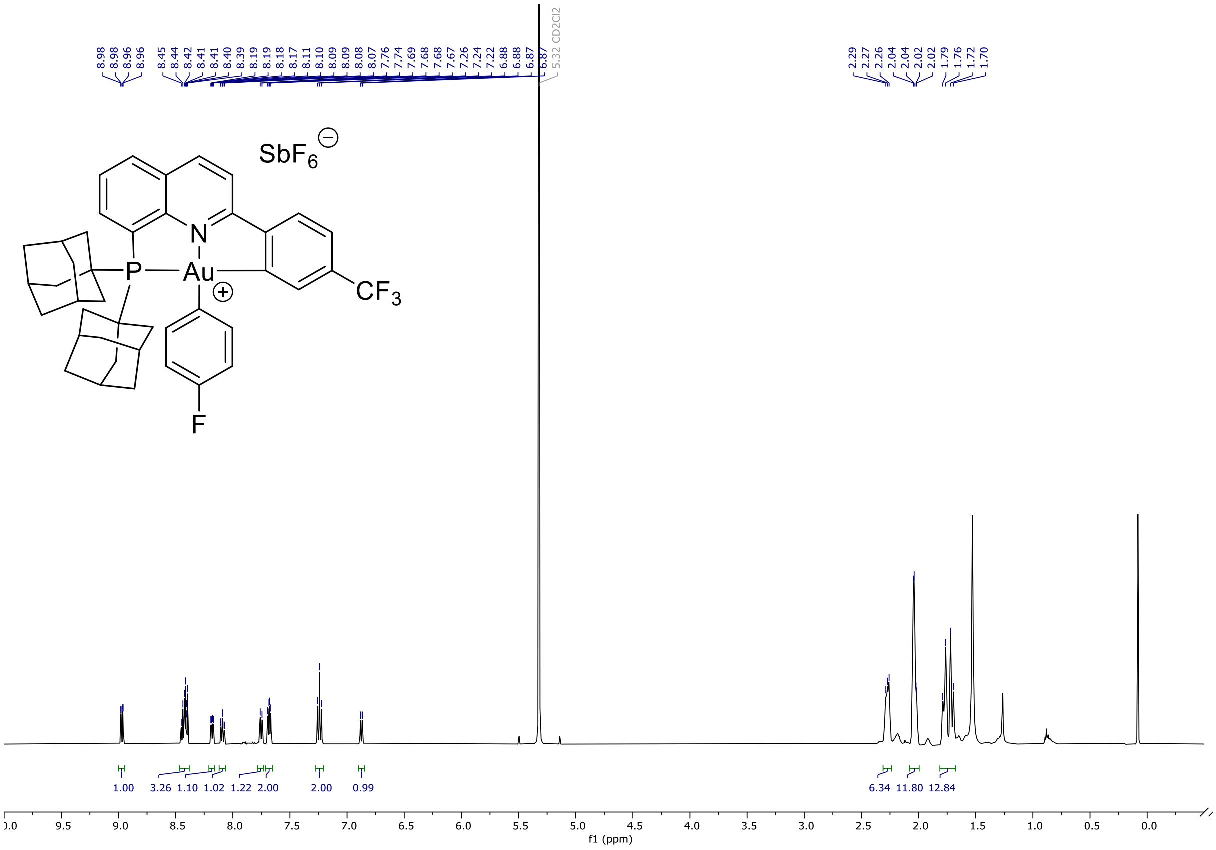

Figure S59. ^{1}H NMR (500.30 MHz, CD_2Cl_2, 298 K) of compound **1f**.

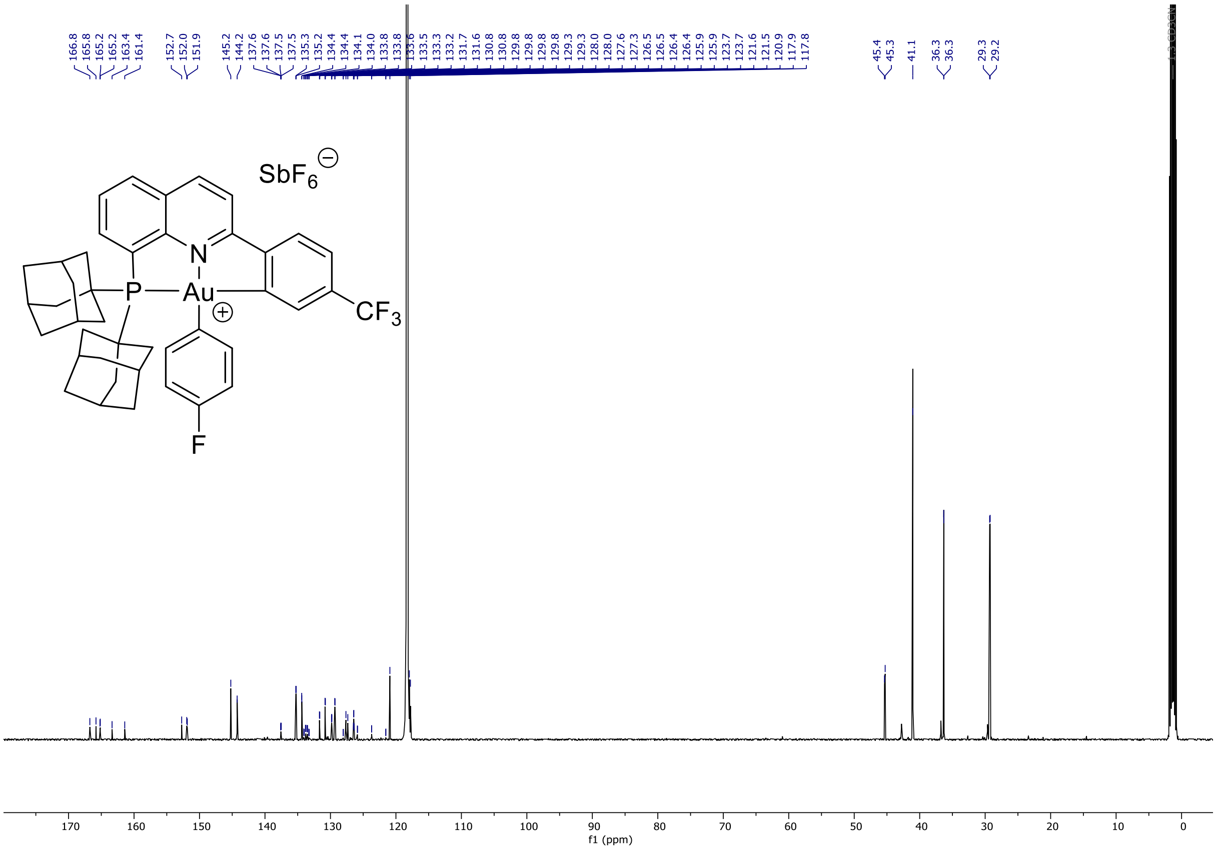

Figure S60. $^{13}C\{^{1}H\}$ NMR (125.81 MHz, CD_3CN, 298 K) of compound **1f**.

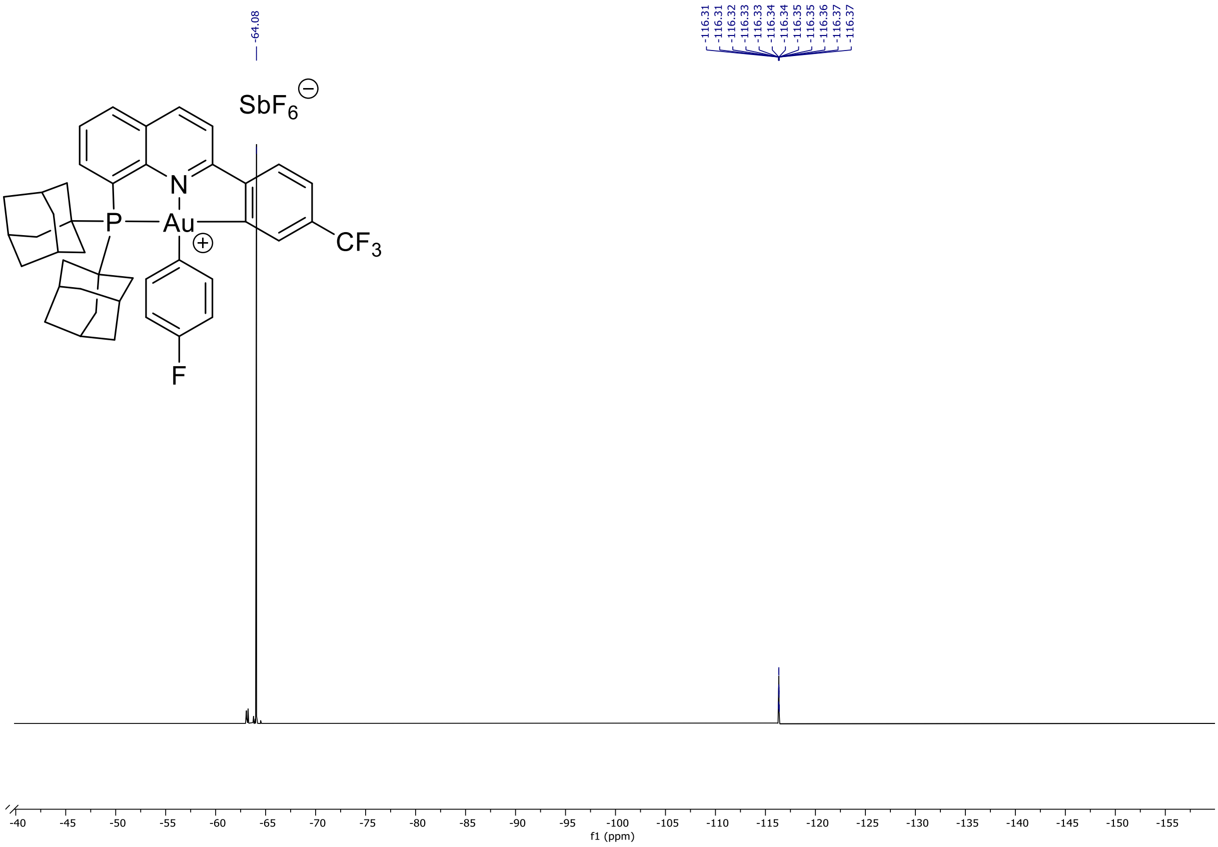

Figure S62. ^{19}F NMR (470.71 MHz, CD_2Cl_2, 298 K) of compound **1f**.

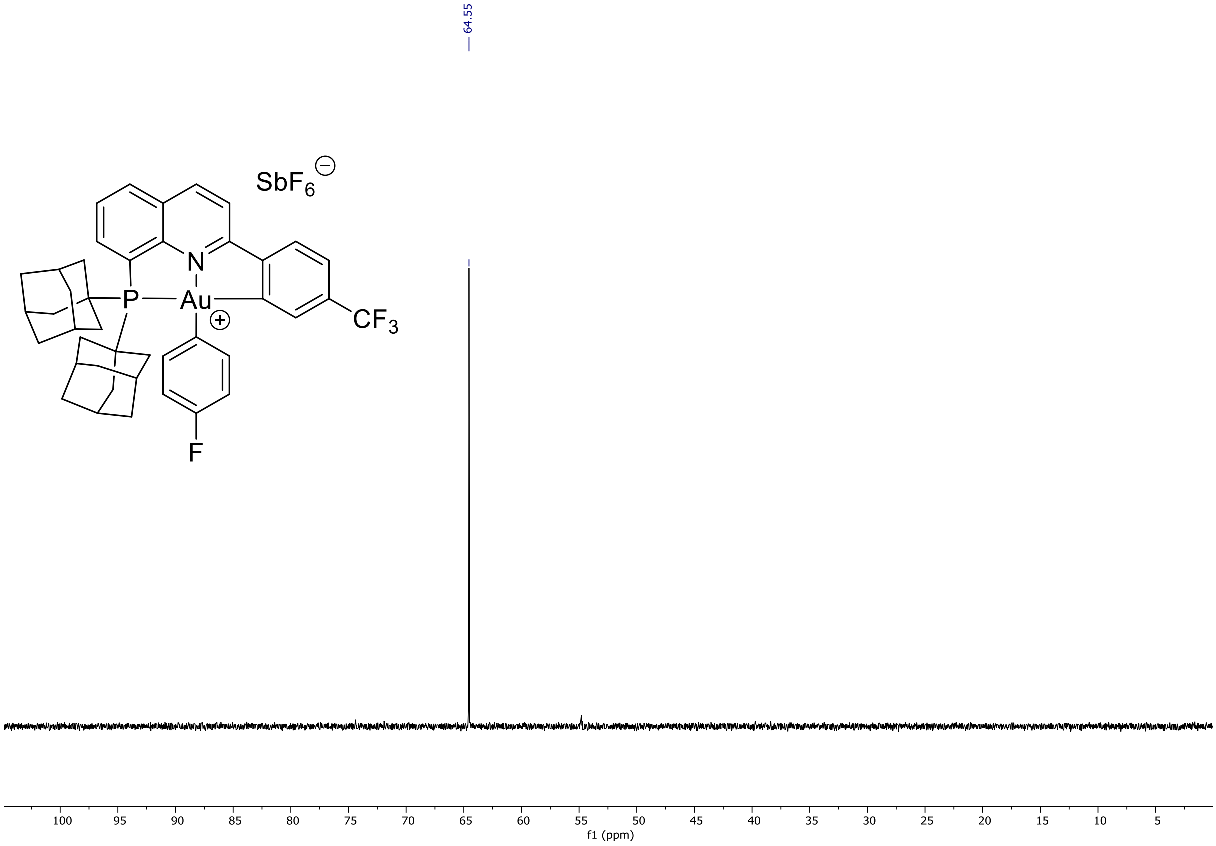

Figure S62. $^{31}P\{^1H\}$ NMR (202.52 MHz, CD_2Cl_2, 298 K) of compound **1f**.

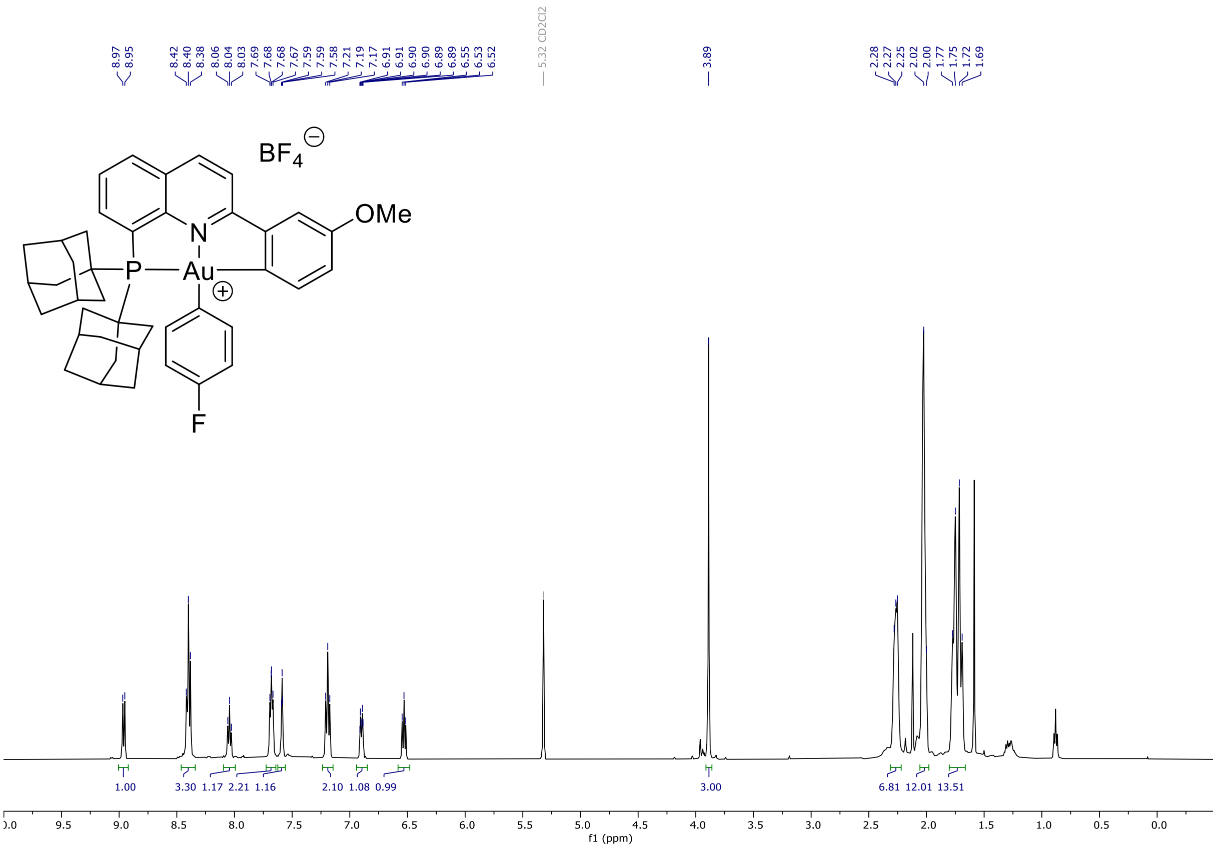

Figure S63. ^{1}H NMR (500.30 MHz, CD_2Cl_2, 298 K) of compound **1g**.

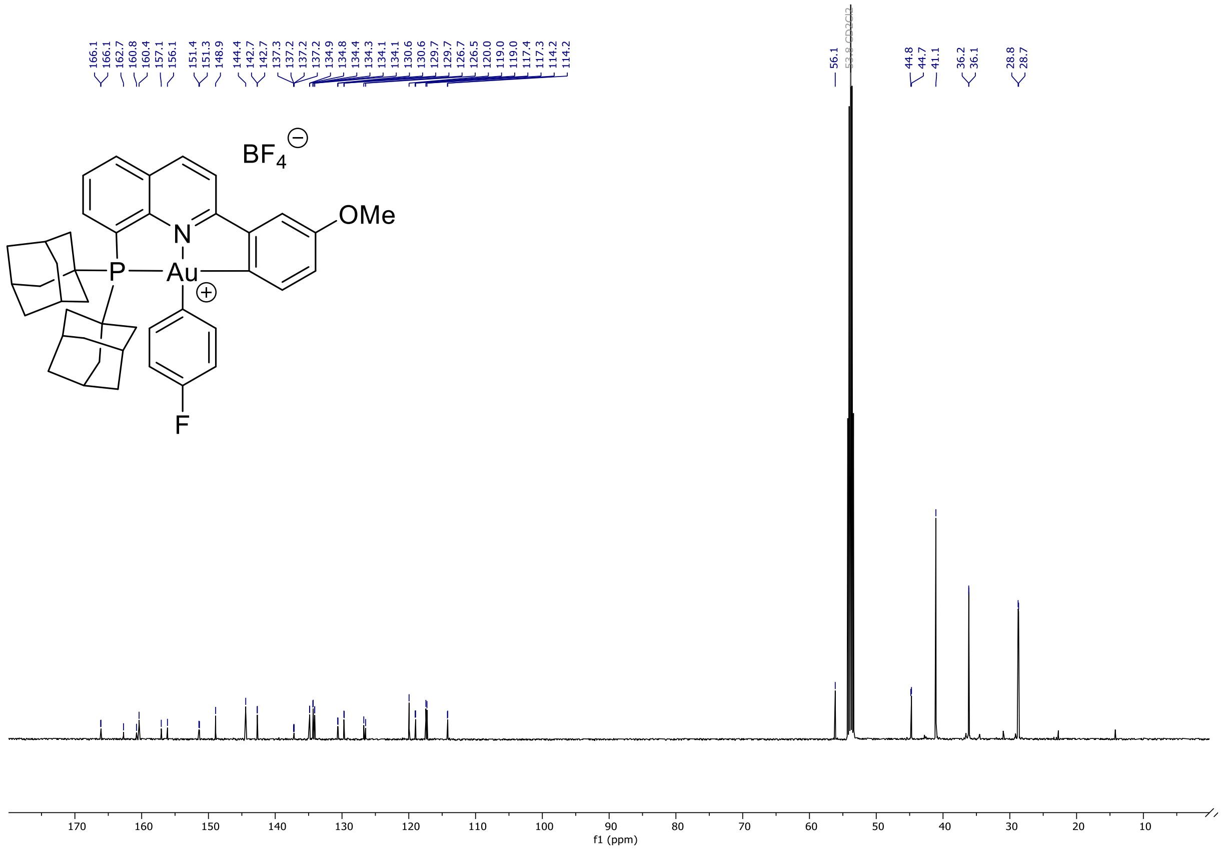

Figure S64. $^{13}C\{^{1}H\}$ NMR (125.81 MHz, CD_2Cl_2, 298 K) of compound **1g**.

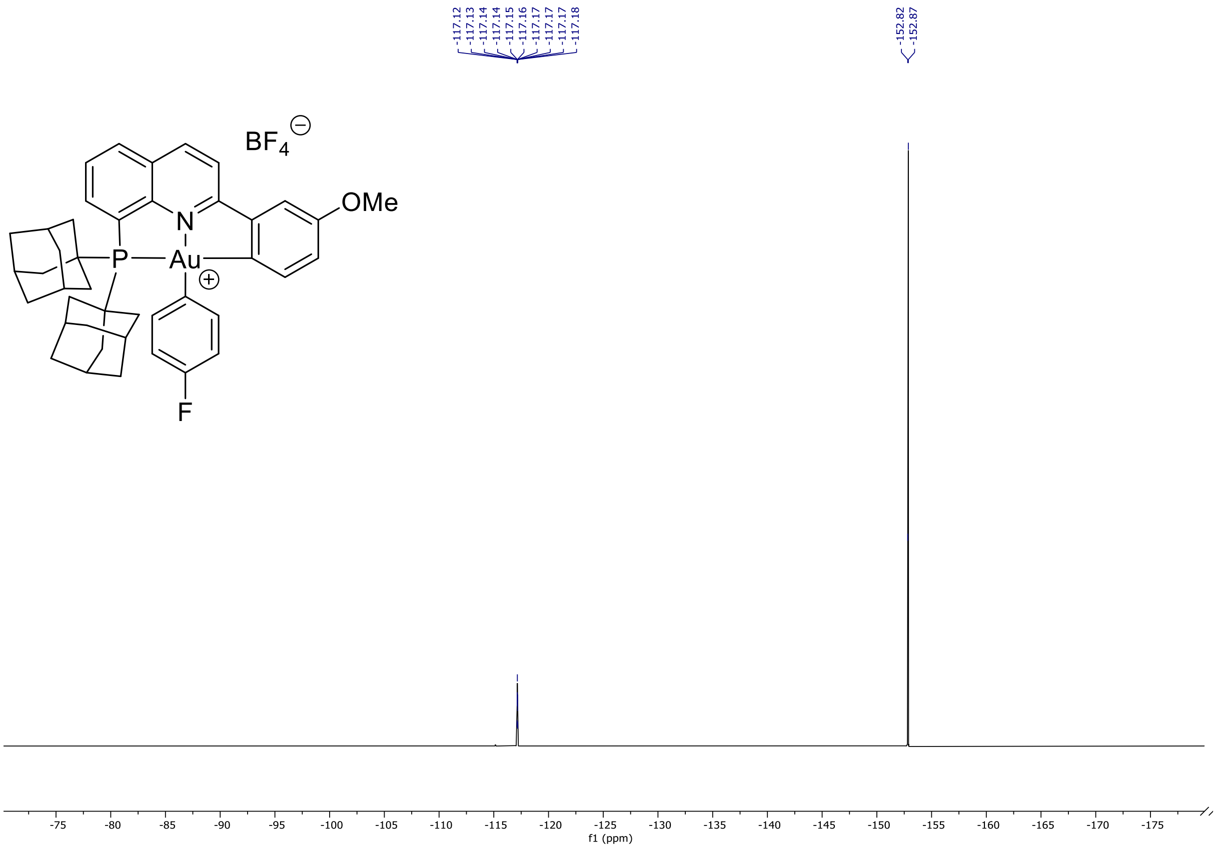

Figure S65. ^{19}F NMR (470.71 MHz, CD_2Cl_2, 298 K) of compound **1g**.

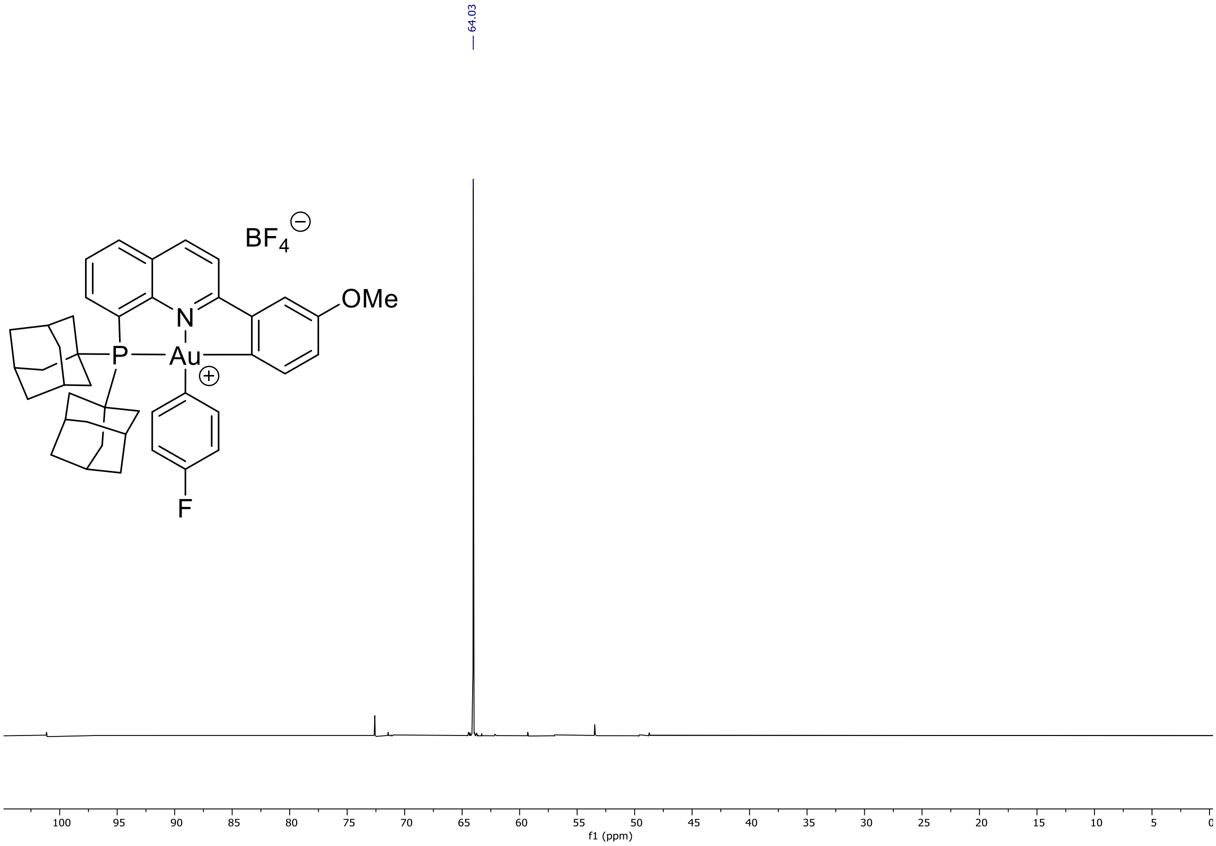

Figure S66. ^{31}P{^{1}H} NMR (202.52 MHz, CD_2Cl_2, 298 K) of compound **1g**.

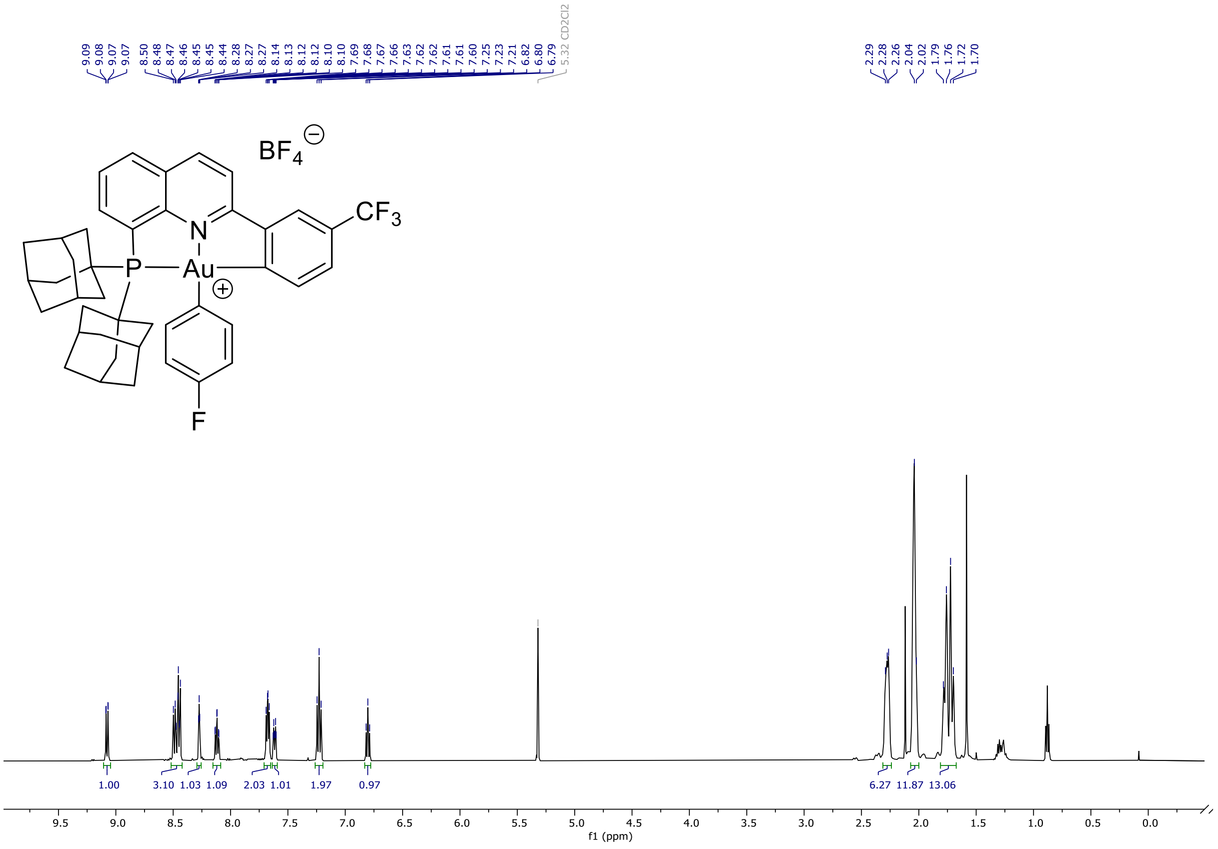

Figure S67. ^{1}H NMR (500.30 MHz, CD_2Cl_2, 298 K) of compound **1h**.

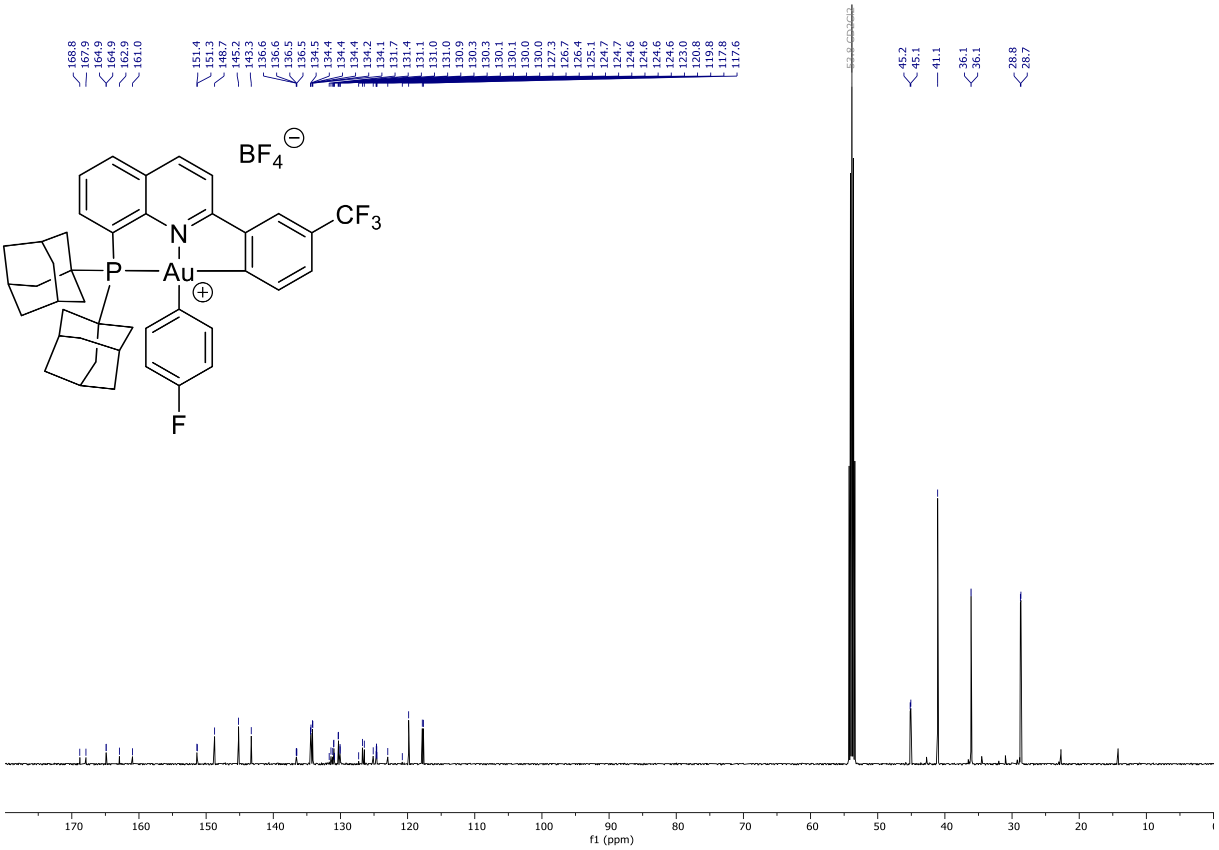

Figure S68. $^{13}C\{^{1}H\}$ NMR (125.81 MHz, CD_2Cl_2, 298 K) of compound **1h**.

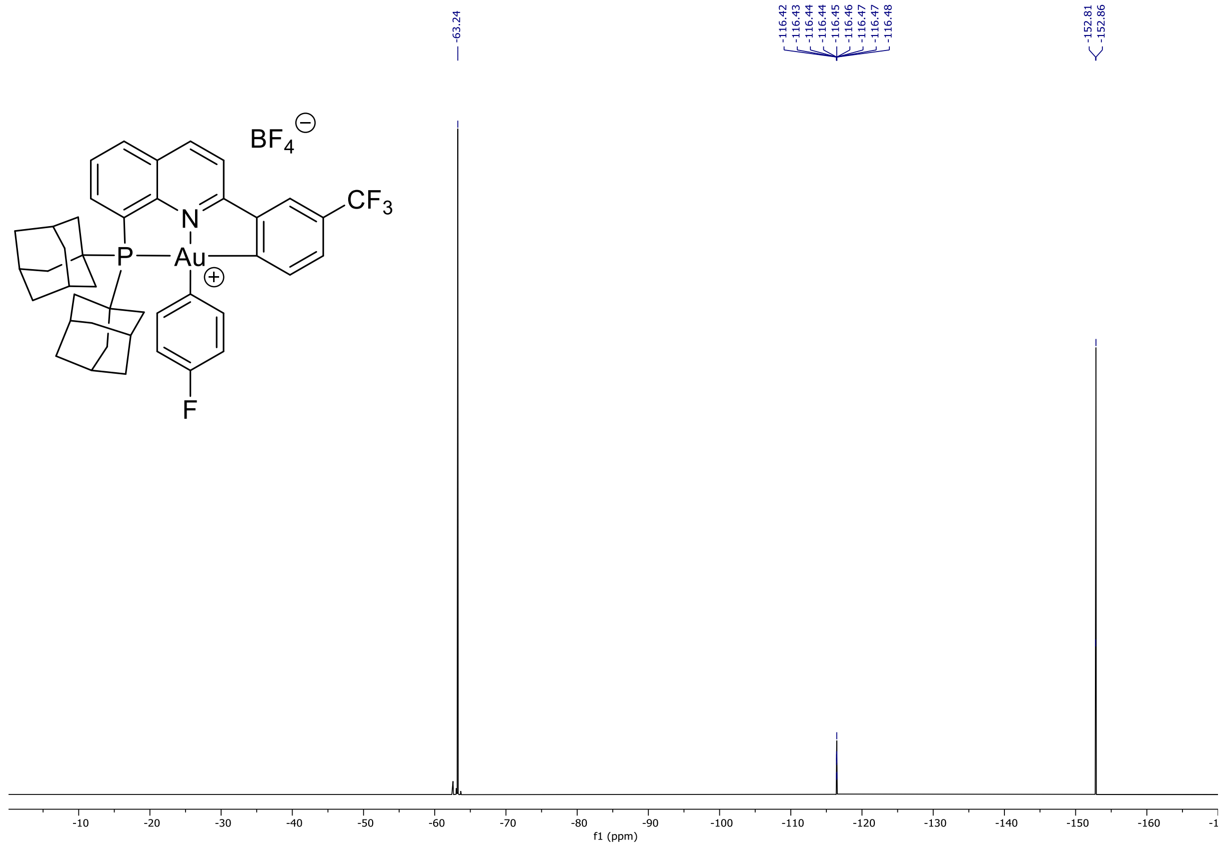

Figure S69. ^{19}F NMR (470.71 MHz, CD_2Cl_2, 298 K) of compound **1h**.

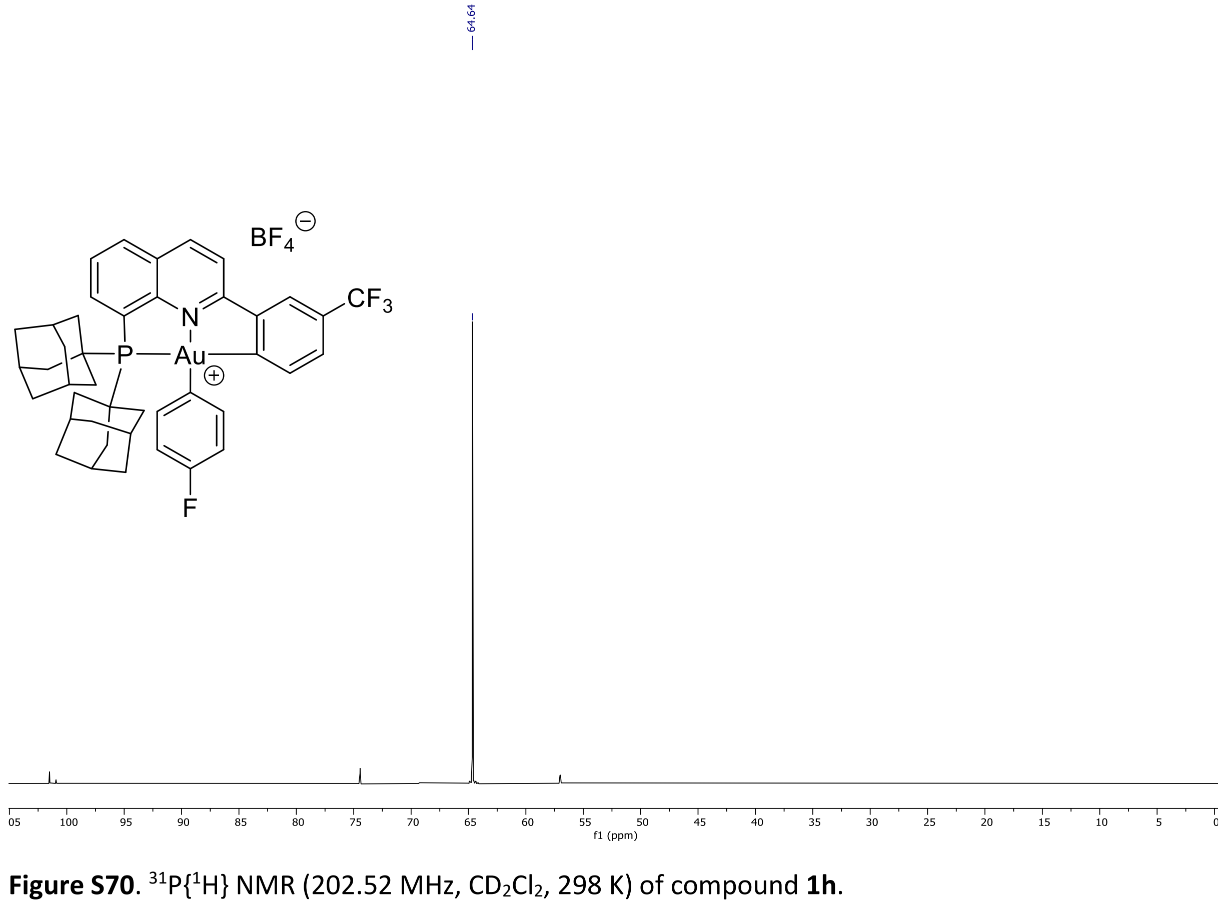

Figure S70. ^{31}P{^{1}H} NMR (202.52 MHz, CD_2Cl_2, 298 K) of compound **1h**.

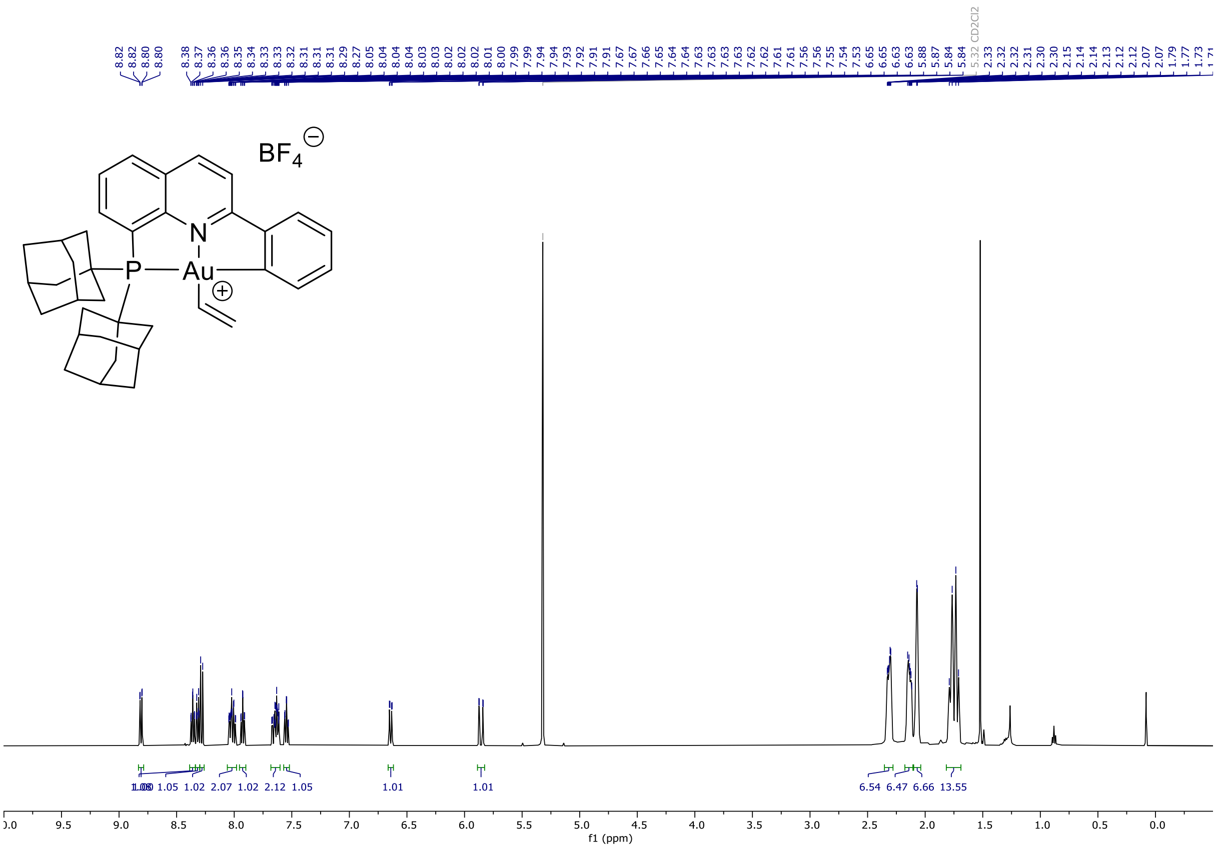

Figure S71. ^{1}H NMR (500.30 MHz, CD_2Cl_2, 298 K) of compound **2a**.

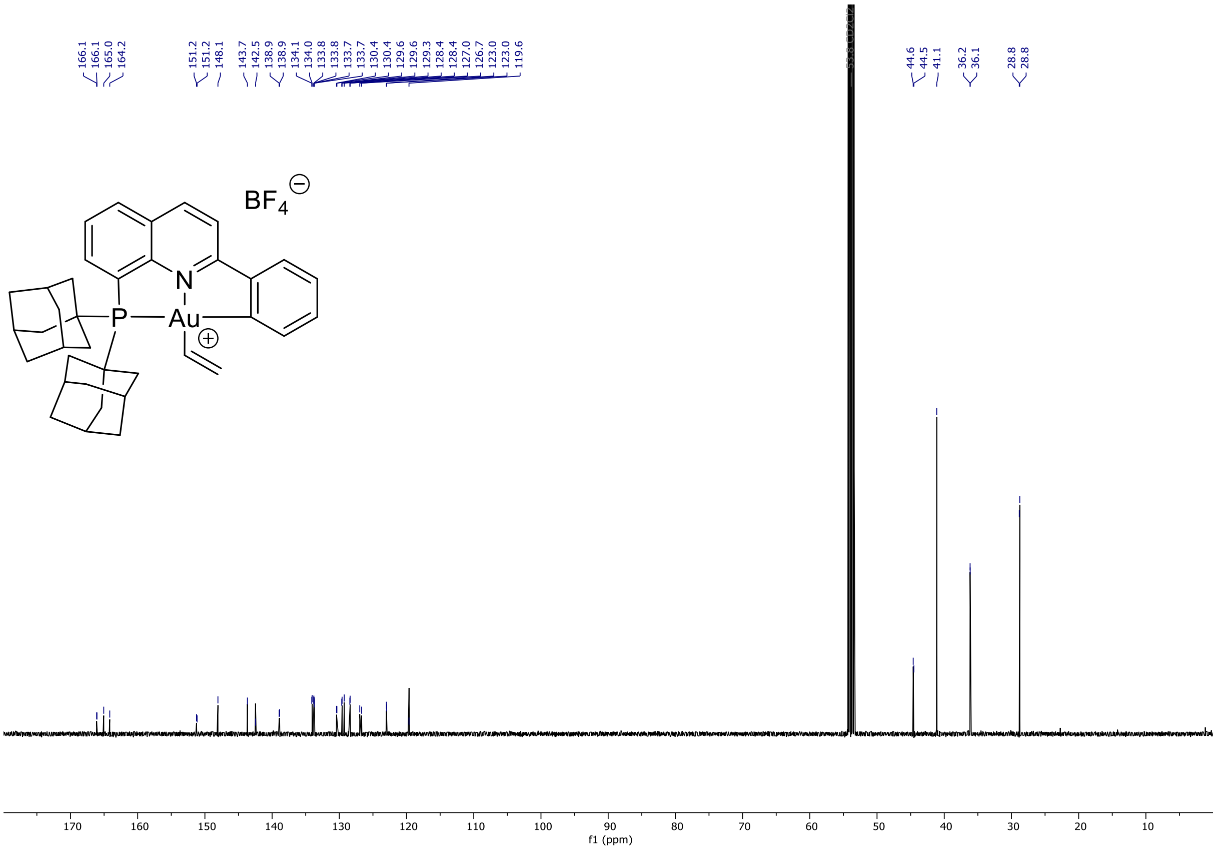

Figure S72. $^{13}C\{^{1}H\}$ NMR (125.81 MHz, CD_2Cl_2, 298 K) of compound **2a**.

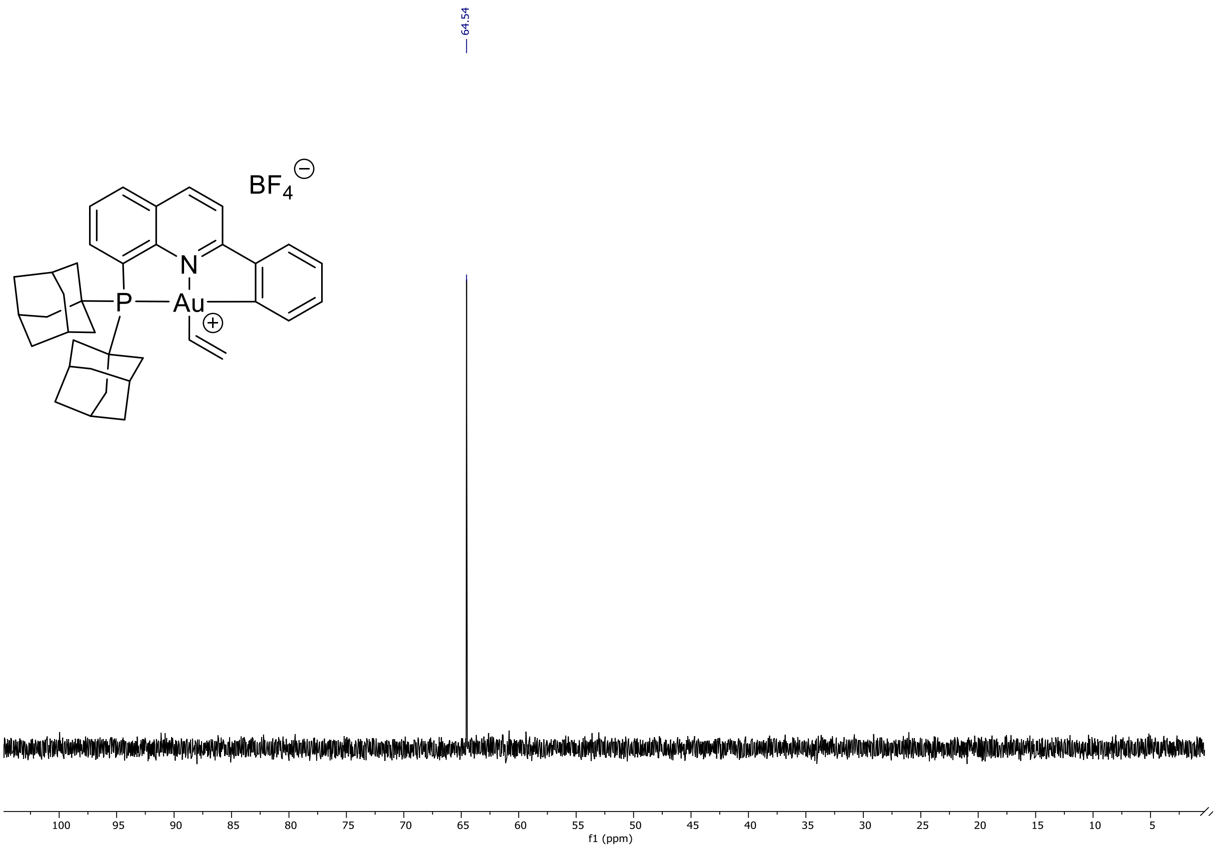

Figure S73. $^{31}P\{^{1}H\}$ NMR (162.02 MHz, CD_2Cl_2, 298 K) of compound **2a**.

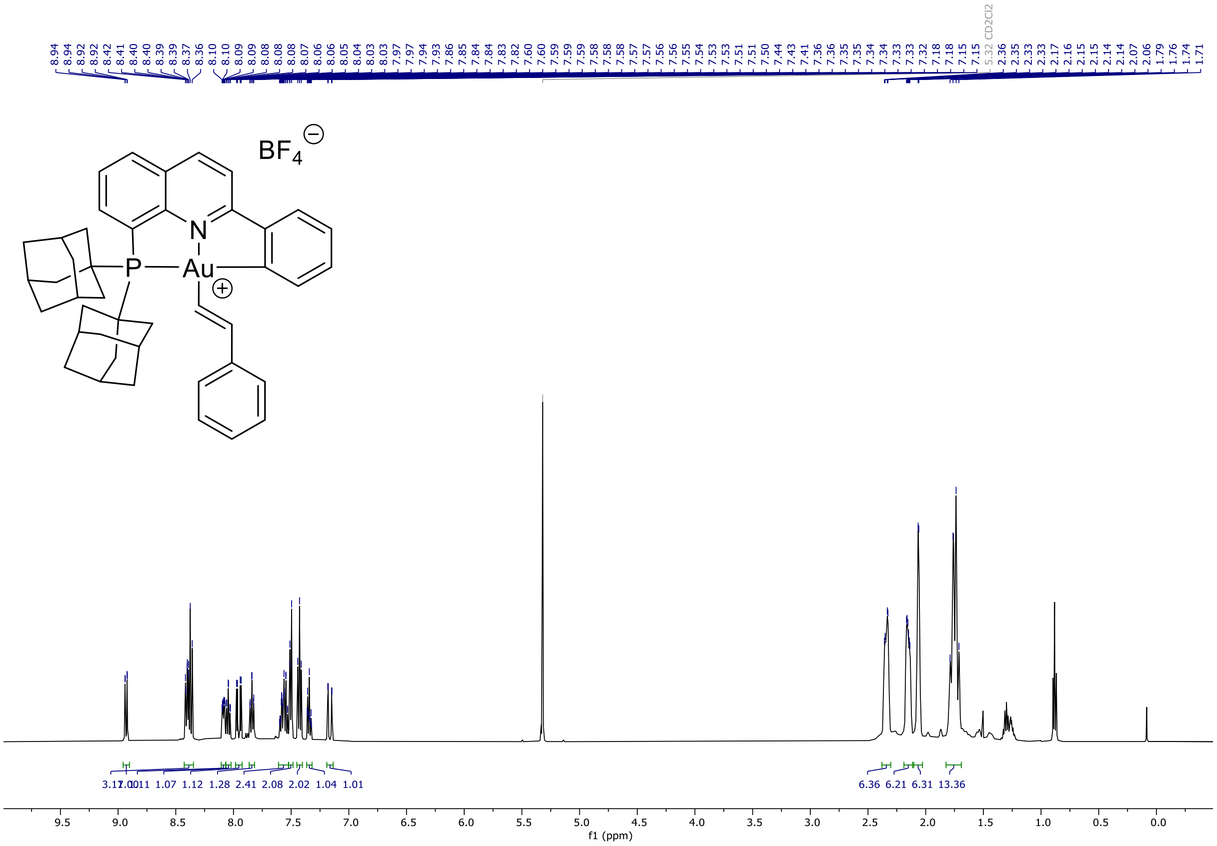

Figure S74. ^{1}H NMR (500.30 MHz, CD_2Cl_2, 298 K) of compound **2b**.

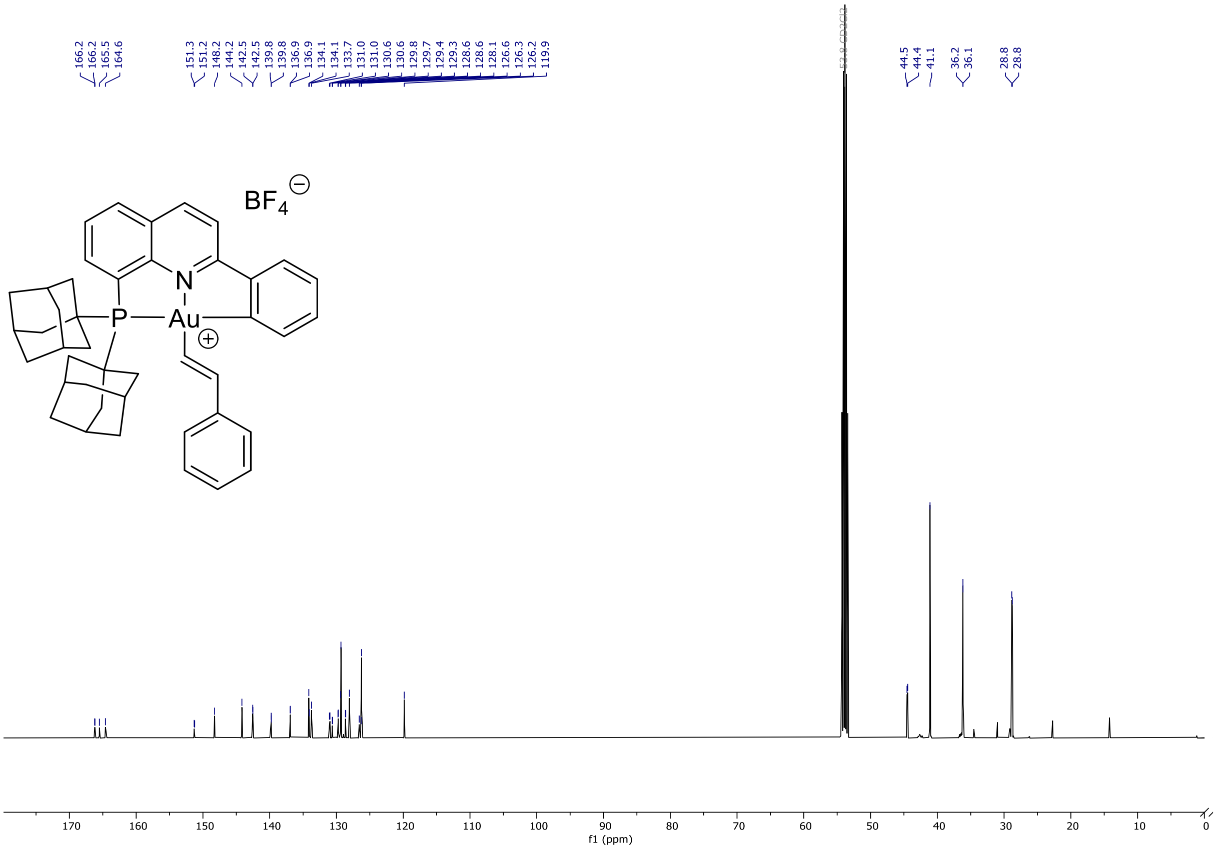

Figure S75. $^{13}C\{^1H\}$ NMR (125.81 MHz, CD_2Cl_2, 298 K) of compound **2b**.

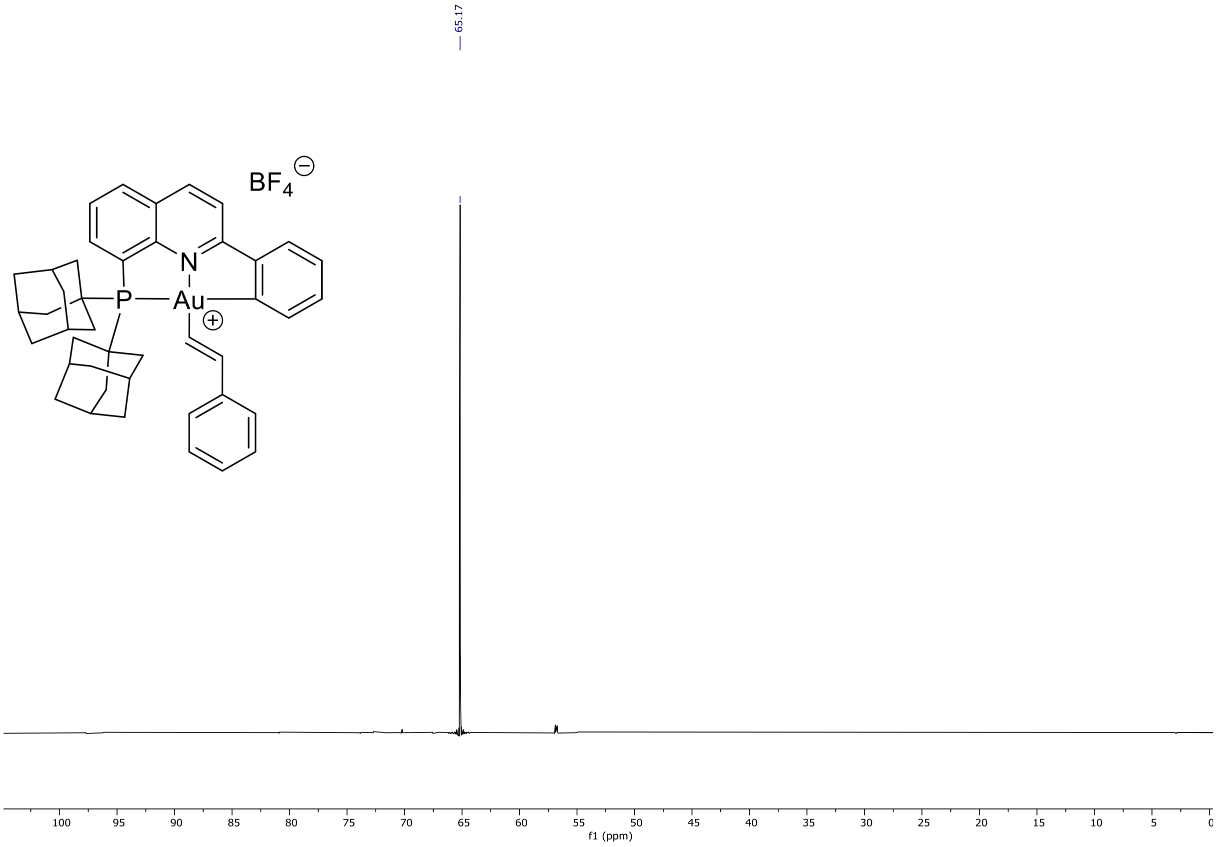

Figure S76. $^{31}P\{^1H\}$ NMR (202.52 MHz, CD_2Cl_2, 298 K) of compound **2b**.

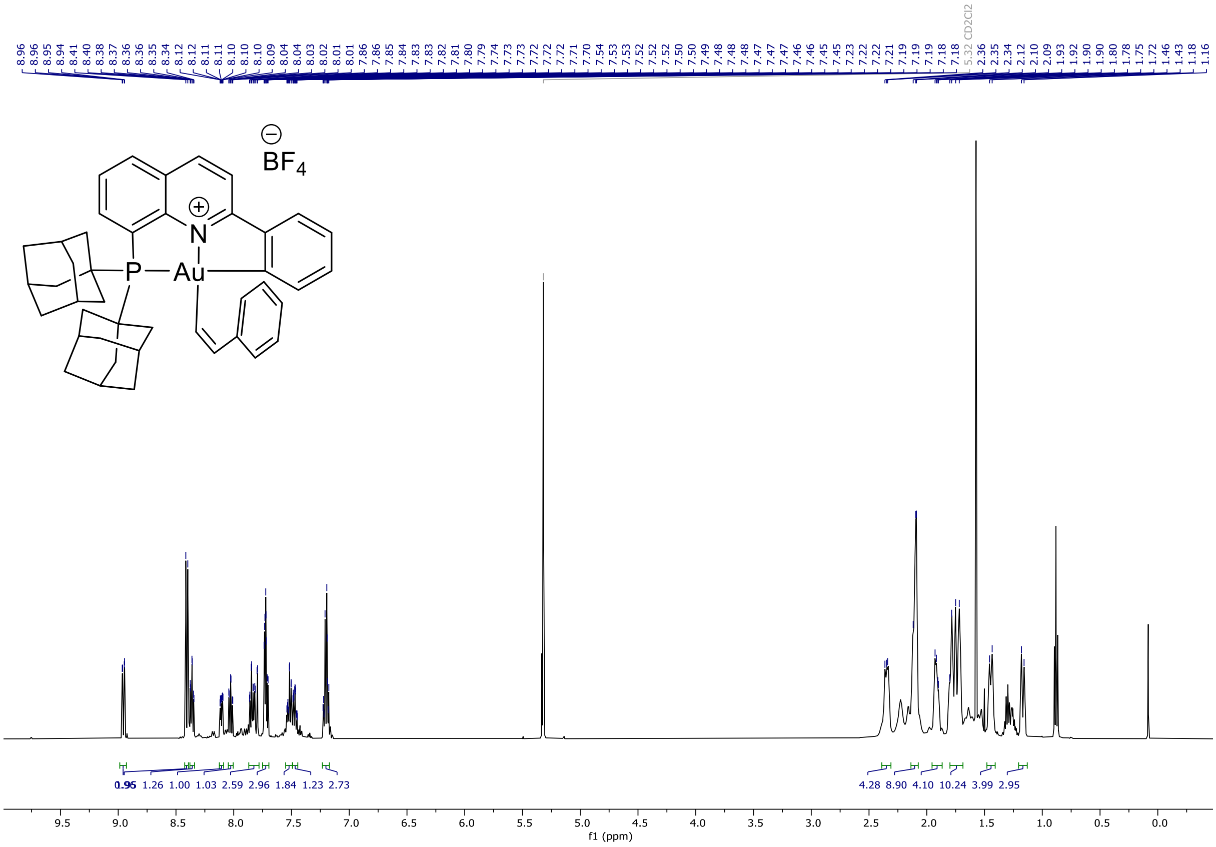

Figure S77. ^{1}H NMR (500.3 MHz, CD_2Cl_2, 298 K) of compound **2c**.

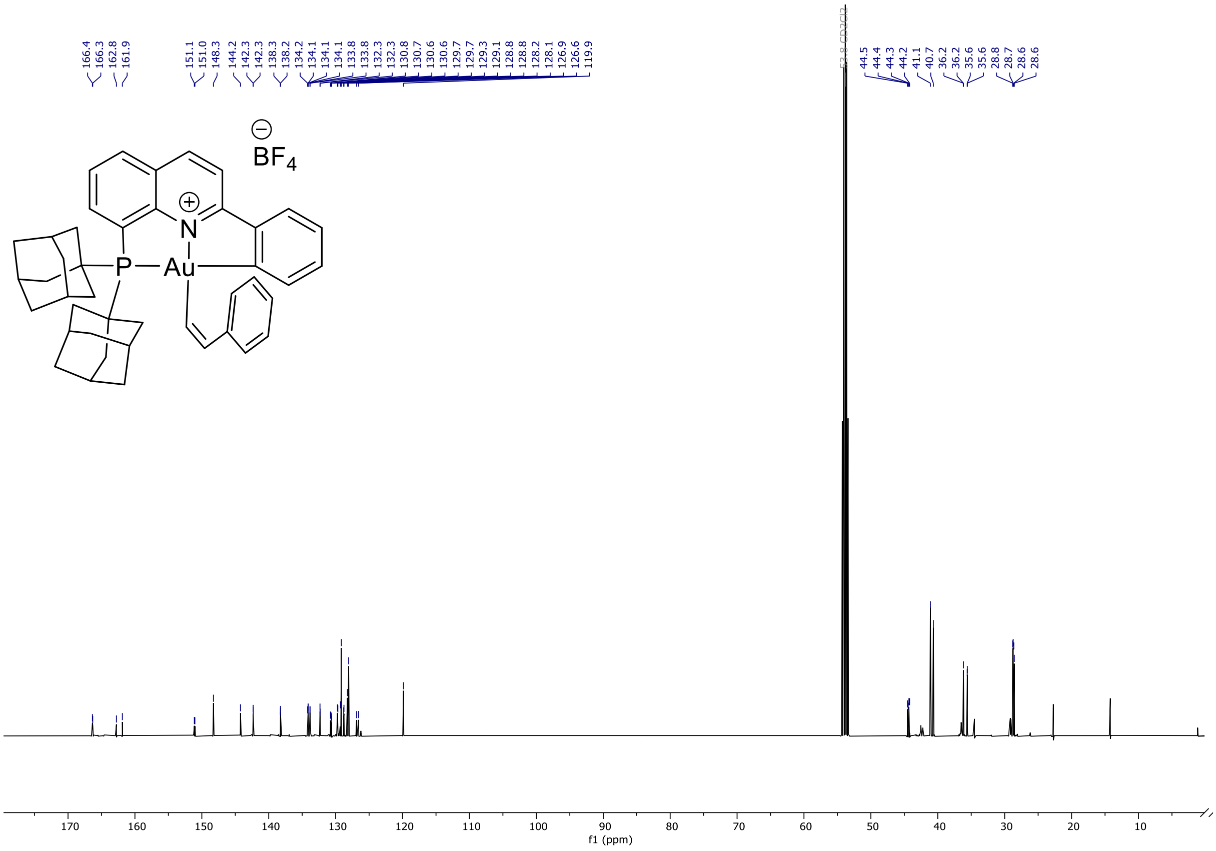

Figure S78. $^{13}C\{^{1}H\}$ NMR (125.81 MHz, CD_2Cl_2, 298 K) of compound **2c**.

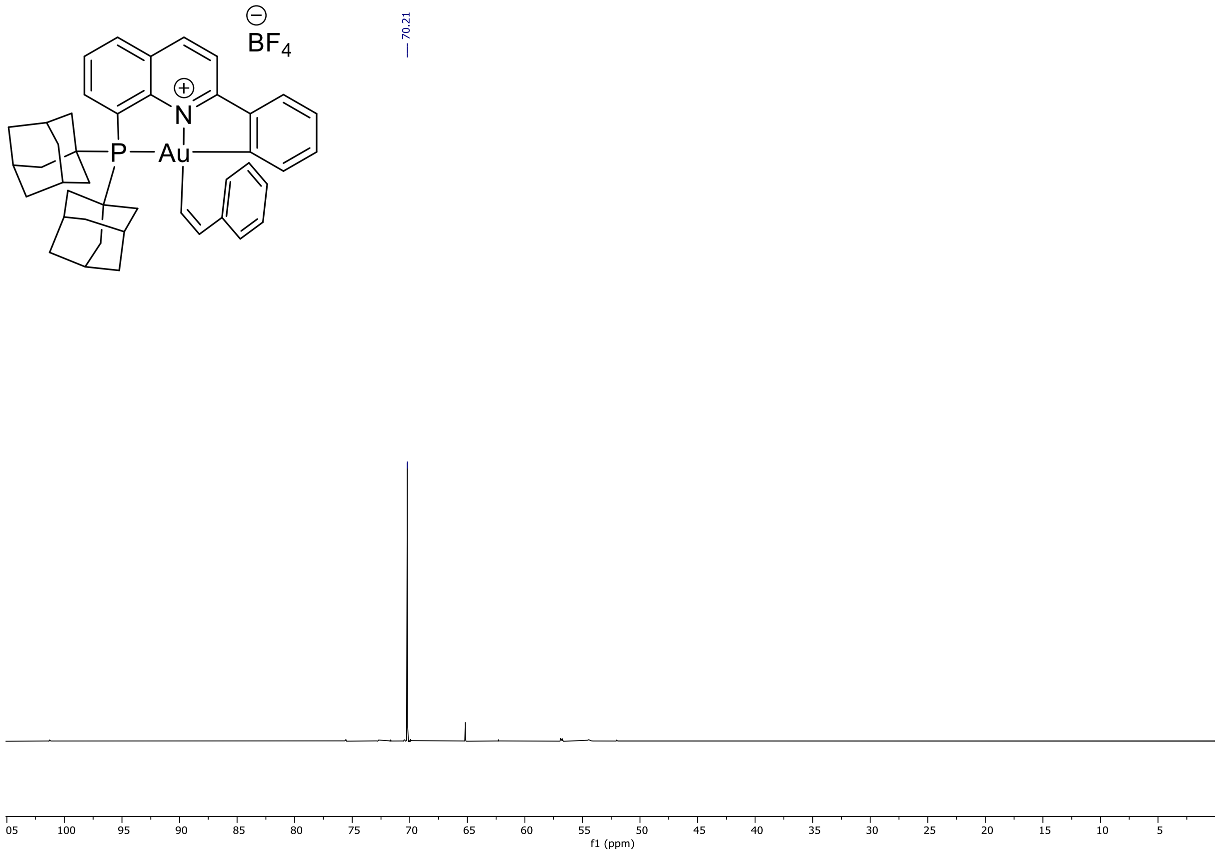

Figure S79. $^{31}P\{^{1}H\}$ NMR (202.52 MHz, CD_2Cl_2, 298 K) of compound **2c**.

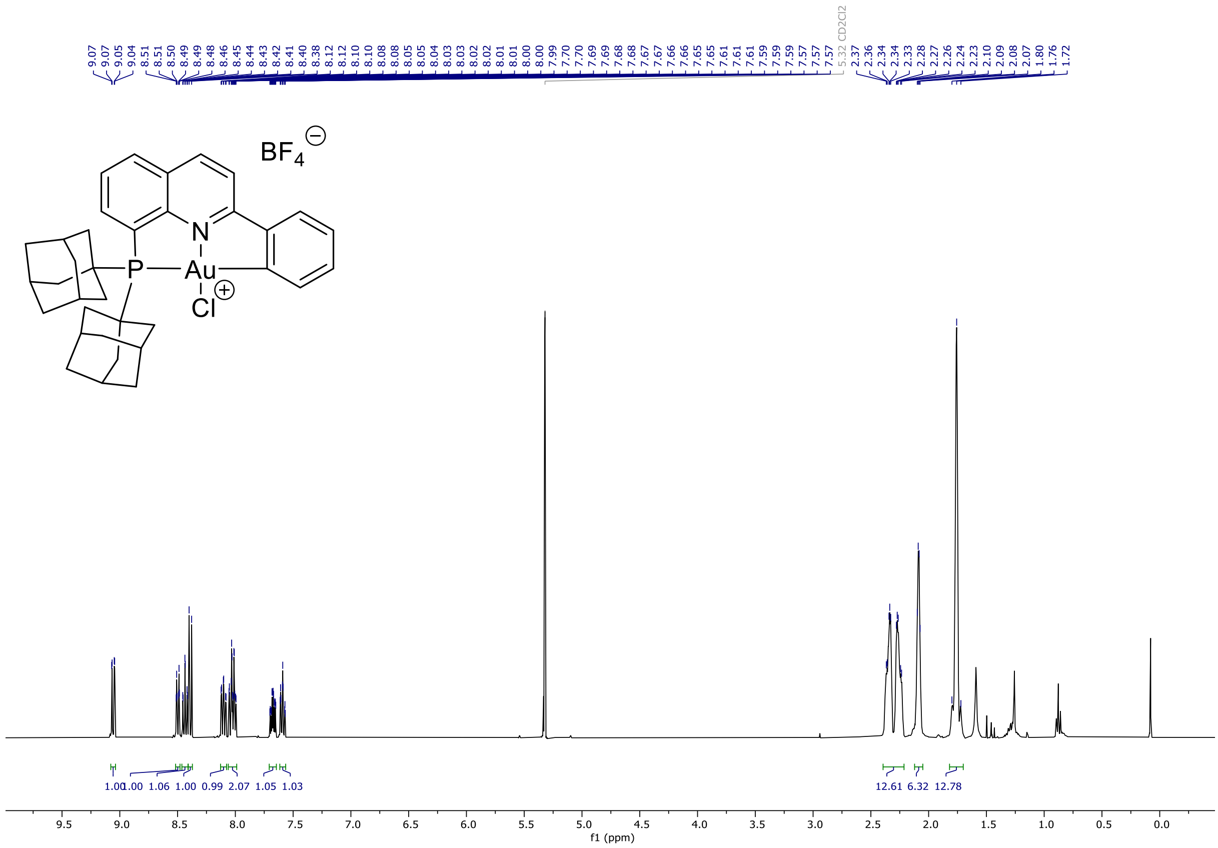

Figure S80. ^{1}H NMR (500.30 MHz, CD_2Cl_2, 298 K) of compound **3**.

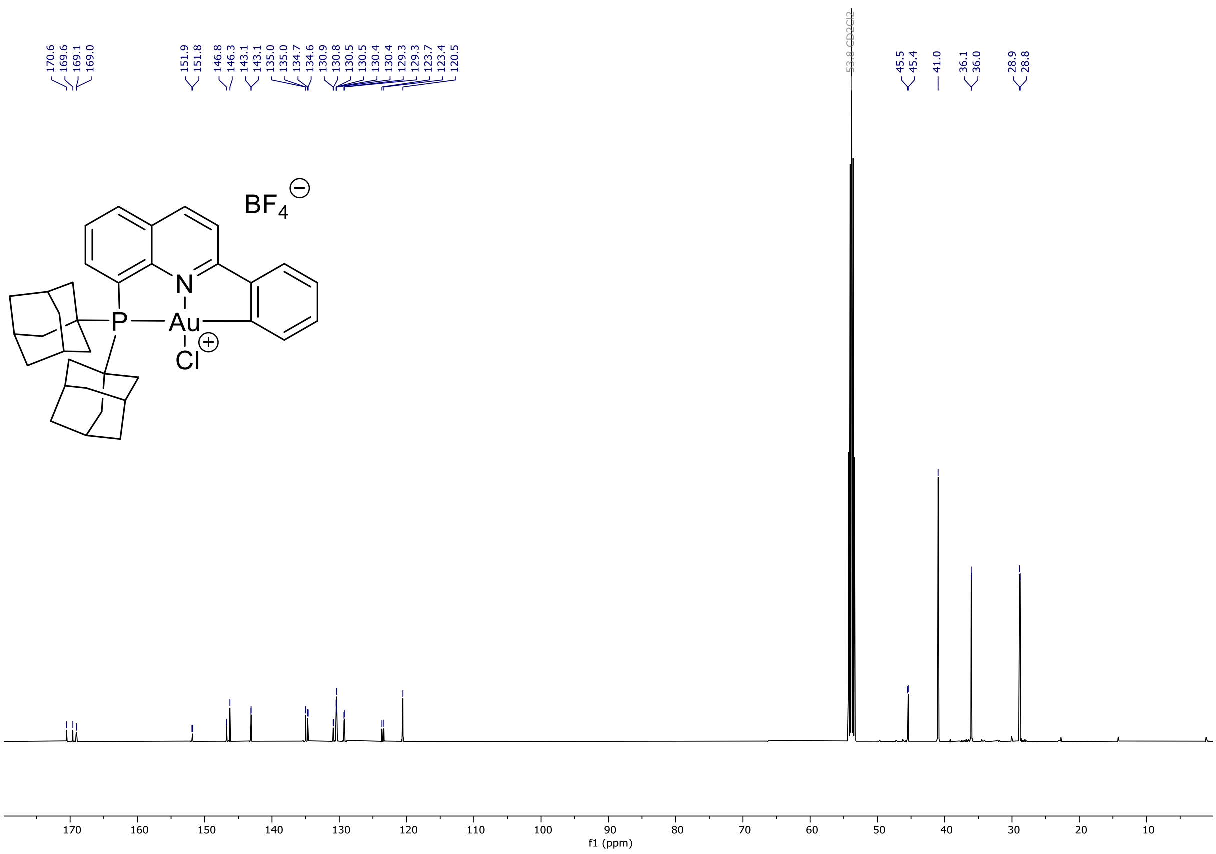

Figure S81. $^{13}C\{^{1}H\}$ NMR (125.81 MHz, CD_2Cl_2, 298 K) of compound **3**.

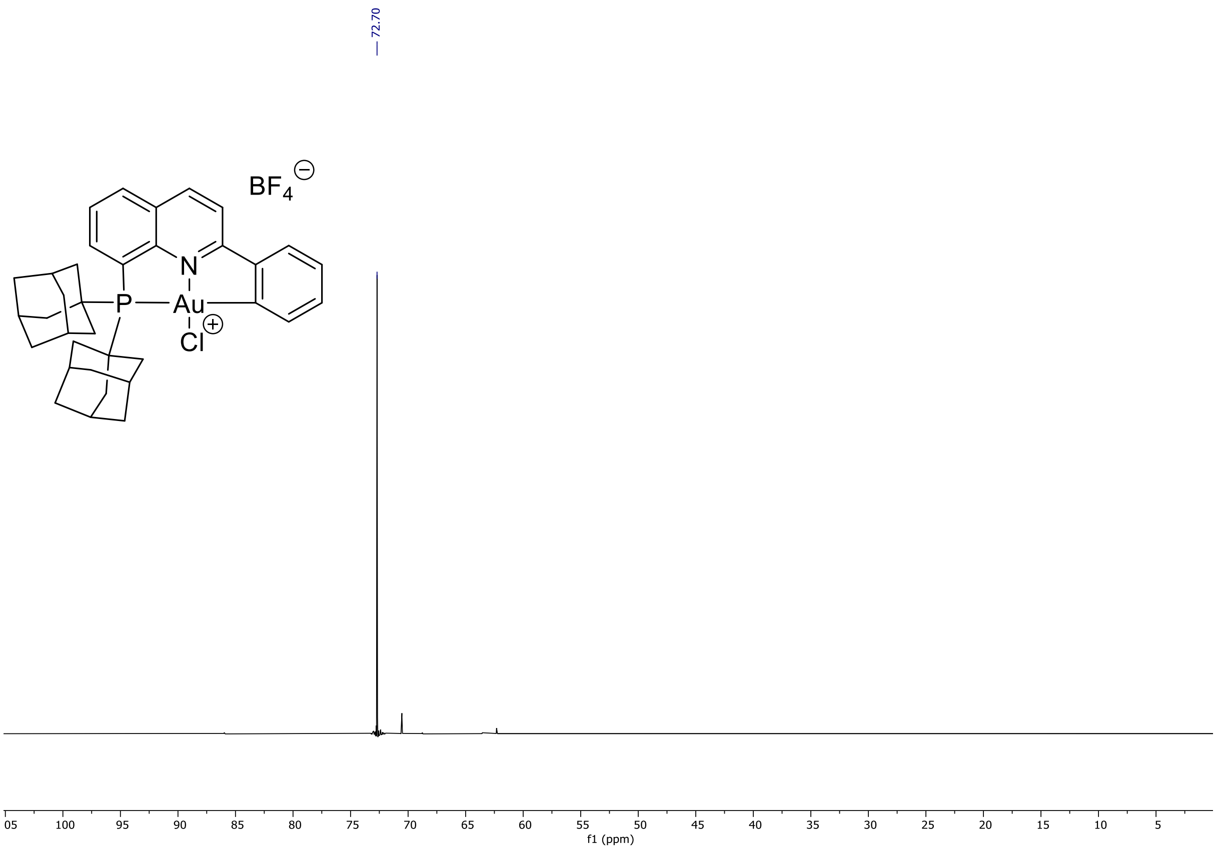

Figure S82. $^{31}P\{^{1}H\}$ NMR (202.52 MHz, CD_2Cl_2, 298 K) of compound **3**.

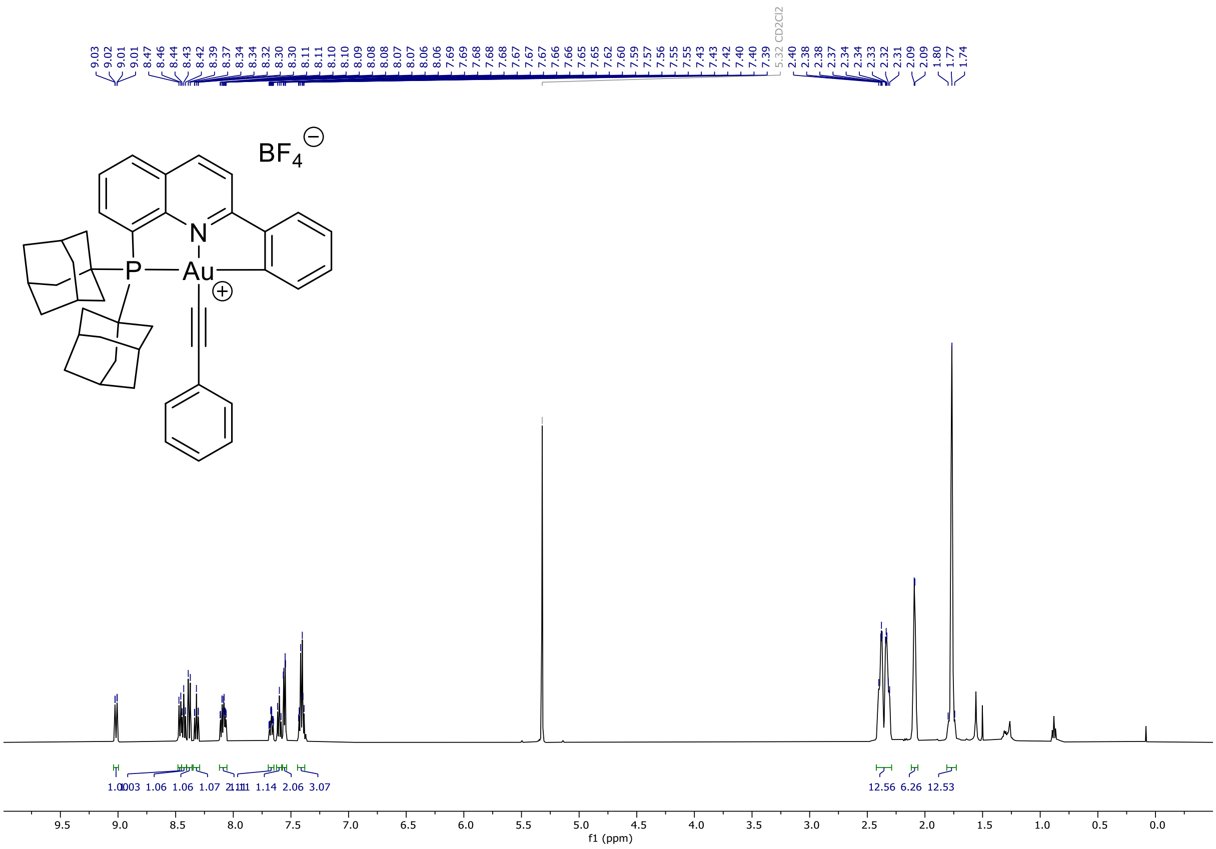

Figure S83. ^{1}H NMR (500.30 MHz, CD_2Cl_2, 298 K) of compound **4a**.

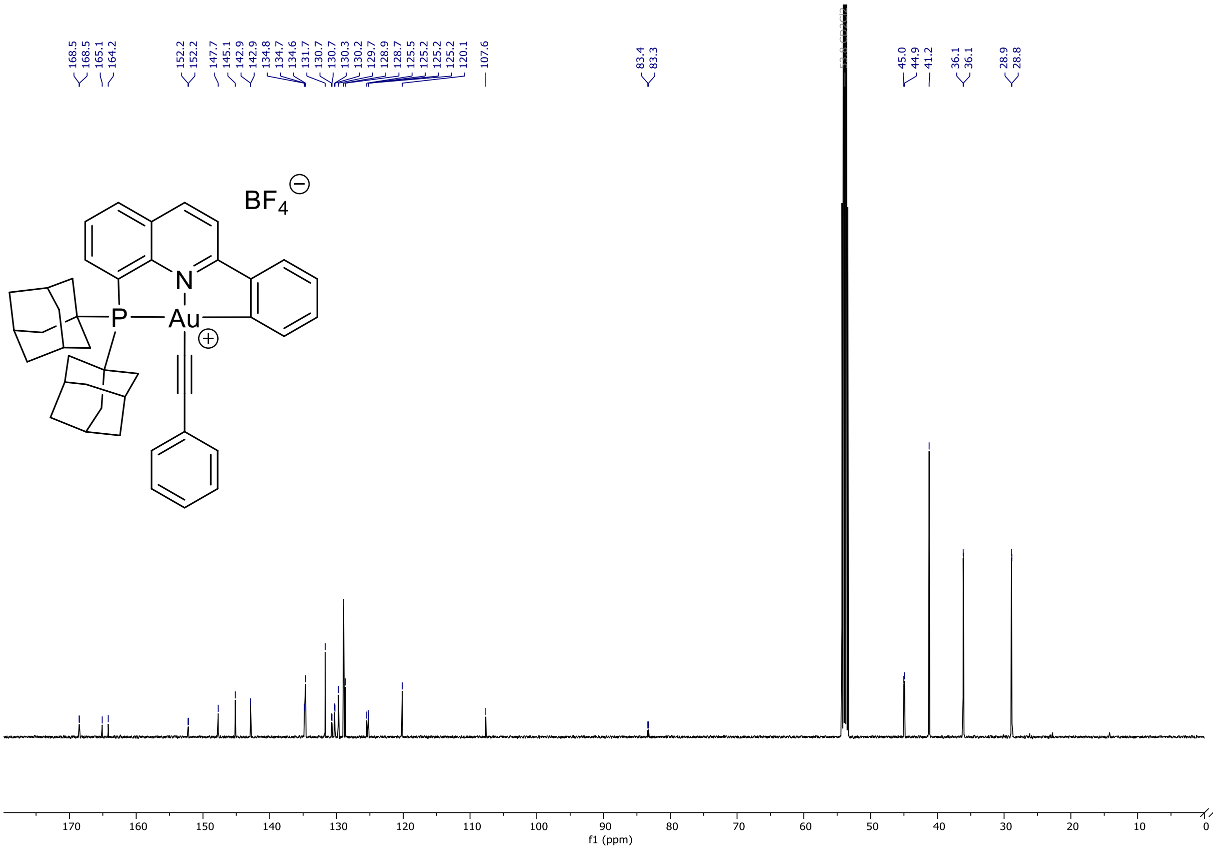

Figure S84. $^{13}C\{^{1}H\}$ NMR (125.81 MHz, CD_2Cl_2, 298 K) of compound **4a**.

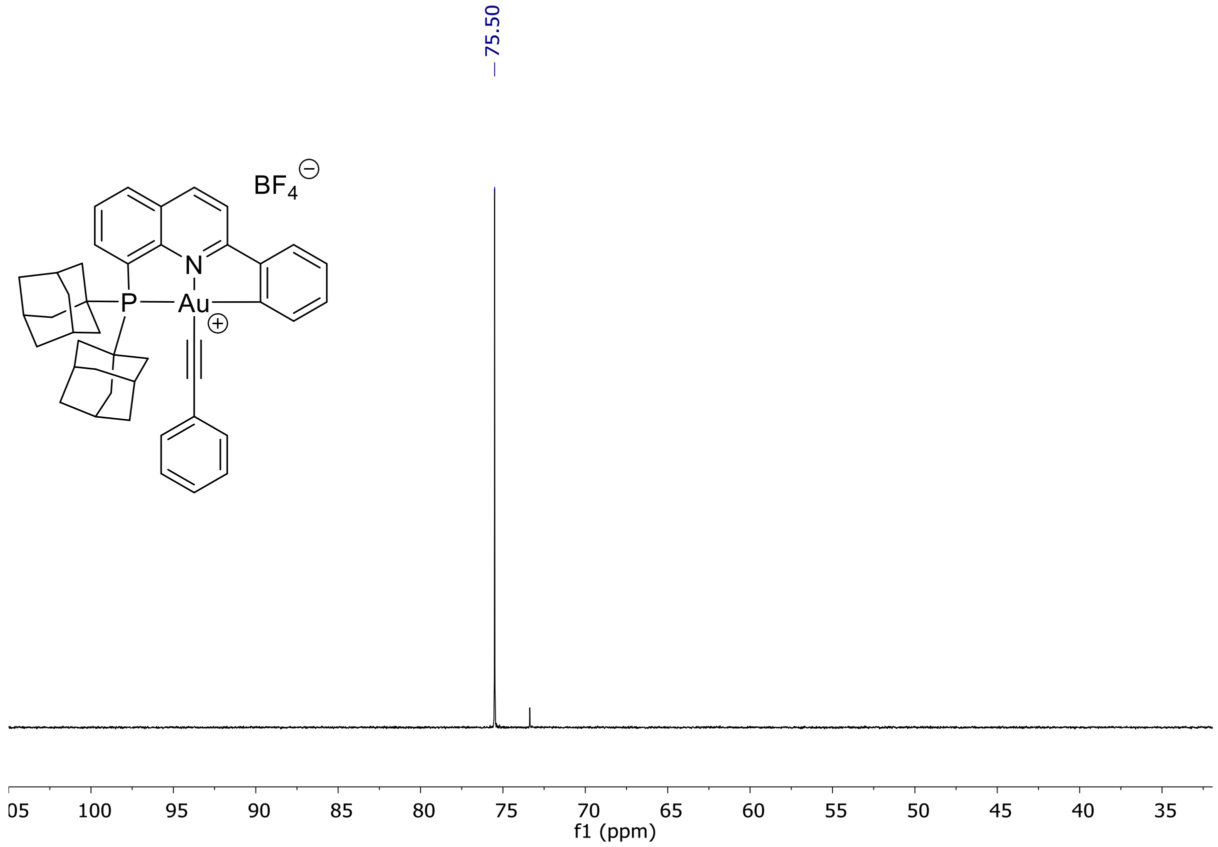

Figure S85. $^{31}P\{^{1}H\}$ NMR (202.52 MHz, CD_2Cl_2, 298 K) of compound **4a**.

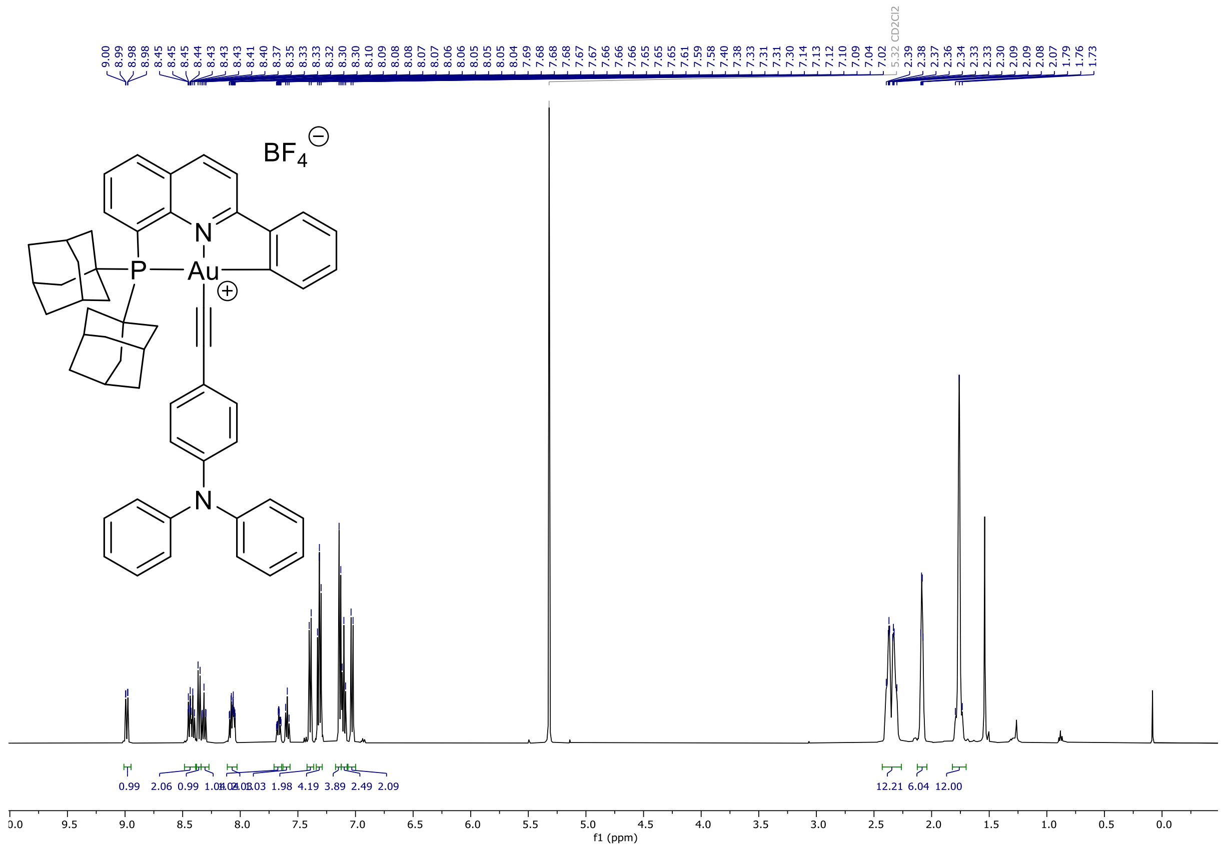

Figure S86. ^{1}H NMR (500.30 MHz, CD_2Cl_2, 298 K) of compound **4b**.

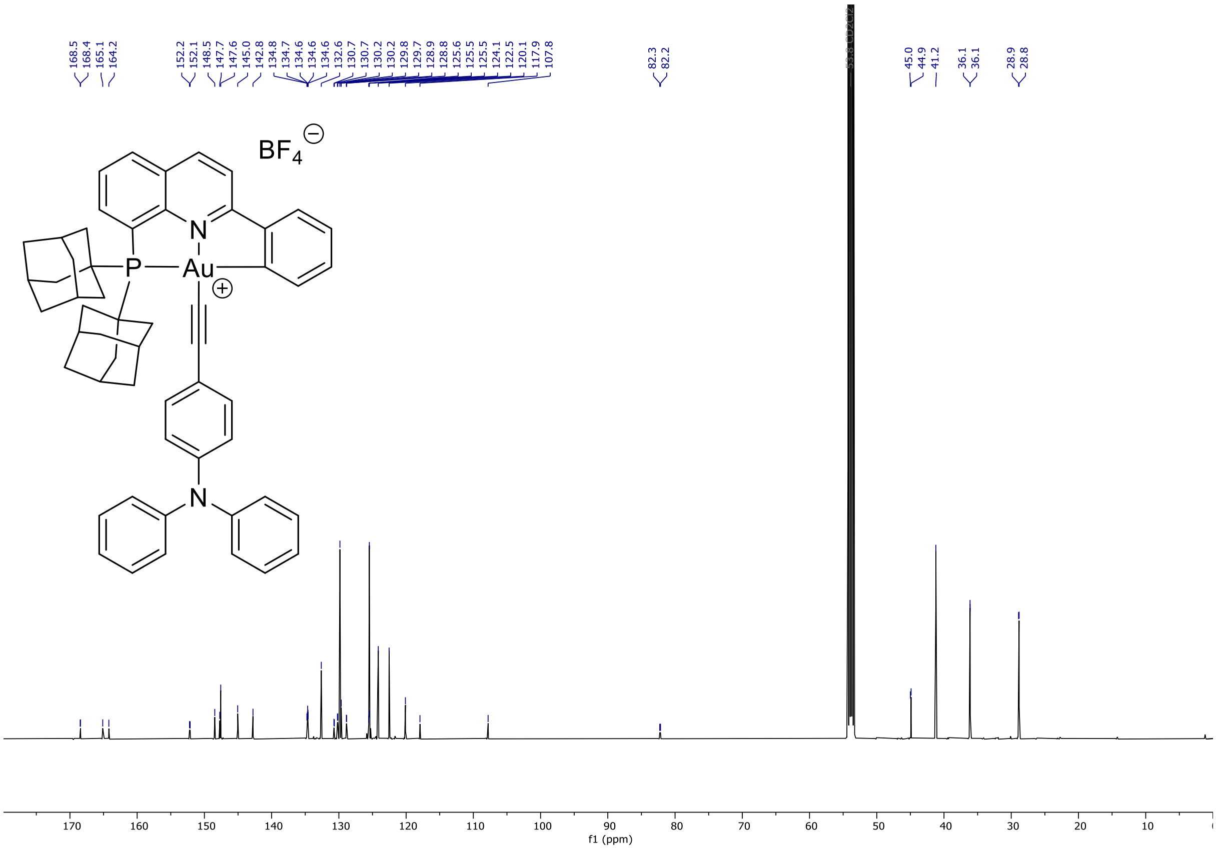

Figure S87. $^{13}C\{^{1}H\}$ NMR (125.81 MHz, CD_2Cl_2, 298 K) of compound **4b**.

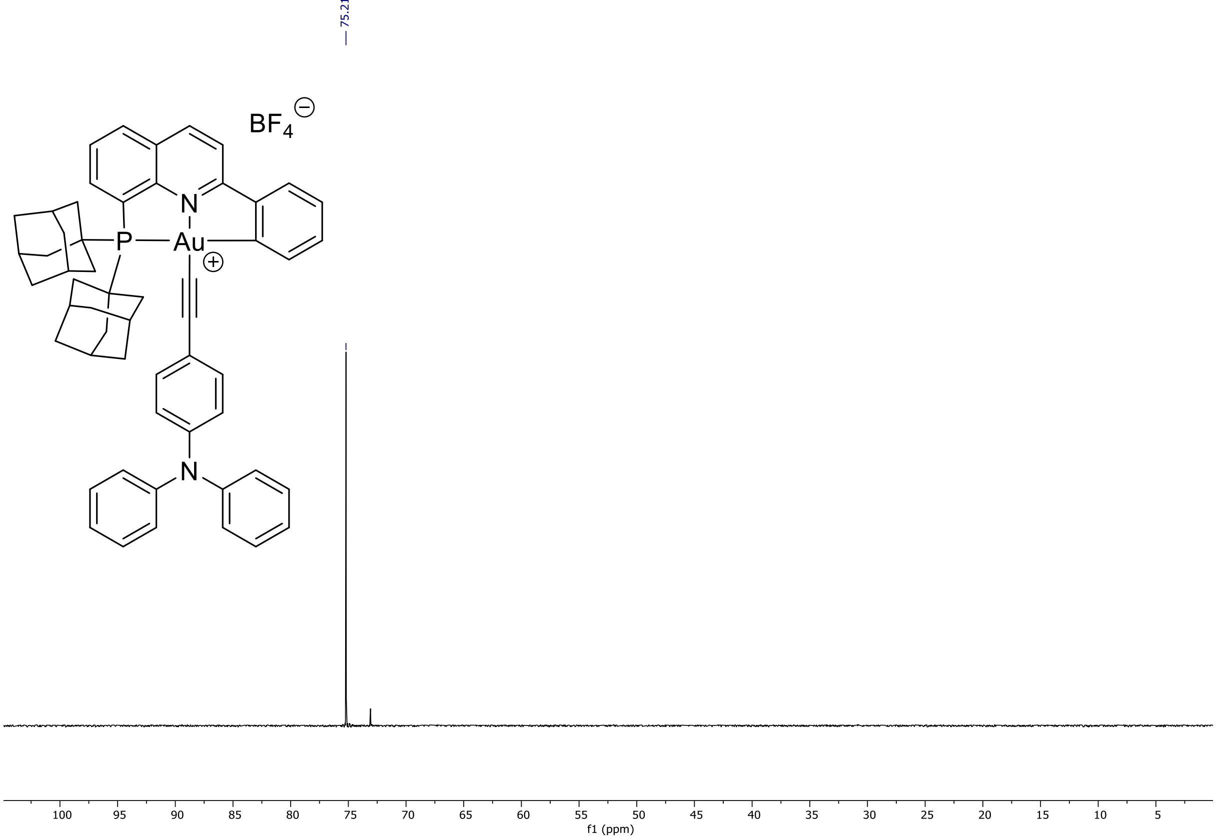

Figure S88. $^{31}P\{^{1}H\}$ NMR (202.52 MHz, CD_2Cl_2, 298 K) of compound **4b**.

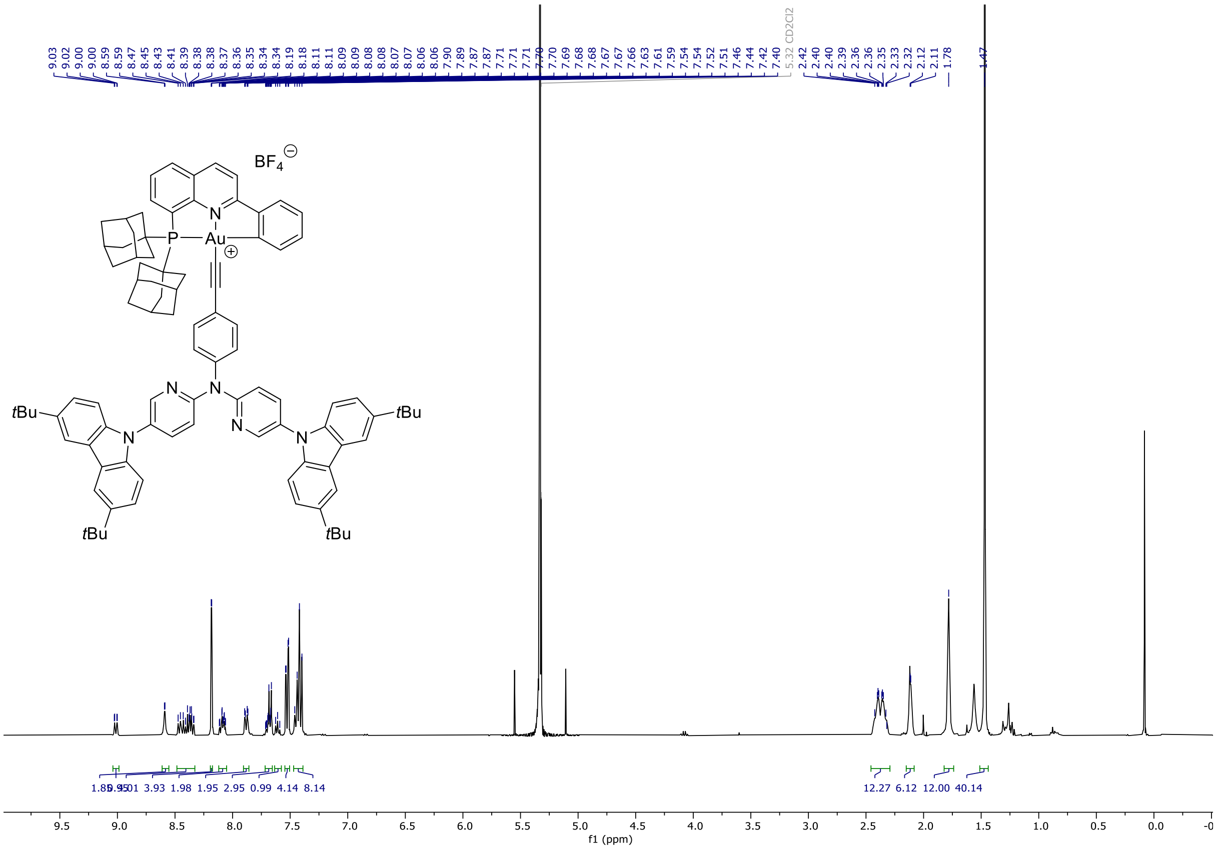

Figure S89. ^{1}H NMR (400.13 MHz, CD_2Cl_2, 298 K) of compound **4c**.

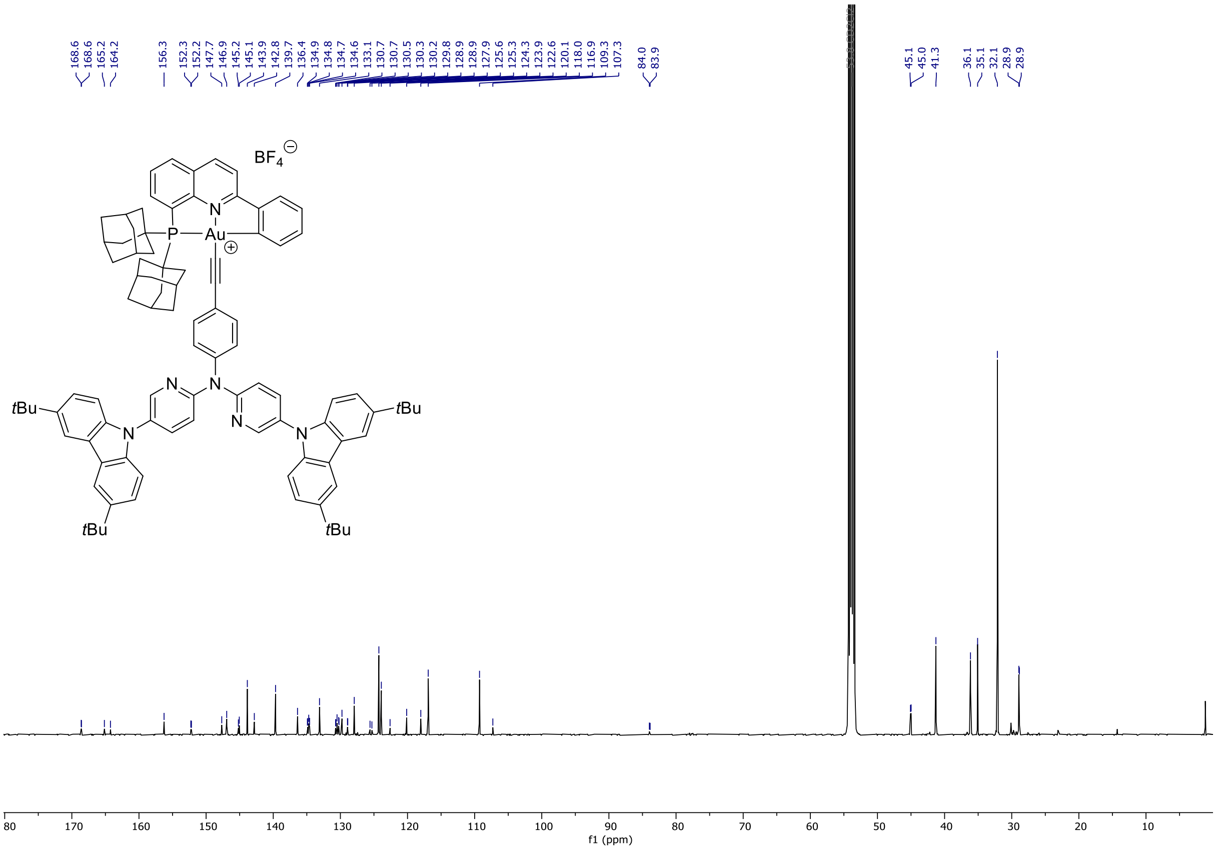

Figure S90. $^{13}C\{^{1}H\}$ NMR (125.81 MHz, CD_2Cl_2, 298 K) of compound **4c**.

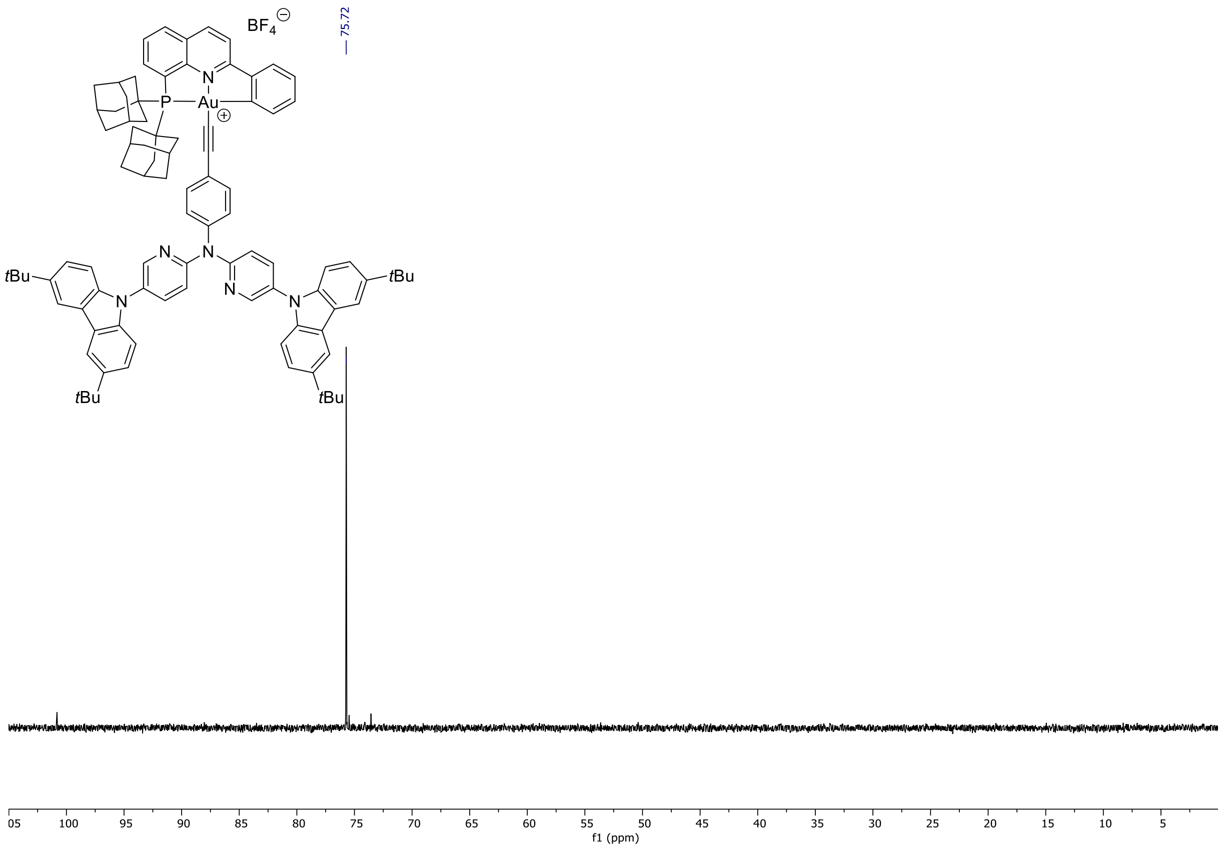

Figure S91. $^{31}P\{^{1}H\}$ NMR (202.52 MHz, CD_2Cl_2, 298 K) of compound **4c**.

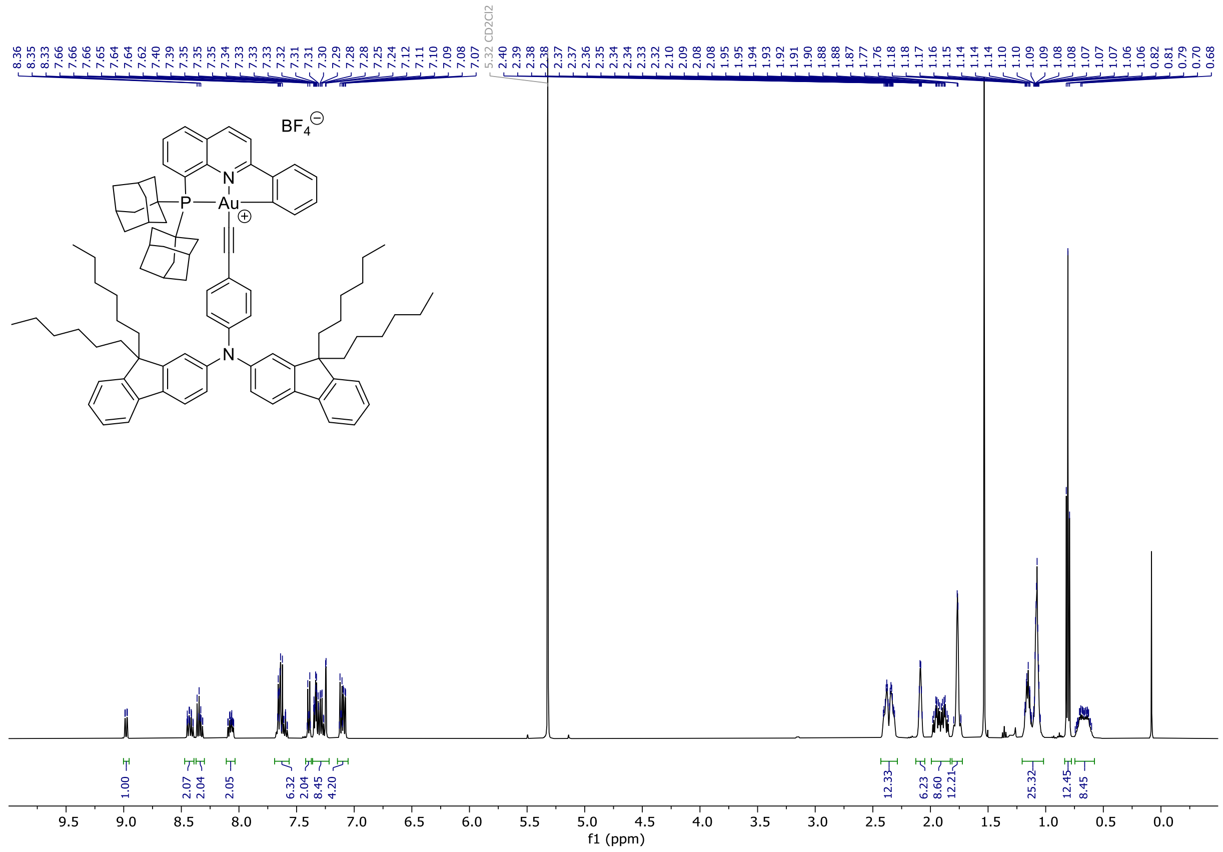

Figure S92. ^{1}H NMR (500.30 MHz, CD_2Cl_2, 298 K) of compound **4d**.

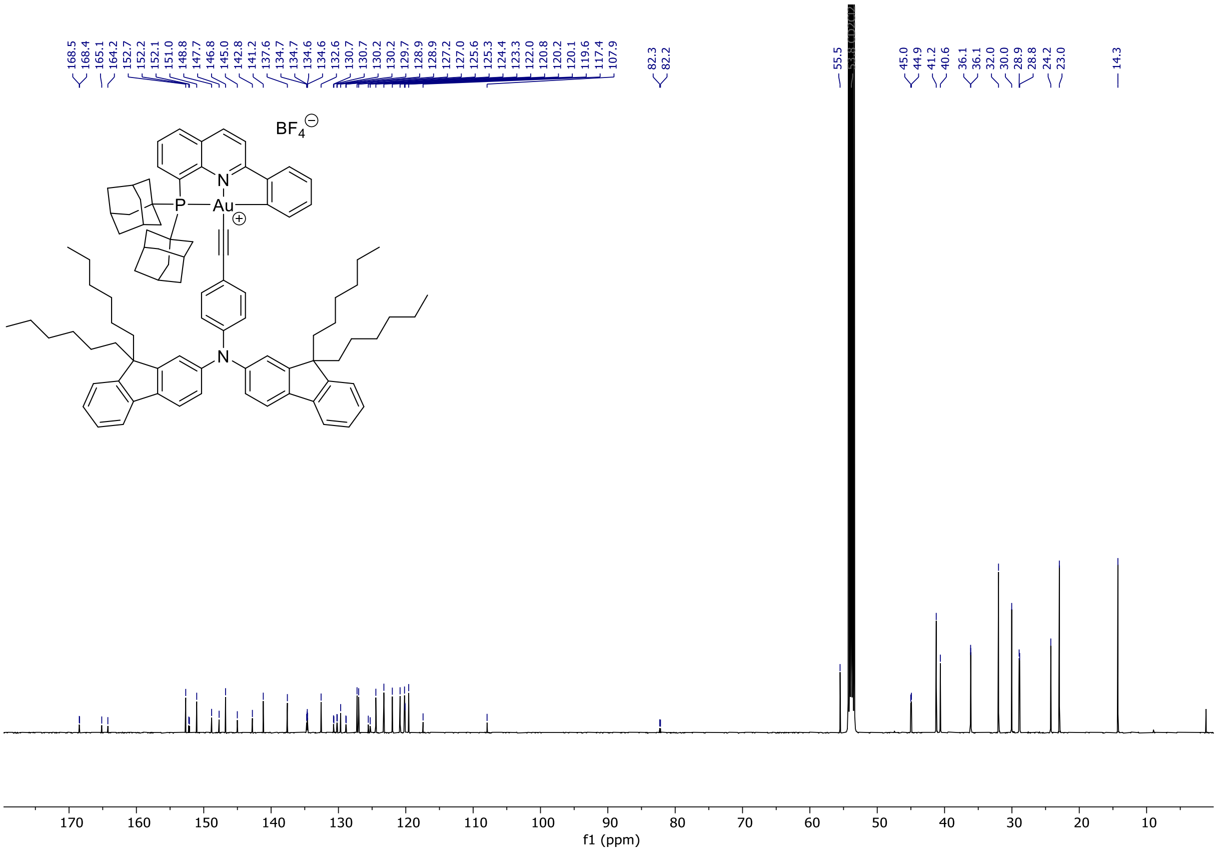

Figure S93. $^{13}C\{^{1}H\}$ NMR (125.81 MHz, CD_2Cl_2, 298 K) of compound **4d**.

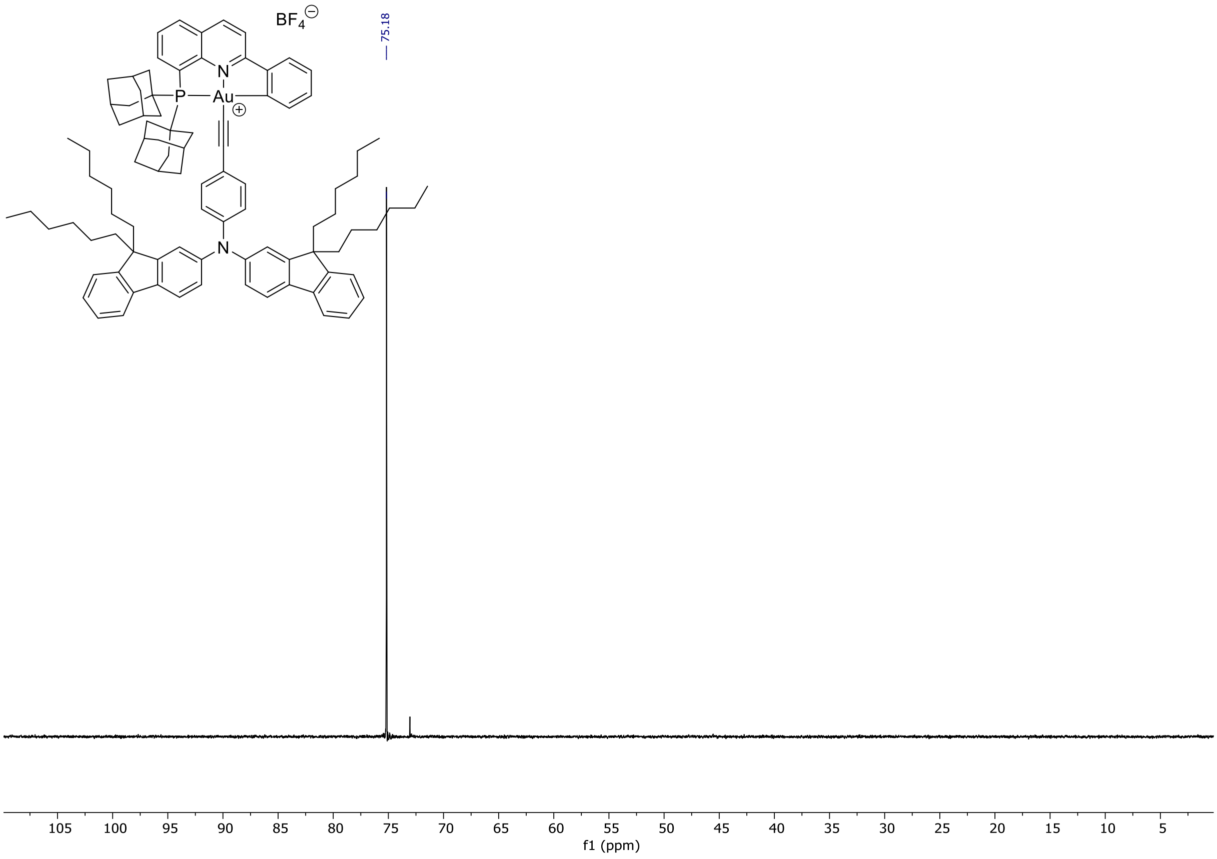

Figure S94. $^{31}P\{^{1}H\}$ NMR (202.52 MHz, CD_2Cl_2, 298 K) of compound **4d**.

7. X-ray diffraction analyses

All measurements were made on a *Rigaku Oxford Diffraction XtaLAB Synergy* diffractometer[11] with a *Pilatus 200K* hybrid pixel area detector using Mo $K\alpha$ radiation (λ = 0.71073 Å) for compound **1a** and Cu $K\alpha$ radiation (λ = 1.54184 Å) for compound **3** from a *PhotonJet* micro-focus X-ray source and an *Oxford Cryosystems Cryostream 800* cooler.

Data reduction was performed with *CrysAlisPro.*[11] The intensities were corrected for Lorentz and polarization effects, and an analytical absorption correction[12] was applied. The space group was uniquely determined by the systematic absences. Equivalent reflections were merged. The data collection and refinement parameters are given in Table S2 and Table S6.

The structure **1a** was solved by direct methods using *SHELXS-2013*,[13a] which revealed the positions of all non-hydrogen atoms. The SbF_6^- anion is disordered. Two sets of positions were defined for all atoms of the anion. The site occupation factor of the major orientation of the anion refined to 0.612(8). Similarity restraints were applied all bond lengths and angles within the anion, as well as to the *cis*-related F···F distances. Neighbouring disordered atoms were restrained to have similar and, for the F-atoms, pseudo-isotropic, atomic displacement parameters. The non-hydrogen atoms were refined anisotropically. All of the H-atoms were placed in geometrically calculated positions and refined by using a riding model where each H-atom was assigned a fixed isotropic displacement parameter with a value equal to $1.2U_{eq}$ of its parent atom. The refinement of the structure was carried out on F^2 by using full-matrix least-squares procedures, which minimised the function $\Sigma w(F_o^2 - F_c^2)^2$. The weighting scheme was based on counting statistics and included a factor to downweight the intense reflections. Plots of $\Sigma w(F_o^2 - F_c^2)^2$ versus $F_c/F_c(\max)$ and resolution showed no unusual trends. A correction for secondary extinction was not applied.

The structure **3** was solved by dual space methods using *SHELXT-2018*,[13b] which revealed the positions of all non-hydrogen atoms. There are two symmetry-independent cations and anions in the asymmetric unit. The atomic coordinates were tested carefully for a relationship from a higher symmetry space group using the program *PLATON*,[14] but none could be found. The anions are disordered. Two sets of positions were defined for all atoms of each anion. The site occupation factors of the major orientation of the anions refined to 0.752(3) and 0.509(4). Similarity restraints were applied all bond lengths and angles within each anion, as well as to the F···F distances. Neighbouring disordered atoms were restrained to have similar and, for the B-atoms and F6, pseudo-isotropic, atomic displacement parameters. The non-hydrogen atoms were refined anisotropically. All of the H-atoms were placed in geometrically calculated positions and refined by using a riding model where each H-atom was assigned a fixed isotropic displacement parameter with a value equal to $1.2U_{eq}$ of its parent atom. The refinement of the structure was carried out on F^2 by using full-matrix least-squares procedures, which minimised the function $\Sigma w(F_o^2 - F_c^2)^2$. The weighting scheme was based on counting statistics and included a factor to downweight the intense reflections. Plots of $\Sigma w(F_o^2 - F_c^2)^2$ versus $F_c/F_c(\max)$ and resolution showed no unusual trends. A correction for secondary

extinction was applied. Two reflections, whose intensities were considered to be extreme outliers, were omitted from the final refinement.

Neutral atom scattering factors for non-hydrogen atoms were taken from Maslen, Fox and O'Keefe,[15a] and the scattering factors for H-atoms were taken from Stewart, Davidson and Simpson.[16] Anomalous dispersion effects were included in F_c,[17] the values for f' and f'' were those of Creagh and McAuley.[15b] The values of the mass attenuation coefficients are those of Creagh and Hubbel.[15c] The *SHELXL-2018* program[18] was used for all calculations.

^{Ad}P^N^C^{H}Au(4-F-C_6H_4). (1a) (CCDC 2451717)

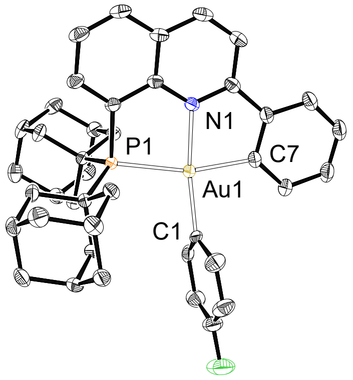

ORTEP representation of **1a** with 50% probability ellipsoids. **Note:** The structure of $C_{41}H_{43}AuFNP^+$ SbF_6^- (**1a**) has been solved and refined successfully. The SbF_6^- anion is disordered.

Crystal-structure determination. A crystal of obtained by slow vapor diffusion of hexane into a solution of the compound in dichloroethane at low temperature, was mounted on a glass fibre and used for a low-temperature (160 K) X-ray structure determination. The unit cell constants and an orientation matrix for data collection were obtained from a least-squares refinement of the setting angles of 29890 reflections in the range 6° < 2Θ < 67°. A total of 884 frames were collected using ω scans with κ offsets, 20.0 seconds exposure time and a rotation angle of 0.5° per frame, and a crystal-detector distance of 35.0 mm.

Table S2. Crystallographic data of **1a.**

Crystallised from	dichloroethane / hexane
Empirical formula	$C_{41}H_{43}AuF_7NPSb$
Formula weight [g mol^{-1}]	1032.45
Crystal colour, habit	colourless, needle
Crystal dimensions [mm]	0.04 × 0.07 × 0.20
Temperature [K]	160(1)
Crystal system	monoclinic
Space group	*P*21/*n* (#14)

Z		4
Reflections for cell determination		29890
2θ range for cell determination [°]		6 – 67
Unit cell parameters	a [Å]	10.43709(19)
	b [Å]	21.5788(4)
	c [Å]	16.1757(3)
	α [°]	90
	β [°]	100.7621(17)
	γ [°]	90
	V [Å3]	3579.01(12)
$F(000)$		2016
D_x [g cm-3]		1.916
μ(Mo Kα) [mm-1]		4.963
Scan type		ω
2θ(max) [°]		61.0
Transmission factors (min; max)		0.474; 0.852
Total reflections measured		57683
Symmetry independent reflections		10898
R_{int}		0.033
Reflections with $I > 2\sigma(I)$		9544
Reflections used in refinement		10898
Parameters refined; restraints		533; 459
Final	$R(F)$ [$I > 2\sigma(I)$ reflections]	0.0277
	$wR(F^2)$ (all data)	0.0650
Weights:		$w = [\sigma^2(Fo^2) + (0.0260P)^2 + 8.3140P]^{-1}$ where $P = (Fo^2 + 2Fc^2)/3$
Goodness of fit		1.029
Final Δmax/σ		0.002
$\Delta\rho$ (max; min) [e Å-3]		1.81; -1.72
$\sigma(d_{(C-C)})$ [Å]		0.004 – 0.006

Table S3. Bond lengths (Å) with standard uncertainties in parentheses.

Au(1)-C(1)	2.049(3)	C(22)-C(27)	1.553(4)
Au(1)-C(7)	2.057(3)	C(23)-C(24)	1.542(4)
Au(1)-N(1)	2.071(2)	C(24)-C(28)	1.522(5)
Au(1)-P(1)	2.3932(7)	C(24)-C(25)	1.535(5)
P(1)-C(18)	1.842(3)	C(25)-C(26)	1.536(5)
P(1)-C(22)	1.864(3)	C(26)-C(31)	1.530(5)
P(1)-C(32)	1.868(3)	C(26)-C(27)	1.531(4)
F(1)-C(4)	1.360(4)	C(28)-C(29)	1.531(4)
N(1)-C(13)	1.339(4)	C(29)-C(31)	1.530(4)
N(1)-C(17)	1.361(4)	C(29)-C(30)	1.540(4)
C(1)-C(2)	1.265(5)	C(32)-C(41)	1.542(4)
C(1)-C(6)	1.424(5)	C(32)-C(37)	1.548(4)

C(2)-C(3)	1.448(5)	C(32)-C(33)	1.552(4)
C(3)-C(4)	1.362(5)	C(33)-C(34)	1.533(4)
C(4)-C(5)	1.381(6)	C(34)-C(35)	1.526(4)
C(5)-C(6)	1.389(5)	C(34)-C(38)	1.530(4)
C(7)-C(8)	1.387(4)	C(35)-C(36)	1.529(5)
C(7)-C(12)	1.410(4)	C(36)-C(40)	1.526(5)
C(8)-C(9)	1.397(4)	C(36)-C(37)	1.544(4)
C(9)-C(10)	1.377(5)	C(38)-C(39)	1.531(4)
C(10)-C(11)	1.379(5)	C(39)-C(40)	1.539(5)
C(11)-C(12)	1.401(4)	C(39)-C(41)	1.544(4)
C(12)-C(13)	1.468(4)	Sb(1)-F(4)	1.848(3)
C(13)-C(14)	1.415(4)	Sb(1)-F(5)	1.855(3)
C(14)-C(15)	1.355(5)	Sb(1)-F(3)	1.857(3)
C(15)-C(16)	1.418(4)	Sb(1)-F(2)	1.860(3)
C(16)-C(21)	1.405(4)	Sb(1)-F(7)	1.865(3)
C(16)-C(17)	1.416(4)	Sb(1)-F(6)	1.865(3)
C(17)-C(18)	1.427(4)	Sb(1a)-F(5a)	1.840(3)
C(18)-C(19)	1.382(4)	Sb(1a)-F(7a)	1.841(3)
C(19)-C(20)	1.410(4)	Sb(1a)-F(6a)	1.857(3)
C(20)-C(21)	1.365(5)	Sb(1a)-F(3a)	1.863(3)
C(22)-C(23)	1.546(4)	Sb(1a)-F(2a)	1.873(3)
C(22)-C(30)	1.552(4)	Sb(1a)-F(4a)	1.875(3)

Table S4. Bond angles (°) with standard uncertainties in parentheses.

C(1)-Au(1)-C(7)	92.17(11)	C(24)-C(25)-C(26)	109.3(3)
C(1)-Au(1)-N(1)	171.07(11)	C(31)-C(26)-C(27)	110.0(3)
C(7)-Au(1)-N(1)	80.87(11)	C(31)-C(26)-C(25)	109.7(3)
C(1)-Au(1)-P(1)	103.56(8)	C(27)-C(26)-C(25)	109.0(3)
C(7)-Au(1)-P(1)	164.15(8)	C(26)-C(27)-C(22)	110.1(2)
N(1)-Au(1)-P(1)	83.66(7)	C(24)-C(28)-C(29)	109.3(2)
C(18)-P(1)-C(22)	107.94(12)	C(31)-C(29)-C(28)	109.7(3)
C(18)-P(1)-C(32)	104.78(13)	C(31)-C(29)-C(30)	109.7(2)
C(22)-P(1)-C(32)	117.23(12)	C(28)-C(29)-C(30)	109.8(2)
C(18)-P(1)-Au(1)	96.92(9)	C(29)-C(30)-C(22)	109.5(2)
C(22)-P(1)-Au(1)	116.44(9)	C(29)-C(31)-C(26)	109.4(2)
C(32)-P(1)-Au(1)	110.74(9)	C(41)-C(32)-C(37)	109.2(2)
C(13)-N(1)-C(17)	122.8(2)	C(41)-C(32)-C(33)	109.1(2)
C(13)-N(1)-Au(1)	115.39(19)	C(37)-C(32)-C(33)	108.0(2)
C(17)-N(1)-Au(1)	121.56(18)	C(41)-C(32)-P(1)	111.21(19)
C(2)-C(1)-C(6)	120.9(3)	C(37)-C(32)-P(1)	113.8(2)
C(2)-C(1)-Au(1)	125.8(3)	C(33)-C(32)-P(1)	105.35(19)
C(6)-C(1)-Au(1)	113.1(3)	C(34)-C(33)-C(32)	110.2(2)
C(1)-C(2)-C(3)	122.1(3)	C(35)-C(34)-C(38)	109.8(3)
C(4)-C(3)-C(2)	116.8(3)	C(35)-C(34)-C(33)	110.0(2)
F(1)-C(4)-C(3)	118.5(4)	C(38)-C(34)-C(33)	109.1(2)

F(1)-C(4)-C(5)	118.7(3)	C(34)-C(35)-C(36)	109.4(2)
C(3)-C(4)-C(5)	122.8(3)	C(40)-C(36)-C(35)	109.7(3)
C(4)-C(5)-C(6)	117.7(3)	C(40)-C(36)-C(37)	109.6(3)
C(5)-C(6)-C(1)	119.7(3)	C(35)-C(36)-C(37)	109.7(3)
C(8)-C(7)-C(12)	119.5(3)	C(36)-C(37)-C(32)	109.5(2)
C(8)-C(7)-Au(1)	128.9(2)	C(34)-C(38)-C(39)	109.0(2)
C(12)-C(7)-Au(1)	111.6(2)	C(38)-C(39)-C(40)	110.3(3)
C(7)-C(8)-C(9)	119.6(3)	C(38)-C(39)-C(41)	110.2(3)
C(10)-C(9)-C(8)	121.0(3)	C(40)-C(39)-C(41)	108.6(3)
C(9)-C(10)-C(11)	120.0(3)	C(36)-C(40)-C(39)	109.2(3)
C(10)-C(11)-C(12)	120.2(3)	C(32)-C(41)-C(39)	109.5(2)
C(11)-C(12)-C(7)	119.7(3)	F(4)-Sb(1)-F(5)	91.08(15)
C(11)-C(12)-C(13)	122.9(3)	F(4)-Sb(1)-F(3)	90.57(14)
C(7)-C(12)-C(13)	117.5(2)	F(5)-Sb(1)-F(3)	89.55(14)
N(1)-C(13)-C(14)	119.3(3)	F(4)-Sb(1)-F(2)	89.79(14)
N(1)-C(13)-C(12)	114.6(2)	F(5)-Sb(1)-F(2)	179.09(18)
C(14)-C(13)-C(12)	126.1(3)	F(3)-Sb(1)-F(2)	90.17(14)
C(15)-C(14)-C(13)	119.8(3)	F(4)-Sb(1)-F(7)	178.52(17)
C(14)-C(15)-C(16)	121.1(3)	F(5)-Sb(1)-F(7)	90.15(14)
C(21)-C(16)-C(17)	119.1(3)	F(3)-Sb(1)-F(7)	90.26(14)
C(21)-C(16)-C(15)	123.5(3)	F(2)-Sb(1)-F(7)	88.99(14)
C(17)-C(16)-C(15)	117.3(3)	F(4)-Sb(1)-F(6)	89.93(14)
N(1)-C(17)-C(16)	119.7(3)	F(5)-Sb(1)-F(6)	89.97(14)
N(1)-C(17)-C(18)	119.4(2)	F(3)-Sb(1)-F(6)	179.32(17)
C(16)-C(17)-C(18)	120.9(3)	F(2)-Sb(1)-F(6)	90.30(14)
C(19)-C(18)-C(17)	117.4(3)	F(7)-Sb(1)-F(6)	89.25(14)
C(19)-C(18)-P(1)	124.8(2)	F(5a)-Sb(1a)-F(7a)	91.35(17)
C(17)-C(18)-P(1)	117.7(2)	F(5a)-Sb(1a)-F(6a)	90.80(16)
C(18)-C(19)-C(20)	121.6(3)	F(7a)-Sb(1a)-F(6a)	90.89(17)
C(21)-C(20)-C(19)	120.8(3)	F(5a)-Sb(1a)-F(3a)	90.42(17)
C(20)-C(21)-C(16)	120.1(3)	F(7a)-Sb(1a)-F(3a)	90.70(16)
C(23)-C(22)-C(30)	108.4(2)	F(6a)-Sb(1a)-F(3a)	178.0(2)
C(23)-C(22)-C(27)	109.4(2)	F(5a)-Sb(1a)-F(2a)	178.9(2)
C(30)-C(22)-C(27)	108.7(2)	F(7a)-Sb(1a)-F(2a)	89.66(16)
C(23)-C(22)-P(1)	115.21(19)	F(6a)-Sb(1a)-F(2a)	89.61(16)
C(30)-C(22)-P(1)	109.16(19)	F(3a)-Sb(1a)-F(2a)	89.14(16)
C(27)-C(22)-P(1)	105.82(18)	F(5a)-Sb(1a)-F(4a)	90.36(17)
C(24)-C(23)-C(22)	109.2(2)	F(7a)-Sb(1a)-F(4a)	178.3(2)
C(28)-C(24)-C(25)	109.6(3)	F(6a)-Sb(1a)-F(4a)	89.23(16)
C(28)-C(24)-C(23)	109.7(3)	F(3a)-Sb(1a)-F(4a)	89.14(16)
C(25)-C(24)-C(23)	110.1(3)	F(2a)-Sb(1a)-F(4a)	88.62(16)

Table S5. Torsion angles (°) with standard uncertainties in parentheses.

C(6)-C(1)-C(2)-C(3)	1.5(5)	C(32)-P(1)-C(22)-C(27)	-168.6(2)
Au(1)-C(1)-C(2)-C(3)	176.8(2)	Au(1)-P(1)-C(22)-C(27)	56.9(2)
C(1)-C(2)-C(3)-C(4)	-0.4(5)	C(30)-C(22)-C(23)-C(24)	60.2(3)
C(2)-C(3)-C(4)-F(1)	-179.5(3)	C(27)-C(22)-C(23)-C(24)	-58.1(3)
C(2)-C(3)-C(4)-C(5)	-0.9(5)	P(1)-C(22)-C(23)-C(24)	-177.2(2)
F(1)-C(4)-C(5)-C(6)	179.5(3)	C(22)-C(23)-C(24)-C(28)	-61.3(3)
C(3)-C(4)-C(5)-C(6)	1.0(6)	C(22)-C(23)-C(24)-C(25)	59.4(3)
C(4)-C(5)-C(6)-C(1)	0.2(5)	C(28)-C(24)-C(25)-C(26)	59.9(3)
C(2)-C(1)-C(6)-C(5)	-1.5(5)	C(23)-C(24)-C(25)-C(26)	-60.9(3)
Au(1)-C(1)-C(6)-C(5)	-177.3(3)	C(24)-C(25)-C(26)-C(31)	-59.5(3)
C(12)-C(7)-C(8)-C(9)	-0.1(5)	C(24)-C(25)-C(26)-C(27)	60.9(3)
Au(1)-C(7)-C(8)-C(9)	177.1(2)	C(31)-C(26)-C(27)-C(22)	59.7(3)
C(7)-C(8)-C(9)-C(10)	1.2(5)	C(25)-C(26)-C(27)-C(22)	-60.5(3)
C(8)-C(9)-C(10)-C(11)	-0.5(5)	C(23)-C(22)-C(27)-C(26)	59.4(3)
C(9)-C(10)-C(11)-C(12)	-1.3(5)	C(30)-C(22)-C(27)-C(26)	-58.8(3)
C(10)-C(11)-C(12)-C(7)	2.3(5)	P(1)-C(22)-C(27)-C(26)	-175.9(2)
C(10)-C(11)-C(12)-C(13)	-178.5(3)	C(25)-C(24)-C(28)-C(29)	-60.3(3)
C(8)-C(7)-C(12)-C(11)	-1.6(4)	C(23)-C(24)-C(28)-C(29)	60.7(3)
Au(1)-C(7)-C(12)-C(11)	-179.2(2)	C(24)-C(28)-C(29)-C(31)	60.4(3)
C(8)-C(7)-C(12)-C(13)	179.2(3)	C(24)-C(28)-C(29)-C(30)	-60.2(3)
Au(1)-C(7)-C(12)-C(13)	1.5(3)	C(31)-C(29)-C(30)-C(22)	-60.5(3)
C(17)-N(1)-C(13)-C(14)	-2.3(4)	C(28)-C(29)-C(30)-C(22)	60.1(3)
Au(1)-N(1)-C(13)-C(14)	-176.2(2)	C(23)-C(22)-C(30)-C(29)	-59.9(3)
C(17)-N(1)-C(13)-C(12)	177.2(3)	C(27)-C(22)-C(30)-C(29)	59.0(3)
Au(1)-N(1)-C(13)-C(12)	3.2(3)	P(1)-C(22)-C(30)-C(29)	173.9(2)
C(11)-C(12)-C(13)-N(1)	177.6(3)	C(28)-C(29)-C(31)-C(26)	-60.0(3)
C(7)-C(12)-C(13)-N(1)	-3.2(4)	C(30)-C(29)-C(31)-C(26)	60.6(3)
C(11)-C(12)-C(13)-C(14)	-2.9(5)	C(27)-C(26)-C(31)-C(29)	-60.2(3)
C(7)-C(12)-C(13)-C(14)	176.2(3)	C(25)-C(26)-C(31)-C(29)	59.6(3)
N(1)-C(13)-C(14)-C(15)	1.7(5)	C(18)-P(1)-C(32)-C(41)	-173.4(2)
C(12)-C(13)-C(14)-C(15)	-177.7(3)	C(22)-P(1)-C(32)-C(41)	-53.8(2)
C(13)-C(14)-C(15)-C(16)	0.3(5)	Au(1)-P(1)-C(32)-C(41)	83.2(2)
C(14)-C(15)-C(16)-C(21)	176.4(3)	C(18)-P(1)-C(32)-C(37)	-49.6(2)
C(14)-C(15)-C(16)-C(17)	-1.8(5)	C(22)-P(1)-C(32)-C(37)	70.0(2)
C(13)-N(1)-C(17)-C(16)	0.8(4)	Au(1)-P(1)-C(32)-C(37)	-153.1(2)
Au(1)-N(1)-C(17)-C(16)	174.4(2)	C(18)-P(1)-C(32)-C(33)	68.6(2)
C(13)-N(1)-C(17)-C(18)	-178.3(3)	C(22)-P(1)-C(32)-C(33)	-171.8(2)
Au(1)-N(1)-C(17)-C(18)	-4.7(4)	Au(1)-P(1)-C(32)-C(33)	-34.9(2)
C(21)-C(16)-C(17)-N(1)	-177.0(3)	C(41)-C(32)-C(33)-C(34)	59.1(3)
C(15)-C(16)-C(17)-N(1)	1.2(4)	C(37)-C(32)-C(33)-C(34)	-59.5(3)
C(21)-C(16)-C(17)-C(18)	2.1(4)	P(1)-C(32)-C(33)-C(34)	178.5(2)
C(15)-C(16)-C(17)-C(18)	-179.7(3)	C(32)-C(33)-C(34)-C(35)	59.9(3)
N(1)-C(17)-C(18)-C(19)	177.4(3)	C(32)-C(33)-C(34)-C(38)	-60.6(3)
C(16)-C(17)-C(18)-C(19)	-1.7(4)	C(38)-C(34)-C(35)-C(36)	60.5(3)

N(1)-C(17)-C(18)-P(1)	-2.8(4)	C(33)-C(34)-C(35)-C(36)	-59.6(3)
C(16)-C(17)-C(18)-P(1)	178.1(2)	C(34)-C(35)-C(36)-C(40)	-60.4(3)
C(22)-P(1)-C(18)-C(19)	-52.5(3)	C(34)-C(35)-C(36)-C(37)	60.1(3)
C(32)-P(1)-C(18)-C(19)	73.2(3)	C(40)-C(36)-C(37)-C(32)	59.7(3)
Au(1)-P(1)-C(18)-C(19)	-173.2(3)	C(35)-C(36)-C(37)-C(32)	-60.9(3)
C(22)-P(1)-C(18)-C(17)	127.8(2)	C(41)-C(32)-C(37)-C(36)	-58.8(3)
C(32)-P(1)-C(18)-C(17)	-106.6(2)	C(33)-C(32)-C(37)-C(36)	59.7(3)
Au(1)-P(1)-C(18)-C(17)	7.1(2)	P(1)-C(32)-C(37)-C(36)	176.3(2)
C(17)-C(18)-C(19)-C(20)	-0.2(5)	C(35)-C(34)-C(38)-C(39)	-59.7(3)
P(1)-C(18)-C(19)-C(20)	-179.9(2)	C(33)-C(34)-C(38)-C(39)	61.0(3)
C(18)-C(19)-C(20)-C(21)	1.6(5)	C(34)-C(38)-C(39)-C(40)	59.0(3)
C(19)-C(20)-C(21)-C(16)	-1.2(5)	C(34)-C(38)-C(39)-C(41)	-60.9(3)
C(17)-C(16)-C(21)-C(20)	-0.6(5)	C(35)-C(36)-C(40)-C(39)	59.3(3)
C(15)-C(16)-C(21)-C(20)	-178.8(3)	C(37)-C(36)-C(40)-C(39)	-61.2(3)
C(18)-P(1)-C(22)-C(23)	70.3(2)	C(38)-C(39)-C(40)-C(36)	-59.1(3)
C(32)-P(1)-C(22)-C(23)	-47.6(3)	C(41)-C(39)-C(40)-C(36)	61.8(3)
Au(1)-P(1)-C(22)-C(23)	177.9(2)	C(37)-C(32)-C(41)-C(39)	60.0(3)
C(18)-P(1)-C(22)-C(30)	-167.5(2)	C(33)-C(32)-C(41)-C(39)	-57.8(3)
C(32)-P(1)-C(22)-C(30)	74.6(2)	P(1)-C(32)-C(41)-C(39)	-173.6(2)
Au(1)-P(1)-C(22)-C(30)	-59.9(2)	C(38)-C(39)-C(41)-C(32)	59.6(3)
C(18)-P(1)-C(22)-C(27)	-50.7(2)	C(40)-C(39)-C(41)-C(32)	-61.4(3)

^{Ad}P^N^C^{H}AuCl. (3) (CCDC 2451722)

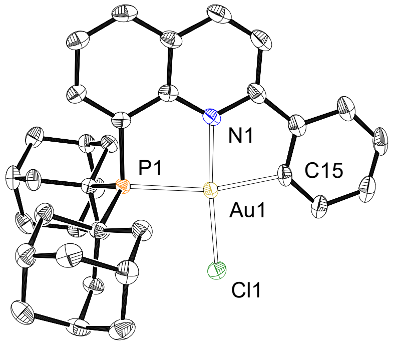

ORTEP representation of **3** with 50% probability ellipsoids. **Note:** The structure of $C_{35}H_{39}AuClNP^{+}$ BF_4^{-} (**3**) has been solved and refined successfully. There are two cations and two disordered anions in the asymmetric unit.

Crystal-structure determination. A crystal of obtained by slow vapor diffusion of hexane into a solution of the compound in dichloromethane at low temperature, was mounted on a glass fibre and used for a low-temperature (160 K) X-ray structure determination. The unit cell constants and an orientation matrix for data collection were obtained from a least-squares refinement of the setting angles of 50207 reflections in the range 7° < 2Θ < 157°. A total of 5650 frames were collected using ω scans with κ offsets, 2.0-8.0 seconds exposure time and a rotation angle of 0.5° per frame, and a crystal-detector distance of 35.0 mm.

Table S6. Crystallographic data of **3.**

Crystallised from		dichloromethane / hexane
Empirical formula		$C_{35}H_{39}AuBClF_4NP$
Formula weight [g mol^{-1}]		823.87
Crystal colour, habit		colourless, plate
Crystal dimensions [mm]		0.03 × 0.06 × 0.09
Temperature [K]		160(1)
Crystal system		triclinic
Space group		$P\bar{1}$ (#2)
Z		4
Reflections for cell determination		50207
2θ range for cell determination [°]		7 – 157
Unit cell parameters	a [Å]	13.77521(16)
	b [Å]	15.6910(2)
	c [Å]	15.82482(14)
	α [°]	81.8253(9)
	β [°]	79.9527(9)
	γ [°]	66.5687(12)
	V [Å3]	3080.23(7)
F(000)		1632

D_x [g cm-3]	1.777
μ(Mo Kα) [mm-1]	10.700
Scan type	ω
2θ(max) [°]	149.0
Transmission factors (min; max)	0.498; 0.784
Total reflections measured	65679
Symmetry independent reflections	12599
R_{int}	0.025
Reflections with $I > 2\sigma(I)$	11939
Reflections used in refinement	12597
Parameters refined; restraints	886; 564
Final $R(F)$ [$I > 2\sigma(I)$ reflections]	0.0254
$wR(F^2)$ (all data)	0.0668
Weights:	$w = [\sigma^2(F_o^2) + (0.0396P)^2 + 5.4817P]^{-1}$ where $P = (F_o^2 + 2F_c^2)/3$
Goodness of fit	1.029
Secondary extinction coefficient	0.00007(1)
Final Δmax/σ	0.003
$\Delta\rho$ (max; min) [e Å-3]	1.31; -1.35
$\sigma(d_{(C-C)})$ [Å]	0.004 – 0.006

Table S7. Bond lengths (Å) with standard uncertainties in parentheses.

Au(1)-N(1)	2.012(2)	N(2)-C(41)	1.364(4)
Au(1)-C(15)	2.066(3)	C(36)-C(37)	1.382(4)
Au(1)-Cl(1)	2.2744(8)	C(36)-C(41)	1.424(4)
Au(1)-P(1)	2.3788(7)	C(37)-C(38)	1.408(4)
P(1)-C(1)	1.822(3)	C(38)-C(39)	1.361(5)
P(1)-C(26)	1.862(3)	C(39)-C(40)	1.415(4)
P(1)-C(16)	1.862(3)	C(40)-C(41)	1.408(4)
N(1)-C(9)	1.346(4)	C(40)-C(42)	1.408(4)
N(1)-C(6)	1.365(4)	C(42)-C(43)	1.365(5)
C(1)-C(2)	1.387(4)	C(43)-C(44)	1.405(4)
C(1)-C(6)	1.413(4)	C(44)-C(45)	1.458(4)
C(2)-C(3)	1.401(4)	C(45)-C(50)	1.399(4)
C(3)-C(4)	1.366(5)	C(45)-C(46)	1.402(4)
C(4)-C(5)	1.417(5)	C(46)-C(47)	1.389(5)
C(5)-C(7)	1.405(5)	C(47)-C(48)	1.384(5)
C(5)-C(6)	1.413(4)	C(48)-C(49)	1.398(4)
C(7)-C(8)	1.363(5)	C(49)-C(50)	1.386(4)
C(8)-C(9)	1.409(5)	C(51)-C(60)	1.537(4)
C(9)-C(10)	1.465(5)	C(51)-C(52)	1.539(4)
C(10)-C(11)	1.396(4)	C(51)-C(56)	1.544(4)
C(10)-C(15)	1.402(5)	C(52)-C(53)	1.550(4)
C(11)-C(12)	1.382(6)	C(53)-C(57)	1.521(6)

C(12)-C(13)	1.376(6)	C(53)-C(54)	1.523(6)
C(13)-C(14)	1.396(5)	C(54)-C(55)	1.529(5)
C(14)-C(15)	1.382(5)	C(55)-C(59)	1.529(5)
C(16)-C(17)	1.539(4)	C(55)-C(56)	1.539(4)
C(16)-C(21)	1.542(4)	C(57)-C(58)	1.523(6)
C(16)-C(24)	1.546(4)	C(58)-C(59)	1.517(5)
C(17)-C(18)	1.542(4)	C(58)-C(60)	1.551(4)
C(18)-C(19)	1.529(5)	C(61)-C(62)	1.536(4)
C(18)-C(22)	1.530(5)	C(61)-C(70)	1.541(4)
C(19)-C(20)	1.518(5)	C(61)-C(66)	1.553(4)
C(20)-C(25)	1.536(5)	C(62)-C(63)	1.546(4)
C(20)-C(21)	1.539(4)	C(63)-C(69)	1.522(5)
C(22)-C(23)	1.521(5)	C(63)-C(64)	1.531(5)
C(23)-C(25)	1.525(5)	C(64)-C(65)	1.533(5)
C(23)-C(24)	1.547(4)	C(65)-C(67)	1.527(5)
C(26)-C(31)	1.539(4)	C(65)-C(66)	1.535(4)
C(26)-C(35)	1.542(4)	C(67)-C(68)	1.530(5)
C(26)-C(27)	1.548(4)	C(68)-C(69)	1.533(5)
C(27)-C(28)	1.539(4)	C(68)-C(70)	1.542(4)
C(28)-C(32)	1.523(5)	F(1a)-B(1a)	1.385(3)
C(28)-C(29)	1.534(4)	F(2a)-B(1a)	1.390(3)
C(29)-C(30)	1.532(5)	F(3a)-B(1a)	1.385(3)
C(30)-C(34)	1.527(5)	F(4a)-B(1a)	1.382(3)
C(30)-C(31)	1.539(4)	F(1b)-B(1b)	1.384(4)
C(32)-C(33)	1.532(5)	F(2b)-B(1b)	1.385(4)
C(33)-C(35)	1.529(4)	F(3b)-B(1b)	1.384(4)
C(33)-C(34)	1.538(5)	F(4b)-B(1b)	1.384(4)
Au(2)-N(2)	2.007(2)	F(5a)-B(2a)	1.389(3)
Au(2)-C(50)	2.061(3)	F(6a)-B(2a)	1.388(4)
Au(2)-Cl(2)	2.2653(7)	F(7a)-B(2a)	1.384(3)
Au(2)-P(2)	2.3757(7)	F(8a)-B(2a)	1.378(3)
P(2)-C(36)	1.828(3)	F(5b)-B(2b)	1.381(3)
P(2)-C(51)	1.868(3)	F(6b)-B(2b)	1.384(3)
P(2)-C(61)	1.868(3)	F(7b)-B(2b)	1.372(3)
N(2)-C(44)	1.349(4)	F(8b)-B(2b)	1.394(3)

Table S8. Bond angles (°) with standard uncertainties in parentheses.

N(1)-Au(1)-C(15)	81.69(12)	C(44)-N(2)-C(41)	123.0(2)
N(1)-Au(1)-Cl(1)	174.71(8)	C(44)-N(2)-Au(2)	116.0(2)
C(15)-Au(1)-Cl(1)	93.58(10)	C(41)-N(2)-Au(2)	120.93(19)
N(1)-Au(1)-P(1)	84.56(8)	C(37)-C(36)-C(41)	118.1(3)
C(15)-Au(1)-P(1)	166.14(10)	C(37)-C(36)-P(2)	125.3(2)
Cl(1)-Au(1)-P(1)	100.22(3)	C(41)-C(36)-P(2)	116.6(2)
C(1)-P(1)-C(26)	106.87(13)	C(36)-C(37)-C(38)	121.4(3)

C(1)-P(1)-C(16)	107.75(13)	C(39)-C(38)-C(37)	120.4(3)
C(26)-P(1)-C(16)	117.75(13)	C(38)-C(39)-C(40)	120.6(3)
C(1)-P(1)-Au(1)	96.81(10)	C(41)-C(40)-C(42)	118.5(3)
C(26)-P(1)-Au(1)	110.85(9)	C(41)-C(40)-C(39)	118.8(3)
C(16)-P(1)-Au(1)	114.35(10)	C(42)-C(40)-C(39)	122.7(3)
C(9)-N(1)-C(6)	123.2(3)	N(2)-C(41)-C(40)	118.7(3)
C(9)-N(1)-Au(1)	115.8(2)	N(2)-C(41)-C(36)	120.6(3)
C(6)-N(1)-Au(1)	121.0(2)	C(40)-C(41)-C(36)	120.7(3)
C(2)-C(1)-C(6)	118.5(3)	C(43)-C(42)-C(40)	121.0(3)
C(2)-C(1)-P(1)	124.6(2)	C(42)-C(43)-C(44)	119.3(3)
C(6)-C(1)-P(1)	116.9(2)	N(2)-C(44)-C(43)	119.4(3)
C(1)-C(2)-C(3)	121.0(3)	N(2)-C(44)-C(45)	114.5(3)
C(4)-C(3)-C(2)	120.8(3)	C(43)-C(44)-C(45)	126.1(3)
C(3)-C(4)-C(5)	120.3(3)	C(50)-C(45)-C(46)	120.0(3)
C(7)-C(5)-C(6)	118.4(3)	C(50)-C(45)-C(44)	117.1(3)
C(7)-C(5)-C(4)	123.0(3)	C(46)-C(45)-C(44)	122.9(3)
C(6)-C(5)-C(4)	118.6(3)	C(47)-C(46)-C(45)	119.0(3)
N(1)-C(6)-C(5)	118.6(3)	C(48)-C(47)-C(46)	120.4(3)
N(1)-C(6)-C(1)	120.6(3)	C(47)-C(48)-C(49)	121.2(3)
C(5)-C(6)-C(1)	120.8(3)	C(50)-C(49)-C(48)	118.4(3)
C(8)-C(7)-C(5)	121.2(3)	C(49)-C(50)-C(45)	120.9(3)
C(7)-C(8)-C(9)	119.3(3)	C(49)-C(50)-Au(2)	128.2(2)
N(1)-C(9)-C(8)	119.3(3)	C(45)-C(50)-Au(2)	110.9(2)
N(1)-C(9)-C(10)	114.8(3)	C(60)-C(51)-C(52)	108.8(3)
C(8)-C(9)-C(10)	125.9(3)	C(60)-C(51)-C(56)	108.7(3)
C(11)-C(10)-C(15)	119.4(3)	C(52)-C(51)-C(56)	109.5(3)
C(11)-C(10)-C(9)	123.6(3)	C(60)-C(51)-P(2)	114.4(2)
C(15)-C(10)-C(9)	117.0(3)	C(52)-C(51)-P(2)	110.4(2)
C(12)-C(11)-C(10)	119.4(3)	C(56)-C(51)-P(2)	104.93(19)
C(13)-C(12)-C(11)	120.8(3)	C(51)-C(52)-C(53)	109.8(3)
C(12)-C(13)-C(14)	120.9(3)	C(57)-C(53)-C(54)	110.0(3)
C(15)-C(14)-C(13)	118.5(3)	C(57)-C(53)-C(52)	108.4(3)
C(14)-C(15)-C(10)	121.1(3)	C(54)-C(53)-C(52)	110.1(3)
C(14)-C(15)-Au(1)	128.2(3)	C(53)-C(54)-C(55)	109.6(3)
C(10)-C(15)-Au(1)	110.7(2)	C(54)-C(55)-C(59)	109.6(3)
C(17)-C(16)-C(21)	109.4(2)	C(54)-C(55)-C(56)	109.1(3)
C(17)-C(16)-C(24)	109.2(2)	C(59)-C(55)-C(56)	109.3(3)
C(21)-C(16)-C(24)	108.7(2)	C(55)-C(56)-C(51)	109.9(2)
C(17)-C(16)-P(1)	109.52(19)	C(53)-C(57)-C(58)	109.6(3)
C(21)-C(16)-P(1)	105.5(2)	C(59)-C(58)-C(57)	109.6(3)
C(24)-C(16)-P(1)	114.4(2)	C(59)-C(58)-C(60)	108.8(3)
C(16)-C(17)-C(18)	109.4(2)	C(57)-C(58)-C(60)	110.6(3)
C(19)-C(18)-C(22)	109.6(3)	C(58)-C(59)-C(55)	109.9(3)
C(19)-C(18)-C(17)	109.9(3)	C(51)-C(60)-C(58)	109.1(3)
C(22)-C(18)-C(17)	109.0(3)	C(62)-C(61)-C(70)	109.5(2)

C(20)-C(19)-C(18)	110.1(3)	C(62)-C(61)-C(66)	109.1(2)
C(19)-C(20)-C(25)	109.7(3)	C(70)-C(61)-C(66)	108.4(2)
C(19)-C(20)-C(21)	109.0(3)	C(62)-C(61)-P(2)	113.7(2)
C(25)-C(20)-C(21)	109.2(3)	C(70)-C(61)-P(2)	110.07(19)
C(20)-C(21)-C(16)	109.9(3)	C(66)-C(61)-P(2)	105.83(19)
C(23)-C(22)-C(18)	109.6(3)	C(61)-C(62)-C(63)	109.3(2)
C(22)-C(23)-C(25)	110.1(3)	C(69)-C(63)-C(64)	109.5(3)
C(22)-C(23)-C(24)	110.2(3)	C(69)-C(63)-C(62)	109.6(3)
C(25)-C(23)-C(24)	108.6(3)	C(64)-C(63)-C(62)	109.8(3)
C(16)-C(24)-C(23)	109.2(2)	C(63)-C(64)-C(65)	109.7(3)
C(23)-C(25)-C(20)	109.7(3)	C(67)-C(65)-C(64)	109.5(3)
C(31)-C(26)-C(35)	109.3(2)	C(67)-C(65)-C(66)	110.2(3)
C(31)-C(26)-C(27)	109.2(2)	C(64)-C(65)-C(66)	109.0(3)
C(35)-C(26)-C(27)	107.9(2)	C(65)-C(66)-C(61)	109.8(2)
C(31)-C(26)-P(1)	113.47(19)	C(65)-C(67)-C(68)	109.1(3)
C(35)-C(26)-P(1)	110.4(2)	C(67)-C(68)-C(69)	109.9(3)
C(27)-C(26)-P(1)	106.33(19)	C(67)-C(68)-C(70)	109.5(3)
C(28)-C(27)-C(26)	109.9(2)	C(69)-C(68)-C(70)	109.7(3)
C(32)-C(28)-C(29)	109.6(3)	C(63)-C(69)-C(68)	109.3(3)
C(32)-C(28)-C(27)	110.2(3)	C(61)-C(70)-C(68)	109.6(2)
C(29)-C(28)-C(27)	109.1(3)	F(4a)-B(1a)-F(1a)	109.3(2)
C(30)-C(29)-C(28)	109.4(3)	F(4a)-B(1a)-F(3a)	110.1(3)
C(34)-C(30)-C(29)	109.2(3)	F(1a)-B(1a)-F(3a)	109.2(3)
C(34)-C(30)-C(31)	109.2(3)	F(4a)-B(1a)-F(2a)	108.5(2)
C(29)-C(30)-C(31)	110.2(3)	F(1a)-B(1a)-F(2a)	110.5(3)
C(30)-C(31)-C(26)	109.7(2)	F(3a)-B(1a)-F(2a)	109.2(3)
C(28)-C(32)-C(33)	109.2(2)	F(4b)-B(1b)-F(1b)	109.5(3)
C(35)-C(33)-C(32)	109.7(3)	F(4b)-B(1b)-F(3b)	109.5(3)
C(35)-C(33)-C(34)	109.7(3)	F(1b)-B(1b)-F(3b)	109.7(3)
C(32)-C(33)-C(34)	109.1(3)	F(4b)-B(1b)-F(2b)	109.4(3)
C(30)-C(34)-C(33)	109.6(3)	F(1b)-B(1b)-F(2b)	109.4(3)
C(33)-C(35)-C(26)	110.1(2)	F(3b)-B(1b)-F(2b)	109.3(3)
N(2)-Au(2)-C(50)	81.47(11)	F(8a)-B(2a)-F(7a)	110.1(3)
N(2)-Au(2)-Cl(2)	175.27(7)	F(8a)-B(2a)-F(6a)	109.2(3)
C(50)-Au(2)-Cl(2)	94.12(9)	F(7a)-B(2a)-F(6a)	108.2(3)
N(2)-Au(2)-P(2)	85.03(7)	F(8a)-B(2a)-F(5a)	110.1(3)
C(50)-Au(2)-P(2)	166.50(9)	F(7a)-B(2a)-F(5a)	109.8(3)
Cl(2)-Au(2)-P(2)	99.39(3)	F(6a)-B(2a)-F(5a)	109.5(3)
C(36)-P(2)-C(51)	107.38(13)	F(7b)-B(2b)-F(5b)	110.4(3)
C(36)-P(2)-C(61)	107.90(13)	F(7b)-B(2b)-F(6b)	110.4(3)
C(51)-P(2)-C(61)	117.93(13)	F(5b)-B(2b)-F(6b)	110.1(3)
C(36)-P(2)-Au(2)	96.66(9)	F(7b)-B(2b)-F(8b)	109.3(3)
C(51)-P(2)-Au(2)	112.67(10)	F(5b)-B(2b)-F(8b)	108.6(3)
C(61)-P(2)-Au(2)	111.89(9)	F(6b)-B(2b)-F(8b)	108.0(3)

Table S9. Torsion angles (°) with standard uncertainties in parentheses.

C(26)-P(1)-C(1)-C(2)	67.1(3)	C(51)-P(2)-C(36)-C(37)	-61.0(3)
C(16)-P(1)-C(1)-C(2)	-60.3(3)	C(61)-P(2)-C(36)-C(37)	67.0(3)
Au(1)-P(1)-C(1)-C(2)	-178.7(2)	Au(2)-P(2)-C(36)-C(37)	-177.3(2)
C(26)-P(1)-C(1)-C(6)	-111.3(2)	C(51)-P(2)-C(36)-C(41)	119.0(2)
C(16)-P(1)-C(1)-C(6)	121.3(2)	C(61)-P(2)-C(36)-C(41)	-112.9(2)
Au(1)-P(1)-C(1)-C(6)	3.0(2)	Au(2)-P(2)-C(36)-C(41)	2.7(2)
C(6)-C(1)-C(2)-C(3)	-1.1(4)	C(41)-C(36)-C(37)-C(38)	-1.5(4)
P(1)-C(1)-C(2)-C(3)	-179.4(2)	P(2)-C(36)-C(37)-C(38)	178.6(2)
C(1)-C(2)-C(3)-C(4)	1.2(5)	C(36)-C(37)-C(38)-C(39)	2.5(5)
C(2)-C(3)-C(4)-C(5)	0.1(5)	C(37)-C(38)-C(39)-C(40)	-1.4(5)
C(3)-C(4)-C(5)-C(7)	178.1(3)	C(38)-C(39)-C(40)-C(41)	-0.5(5)
C(3)-C(4)-C(5)-C(6)	-1.4(5)	C(38)-C(39)-C(40)-C(42)	178.8(3)
C(9)-N(1)-C(6)-C(5)	-1.0(4)	C(44)-N(2)-C(41)-C(40)	-0.6(4)
Au(1)-N(1)-C(6)-C(5)	178.8(2)	Au(2)-N(2)-C(41)-C(40)	177.3(2)
C(9)-N(1)-C(6)-C(1)	178.6(3)	C(44)-N(2)-C(41)-C(36)	179.6(3)
Au(1)-N(1)-C(6)-C(1)	-1.6(4)	Au(2)-N(2)-C(41)-C(36)	-2.5(4)
C(7)-C(5)-C(6)-N(1)	1.6(4)	C(42)-C(40)-C(41)-N(2)	2.3(4)
C(4)-C(5)-C(6)-N(1)	-178.8(3)	C(39)-C(40)-C(41)-N(2)	-178.4(3)
C(7)-C(5)-C(6)-C(1)	-178.0(3)	C(42)-C(40)-C(41)-C(36)	-177.8(3)
C(4)-C(5)-C(6)-C(1)	1.6(4)	C(39)-C(40)-C(41)-C(36)	1.4(4)
C(2)-C(1)-C(6)-N(1)	-179.9(3)	C(37)-C(36)-C(41)-N(2)	179.3(3)
P(1)-C(1)-C(6)-N(1)	-1.4(4)	P(2)-C(36)-C(41)-N(2)	-0.7(4)
C(2)-C(1)-C(6)-C(5)	-0.4(4)	C(37)-C(36)-C(41)-C(40)	-0.5(4)
P(1)-C(1)-C(6)-C(5)	178.1(2)	P(2)-C(36)-C(41)-C(40)	179.5(2)
C(6)-C(5)-C(7)-C(8)	-0.7(5)	C(41)-C(40)-C(42)-C(43)	-1.4(5)
C(4)-C(5)-C(7)-C(8)	179.7(3)	C(39)-C(40)-C(42)-C(43)	179.3(3)
C(5)-C(7)-C(8)-C(9)	-0.7(5)	C(40)-C(42)-C(43)-C(44)	-1.3(5)
C(6)-N(1)-C(9)-C(8)	-0.5(4)	C(41)-N(2)-C(44)-C(43)	-2.2(4)
Au(1)-N(1)-C(9)-C(8)	179.7(2)	Au(2)-N(2)-C(44)-C(43)	179.8(2)
C(6)-N(1)-C(9)-C(10)	178.5(3)	C(41)-N(2)-C(44)-C(45)	176.6(2)
Au(1)-N(1)-C(9)-C(10)	-1.3(3)	Au(2)-N(2)-C(44)-C(45)	-1.4(3)
C(7)-C(8)-C(9)-N(1)	1.4(5)	C(42)-C(43)-C(44)-N(2)	3.1(5)
C(7)-C(8)-C(9)-C(10)	-177.5(3)	C(42)-C(43)-C(44)-C(45)	-175.5(3)
N(1)-C(9)-C(10)-C(11)	-177.6(3)	N(2)-C(44)-C(45)-C(50)	0.8(4)
C(8)-C(9)-C(10)-C(11)	1.4(5)	C(43)-C(44)-C(45)-C(50)	179.4(3)
N(1)-C(9)-C(10)-C(15)	1.0(4)	N(2)-C(44)-C(45)-C(46)	-177.4(3)
C(8)-C(9)-C(10)-C(15)	180.0(3)	C(43)-C(44)-C(45)-C(46)	1.2(5)
C(15)-C(10)-C(11)-C(12)	0.7(5)	C(50)-C(45)-C(46)-C(47)	0.0(4)
C(9)-C(10)-C(11)-C(12)	179.3(3)	C(44)-C(45)-C(46)-C(47)	178.1(3)
C(10)-C(11)-C(12)-C(13)	0.8(5)	C(45)-C(46)-C(47)-C(48)	1.1(5)
C(11)-C(12)-C(13)-C(14)	-1.3(5)	C(46)-C(47)-C(48)-C(49)	-0.7(5)
C(12)-C(13)-C(14)-C(15)	0.2(5)	C(47)-C(48)-C(49)-C(50)	-0.7(5)
C(13)-C(14)-C(15)-C(10)	1.4(5)	C(48)-C(49)-C(50)-C(45)	1.8(4)

C(13)-C(14)-C(15)-Au(1)	-178.9(2)	C(48)-C(49)-C(50)-Au(2)	-178.1(2)
C(11)-C(10)-C(15)-C(14)	-1.9(4)	C(46)-C(45)-C(50)-C(49)	-1.4(4)
C(9)-C(10)-C(15)-C(14)	179.5(3)	C(44)-C(45)-C(50)-C(49)	-179.6(3)
C(11)-C(10)-C(15)-Au(1)	178.4(2)	C(46)-C(45)-C(50)-Au(2)	178.5(2)
C(9)-C(10)-C(15)-Au(1)	-0.3(3)	C(44)-C(45)-C(50)-Au(2)	0.3(3)
C(1)-P(1)-C(16)-C(17)	173.6(2)	C(36)-P(2)-C(51)-C(60)	51.4(3)
C(26)-P(1)-C(16)-C(17)	52.7(2)	C(61)-P(2)-C(51)-C(60)	-70.6(3)
Au(1)-P(1)-C(16)-C(17)	-80.1(2)	Au(2)-P(2)-C(51)-C(60)	156.7(2)
C(1)-P(1)-C(16)-C(21)	-68.9(2)	C(36)-P(2)-C(51)-C(52)	174.5(2)
C(26)-P(1)-C(16)-C(21)	170.3(2)	C(61)-P(2)-C(51)-C(52)	52.5(3)
Au(1)-P(1)-C(16)-C(21)	37.5(2)	Au(2)-P(2)-C(51)-C(52)	-80.2(2)
C(1)-P(1)-C(16)-C(24)	50.5(2)	C(36)-P(2)-C(51)-C(56)	-67.6(2)
C(26)-P(1)-C(16)-C(24)	-70.3(2)	C(61)-P(2)-C(51)-C(56)	170.4(2)
Au(1)-P(1)-C(16)-C(24)	156.9(2)	Au(2)-P(2)-C(51)-C(56)	37.6(2)
C(21)-C(16)-C(17)-C(18)	58.5(3)	C(60)-C(51)-C(52)-C(53)	-61.1(4)
C(24)-C(16)-C(17)-C(18)	-60.3(3)	C(56)-C(51)-C(52)-C(53)	57.6(4)
P(1)-C(16)-C(17)-C(18)	173.6(2)	P(2)-C(51)-C(52)-C(53)	172.7(3)
C(16)-C(17)-C(18)-C(19)	-58.9(3)	C(51)-C(52)-C(53)-C(57)	61.7(4)
C(16)-C(17)-C(18)-C(22)	61.2(4)	C(51)-C(52)-C(53)-C(54)	-58.6(4)
C(22)-C(18)-C(19)-C(20)	-59.5(4)	C(57)-C(53)-C(54)-C(55)	-59.2(4)
C(17)-C(18)-C(19)-C(20)	60.2(3)	C(52)-C(53)-C(54)-C(55)	60.2(4)
C(18)-C(19)-C(20)-C(25)	59.1(3)	C(53)-C(54)-C(55)-C(59)	58.7(3)
C(18)-C(19)-C(20)-C(21)	-60.4(4)	C(53)-C(54)-C(55)-C(56)	-61.0(4)
C(19)-C(20)-C(21)-C(16)	60.3(3)	C(54)-C(55)-C(56)-C(51)	60.6(3)
C(25)-C(20)-C(21)-C(16)	-59.6(3)	C(59)-C(55)-C(56)-C(51)	-59.3(3)
C(17)-C(16)-C(21)-C(20)	-59.5(3)	C(60)-C(51)-C(56)-C(55)	59.6(3)
C(24)-C(16)-C(21)-C(20)	59.6(3)	C(52)-C(51)-C(56)-C(55)	-59.1(3)
P(1)-C(16)-C(21)-C(20)	-177.2(2)	P(2)-C(51)-C(56)-C(55)	-177.6(2)
C(19)-C(18)-C(22)-C(23)	59.3(4)	C(54)-C(53)-C(57)-C(58)	59.8(4)
C(17)-C(18)-C(22)-C(23)	-61.0(4)	C(52)-C(53)-C(57)-C(58)	-60.7(4)
C(18)-C(22)-C(23)-C(25)	-59.5(3)	C(53)-C(57)-C(58)-C(59)	-59.9(4)
C(18)-C(22)-C(23)-C(24)	60.3(4)	C(53)-C(57)-C(58)-C(60)	60.1(4)
C(17)-C(16)-C(24)-C(23)	58.7(3)	C(57)-C(58)-C(59)-C(55)	59.7(3)
C(21)-C(16)-C(24)-C(23)	-60.5(3)	C(60)-C(58)-C(59)-C(55)	-61.4(4)
P(1)-C(16)-C(24)-C(23)	-178.1(2)	C(54)-C(55)-C(59)-C(58)	-59.2(3)
C(22)-C(23)-C(24)-C(16)	-59.0(4)	C(56)-C(55)-C(59)-C(58)	60.4(4)
C(25)-C(23)-C(24)-C(16)	61.7(3)	C(52)-C(51)-C(60)-C(58)	58.8(4)
C(22)-C(23)-C(25)-C(20)	59.1(3)	C(56)-C(51)-C(60)-C(58)	-60.4(4)
C(24)-C(23)-C(25)-C(20)	-61.6(3)	P(2)-C(51)-C(60)-C(58)	-177.3(2)
C(19)-C(20)-C(25)-C(23)	-58.8(4)	C(59)-C(58)-C(60)-C(51)	61.6(4)
C(21)-C(20)-C(25)-C(23)	60.7(4)	C(57)-C(58)-C(60)-C(51)	-58.9(4)
C(1)-P(1)-C(26)-C(31)	-71.7(2)	C(36)-P(2)-C(61)-C(62)	-66.4(2)
C(16)-P(1)-C(26)-C(31)	49.6(3)	C(51)-P(2)-C(61)-C(62)	55.4(3)
Au(1)-P(1)-C(26)-C(31)	-176.0(2)	Au(2)-P(2)-C(61)-C(62)	-171.5(2)
C(1)-P(1)-C(26)-C(35)	165.2(2)	C(36)-P(2)-C(61)-C(70)	170.3(2)

C(16)-P(1)-C(26)-C(35)	-73.5(2)	C(51)-P(2)-C(61)-C(70)	-67.9(2)
Au(1)-P(1)-C(26)-C(35)	60.9(2)	Au(2)-P(2)-C(61)-C(70)	65.2(2)
C(1)-P(1)-C(26)-C(27)	48.4(2)	C(36)-P(2)-C(61)-C(66)	53.4(2)
C(16)-P(1)-C(26)-C(27)	169.7(2)	C(51)-P(2)-C(61)-C(66)	175.2(2)
Au(1)-P(1)-C(26)-C(27)	-56.0(2)	Au(2)-P(2)-C(61)-C(66)	-51.7(2)
C(31)-C(26)-C(27)-C(28)	-59.5(3)	C(70)-C(61)-C(62)-C(63)	-59.4(3)
C(35)-C(26)-C(27)-C(28)	59.2(3)	C(66)-C(61)-C(62)-C(63)	59.1(3)
P(1)-C(26)-C(27)-C(28)	177.7(2)	P(2)-C(61)-C(62)-C(63)	177.0(2)
C(26)-C(27)-C(28)-C(32)	-60.0(3)	C(61)-C(62)-C(63)-C(69)	60.5(3)
C(26)-C(27)-C(28)-C(29)	60.4(3)	C(61)-C(62)-C(63)-C(64)	-59.8(3)
C(32)-C(28)-C(29)-C(30)	60.3(3)	C(69)-C(63)-C(64)-C(65)	-60.0(3)
C(27)-C(28)-C(29)-C(30)	-60.4(3)	C(62)-C(63)-C(64)-C(65)	60.4(4)
C(28)-C(29)-C(30)-C(34)	-59.8(3)	C(63)-C(64)-C(65)-C(67)	60.1(3)
C(28)-C(29)-C(30)-C(31)	60.2(3)	C(63)-C(64)-C(65)-C(66)	-60.5(3)
C(34)-C(30)-C(31)-C(26)	60.6(3)	C(67)-C(65)-C(66)-C(61)	-59.8(3)
C(29)-C(30)-C(31)-C(26)	-59.3(3)	C(64)-C(65)-C(66)-C(61)	60.3(3)
C(35)-C(26)-C(31)-C(30)	-59.4(3)	C(62)-C(61)-C(66)-C(65)	-60.0(3)
C(27)-C(26)-C(31)-C(30)	58.5(3)	C(70)-C(61)-C(66)-C(65)	59.2(3)
P(1)-C(26)-C(31)-C(30)	176.9(2)	P(2)-C(61)-C(66)-C(65)	177.2(2)
C(29)-C(28)-C(32)-C(33)	-60.6(3)	C(64)-C(65)-C(67)-C(68)	-59.8(3)
C(27)-C(28)-C(32)-C(33)	59.5(3)	C(66)-C(65)-C(67)-C(68)	60.1(3)
C(28)-C(32)-C(33)-C(35)	-59.9(3)	C(65)-C(67)-C(68)-C(69)	60.1(3)
C(28)-C(32)-C(33)-C(34)	60.3(3)	C(65)-C(67)-C(68)-C(70)	-60.5(3)
C(29)-C(30)-C(34)-C(33)	60.0(3)	C(64)-C(63)-C(69)-C(68)	59.8(3)
C(31)-C(30)-C(34)-C(33)	-60.6(3)	C(62)-C(63)-C(69)-C(68)	-60.8(3)
C(35)-C(33)-C(34)-C(30)	59.9(3)	C(67)-C(68)-C(69)-C(63)	-60.1(3)
C(32)-C(33)-C(34)-C(30)	-60.3(3)	C(70)-C(68)-C(69)-C(63)	60.4(3)
C(32)-C(33)-C(35)-C(26)	61.1(3)	C(62)-C(61)-C(70)-C(68)	59.1(3)
C(34)-C(33)-C(35)-C(26)	-58.8(3)	C(66)-C(61)-C(70)-C(68)	-59.9(3)
C(31)-C(26)-C(35)-C(33)	58.6(3)	P(2)-C(61)-C(70)-C(68)	-175.2(2)
C(27)-C(26)-C(35)-C(33)	-60.1(3)	C(67)-C(68)-C(70)-C(61)	61.3(3)
P(1)-C(26)-C(35)-C(33)	-175.9(2)	C(69)-C(68)-C(70)-C(61)	-59.5(3)

8 References

(1) (a) Mao, L.; Moriuchi, T.; Sakurai, H.; Fujii, H.; Hirao, T. New tridentate cyclometalated platinum(II) and palladium(II) complexes of N,2-diphenyl-8-quinolinamine: syntheses, crystal structures, and photophysical properties. *Tetrahedron Lett.* **2005**, *46*, 8419-8422; (b) Wickramasinghe, L. D.; Zhou, R. W.; Zong, R. F.; Vo, P.; Gagnon, K. J.; Thummel, R. P. Iron Complexes of Square Planar Tetradentate Polypyridyl-Type Ligands as Catalysts for Water Oxidation. *J. Am. Chem. Soc.* **2015**, *137*, 13260-13263.

(2) Jeremias, N.; Mohr, L. M.; Bach, T. Intermolecular [2+2] Photocycloaddition of α,β-Unsaturated Sulfones: Catalyst-Free Reaction and Catalytic Variants. *Org. Lett.* **2021**, *23*, 5674-5678.

(3) Lu, X.-L.; Shannon, M.; Peng, X.-S.; Wong, H. N. C. Stereospecific Iron-Catalyzed Carbon(sp^2)–Carbon(sp^3) Cross-Coupling with Alkyllithium and Alkenyl Iodides. *Org. Lett.* **2019**, *21*, 2546-2549.

(4) Beucher, H.; Kumar, S.; Kumar, R.; Merino, E.; Hu, W.-H.; Stemmler, G.; Cuesta-Galisteo, S.; González, J. A.; Bezinge, L.; Jagielski, J.; Shih, C.-J.; Nevado, C. Phosphorescent κ^3-(N^C^C)-Gold(III) Complexes: Synthesis, Photophysics, Computational Studies and Application to Solution-Processable OLEDs. *Chem. Eur. J.* **2020**, *26*, 17604-17612.

(5) (a) Mori, M.; Suzuki, T. Mixed-ligand platinum(II) complexes containing 2-(2′-pyridyl)phenyl and 8-quinolylphosphines: Synthesis and molecular structures in the crystals and in solution. *Inorg. Chim. Acta* **2020**, *512*, 119862; (b) Mori, M.; Sunatsuki, Y.; Suzuki, T. Sterically Demanding 8-(Diphenylphosphino)quinoline Complexes of Group 10 Metal(II): Synthesis, Crystal Structures, and Properties in Solution. *Inorg. Chem.* **2020**, *59*, 18225.

(6) Lord, A.-M.; Mahon, M. F.; Lloyd, M. D.; Threadgill, M. D. Design, Synthesis, and Evaluation in Vitro of Quinoline-8-carboxamides, a New Class of Poly(adenosine-diphosphate-ribose)polymerase-1 (PARP-1) Inhibitor. *J. Med. Chem.* **2009**, *52*, 868-877.

(7) Ronellenfitsch, M.; Wadepohl, H.; Enders, M. Chromium Aryl Complexes with N-Donor Ligands as Catalyst Precursors for Selective Ethylene Trimerization. *Organometallics* **2014**, *33*, 5758-5766.

(8) Martín, J.; Schörgenhumer, J.; Biedrzycki, M.; Nevado, C. (P^N^C) Ligands to Stabilize Gold(III): A Straightforward Access to Hydroxo, Formate, and Hydride Complexes. *Inorg. Chem.* **2024**, *63*, 8390-8396.

(9) McCallum, T. Heart of gold: enabling ligands for oxidative addition of haloorganics in Au(I)/Au(III) catalysed cross-coupling reactions. *Org. Biomol. Chem.* **2023**, *21*, 1629-1646.

(10) Rocchigiani, L.; Fernandez-Cestau, J.; Budzelaar, P. H. M.; Bochmann, M. Reductive Elimination Leading to C-C Bond Formation in Gold(III) Complexes: A Mechanistic and Computational Study. *Chem. Eur. J.* **2018**, *24*, 8893-8903.

(11) Rigaku Oxford Diffraction, *CrysAlisPro* Software System, Version 1.171.40.53, Rigaku Corporation, Oxford, UK, **2019**.

(12) Clark, R.C.; Reid, J.S. The analytical calculation of absorption in multifaceted crystals. *Acta Crystallogr. Sect. A*, **1995**, *51*, 887.

(13) (a) Sheldrick, G.M. A short history of SHELX. *Acta Crystallogr. Sect. A*, **2008**, *64*, 112-122; (b) Sheldrick, G. M. SHELXT – Integrated space-group and crystal-structure determination. *Acta Crystallogr. Sect. A*, **2015**, *71*, 3-8.

(14) Spek, A. PLATON SQUEEZE: a tool for the calculation of the disordered solvent contribution to the calculated structure factors. *Acta Crystallogr. Sect. C* **2015**, *71*, 9-18.

(15) (a) Maslen, E.N.; Fox, A.G.; O'Keefe, M.A. in 'International Tables for Crystallography', Ed. A.J.C. Wilson, Kluwer Academic Publishers, Dordrecht, **1992**, Vol. C, Table 6.1.1.1, pp. 477-486; (b) Creagh, D.C.; McAuley, W.J. *ibid*. Table 4.2.6.8, pp. 219-222; (c) Creagh, D.C.; Hubbell, J.H. *ibid*. Table 4.2.4.3, pp. 200-206.

(16) Stewart, R. F.; Davidson, E. R.; Simpson, W. T. Coherent X-Ray Scattering for Hydrogen Atom in Hydrogen Molecule. *J. Chem. Phys.* **1965**, *42*, 3175-3187.

(17) Ibers, J. A.; Hamilton, W. C. Dispersion Corrections + Crystal Structure Refinements. *Acta Crystallogr.* **1964**, *17*, 781-782.

(18) Sheldrick, G. M. Crystal structure refinement with SHELXL. *Acta Crystallogr. Sect. C*, **2015**, *71*, 3-8.
